# Combining Rational and Random Strategies in β-Glucosidase Zm-p60.1 Protein Library Construction

**DOI:** 10.1371/journal.pone.0108292

**Published:** 2014-09-26

**Authors:** Dušan Turek, Pavel Klimeš, Pavel Mazura, Břetislav Brzobohatý

**Affiliations:** Laboratory of Plant Molecular Biology, Institute of Biophysics AS CR, v.v.i. CEITEC – Central European Institute of Technology, Mendel University in Brno, Brno, Czech Republic; Imperial College London, United Kingdom.

## Abstract

Saturation mutagenesis is a cornerstone technique in protein engineering because of its utility (in conjunction with appropriate analytical techniques) for assessing effects of varying residues at selected positions on proteins’ structures and functions. Site-directed mutagenesis with degenerate primers is the simplest and most rapid saturation mutagenesis technique. Thus, it is highly appropriate for assessing whether or not variation at certain sites is permissible, but not necessarily the most time- and cost-effective technique for detailed assessment of variations’ effects. Thus, in the presented study we applied the technique to randomize position W373 in β-glucosidase Zm-p60.1, which is highly conserved among β-glucosidases. Unexpectedly, β-glucosidase activity screening of the generated variants showed that most variants were active, although they generally had significantly lower activity than the wild type enzyme. Further characterization of the library led us to conclude that a carefully selected combination of randomized codon-based saturation mutagenesis and site-directed mutagenesis may be most efficient, particularly when constructing and investigating randomized libraries with high fractions of positive hits.

## Introduction

There are several important considerations when designing randomized libraries of protein variants. The mutagenesis method used should produce the required kind and range of variability. It should also be sufficiently reliable to enable robust predictions of the libraries’ composition and completeness, which are required to optimize screening efforts. For oligonucleotide-directed mutagenesis, equations have been derived to predict how complete a given library is likely to be, and the size of a library required for a given probability of 100% coverage, or other selected threshold [1]. These formulas are widely applied in the design of mutagenesis and screening experiments, but the predictions they generate should be treated cautiously as they are generally based on probabilities of randomly drawing subsets from populations.

In practice, every method for generating protein variants has strengths, weaknesses and biases. Thus, ideally parameters describing the performance of mutagenesis methods should be empirically estimated and incorporated into the formulas to avoid underestimation of required screening efforts [2]. The screening is usually the most expensive part of the overall effort, so it is highly desirable to identify the most appropriate screening scale. Thus, the common goal of these procedures is to identify the optimal scale, at which risks of missing positive hits are acceptably low and numbers of clones are not prohibitively high for screening.

Frequently, the most important objective is to avoid missing rare “positive hits”. However, in some cases (such as the one described here) there may be numerous “positive hits”. Thus, retaining variability may be more important and this may have significant implications for mutation generation and screening strategies.

β-glucosidase Zm-p60.1 is an enzyme that was originally identified in maize, where it catalyzes the release of active cytokinin from glucoside transport and storage forms, thereby playing a key role in hormonal regulation [3], [4]. Zm-p60.1 is a glucohydrolase (E.C.:3.2.1.21) of the glycoside hydrolase 1 family (GH1). Amino acid residues involved in its substrate interactions and catalysis have been identified using site-directed mutagenesis and X-ray crystallography [5]–[9]. A shared feature of GH1 members, and other (α/β)_8_ proteins, is an active site formed by four variable loops extending from the conserved structure of the protein. A key structural determinant of substrate specificity in Zm-p60.1 – repeatedly confirmed in structural, docking and kinetic studies – is the F193–F200–W373–F461 cluster [5], [7], [9].

One of the findings of the cited studies is that W373 plays an important role in stabilizing the aglycone part of the substrate in the entrance of the active site [5], [8], [9], [10]. Residues in corresponding positions often play similar roles in other glucosidases [11], although the amino acid composition is not preserved in all cases [12]. Furthermore, there are complex structure-activity relationships in the region. For example, mutation W373K, inspired by the composition of the homologous enzyme Bgl4∶1 in *Brassica napus*, severely impairs catalytic activity [7], [11]. In addition, replacement of Zm-p60.1′s hydrophobic cluster with the *Brassicaceae* consensus cluster (F193A/F200K/W373K/F461L) leads to extensive structural alterations and almost complete abolition of enzymatic activity [7]. These results strongly suggest that a specific combination of amino acids in this region is important for both specificity and structural stability. This hypothesis has been confirmed by experiments involving random mutagenesis close to W373K, and the identification of a variant (P372T/W373K/M376L) with partially rescued catalytic activity and changed specificity [8].

In the presented study we further explored the effects of varying position W373, and unexpectedly found that numerous generated variants had enzymatic activity. This finding led to a detailed consideration of the optimal combination of rational and random strategies for constructing β-glucosidase Zm-p60.1 protein libraries for further structural-functional analyses.

## Results and Discussion

**Figure 1:**
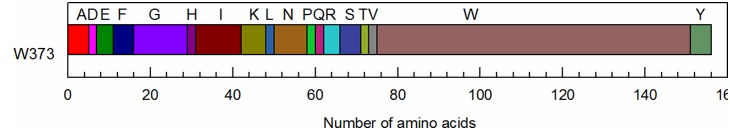
Bioinformatics-based estimation of variability in the set of 167 β-glycosidases defined by Zhao et al. [13] at the position corresponding to W373 in β-glucosidase Zm-p60.1. The whole alignment is included as File S2 and S3.

Published data [14] were used to define and align a set of 167 related glycosidases of the GH1 family for bioinformatics analysis and determination of variability at the W373 position (Figure 1). The whole alignment is included as File S2 and File S3.The alignment clearly shows that W373 is frequently conserved at the position, but other amino acids are present at related positions in some homologous proteins. Furthermore, higher variability has been recently detected at the position in the closely phylogenetically related group of β-glucosidases [13] than in previous analysis [7]. Therefore, we applied saturation mutagenesis followed by activity screening to identify other amino acid residues at the W373 position that could be compatible with the composition of β-glucosidase Zm-p60.1 and catalytic effects of the substitutions.

Several codon randomization schemes [15], [16] can be used to generate variants of proteins with different sets of amino acids at selected positions. For random saturation mutagenesis (A/C/G/T, A/C/G/T, G/T) or NNN (A/C/G/T, A/C/G/T, A/C/G/T) codon randomization are widely applied to introduce all amino acids. NNN randomization is particularly valuable when comprehensive variation is required and codon selection could influence the transcription and expression machinery, as it uses all 64 codons. However, this also inevitably maximizes redundancy and the size of libraries for screening. Theoretically, there will be 95% probability that an NNN-generated library includes all possible amino acid variants when it contains 240 clones.

NNK randomization is the most widely used procedure for saturation mutagenesis because it provides all amino acids and only one stop codon. Thus, it generates significantly smaller libraries for sequencing than NNN randomization. Theoretically, there will be 95% probability that an NNK-generated library includes all possible amino acid variants when it contains 171 clones.

However, in our Zm-p60.1 engineering project we initially used NNM (A/C/G/T, A/C/G/T, A/C/) codon randomization and functional screening of *E. coli* clones [8]. NNM randomization provides similar performance to NNK, but the resulting libraries do not contain variants with Trp or Met residues at the varied positions. If there is a Met or Trp in the starting structure (as in our case) this could be beneficial, because all variants generated will be new. Mutagenesis by NNM codon degeneration yields 32 codon variants, encoding 18 amino acids and 2 stop codons. Since the number of amino acids is reduced, the screening requirements are also lower. Theoretically, there will be 95% probability that an NNK-generated library includes all possible amino acid variants when it contains 163 clones.

On the basis of our previous work we expected to find one or a few functional variants of the weakly catalyzing mutant W373K [7], [8]. However, most clones proved to be active, for example 20 of 21 clones initially obtained displayed various levels of β-glucosidase activity. In further research we investigated: a) the theoretical and real composition of the generated library, and b) practical obstacles to the creation of a complete library.

Theoretically, every codon has an equal chance of being present in any clone, resulting in a 30/32 probability of coding codons and 2/32 probability of stop codons at the varied position. However, amino acids in NNM randomization are coded by different numbers of codons, so the probabilities of occurrence at a varied position in the final library are: 1/32 for Cys, Asp, Glu, Phe, His, Lys, Asn, Gln and Tyr; 2/32 for Ala, Gly, Ile, Pro, Thr and Val; 3/32 for Leu, Arg and Ser; 0 for Met and Trp.

A simple calculation of the combined probability even for the most frequent amino acids (3/32)^21^ shows that it is highly improbable that all of the positive clones we obtained represented a single variant, and even the negative clone could have had a stop codon at the varied site. Thus, the identification of new active (and inactive variants) of Zm-p60.1 seemed highly feasible. Furthermore, even with such a small set of variants useful predictions can be obtained of possible library compositions and likely relative proportions of positive hits due to new variants and random multiple occurrences of the same variant.

Thanks to the small size of the library we sequenced all 21 clones. We identified 10 variants at the 373 position: Leu (five copies); Pro and Arg (each in three copies); Ser, Ala and Gly (each in two copies); His, Thr, Asp and Phe (all in one copy). Thus, even in this small sample there was a clear correlation between the prevalence of amino acids at the varied site and the numbers of corresponding codons used in the randomization procedure.

The clone with no glucosidase activity proved to be a proline variant with an additional stop codon introduced at position 206 by an unknown error in the mutagenesis procedure.

**Figure 2:**
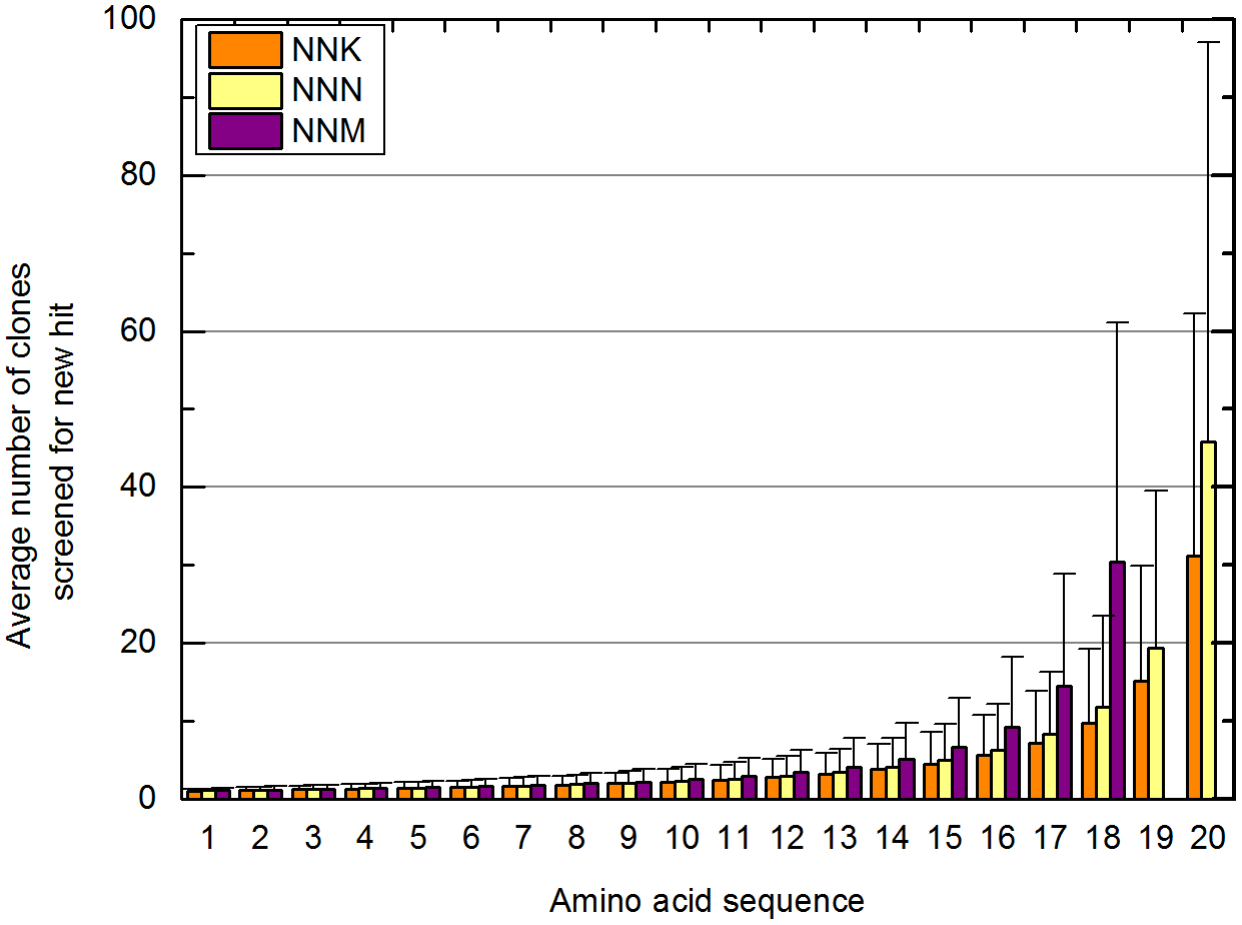
Simulation of random clone picking in randomized codon based saturation mutagenesis and calculation of the average number of clones needed to detect a new variant in libraries generated by NNK, NNN and NNM randomization.

The results show that there are at least 10 active variants of the β-glucosidase, and raise the question whether there are other active variants at position W373 or all variants are active. The simplest procedure for finding another active variant would have been to broaden the screening and identify positive clones, but this procedure would have been more likely to find variants that had already been identified. When screening libraries with hundreds or thousands of variants and only a few clones have been identified (a much more common scenario) this is not an issue. However, when there are high fractions of positive clones and high numbers of identified mutants, attempting to complete the library by extending the random mutagenesis and screening could be unnecessarily expensive. To demonstrate this problem, we determined theoretical average numbers of clones needed to find new variants and complete the library in computer simulations (Figure 2).

**Figure 3:**
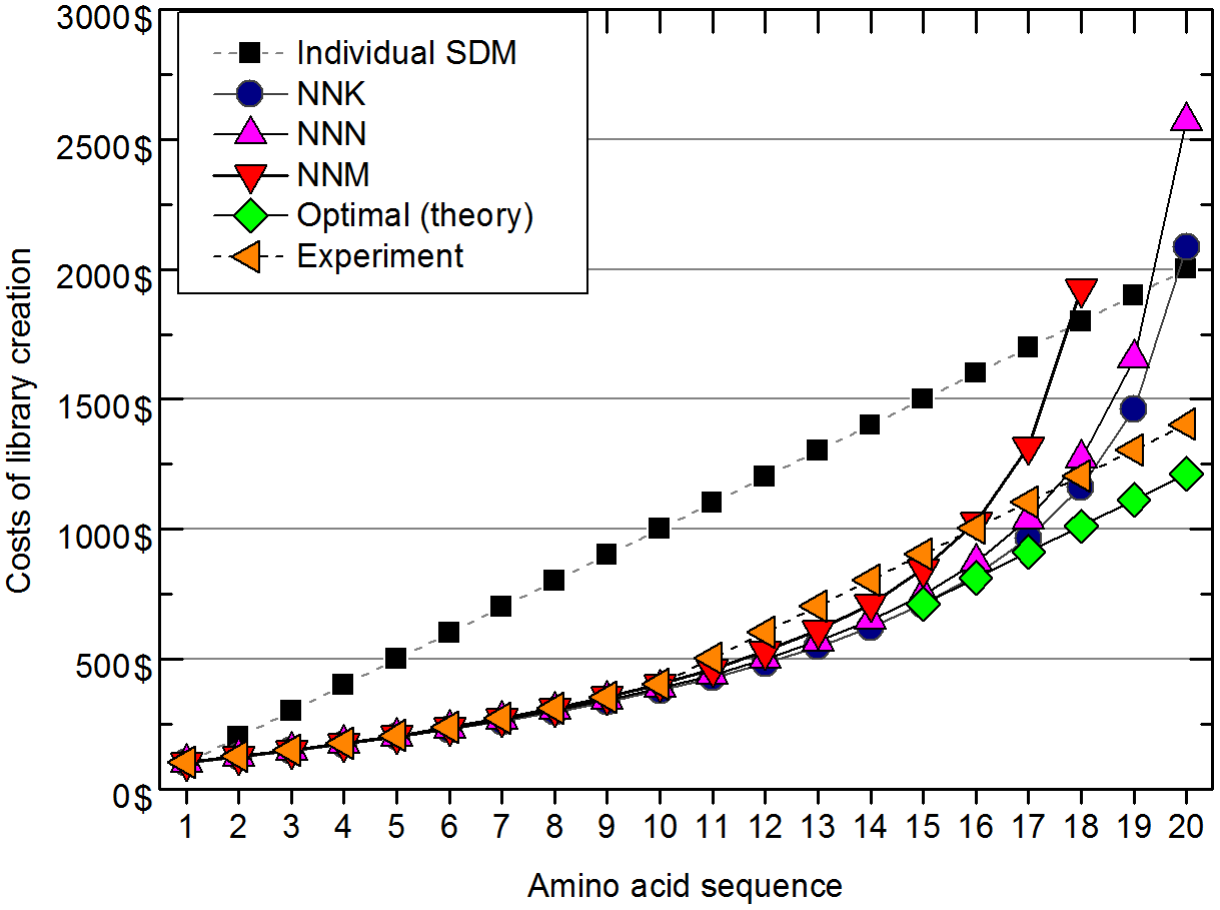
Comparison of projected costs of randomized codon-based saturation mutagenesis library creation and one-by-one site-directed mutagenesis (SDM). Empirically determined results for the combined β-glucosidase Zm-p60.1 mutagenesis approach are also shown (experimental).

The simulations show that there is a sharp increase in numbers of clones needed for the last quarter of library construction, and increases in the variability of clone numbers, using all three codon randomization procedures (NNN, NNM and NNK). These results are reflected in the costs of library creation by saturation mutagenesis. Because of the high probability of finding new variants in the beginning of the screening we propose that an ideal strategy should combine random mutagenesis with site-directed mutagenesis (SDM) to generate missing variants (Figure 3).

Thus, in the mutagenesis of Zm-p60.1 we initially used NNM codon randomization and after finding 10 variants completed the library by using SDM to obtain missing variants.

**Figure 4:**
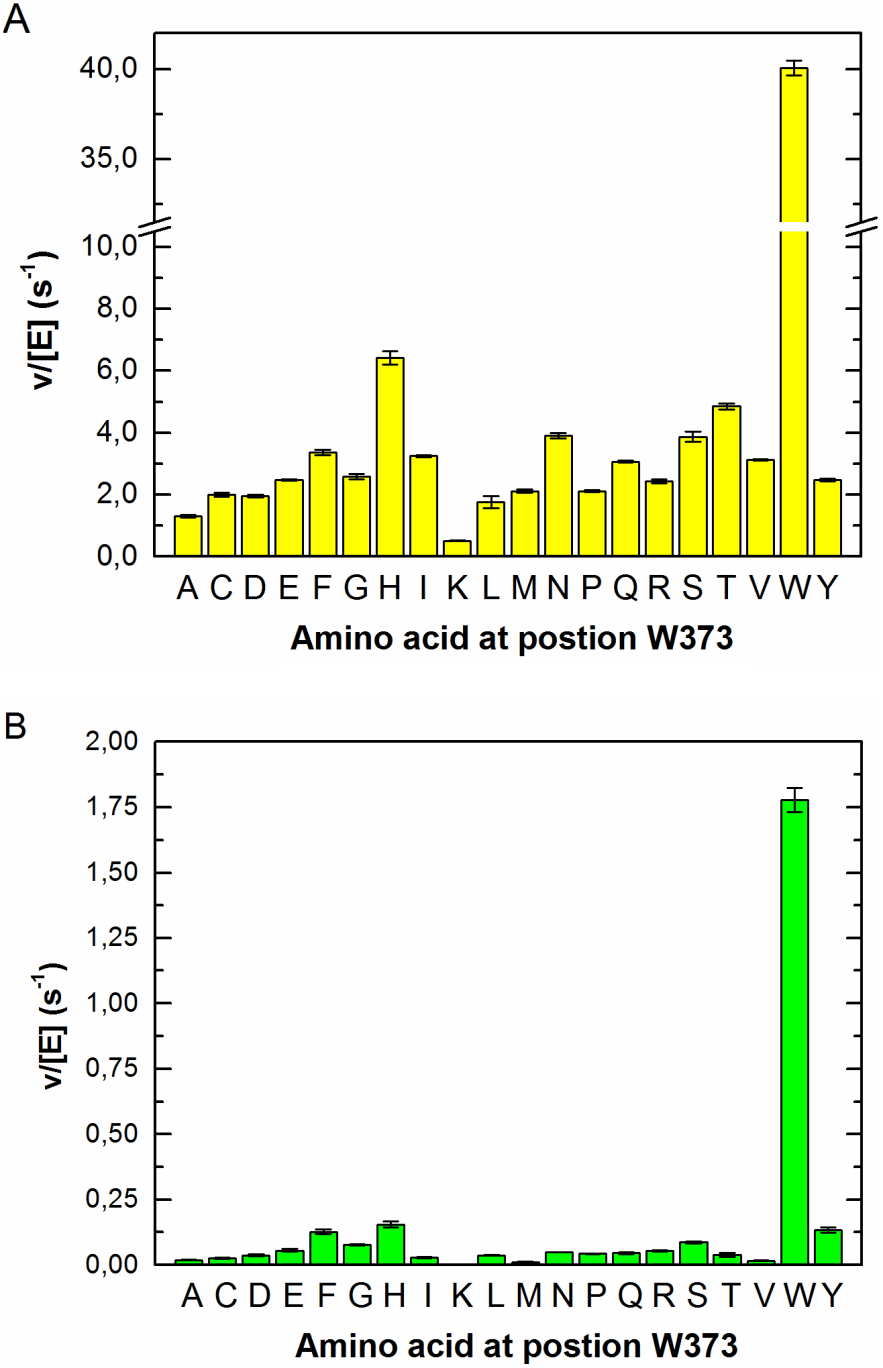
Hydrolysis rates for substrates *p*NPG (A) and *t*ZOG (B) by β-glucosidase Zm-p60.1 variants produced in the course of saturation mutagenesis of the W373position.

The complete library was recloned in the *E. coli* expression strain, all 20 variants at position 373 were expressed, purified to homogeneity and tested for catalytic effect. Our results show that in surprising contrast to the high conservation of Trp it is possible to construct variants that have residual catalytic activity with all amino acid residues at this position. The residual hydrolytic activities of purified enzymes towards the natural substrate *trans*-zeatin-O-β-D-glucopyranoside (*t*ZOG) and artificial substrate *p*-nitrophenyl β-D-glucopyranoside (*p*NPG) were also measured. The *t*ZOG hydrolytic rate constant was low for all mutants, but slightly higher for W373H W373F, W373M, W373Y mutants than for the others. W373H also had slightly higher activity than the other variants towards *p*NPG (Figure 4).

The variants’ *t*ZOG and *p*NPG hydrolytic rate constant profiles also differed, but we do not regard these changes as robust indicators of changes in specificity due to the variants’ low overall activity. These results suggest that the reductions in activity are due to the loss of specific stacking interactions with the tryptophan residue, which cannot be replaced by other residues, not even those with aromatic side chains. To examine effects of the substitutions on the pH optimum we analyzed activities of the most and least active variants (W373H and W373K) in relation to pH. We found no significant difference between the W373K mutant and WT pH profiles, but the pH optimum of the W373H mutant shifted from 5.5 to 6.0 (File S1). These results suggest that mutagenesis at the 373 position can influence the optimum pH of Zm-p60.1, but this effect alone is not the cause of the activity reductions.

All protein variants are sufficiently structurally stable for further analysis of combined effects of amino acid changes in the active site of Zm-p60.1. The extent of functional variability in the entrance part of the active site could be seen in recently published structure of β-primeverosidase which is otherwise very similar to β-glucosidase Zm-p60.1 with an overall RMSD of 1.06 Å in the superimposed structure. Here the hydrophobic cluster F193–F200–W373–F461 in Zm-p60.1 corresponds to G210-L217-A387-L472 in β-primeverosidase. The unique combination of small amino acid residues, and most importantly the absence of tryptophan at the 387 position, forms a large cavity providing the basis for the broad aglycone specificity of β-primeverosidase and possibility to accept aglycones with a bulky disaccharide-glycone part [16].

## Conclusions

Increasing the coverage of variant libraries with a high fraction of positive hits simply by random mutagenesis and/or more extensive screening may be inefficient because the probabilities of detecting variants that have already been detected rather than new ones rapidly increase as libraries approach completion. However, knowledge of the theoretical probabilities of finding new variants and the factors influencing the probabilities can greatly facilitate efforts to identify the most appropriate strategy for further screening (particularly for libraries that are sufficiently small for all variants to be conveniently investigated). The presented study illustrates the efficiency of NNM codon degeneration and finalization by SDM for saturation mutagenesis of β-glucosidase Zm-p60.1.

Randomized codon-based saturation mutagenesis becomes inefficient for finding new variants beyond coverage thresholds that are strongly influenced by differences in frequencies of amino acid substitutions in generated libraries. These differences can be at least partly predicted and included in the library design. However, the reliability and other mutagenesis parameters strongly depend on the procedure used, so observed and predicted frequencies of substitutions may differ due to random errors and false negative clones in the screening. The screening could also be adversely affected by various factors, including low competency of the *E. coli* (or other) expression strains, variations in expression levels of protein variants and mixing of clones when picking colonies. Given all these considerations, a combination of randomized codon-based saturation mutagenesis and standard SDM could be beneficial, especially when a complete library is needed.

In the course of saturation mutagenesis at the W373 position of β-glucosidase Zm-p60.1 all expressed variants were tested for their ability to cleave the natural substrate *t*ZOG and artificial substrate *p*NPG. In surprising contrast to the high conservation at this position all enzyme variants were able to hydrolyze the substrates, albeit at reduced levels (Figure 4). Thus, although their catalytic activity was significantly impaired, we have demonstrated that variation at position 373 is possible, without complete loss of activity, and that variants with mutations at the position could be used in efforts to modulate and explain β-glucosidases’ specificity.

## Materials and Methods

### Bioinformatics analysis

The carbohydrate-active enzymes database (CAZy; http://www.cazy.org) [17] was used to obtain reference information for bioinformatics analysis. A set of 168 β-glycosidase-encoding DNA sequences was selected, as previously described [14], and translated into amino acid sequences. One of them (AtBglu6) was omitted from further analyses due to the presence of stop codons in the sequence. Multiple sequence alignment was performed with the T-Coffee algorithm version 9.01 under default parameters [18]. W373 in Zm-p60.1 was compared with corresponding residues in related proteins and frequencies of exchanges were calculated (using the alignment in File S2, where position 1833 corresponds to the position of W373 in Zm-p60.1 or in File S3 position 18 in detailed view).

The simulation used to calculate theoretical average numbers of clones that needed to be screened for a new hit using each codon degeneration strategy was based on Monte Carlo method (scripts are enclosed as a File S6) implemented in Python 2.7.3 with 1 000 000 iterations simulating random clone picking. The cost-effectiveness of the mutagenesis strategies was compared using estimates for (a) SDM and sequencing based on costs of commercial oligonucleotide synthesis + mutagenesis (by a QuikChange kit) + DNA isolation (by a Qiagen kit) + commercial sequencing (ca. 100 USD per variant in total), and b) screening only, based on DNA isolation (by a Qiagen kit) + commercial sequencing (ca. 20 USD per variant in total).

### Bacterial strains and expression vectors

*E. coli* XJb(DE3) Δ*bgl* (phenotype *bgl-*, constructed by Mahadevan of the Indian Institute of Science, Bangalore, India [19]) was used as a host strain for gene cloning, mutant library construction and screening. Recombinant proteins were produced in the *E. coli* expression strain BL21(DE3)pLysS-T1^R^ (Sigma–Aldrich, St. Louis, MO, USA). All wild-type and engineered variants of *Zm-p60.r* were subcloned into the pRSET A vector (Invitrogen; Carlsbad, CA, USA), in which expression of recombinant protein is under control of the phage T7 promoter and all recombinant enzymes are His-tagged at their N-terminus ends.

### Mutagenesis

For randomized codon-based saturation mutagenesis the “QuikChange” Site-Directed Mutagenesis protocol (Agilent/Stratagene; La Jolla, CA, USA) was applied, with minor adaptations, using the pRSET A::*Zm-p60.r* construct as a template. Plasmid DNA was isolated using a QIAprep Spin Miniprep Kit (Qiagen, USA). A library of Zm-p60.1 mutants with variations at the Trp373 position was created using the following set of primers introducing NNM degeneracy:

5′-cctcctatgggaaatccaNNMatctacatgtaccctgagggc-3′
5′-gccctcagggtacatgtagatKNNtggatttcccataggagg-3′

Mutant strands were synthesized using a 2400 GeneAmp PCR system (PerkinElmer; Waltham, MA, USA), with 60 cycles of denaturation at 95°C for 2 min, annealing at 55°C for 20 s and extension at 65°C for 2 min 18 s. *Dpn* I restriction was then applied to avoid amplification of the template. Competent *E. coli* cells were transformed, the transformants were grown and selected. The number of colonies was low due to the low transformation efficiency of the engineered *E. coli* strain. Finally, to complete the library we created missing variants using the QuikChange kit again in individual reactions with the same template and following primers –

Cys (5′-gccctcagggtacatgtagatGCAtggatttcccataggagg-3′)
Glu (5′-gccctcagggtacatgtagatTTCtggatttcccataggagg-3′)
Ile (5′-gccctcagggtacatgtagatAATtggatttcccataggagg-3′)
Met (5′-gccctcagggtacatgtagatCATtggatttcccataggagg-3′)
Asn (5′-gccctcagggtacatgtagatGTTtggatttcccataggagg-3′)
Gln (5′-gccctcagggtacatgtagatCTGtggatttcccataggagg-3′)
Val (5′-gccctcagggtacatgtagatCACtggatttcccataggagg-3′)
Tyr (5′-gccctcagggtacatgtagatATAtggatttcccataggagg-3′).

Due to expression problems with Leu and Ser variants their codons were changed to TCC for Ser and CTG for Leu. W373K was obtained previously [7]. All variants were finally confirmed by sequencing (SEQme, Czech Republic).

### Screening

Positive clones were screened for activity directly on Petri dishes by a procedure developed to identify clones with β-glucosidase activity using the chromogenic substrate 5-bromo-4-chloro-3-indolyl-O-β-D-glucopyranoside (XGlc, supplied by Biosynth AG, Staad, Switzerland), which yields a blue reaction product [2]. A 40 mg L^−1^ dose of XGlc was found to be sufficient and cultivation at 37°C for 12 h, followed by 12 h at 16°C, gave the best results.

### Characterization of β-glucosidase Zm-p60.1 variants

The protein variants were expressed and purified as previously reported [8] and described in Supplemental Results (File S4). Protein concentration was determined by the Bradford Protein Assay with BSA as the calibration standard (Bio-Rad Laboratories, Hercules, CA, USA). The average purified enzyme yield was 7.7 mg/L of bacterial culture. All purified enzymes were tested for their activity towards the natural substrate *trans*-zeatin-O-β-D-glucoside (*t*ZOG, OlChemIm, Olomouc, Czech Republic) at a concentration of 1 mM. An enzyme reaction with β-glucosidase was performed in triplicate at 30°C, in 50 mM citrate-phosphate buffer (C-P buffer, pH = 5.5). Amounts of glucose released in 25 µl portions of the enzymatic reaction mixtures were measured using an Amplex Red Glucose/Glucose Oxidase Assay Kit (Life Science, St. Petersburg, FL, USA) and a Tecan Infinite 200 PRO fluorescent reader (Tecan Systems Inc, San Jose, CA, USA). Hydrolysis rates for each variant were then calculated using a glucose standard calibration curve. The procedures applied for determining enzyme activity, using 4-nitrophenyl-O-β-D-glucoside (*p*NPG, Sigma–Aldrich, St. Louis, MO, USA), have been previously described [7] and the results are shown in File S5.

## Supporting Information

File S1 The pH optimum analysis of the most and least active variants (W373H and W373K).
(DOC)

File S2 Multiple sequence alignment (File S2 where position 1833 corresponds to the position of W373 in Zm-p60.1 or in File S3 position 18 in detailed view).
(PDF)

File S3 Multiple sequence alignment (File S2, where position 1833 corresponds to the position of W373 in Zm-p60.1 or in File S3 position 18 in detailed view).
(PDF)

File S4 The protein variants expression and purification details.
(DOC)

File S5 Determination of enzymes activity, using 4-nitrophenyl-O-β-D-glucoside.
(XLS)

File S6 The simulation used to calculate theoretical average numbers of clones that needed to be screened for a new hit based on Monte Carlo method.
(ZIP)
