# Supplementary material for: Combining Rational and Random Strategies in β-Glucosidase Zm-p60.1 Protein Library Construction
Source: PLoS One. 2014 Sep 26;9(9):e108292. doi: 10.1371/journal.pone.0108292 (PMC4178128; doi:10.1371/journal.pone.0108292)
Supplement: File S1 — The pH optimum analysis of the most and least active variants (W373H and W373K). (DOC) [file pone.0108292.s001.doc]

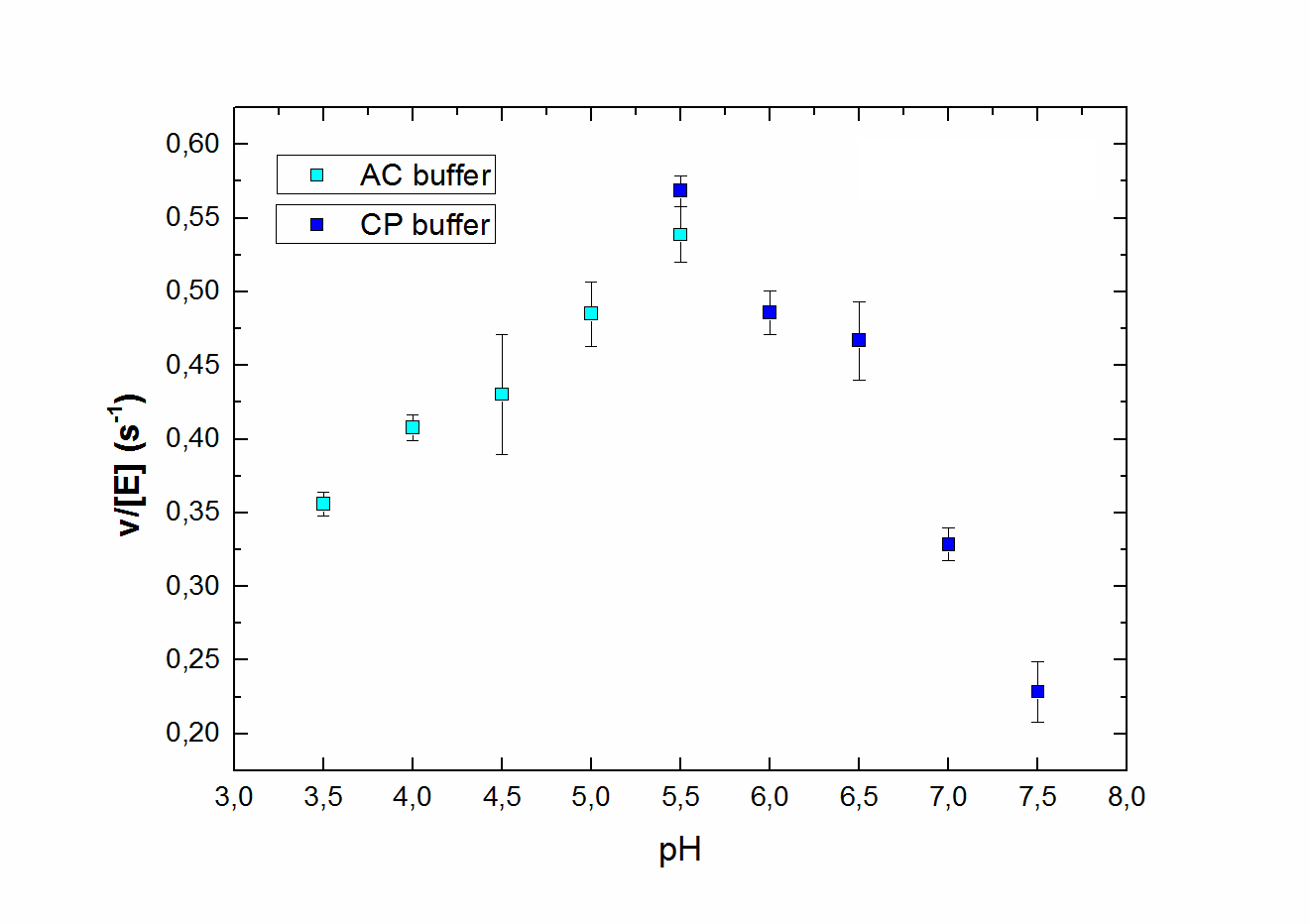

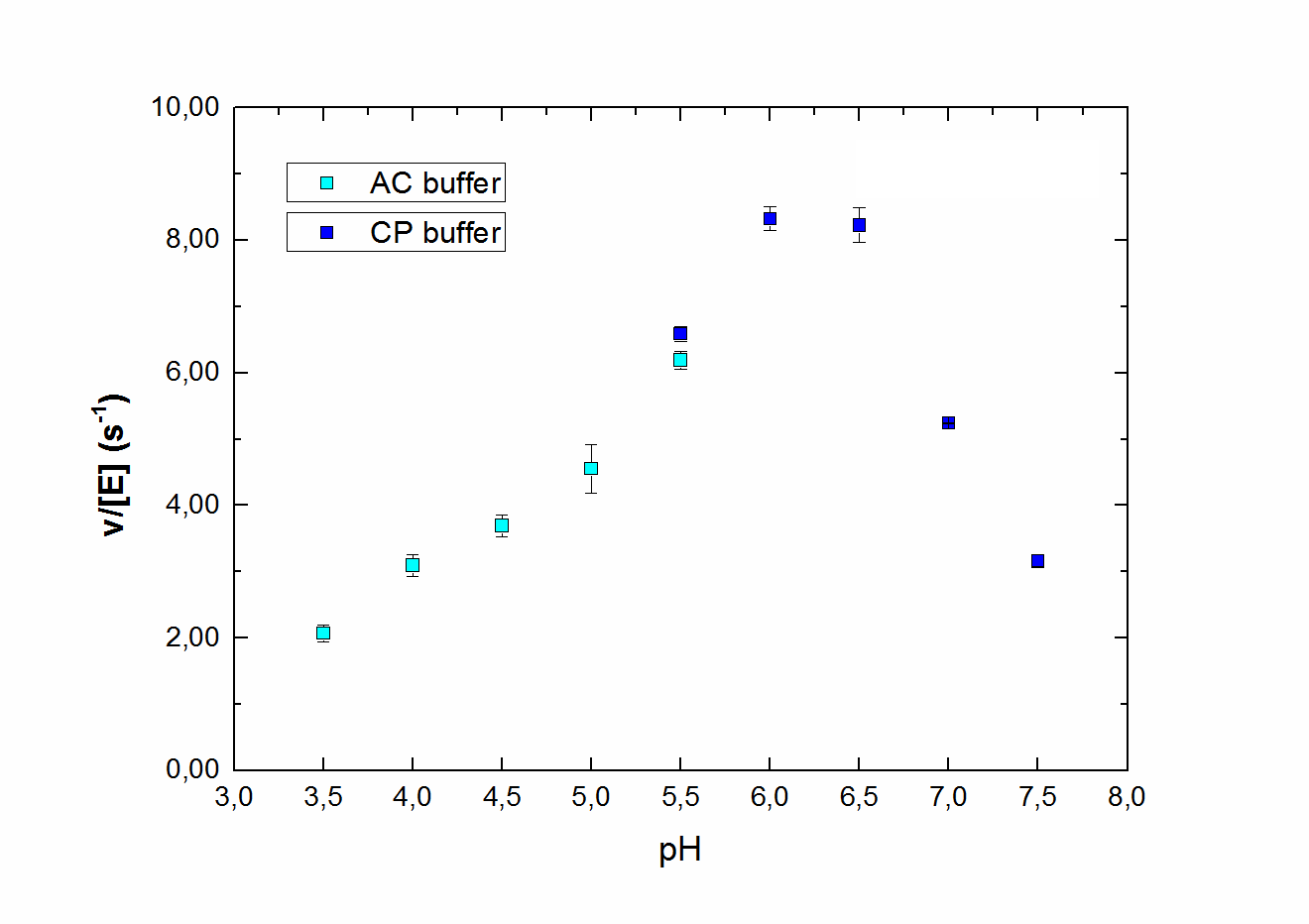


**W373K**

**W373H**

Fig. 01 - pH profiles of the most (W373H) and least (W373K) active mutants in the W373X library.

100 mM acetate buffer (pH = 3.5–5.5)

100 mM citrate-phosphate (pH = 5.5–7.5)

Rate constants were measured with 40 mM *p*NPG at 30°C
