# Supplementary material for: Combining Rational and Random Strategies in β-Glucosidase Zm-p60.1 Protein Library Construction
Source: PLoS One. 2014 Sep 26;9(9):e108292. doi: 10.1371/journal.pone.0108292 (PMC4178128; doi:10.1371/journal.pone.0108292)
Supplement: File S2 — Multiple sequence alignment (File S2 where position 1833 corresponds to the position of W373 in Zm-p60.1 or in File S3 position 18 in detailed view). (PDF) [file pone.0108292.s002.pdf]

```
                            10        20        30        40        50        60        70        80        90       100
                   ....|....|....|....|....|....|....|....|....|....|....|....|....|....|....|....|....|....|....|....|
AC155376.2_FGT005  MGST------------------------------------------------------------------------------------------------
AC211140.2_FGT007  MQPR------------------------------------------------------------------------------------------------
AC217401.3_FGT001  MIAVRW----------------------------------------------------------------------------------------------
AC217687.3_FGT004  MDARWA----------------------------------------------------------------------------------------------
AtBglu1            MED-------------------------------------------------------------------------------------------------
AtBglu10           MKL-------------------------------------------------------------------------------------------------
AtBglu11           MKL-------------------------------------------------------------------------------------------------
AtBglu12           MRTIYL----------------------------------------------------------------------------------------------
AtBglu13           MRTKYF----------------------------------------------------------------------------------------------
AtBglu14           MTSKYF----------------------------------------------------------------------------------------------
AtBglu15           MRGNYL----------------------------------------------------------------------------------------------
AtBglu16           MRGKFL----------------------------------------------------------------------------------------------
AtBglu17           MAIKSI----------------------------------------------------------------------------------------------
AtBglu18           MVRFEK--------------V------------H------------------------------------------------------------------
AtBglu19           MKIP------------------------------------------------------------------------------------------------
AtBglu2            MLH-------------------------------------------------------------------------------------------------
AtBglu20           MGRFHK--------------F------------P------------------------------------------------------------------
AtBglu21           MALQKF--------------P------------L------------------------------------------------------------------
AtBglu22           MALQKF--------------P------------L------------------------------------------------------------------
AtBglu23           MVLQKL--------------P------------L------------------------------------------------------------------
AtBglu24           MVLQKL--------------P------------L------------------------------------------------------------------
AtBglu25           MALKAI--------------L------------F------------------------------------------------------------------
AtBglu26           MAHLQR--------------T-------------------------------------------------------------------------------
AtBglu27           MYS-------------------------------------------------------------------------------------------------
AtBglu28           MKMHF-----------------------------------------------------------------------------------------------
AtBglu29           MNVQI-----------------------------------------------------------------------------------------------
AtBglu3            MEL-------------------------------------------------------------------------------------------------
AtBglu30           MAKGSW--------------F-------------------------------------------------------------------------------
AtBglu31           MAIKL-----------------------------------------------------------------------------------------------
AtBglu32           MAIKL-----------------------------------------------------------------------------------------------
AtBglu33           MATATL--------------T------------L------------------------------------------------------------------
AtBglu34           MAIPKA--------------H------------Y------------------------------------------------------------------
AtBglu35           MAIPKA--------------H------------Y------------------------------------------------------------------
AtBglu36           MQSRMQ--------------G------------Q------------------------------------------------------------------
AtBglu37           MQHNTY--------------I------------Y------------------------------------------------------------------
AtBglu38           MKLLML----------------------------------------------------------------------------------------------
AtBglu39           MKFRAL----------------------------------------------------------------------------------------------
AtBglu4            MEQ-------------------------------------------------------------------------------------------------
AtBglu40           MAHRRL----------------------------------------------------------------------------------------------
AtBglu41           MESL------------------------------------------------------------------------------------------------
AtBglu42           MAQKLN----------------------------------------------------------------------------------------------
AtBglu43           M---------------------------------------------------------------------------------------------------
AtBglu44           MRHLSS--------------P-------------------------------------------------------------------------------
AtBglu45           MKNLTS----------------------------------------------------------------------------------------------
AtBglu46           MKTFAN----------------------------------------------------------------------------------------------
AtBglu47           MKKSIV--------------Y------------EIM------------------------------------------------------------ETKS
```

```
AtBglu5         MFD-------------------------------------------------------------------------------------------------
AtBglu7         MKP-------------------------------------------------------------------------------------------------
AtBglu8         MKH-------------------------------------------------------------------------------------------------
AtBglu9         MKH-------------------------------------------------------------------------------------------------
BD1G10890       MARSTT----------------------------------------------------------------------------------------------
BD1G10920       MARSTT----------------------------------------------------------------------------------------------
BD1G10930       MVAAAR----------------------------------------------------------------------------------------------
BD1G10940       MAAAAP--------------S------------AAR------------------------------------------------------------G---
BD1G19270       MKSTAL--------------V-------------------------------------------------------------------------------
BD1G33040       MGVL------------------------------------------------------------------------------------------------
BD1G42690       MSFVL-----------------------------------------------------------------------------------------------
BD1G70170       MGIKRP--------------G------------R-------------C-----------------------------------------N----CAT---
BD2G09190       MRPLR-----------------------------------------------------------------------------------------------
BD2G09200       MDGYTM----------------------------------------------------------------------------------------------
BD2G27770       MRAIPC--------------A------------Y------------------------------------------------------------------
BD2G37450       MANPDD--------------G-------------------------------------------------------------------------------
BD2G57640       MGSTGR----------------------------------------------------------------------------------------------
BD2G59650       MAA-------------------------------------------------------------------------------------------------
BD2G59660       MAI-------------------------------------------------------------------------------------------------
BD3G00650       MRMAYK--------------W------------G--------------------------------------------------------------C---
BD3G40000       MSVSAG--------------M-------------------------------------------------------------------------------
BD3G40010       MEHSR-----------------------------------------------------------------------------------------------
BD3G45610       MGV-------------------------------------------------------------------------------------------------
BD3G45630       MGV-------------------------------------------------------------------------------------------------
BD3G45640       MGG-------------------------------------------------------------------------------------------------
BD3G45650       MGL-------------------------------------------------------------------------------------------------
BD4G08040       MEPHPH--------------A-------------------------------------------------------------------------------
BD4G09920       MPWPAF--------------L-------------------------------------------------------------------------------
BD4G34930       MAQTAA----------------------------------------------------------------------------------------------
BD4G34940       MALLRV----------------------------------------------------------------------------------------------
BD4G34950       MAMLLY----------------------------------------------------------------------------------------------
BD5G13260       MAAGAL--------------V------------A------------------------------------------------------------------
BD5G13270       MNTRVW----------------------------------------------------------------------------------------------
BD5G15530       MASEAA--------------A-------------------------------------------------------------------------------
BD5G15540       MAAATG----------------------------------------------------------------------------------------------
GRMZM2G012236   MARLFL----------------------------------------------------------------------------------------------
GRMZM2G015804   MHCA------------------------------------------------------------------------------------------------
GRMZM2G031660   MAGRAR--------------A-------------------------------------------------------------------------------
GRMZM2G055699   MGGPGR--------------P------------W------------------------------------------------------------------
GRMZM2G069024   MAA-------------------------------------------------------------------------------------------------
GRMZM2G108133   MMGRKA----------------------------------------------------------------------------------------------
GRMZM2G110948   MPRTVT--------------P-------------------------TPVPCASPYLWPPPQNWPRASQGSLAHFFPAECAVVMANASRGRV--GG-G---
GRMZM2G112704   MATAAP--------------L-------------------------------------------------------------------------------
GRMZM2G128197   MGASGP--------------A------------APP---------------------------------------------------------ATHR---
GRMZM2G148176   MQSSVK--------------L-------------------------------------------------------------------------------
GRMZM2G163544   MAVL------------------------------------------------------------------------------------------------
GRMZM2G177661   MEPT------------------------------------------------------------------------------------------------
GRMZM2G376416   MIC-------------------------------------------------------------------------------------------------
```

```
GRMZM2G457040    MATARA--------------V-------------------------------------------------------------------------------
Os10bglu34       MGNGGR----------------------------------------------------------------------------------------------
Os11bglu36       MPLPAF--------------V-------------------------------------------------------------------------------
Os12bglu38       MNMPL-----------------------------------------------------------------------------------------------
Os1bglu1         MGHRL-----------------------------------------------------------------------------------------------
Os1bglu2         MGA-------------------------------------------------------------------------------------------------
Os1bglu3         MAA-------------------------------------------------------------------------------------------------
Os1bglu4         MGST------------------------------------------------------------------------------------------------
Os1bglu5         MKLEIK--------------P----PQPHVLTRM------------------------------------------------------------------
Os3bglu6         MGRIKS--------------S------------S-------------G-----------------------------------------R----CST---
Os3bglu7         MAARRA----------------------------------------------------------------------------------------------
Os3bglu8         MGCAPA--------------A------------HYL------------------------------------------------------P--G--G---
Os4bglu10        MAVAGA--------------M------------V------------------------------------------------------------------
Os4bglu11        MAVAGA--------------M------------V------------------------------------------------------------------
Os4bglu12        MAAAGA----------------------------------------------------------------------------------------------
Os4bglu13        MSLFQPLGHTHILPMAAAGEV------------V------------------------------------------------------------------
Os4bglu14        MAAAWL----------------------------------------------------------------------------------------------
Os4bglu16        MAVAAA--------------T-------------------------------------------------------------------------------
Os4bglu18        MAGGSK--------------T-------------------------------------------------------------------------------
Os4bglu27        MRW-------------------------------------------------------------------------------------------------
Os4bglu28        MDRRL-----------------------------------------------------------------------------------------------
Os4bglu9         MAVAGA--------------V------------A------------------------------------------------------------------
Os5bglu19        MCGCAS--------------G---EDAMETRRPL------------------------------------------------------------------
Os5bglu21        MERPLH----------------------------------------------------------------------------------------------
Os5bglu22        MAVSSS--------------T------------S------------------------------------------------------------------
Os5bglu23        MAACTS--------------S------------L------------------------------------------------------------------
Os5gblu20        MERRPL----------------------------------------------------------------------------------------------
Os6bglu24        MELLW-----------------------------------------------------------------------------------------------
Os6bglu25        MSLL------------------------------------------------------------------------------------------------
Os7bglu26        MPPEDK--------------A------------LFVSRLPAPSVCNA-------------------------------------------------D---
Os9bglu29        MAWLGI--------------G-------------------------------------------------------------------------------
Os9bglu30        MGIRM-----------------------------------------------------------------------------------------------
Os9bglu31        MTPARV----------------------------------------------------------------------------------------------
Os9bglu32        MAAGAR----------------------------------------------------------------------------------------------
Os9bglu33        MATGEL----------------------------------------------------------------------------------------------
SB01G010825      MGAAAA--------------G------------PAA------------------------------------------------------APPATTH---
SB01G010830      MVAARW----------------------------------------------------------------------------------------------
SB01G013360      ----------------------------------------------------------------------------------------------------
SB01G043030      MPPV------------------------------------------------------------------------------------------------
SB02G028400      MGVGNG----------------------------------------------------------------------------------------------
SB02G029620      MAAKTT----------------------------------------------------------------------------------------------
SB02G041550      MQSTVK--------------L-------------------------------------------------------------------------------
SB03G037780      MAA-------------------------------------------------------------------------------------------------
SB03G042690      MERTNC--------------H------------T-------------G-----------------------------------------HSALGSAS---
SB06G019830      MF--------------------------------------------------------------------------------------------------
SB06G019840      MAAVRV-------------AA------------A------------------------------------------------------------------
SB06G019850      MLARLL----------------------------------------------------------------------------------------------
SB06G019860      MLARLL----------------------------------------------------------------------------------------------
```

```
SB06G019880    ----------------------------------------------------------------------------------------------------
SB06G022410    MARLFF----------------------------------------------------------------------------------------------
SB06G022420    MRAHQL--------------G------------SSA------------------------------------------------------------T---
SB06G022450    MAGVII--------------V-------------------------------------------------------------------------------
SB06G022460    MAGVII--------------V-------------------------------------------------------------------------------
SB06G022490    MATARA--------------I-------------------------------------------------------------------------------
SB06G022500    MAGRAR--------------A-------------------------------------------------------------------------------
SB06G022510    MAAAGS--------------W------------PRA------------------------------------------------------------SA--
SB08G007586    MALLL-----------------------------------------------------------------------------------------------
SB08G007650    MALLL-----------------------------------------------------------------------------------------------
SB09G018160    MGGRLL----------------------------------------------------------------------------------------------
SB10G012220    MSRLA-----------------------------------------------------------------------------------------------
SB10G022300    MVELTP--------------RGNGC--------C------------------------------------------------------------------
SB10G027600    MGVL------------------------------------------------------------------------------------------------
SB10G028060    MASSAA--------------A------------PWS----------------------------------------------------------------
SbDhr1         MALLL-----------------------------------------------------------------------------------------------
SbDhr2         MAPLL-----------------------------------------------------------------------------------------------
Zm-p60.1       MAPLL-----------------------------------------------------------------------------------------------
ZmGLU2         MAPLL-----------------------------------------------------------------------------------------------
ZmGLU3         MAPLV-----------------------------------------------------------------------------------------------
ZmGLU4         MAMNH-----------------------------------------------------------------------------------------------
ZmGLU5         MAPLV-----------------------------------------------------------------------------------------------
ZmGLU6         MDRFG-----------------------------------------------------------------------------------------------
ZmGLU7         MAPLL-----------------------------------------------------------------------------------------------
ZmGLU8         MAPLL-----------------------------------------------------------------------------------------------
```

```
                           110       120       130       140       150       160       170       180       190       200
                    ....|....|....|....|....|....|....|....|....|....|....|....|....|....|....|....|....|....|....|....|
AC155376.2_FGT005   ----------------------------------------------------------------------------------------------------
AC211140.2_FGT007   -------P---A-L-LLLVLVLG-----------------------------------------------------------------------------
AC217401.3_FGT001   ----A------A---LLVALV-V-----------------------------------------------------------------------------
AC217687.3_FGT004   ---------------VLLALLVA-----------------------------------------------------------------------------
AtBglu1             ------VL--TL---ITMIVLLL-----------------------------------------------------------------------------
AtBglu10            ------YS--LL----SVFLVIL-----------------------------------------------------------------------------
AtBglu11            ------LS--NS---LMFLPLLA-----------------------------------------------------------------------------
AtBglu12            -----------S---LLVFIIVL-----------------------------------------------------------------------------
AtBglu13            -----------S---LLVFIIVL-----------------------------------------------------------------------------
AtBglu14            -----------S---VLVFII-L-----------------------------------------------------------------------------
AtBglu15            -----------S---LLVVLIVL-----------------------------------------------------------------------------
AtBglu16            -----------S---LLLLI-TL-----------------------------------------------------------------------------
AtBglu17            -----------F---IIIIISII-----------------------------------------------------------------------------
AtBglu18            ----L--V---L---GLALVLTL-----------------------------------------------------------------------------
AtBglu19            ----L------L---GLLLLISL-----------------------------------------------------------------------------
AtBglu2             ------CI--TT---I---FLSI-----------------------------------------------------------------------------
AtBglu20            ----L------L---GLVLFLGL-----------------------------------------------------------------------------
AtBglu21            ----M--G--------LLLLLTI-----------------------------------------------------------------------------
AtBglu22            ----L--G--------LLFLITI-----------------------------------------------------------------------------
AtBglu23            ----I--G--------LLLLLTI-----------------------------------------------------------------------------
AtBglu24            ----M--S---I---GLLWLLII-----------------------------------------------------------------------------
AtBglu25            ----L--G--------LF-LVVI-----------------------------------------------------------------------------
AtBglu26            ----------------------------------------------------------------------------------------------------
AtBglu27            ----------------------------------------------------------------------------------------------------
AtBglu28            -----------F---ILLVITSW-----------------------------------------------------------------------------
AtBglu29            -----------F---ILLLIISW-----------------------------------------------------------------------------
AtBglu3             ------TL--SL---LTI-FLLF-----------------------------------------------------------------------------
AtBglu30            -----------F---IILFIISM-----------------------------------------------------------------------------
AtBglu31            -----------I---ALVITLCV-----------------------------------------------------------------------------
AtBglu32            -----------I---ALVITICV-----------------------------------------------------------------------------
AtBglu33            ----------------FLGLLAL-----------------------------------------------------------------------------
AtBglu34            ---SL--A-------VLVLLFVV-----------------------------------------------------------------------------
AtBglu35            ---SL--A-------VLVLLFVV-----------------------------------------------------------------------------
AtBglu36            -------R-------TLQL---R-----------------------------------------------------------------------------
AtBglu37            ---IL--TMKLLGF-ALAILLVV-----------------------------------------------------------------------------
AtBglu38            ---------------AFVFLLAL-----------------------------------------------------------------------------
AtBglu39            ---------------GLVLLLAV-----------------------------------------------------------------------------
AtBglu4             ------IL--AL---FAI-FL-A-----------------------------------------------------------------------------
AtBglu40            ----I-MT---M-T-KMMMMVTM-----------------------------------------------------------------------------
AtBglu41            -------M---R-L-VLVLFPFF-----------------------------------------------------------------------------
AtBglu42            -------------L-L------------------------------------------------------------------------------------
AtBglu43            ---------------FLFLLLLS-----------------------------------------------------------------------------
AtBglu44            ------PW---P---LLLLLLLS-----------------------------------------------------------------------------
AtBglu45            -------F---V-I--VILLQSL-----------------------------------------------------------------------------
AtBglu46            -------F---A-I--LFLLQSL-----------------------------------------------------------------------------
AtBglu47            SMYLS-QF---R-L-WLCFIITT-----------------------------------------------------------------------------
```

```
AtBglu5          ----------------------------------------------------------------------------------------------------
AtBglu7          ------FS--QF----FVFVVTV-----------------------------------------------------------------------------
AtBglu8          ------FN--LL----SIILVIV-----------------------------------------------------------------------------
AtBglu9          ------FS--LL----FIFLVIL-----------------------------------------------------------------------------
BD1G10890        ----A------R---WLLLLALV-----------------------------------------------------------------------------
BD1G10920        ----T--A---R---WLLLLALV-----------------------------------------------------------------------------
BD1G10930        ----S-TA---R---WLLLLALV-----------------------------------------------------------------------------
BD1G10940        ---LP-RL---L---LLLLVAVL-----------------------------------------------------------------------------
BD1G19270        ----------------LLTLAAH-----------------------------------------------------------------------------
BD1G33040        -------T-----L-VHIL---------------------------------------------------------------------------------
BD1G42690        ---------------MILL-SLT-----------------------------------------------------------------------------
BD1G70170        ---A--AE---L-L-ALLLIILG-----------------------------------------------------------------------------
BD2G09190        -------------R-VVALLLAA-----------------------------------------------------------------------------
BD2G09200        ----M-MP--LR-S-VLALLLAA-----------------------------------------------------------------------------
BD2G27770        -C--R-RK--SP-L-LVSLLLLV-----------------------------------------------------------------------------
BD2G37450        ----------GA-A-AG-----------------------------------------------------------------------------------
BD2G57640        ----------------------------------------------------------------------------------------------------
BD2G59650        ------IV--FV----FVFSLLL-----------------------------------------------------------------------------
BD2G59660        ------LS--SL----LLLLLLL-----------------------------------------------------------------------------
BD3G00650        ---ME-AT---T-M-VLVVWLAA-----------------------------------------------------------------------------
BD3G40000        ---DR--V---L---LLPALLLA-----------------------------------------------------------------------------
BD3G40010        -------R---A---LLCTLLLA-----------------------------------------------------------------------------
BD3G45610        ------TA---F----FYILLSL-----------------------------------------------------------------------------
BD3G45630        ------TA---F----FYILLFL-----------------------------------------------------------------------------
BD3G45640        ------VT--AF----FYILLSL-----------------------------------------------------------------------------
BD3G45650        ------AA---F----FFILLSL-----------------------------------------------------------------------------
BD4G08040        ------AA---R---ALVFLSLA-----------------------------------------------------------------------------
BD4G09920        ----------AA-A-VKLAVLAA-----------------------------------------------------------------------------
BD4G34930        -------------A-FVFLLLAA-----------------------------------------------------------------------------
BD4G34940        ----L--T--PP----LFLSFFF-----------------------------------------------------------------------------
BD4G34950        ----F-----------LLL-LLR-----------------------------------------------------------------------------
BD5G13260        ---ML--A---R---LVLP-FLL-----------------------------------------------------------------------------
BD5G13270        -----------------V----------------------------------------------------------------------------------
BD5G15530        ----R----------LLLLLSAH-----------------------------------------------------------------------------
BD5G15540        ---------------ICAVLLAI-----------------------------------------------------------------------------
GRMZM2G012236    ----------------LLLFS-------------------------------------------------------------------------------
GRMZM2G015804    -------G---R----------------------------------------------------------------------------------------
GRMZM2G031660    -RRFV-LA---G-A-LMIAWLLC-----------------------------------------------------------------------------
GRMZM2G055699    -H--R-HV--FL-V-VSLQLLLV-----------------------------------------------------------------------------
GRMZM2G069024    ------TF--AF----IPLLLLV-----------------------------------------------------------------------------
GRMZM2G108133    ----P-LG--CA-T-TLLLLLAA-----------------------------------------------------------------------------
GRMZM2G110948    ---GR-SS---V---LALLLLLS-----------------------------------------------------------------------------
GRMZM2G112704    ------LL---F-H-G--LLLLL-----------------------------------------------------------------------------
GRMZM2G128197    ---RR-CI---Q---LLLLLALA-----------------------------------------------------------------------------
GRMZM2G148176    ---------------AVLALLLA-----------------------------------------------------------------------------
GRMZM2G163544    -------T-----L-VNIL---------------------------------------------------------------------------------
GRMZM2G177661    -------R---L-L--LLLLVLG-----------------------------------------------------------------------------
GRMZM2G376416    ----------------------------------------------------------------------------------------------------
```

```
GRMZM2G457040   ----A-AA---A-A-ALAAVVLV-----------------------------------------------------------------------------
Os10bglu34      ----C-MV---E-V-VILLVLMA-----------------------------------------------------------------------------
Os11bglu36      ----------AA-A-ARLAVLVA-----------------------------------------------------------------------------
Os12bglu38      -------L-------LLIAIVVV-----------------------------------------------------------------------------
Os1bglu1        -------V---V---VLLLALLV-----------------------------------------------------------------------------
Os1bglu2        ------AA--AA-G-FFFVLLFL-----------------------------------------------------------------------------
Os1bglu3        ------AA--A----FFCALLFI-----------------------------------------------------------------------------
Os1bglu4        ----------------------------------------------------------------------------------------------------
Os1bglu5        -A--A-AI--AV---VYLSLLLL-----------------------------------------------------------------------------
Os3bglu6        ---AR-LE---A-V-AVLVVVFG-----------------------------------------------------------------------------
Os3bglu7        ----N--C---A---LVLVLALA-----------------------------------------------------------------------------
Os3bglu8        ---GW-RR---L---LVVVVALV-----------------------------------------------------------------------------
Os4bglu10       ---MS--G---G---VLL-L-LL-----------------------------------------------------------------------------
Os4bglu11       ---MS--G---A---LLL-LHLL-----------------------------------------------------------------------------
Os4bglu12       ---MP--G---G---LLLTFLLL-----------------------------------------------------------------------------
Os4bglu13       ---ML--G---G---ILLP-LLL-----------------------------------------------------------------------------
Os4bglu14       ---------------VVLLTVHR-----------------------------------------------------------------------------
Os4bglu16       ----R-IA-------VVVVVLAL-----------------------------------------------------------------------------
Os4bglu18       -R-I--HA-----S-LVSTLLLL-----------------------------------------------------------------------------
Os4bglu27       ---------------LLLALFLV-----------------------------------------------------------------------------
Os4bglu28       ---------------LLSALLFI-----------------------------------------------------------------------------
Os4bglu9        ---MSGGI---L---LLLLLLLL-----------------------------------------------------------------------------
Os5bglu19       -H--P-LL--LF-FSPWLLLLLL-----------------------------------------------------------------------------
Os5bglu21       ----L--L--LV-F--LSS-PWL-----------------------------------------------------------------------------
Os5bglu22       -T--C-SS--FS-L-LLLLLLLA-----------------------------------------------------------------------------
Os5bglu23       ------VS--LL-L-LLLLLLLL-----------------------------------------------------------------------------
Os5gblu20       -H--L-HL--LL-F--FSAWLLL-----------------------------------------------------------------------------
Os6bglu24       ---------------LLLL-LLM-----------------------------------------------------------------------------
Os6bglu25       -------T-----L-VHIL---------------------------------------------------------------------------------
Os7bglu26       ---MR-KF---I---AALRLALA-----------------------------------------------------------------------------
Os9bglu29       ---MG--R---Q---IVPVLVFV-----------------------------------------------------------------------------
Os9bglu30       ----G--R---R---LLFTLFLG-----------------------------------------------------------------------------
Os9bglu31       ----V------F-I-CCVVLLAA-----------------------------------------------------------------------------
Os9bglu32       ----A-LV--PS-P-FIVVVFLL-----------------------------------------------------------------------------
Os9bglu33       ----A-LV--SS-L-FIVVVFLL-----------------------------------------------------------------------------
SB01G010825     ---CR-CI---Q----LLLLALA-----------------------------------------------------------------------------
SB01G010830     ----A--G---A---LLLALLVV-----------------------------------------------------------------------------
SB01G013360     ----------------------------------------------------------------------------------------------------
SB01G043030     -------V---V-L-LLLLVVLG-----------------------------------------------------------------------------
SB02G028400     ---MA--K---L---LLPALLLA-----------------------------------------------------------------------------
SB02G029620     ----H-S---YD-A-ALLFLLAG-----------------------------------------------------------------------------
SB02G041550     ---------------AVLALLLA-----------------------------------------------------------------------------
SB03G037780     ------AF--TV----ISLLLSV-----------------------------------------------------------------------------
SB03G042690     ---PA-RP-----M-LSRLA--RVTPPSIGRQGRHAVVAAHVFSPCALAGRGKRRVCRLPLRANANVRRQRHLLIDTPHRNPKAHASEFTNSSSSPRRDR
SB06G019830     ----------------------------------------------------------------------------------------------------
SB06G019840     ---ML--G---R---LLLLPLLL-----------------------------------------------------------------------------
SB06G019850     -----------P---LLL-LLLL-----------------------------------------------------------------------------
SB06G019860     -----------P---LLL-LLAA-----------------------------------------------------------------------------
```

```
SB06G019880    ----------------------------------------------------------------------------------------------------
SB06G022410    ---------------FVLLYP-------------------------------------------------------------------------------
SB06G022420    ----A----------APVLSV-------------------------------------------------------------------------------
SB06G022450    ---------------LVFFLAHQ-----------------------------------------------------------------------------
SB06G022460    ---------------LAFFLAHQ-----------------------------------------------------------------------------
SB06G022490    ----A-AA---------AAVLVA-----------------------------------------------------------------------------
SB06G022500    -RRLM-LA---G-A-LLLL-LGL-----------------------------------------------------------------------------
SB06G022510    -RRLL-LA---G-A-YYSL-LWL-----------------------------------------------------------------------------
SB08G007586    -----------------ASAMNH-----------------------------------------------------------------------------
SB08G007650    -----------------ASAMNH-----------------------------------------------------------------------------
SB09G018160    ----L--F--LP----W-LLQLL-----------------------------------------------------------------------------
SB10G012220    ---------------LLLLFALP-----------------------------------------------------------------------------
SB10G022300    -P--R-VL--SV-S-LLLLLLAA-----------------------------------------------------------------------------
SB10G027600    -------T-----L-VHIL---------------------------------------------------------------------------------
SB10G028060    ---LT-LF---F-L-PLCFLLVV-----------------------------------------------------------------------------
SbDhr1         -----------------ASAMNH-----------------------------------------------------------------------------
SbDhr2         -----------------LHASAI-----------------------------------------------------------------------------
Zm-p60.1       -----------------AAAMNH-----------------------------------------------------------------------------
ZmGLU2         -----------------AAAMNH-----------------------------------------------------------------------------
ZmGLU3         -----------------AAATNH-----------------------------------------------------------------------------
ZmGLU4         -----------------AGHPG------------------------------------------------------------------------------
ZmGLU5         -----------------ATATMN-----------------------------------------------------------------------------
ZmGLU6         -----------------NYKNYQ-----------------------------------------------------------------------------
ZmGLU7         -----------------LASRTA-----------------------------------------------------------------------------
ZmGLU8         -----------------LASRTA-----------------------------------------------------------------------------
```

```
                           210       220       230       240       250       260       270       280       290       300
                   ....|....|....|....|....|....|....|....|....|....|....|....|....|....|....|....|....|....|....|....|
AC155376.2_FGT005  ----------------------------------------------------------------------------------------------------
AC211140.2_FGT007  --------------------VSL-R----GCI--A-----------------------------------------------------------------
AC217401.3_FGT001  --------------------ACG-S----AAAGAAA--------G-------------------------------------------------------
AC217687.3_FGT004  --------------------SGG-V----RVCAAAG--------A-------------------------------------------------------
AtBglu1            --------------------LAF-H----G----------------------------------------------------------------------
AtBglu10           --------------------LA------------------------------------------------------------------------------
AtBglu11           --------------------LA-------L----------------------------------------------------------------------
AtBglu12           --------------------ALN-E----VM-AK------------------------------------------------------------------
AtBglu13           --------------------ASN-E----VI-AK------------------------------------------------------------------
AtBglu14           --------------------ASN-E----VV-AK------------------------------------------------------------------
AtBglu15           --------------------ASN-D----VL-AN------------------------------------------------------------------
AtBglu16           --------------------ACI-G----VS-AK------------------------------------------------------------------
AtBglu17           --------------------TSI-S----ELYAL----------------------------------------------------------D-------
AtBglu18           --------------------VGA-P----TKAQGPV--------C-------------------------------------------------------
AtBglu19           --------------------VGS-P----TRAEEG---------P-------------------------------------------------------
AtBglu2            --------------------SRM-T---------------------------------------------------------------------------
AtBglu20           --------------------TGS-L----IAANEY---------A-------------------------------------------------------
AtBglu21           --------------------LVS-V----TTAVD-D--------P-------------------------------------------------------
AtBglu22           --------------------VVS-S----TIAVD-D--------P-------------------------------------------------------
AtBglu23           --------------------VAS-P----A-NAD-G--------P-------------------------------------------------------
AtBglu24           --------------------VGP-L----VNADGPV--------C-------------------------------------------------------
AtBglu25           --------------------VSP-I----T-VYG-G--------A-------------------------------------------------------
AtBglu26           ----------------------------------------------------------------------------------------------------
AtBglu27           ----------------------------------------------------------------------------------------------------
AtBglu28           --------------------LSE-K----I-T------------S-------------------------------------------------------
AtBglu29           --------------------LTP-K----I-T------------S-------------------------------------------------------
AtBglu3            --------------------FAL-S---------------------------------------------------------------------------
AtBglu30           --------------------LEN-M---------------------------------------------------------------------------
AtBglu31           --------------------ASW-D----V-AQG-R--------S-------------------------------------------------------
AtBglu32           --------------------ASW-D----S-AQG-R--------S-------------------------------------------------------
AtBglu33           --------------------TST-I----LSFNADA--------R----P----------------------------------------QPSDEDLGTI
AtBglu34           --------------------VSS-S----QKVCNPE--------C----K----------------------------------------AKE-------
AtBglu35           --------------------VSS-S----QKVCNPE--------C----K----------------------------------------AKE-------
AtBglu36           --------------------QNS-C---------------------------------------------------------------------------
AtBglu37           --------------------ATC-K----PEE-EIT--------C----E----------------------------------------ENV-------
AtBglu38           --------------------ATC-K----GDE--FV--------C----E----------------------------------------ENE-------
AtBglu39           --------------------ETC-K----AEE--IT--------C----E----------------------------------------ETK-------
AtBglu4            --------------------FA-------F----------------------------------------------------------------------
AtBglu40           --------------------MMM-M---------------------------------------------------------------------------
AtBglu41           --------------------VVF-F-------V-P-----------------------------------------------------------------
AtBglu42           ----------------------------------------------------------------------------------------------------
AtBglu43           --------------------ASR-S----GEESP-S--------------------------------------------------------G-------
AtBglu44           --------------------SFT-S----GESSL-S--------------------------------------------------------A-------
AtBglu45           --------------------LFH-V----YGRHQ------------------------------------------------------------------
AtBglu46           --------------------LFP-L----Y---S------------------------------------------------------------------
AtBglu47           --------------------LVS-L----SSSTRWY--------D-----------------------------------------------D-------
```

```
AtBglu5          ----------------------------------------------------------------------------------------------------
AtBglu7          --------------------SA------------------------------------------------------------------------------
AtBglu8          --------------------LA------------------------------------------------------------------------------
AtBglu9          --------------------LA------------------------------------------------------------------------------
BD1G10890        --------------------AAY------HGGGASV--------R-----------------------------------------------AA------
BD1G10920        --------------------AAY------HGGGASV--------R-----------------------------------------------AA------
BD1G10930        --------------------AAY------HGGGASV--------R-----------------------------------------------AA------
BD1G10940        --------------------VLL-G----D--GTGR--------V-------------------------------------------------------
BD1G19270        --------------------VLL-A----QCH--------------------------------------------------------------------
BD1G33040        --------------------VS--F---------------------------------------------------------------------------
BD1G42690        --------------------ALQ-L----QV---------------------------------------------------------------------
BD1G70170        --------------------AS-------SLTG-C-----------------------------------------------------------------
BD2G09190        --------------------FSV-V----G-----S------AAP-------------------------------------------------------
BD2G09200        --------------------VSV-V----G-----S------AAP-------------------------------------------------------
BD2G27770        --------------------ALW-Q----G----------------------------------------------------------------------
BD2G37450        ----------------------------------------------------------------------------------------------------
BD2G57640        --------------------D-------------------------------------------------------------------------------
BD2G59650        --------------------LLS-----------------------------------------------------------------------------
BD2G59660        --------------------SAA-H----G----------------------------------------------------------------------
BD3G00650        --------------------LSM-A-------T-T-----------------------------------------------------------------
BD3G40000        --------------------ALL-L----AC--S------------------------------------------------------------------
BD3G40010        --------------------ALA-V----GG---------------------------------------------------------------------
BD3G45610        --------------------WVQ------D----------------------------------------------------------------------
BD3G45630        --------------------WVH------D----------------------------------------------------------------------
BD3G45640        --------------------WVQ------D----------------------------------------------------------------------
BD3G45650        --------------------WVQ------D----------------------------------------------------------------------
BD4G08040        --------------------LLA-H---------------------------------------------------------------------------
BD4G09920        --------------------AAA-T----AANAASY--------G-------------------------------------------------------
BD4G34930        --------------------A-A-----------------------------------------------------------------------------
BD4G34940        --------------------VAA-L---------------------------------------------------------------------------
BD4G34950        --------------------LTA-----------------------------------------------------------------------------
BD5G13260        --------------------VAA-A----SG---------------------------------------------------------------------
BD5G13270        ---------------------SS-K----E--AA------------------------------------------------------------------
BD5G15530        --------------------QL--F----F----------------------------------------------------------------------
BD5G15540        --------------------LA--A----A----------------------------------------------------------------------
GRMZM2G012236    --------------------LL-------H----------------------------------------------------------------------
GRMZM2G015804    -----------------------------GRR--------------------------------------------------------------------
GRMZM2G031660    --------------------LL--P----R----------------------------------------------------------------------
GRMZM2G055699    --------------------APW-Q----D----------------------------------------------------------------------
GRMZM2G069024    --------------------CVQ------S----------------------------------------------------------------------
GRMZM2G108133    --------------------AVA-P----------AELSVLGLGG-------------------------------------------------------
GRMZM2G110948    --------------------AGG-A----RASYD-G--------E-----------------------------------------------GE------
GRMZM2G112704    --------------------SLA-L----GAHGVN-----------------------------------------------------------------
GRMZM2G128197    --------------------VAP-L----GSRGLRV--------R-------------------------------------------------------
GRMZM2G148176    --------------------AAA-H----HGLLPLP--------T-----------------------------------------------AR------
GRMZM2G163544    --------------------IS--F---------------------------------------------------------------------------
GRMZM2G177661    --------------------VSL-Q----GCI--A-----------------------------------------------------------------
GRMZM2G376416    ----------------------------------------------------------------------------------------------------
```

```
GRMZM2G457040  --------------------AL--L----A----------------------------------------------------------------------
Os10bglu34     --------------------MSQ-G-------C-D-----------------------------------------------------------------
Os11bglu36     --------------------AAA-T----AANAASY--------A-------------------------------------------------------
Os12bglu38     --------------------SLS-H----G----------------------------------------------------------------------
Os1bglu1       --------------------AGA-A----RAAEQ-A--------A-----------------------------------------------GE------
Os1bglu2       --------------------SVQ-----------------------------------------------------------------------------
Os1bglu3       --------------------SVQ-----------------------------------------------------------------------------
Os1bglu4       ----------------------------------------------------------------------------------------------------
Os1bglu5       --------------------LLH------G----------------------------------------------------------------------
Os3bglu6       --------------------VAS-S----SLRG-C-----------------------------------------------------------------
Os3bglu7       --------------------LLA-A----RDAGAAA--------V-------------------------------------------------------
Os3bglu8       --------------------VLD-R----A--GARV--------R-------------------------------------------------------
Os4bglu10      --------------------AFT-C----A--AY------------------------------------------------------------------
Os4bglu11      --------------------AFT-C----V--AC------------------------------------------------------------------
Os4bglu12      --------------------AVV-A----SG-AY------------------------------------------------------------------
Os4bglu13      --------------------VVA-V----S----------------------------------------------------------------------
Os4bglu14      --------------------LL--H----L----------------------------------------------------------------------
Os4bglu16      --------------------AV--L----A----------------------------------------------------------------------
Os4bglu18      --------------------LP------------------------------------------------------------------------------
Os4bglu27      --------------------ALV-S----N----------------------------------------------------------------------
Os4bglu28      --------------------ALA-C----S----------------------------------------------------------------------
Os4bglu9       --------------------LLA-A----A--CV------------------------------------------------------------------
Os5bglu19      --------------------LVV------Q----------------------------------------------------------------------
Os5bglu21      --------------------LLL------Q----------------------------------------------------------------------
Os5bglu22      --------------------AAPWRSGEAA----------------------------------------------------------------------
Os5bglu23      --------------------LVA-G----E----------------------------------------------------------------------
Os5gblu20      --------------------LLL------Q----------------------------------------------------------------------
Os6bglu24      --------------------ASS-T----SS-RS------------------------------------------------------------------
Os6bglu25      --------------------VS--F---------------------------------------------------------------------------
Os7bglu26      --------------------AAA-H----L-LLTLP--------P-----------------------------------------------AQ------
Os9bglu29      --------------------AVL-C---------------------------------------------------------------------------
Os9bglu30      --------------------ALF-C---------------------------------------------------------------------------
Os9bglu31      --------------------AAA-A---------------------------------------------------------------------------
Os9bglu32      --------------------LAA-----------------------------------------------------------------------------
Os9bglu33      --------------------LGA-----------------------------------------------------------------------------
SB01G010825    --------------------VVP-L----GSRGLRV--------R-------------------------------------------------------
SB01G010830    --------------------ACG-G----ARAGAAA--------A-----------------------------------------------AA------
SB01G013360    ----------------------------------------------------------------------------------------------------
SB01G043030    --------------------VSL-Q----GRC--------------------------------------------------------------------
SB02G028400    --------------------ATA-C---------------------------------------------------------------------------
SB02G029620    --------------------VLA-----------------------------------------------------------------------------
SB02G041550    --------------------AAA-H----HGLLPLP--------T-----------------------------------------------AL------
SB03G037780    --------------------CVQ------G----------------------------------------------------------------------
SB03G042690    SHAFHSSAPATPPLSPPAGAM-------------------------------------------------------------------------------
SB06G019830    ----------------------------------------------------------------------------------------------------
SB06G019840    --------------------AVA------SD-AY------------------------------------------------------------------
SB06G019850    --------------------LAA-A----SN-AC----------------------------------------------------------D-------
SB06G019860    --------------------AAA-A----SN-AS----------------------------------------------------------Y-------
```

```
SB06G019880    ----------------------------------------------------------------------------------------------------
SB06G022410    --------------------LL-------C----------------------------------------------------------------------
SB06G022420    --------------------VV-------A----------------------------------------------------------------------
SB06G022450    ---------------------L--L----P----------------------------------------------------------------------
SB06G022460    --------------------LL--L----P----------------------------------------------------------------------
SB06G022490    --------------------AL--L----A----------------------------------------------------------------------
SB06G022500    --------------------LD--L----P----------------------------------------------------------------------
SB06G022510    --------------------LD--L----P----------------------------------------------------------------------
SB08G007586    --------------------T-A-H----PA-ELRS--------HL-GRPNNESFSRHHLCSS-PQNISKRRSNLSFRPRAQTISS----EPG-------
SB08G007650    --------------------S-A-H----PA-GLRS--------Q----SNNQSFSRHHLCSS-PKNISKRRCKLSFRPRAERVGS----ENG-------
SB09G018160    --------------------LPS------H----------------------------------------------------------------------
SB10G012220    --------------------FLV-V----VS---------------------------------------------------------------------
SB10G022300    --------------------LLP-S----T-----H--------V-------------------------------------------------------
SB10G027600    --------------------VS--F---------------------------------------------------------------------------
SB10G028060    --------------------SVA-L----GAHDVN-----------------------------------------------------------------
SbDhr1         --------------------T-A-H----PA-GLRS--------H----PNNESFSRHHLCSS-PQNISKRRSNLSFRPRAQTISSE---SAG-------
SbDhr2         --------------------SHS-A----HP-GLRS--------H-IG-PNNEHISRH-LSSS-SQN-TKRRCNISLRSRAQRISS----QLG-------
Zm-p60.1       --------------------A-A-A----HP-GLRS--------HLVG-PNNESFSRHHLPSSSPQS-SKRRCNLSFTTRSARVGS----QNG-------
ZmGLU2         --------------------A-A-H-----P-VLRS--------H-LG-PNNESFSRHHL-SSSPQS-SKRRFNLSFTPRSARVGN----QNG-------
ZmGLU3         --------------------T-A-H----R-----S--------HIVG-PNNENFPRHQ-PCSS-QN-RNKR--LRLRSRAQRISS---QLLA-------
ZmGLU4         -------------------------------------------------PNHESFSR-------------------------------------------
ZmGLU5         --------------------H-A-V----AH-L-------------LG-PNHESFSRHHLSSSLQQN-SKRRCNLSFRPRAA--ES----QNG-------
ZmGLU6         --------------------T-R------RY-E-------------DG-------------------------------------D----DDA-------
ZmGLU7         --------------------C---H-----P-GLTS--------S-R-----HDNGRHGLSSSP-GSSSKRSRSLSFIPRAEKTTTGGEGHVV-------
ZmGLU8         --------------------C---R-----P-GLTS--------R-HA----HDNGRHGLSSSPPGSRSKRSRSLSLRPRAEKTTTGGGGHVV-------
```

```
                         310       320       330       340       350       360       370       380       390       400
                  ....|....|....|....|....|....|....|....|....|....|....|....|....|....|....|....|....|....|....|....|
AC155376.2_FGT005 ---------------------------------------------------------------------------GREPE--------------------
AC211140.2_FGT007 -----------------------------------------------Q-------------------------GGGGGGG--------------------
AC217401.3_FGT001 -----------------------------------------------E-------------------------GPNWLGG--------------------
AC217687.3_FGT004 -----------------------------------------------K-------------------------GANWLGG--------------------
AtBglu1           -----------------------------------------------F-------------------------GKCSSDL--------------------
AtBglu10          ---------------------------------------------------------------------------TSDSDA--------------------
AtBglu11          -----------------------------------------------T--------------------------AVSSLK--------------------
AtBglu12          -------------------------------------------------------------------------KHSSTPK--------------------
AtBglu13          -------------------------------------------------------------------------KHSSTPK--------------------
AtBglu14          -------------------------------------------------------------------------RHSSTPK--------------------
AtBglu15          -------------------------------------------------------------------------NNSSTPK--------------------
AtBglu16          -------------------------------------------------------------------------KHSTRPR--------------------
AtBglu17          ----------------------------------------------PS-------------------------FLRLSTS--------------------
AtBglu18          -------------------------------------------------------------------------GAGLPDK--------------------
AtBglu19          -------------------------------------------------------------------------VCPKTET--------------------
AtBglu2           -------------------------------------------------------------------------M--------------------------
AtBglu20          -------------------------------------------------------------------------CSSTDIH--------------------
AtBglu21          -------------------------------------------------------------------------VCPATSK--------------------
AtBglu22          -------------------------------------------------------------------------VCPTTSK--------------------
AtBglu23          -------------------------------------------------------------------------VCPPSNK--------------------
AtBglu24          -------------------------------------------------------------------------PPKPSDK--------------------
AtBglu25          -------------------------------------------------------------------------VCPASST--------------------
AtBglu26          --------------------------------------------------------------------------FPTEMS--------------------
AtBglu27          ----------------------------------------------------------------------------KKNS--------------------
AtBglu28          -------------------------------------------------------------------------LPPDSRV--------------------
AtBglu29          -------------------------------------------------------------------------LPPESQV--------------------
AtBglu3           -------------------------------------------------------------------------G----RC--------------------
AtBglu30          ---------------------------------------------------------------------------INSLE--------------------
AtBglu31          -------------------------------------------------------------------------LRFSTTP--------------------
AtBglu32          -------------------------------------------------------------------------LRFSTTP--------------------
AtBglu33          IGPHQTSFDDEIGIVIGPHATVDDEDIDMDMGTTVGPQTNLNDDDLGT-------------------------IIGPEFE--------------------
AtBglu34          ----------------------------------------------PF-------------------------HCDNTHA--------------------
AtBglu35          ----------------------------------------------PF-------------------------HCDNTHA--------------------
AtBglu36          -------------------------------------------------------------------------------I--------------------
AtBglu37          ----------------------------------------------PF-------------------------TCSQTDR--------------------
AtBglu38          ----------------------------------------------PF-------------------------TCNQTKL--------------------
AtBglu39          ----------------------------------------------PF-------------------------TCNQTDR--------------------
AtBglu4           -----------------------------------------------S--------------------------GKCSDV--------------------
AtBglu40          -----------------------------------------------D-------------------------KTCICAD--------------------
AtBglu41          -----------------------------------------------L-------------------------DHVSSES--------------------
AtBglu42          -------------------------------------------------------------------------NLAVPPV--------------------
AtBglu43          ----------------------------------------------DA-------------------------VPLATGG--------------------
AtBglu44          ----------------------------------------------EK-------------------------NKLHTGG--------------------
AtBglu45          -------------------------------------------------------------------------SSSKNIL--------------------
AtBglu46          -------------------------------------------------------------------------SCLHQTS--------------------
AtBglu47          ----------------------------------------------HI-------------------------SLKEIHA--------------------
```

```
AtBglu5         ----------------------------------------------------------------------------------------------------
AtBglu7         --------------------------------------------------------------------------TSYIDA--------------------
AtBglu8         --------------------------------------------------------------------------TSYIDA--------------------
AtBglu9         --------------------------------------------------------------------------TSYSDA--------------------
BD1G10890       ----------------------------------------V-----GA-------------------------GAHWLGG--------------------
BD1G10920       ----------------------------------------AG---AGA-------------------------GSHWLGG--------------------
BD1G10930       ----------------------------------------A-----GA-------------------------GAHWLGG--------------------
BD1G10940       -----------------------------------------------Q-------------------------AAVDTGG--------------------
BD1G19270       ----------------------------------------------RP-------------------------EIHDTGG--------------------
BD1G33040       --------------------------------------------------------------------------AASAEA--------------------
BD1G42690       -------------------------------------------------------------------------D-TTPSE--------------------
BD1G70170       -----------------------------------------------I-------------------------AASGGAG--------------------
BD2G09190       -----------------------------------------------S-------------------------PARSEGI--------------------
BD2G09200       -----------------------------------------------S-------------------------SARSEGI--------------------
BD2G27770       -----------------------------------------------G-------------------------TAARALD--------------------
BD2G37450       ----------------------------------------------------------------------------------------------------
BD2G57640       -----------------------------------------------E-------------------------EAVAAAE--------------------
BD2G59650       ---------------------------------------------------------------------------AAALG--------------------
BD2G59660       -----------------------------------------------A-------------------------AAPAPDG--------------------
BD3G00650       -----------------------------------------------T-------------------------RGQQRSN--------------------
BD3G40000       -------------------------------------------------------------------------GGIHGAT--------------------
BD3G40010       -------------------------------------------------------------------------ARGAGSS--------------------
BD3G45610       -------------------------------------------------------------------------A-AAIID--------------------
BD3G45630       -------------------------------------------------------------------------A-AAIIG--------------------
BD3G45640       -------------------------------------------------------------------------A-AAIIG--------------------
BD3G45650       -------------------------------------------------------------------------A-AATIG--------------------
BD4G08040       ---------------------------------------------------------------------------GKPGD--------------------
BD4G09920       -----------------------------------------------RFRRRNLRRIACPIDDSDDPVADFRALPLSSSS--------------------
BD4G34930       --------------------------------------------------------------------------SAAAGK--------------------
BD4G34940       -----------------------------------------------T-------------------------TPRGASA--------------------
BD4G34950       -------------------------------------------------------------------------LPRDSAA--------------------
BD5G13260       --------------------------------------------------------------------------AGHQEL--------------------
BD5G13270       -----------------------------------------------N-------------------------NS-QETY--------------------
BD5G15530       ---------------------------------------------------------------------------SCASA--------------------
BD5G15540       ---------------------------------------------------------------------------PSARG--------------------
GRMZM2G012236   ---------------------------------------------------------------------------SSVTG--------------------
GRMZM2G015804   -------------------------------------------------------------------------R-----R--------------------
GRMZM2G031660   ---------------------------------------------------------------------------ATAAA--------------------
GRMZM2G055699   -----------------------------------------------E-------------------------TAARALN--------------------
GRMZM2G069024   -------------------------------------------------------------------------A-APVLG--------------------
GRMZM2G108133   -----------------------------------------------G-------------------------AAAASGA--------------------
GRMZM2G110948   ----------------------------------AA---RAGAEEKEK-------------------------AAAWTGG--------------------
GRMZM2G112704   -----------------------------------------------V-------------------------KPGEHHI--------------------
GRMZM2G128197   -----------------------------------------------A-------------------------AGADTGG--------------------
GRMZM2G148176   ------------------------------------------CYWLNP-------------------------EIYDAGG--------------------
GRMZM2G163544   --------------------------------------------------------------------------AACAEA--------------------
GRMZM2G177661   -----------------------------------------------Q-------------------------GGGGAGG--------------------
GRMZM2G376416   ----------------------------------------------------------------------------------------------------
```

```
GRMZM2G457040   --------------------------------------------------------------------------PAVRG--------------------
Os10bglu34      ------------------------------------------------------------------------AQNTTGG--------------------
Os11bglu36      ----------------------------------------------RYRRRHLRRIPSPIDESADPLADFRAFP---SS--------------------
Os12bglu38      -------------------------------------------------------------------------NGEQTD--------------------
Os1bglu1        ---------------------------------DGIRGGAGA-DHQE-------------------------AAGITGG--------------------
Os1bglu2        --------------------------------------------------------------------------GGAVG--------------------
Os1bglu3        ------------------------------------------------------------------------H-GVLGG--------------------
Os1bglu4        --------------------------------------------------------------------------GRDAE--------------------
Os1bglu5        ------------------------------------------------------------------------AAPAVLG--------------------
Os3bglu6        ----------------------------------------------I-------------------------AQQSGGG--------------------
Os3bglu7        ----------------------------------------------P-------------------------KPNWLGG--------------------
Os3bglu8        ----------------------------------------------A-------------------------ADDDTGG--------------------
Os4bglu10       ----------------------------------------------N-------------------------DAGELPP--------------------
Os4bglu11       ----------------------------------------------N-------------------------GGSELPP--------------------
Os4bglu12       ----------------------------------------------N--------------------------GAGEPP--------------------
Os4bglu13       --------------------------------------------------------------------------GEPPP--------------------
Os4bglu14       --------------------------------------------------------------------------SGVSA--------------------
Os4bglu16       --------------------------------------------------------------------------PAARG--------------------
Os4bglu18       ---------------------------------------------------------------------------LASA--------------------
Os4bglu27       ------------------------------------------------------------------------GAAVHGA--------------------
Os4bglu28       ------------------------------------------------------------------------SNRVHGA--------------------
Os4bglu9        ------------------------------------------------------------------------EAGELPP--------------------
Os5bglu19       ------------------------------------------------------------------------G-VRSLQ--------------------
Os5bglu21       ------------------------------------------------------------------------G-VSSLQ--------------------
Os5bglu22       ----------------------------------------------A-------------------------AAARALN--------------------
Os5bglu23       ----------------------------------------------A-------------------------TAEAALN--------------------
Os5gblu20       ------------------------------------------------------------------------G-VSSLQ--------------------
Os6bglu24       ------------------------------------------------------------------------EMKAGEV--------------------
Os6bglu25       -------------------------------------------------------------------------SACVEA--------------------
Os7bglu26       -----------------------------------------CYWLNP-------------------------EIYDAGG--------------------
Os9bglu29       -------------------------------------------------------------------------SGVDAS--------------------
Os9bglu30       -------------------------------------------------------------------------NGVYAK--------------------
Os9bglu31       ----------------------------------------------A-------------------------SSSTAAG--------------------
Os9bglu32       ------------------------------------------------------------------------AARDASA--------------------
Os9bglu33       ------------------------------------------------------------------------VAREASA--------------------
SB01G010825     ----------------------------------------------A-------------------------VGADTGG--------------------
SB01G010830     ---------------------------------------AG--GGGA-------------------------GASWLGG--------------------
SB01G013360     ---------------------------------------------------------------------------------------------------
SB01G043030     ------------------------------------------------------------------------TAQGGGG--------------------
SB02G028400     -------------------------------------------------------------------------IGVHAK--------------------
SB02G029620     -------------------------------------------------------------------------AAPAAS--------------------
SB02G041550     -----------------------------------------CYWLNP-------------------------EIYDAGG--------------------
SB03G037780     ------------------------------------------------------------------------A-APVLS--------------------
SB03G042690     ----------------------------------------------G-------------------------STGRDPE--------------------
SB06G019830     ---------------------------------------------------------------------------------------------------
SB06G019840     ----------------------------------------------D--------------------------GGGQPP--------------------
SB06G019850     ----------------------------------------------C-------------------------AGRQPPI--------------------
SB06G019860     ----------------------------------------------D-------------------------GAAGQPP--------------------
```

```
SB06G019880    ----------------------------------------------------------------------------------------------------
SB06G022410    ---------------------------------------------------------------------------PSITG--------------------
SB06G022420    ---------------------------------------------------------------------------AVVLPPRFPPPPRRRLDVPRLLEER
SB06G022450    ---------------------------------------------------------------------------CASSA--------------------
SB06G022460    ---------------------------------------------------------------------------CASSA--------------------
SB06G022490    ---------------------------------------------------------------------------PAARG--------------------
SB06G022500    ---------------------------------------------------------------------------WATAA--------------------
SB06G022510    ---------------------------------------------------------------------------WATAA--------------------
SB08G007586    ----------------------------------------------IH-------------------------MLSPWEI--------------------
SB08G007650    ----------------------------------------------NH-------------------------RLSPREI--------------------
SB09G018160    -------------------------------------------------------------------------D-VTAFR--------------------
SB10G012220    -------------------------------------------------------------------------AVKASSN--------------------
SB10G022300    -----------------------------------------------S-------------------------SAAAAAA--------------------
SB10G027600    --------------------------------------------------------------------------AACAEA--------------------
SB10G028060    -------------------------------------------------------------------------KPGGHYN--------------------
SbDhr1         ----------------------------------------------IH-------------------------RLSPWEI--------------------
SbDhr2         ----------------------------------------------GQ-------------------------KLEHWEI--------------------
Zm-p60.1       ----------------------------------------------VQ-------------------------MLSPSEI--------------------
ZmGLU2         ----------------------------------------------VQ-------------------------LLSPSEI--------------------
ZmGLU3         ----------------------------------------------SR-------------------------KLMPWQI--------------------
ZmGLU4         ---------------------------------------------------------------------------HHLSC--------------------
ZmGLU5         ----------------------------------------------SQ-------------------------TLSPSEV--------------------
ZmGLU6         ----------------------------------------------KQ-------------------------MLRPWEV--------------------
ZmGLU7         ----------------------------------------------SE-------------------------KLSPWQI--------------------
ZmGLU8         ----------------------------------------------SE-------------------------KLSPWQI--------------------
```

```
                           410       420       430       440       450       460       470       480       490       500
                    ....|....|....|....|....|....|....|....|....|....|....|....|....|....|....|....|....|....|....|....|
AC155376.2_FGT005   ----------------------------------------------------------------------------------------------------
AC211140.2_FGT007   ----------------------------------------------------------------------------------------------------
AC217401.3_FGT001   ----------------------------------------------------------------------------------------------------
AC217687.3_FGT004   ----------------------------------------------------------------------------------------------------
AtBglu1             ----------------------------------------------------------------------------------------------------
AtBglu10            ----------------------------------------------------------------------------------------------------
AtBglu11            ----------------------------------------------------------------------------------------------------
AtBglu12            ----------------------------------------------------------------------------------------------------
AtBglu13            ----------------------------------------------------------------------------------------------------
AtBglu14            ----------------------------------------------------------------------------------------------------
AtBglu15            ----------------------------------------------------------------------------------------------------
AtBglu16            ----------------------------------------------------------------------------------------------------
AtBglu17            ----------------------------------------------------------------------------------------------------
AtBglu18            ----------------------------------------------------------------------------------------------------
AtBglu19            ----------------------------------------------------------------------------------------------------
AtBglu2             ----------------------------------------------------------------------------------------------------
AtBglu20            ----------------------------------------------------------------------------------------------------
AtBglu21            ----------------------------------------------------------------------------------------------------
AtBglu22            ----------------------------------------------------------------------------------------------------
AtBglu23            ----------------------------------------------------------------------------------------------------
AtBglu24            ----------------------------------------------------------------------------------------------------
AtBglu25            ----------------------------------------------------------------------------------------------------
AtBglu26            ----------------------------------------------------------------------------------------------------
AtBglu27            ----------------------------------------------------------------------------------------------------
AtBglu28            ----------------------------------------------------------------------------------------------------
AtBglu29            ----------------------------------------------------------------------------------------------------
AtBglu3             ----------------------------------------------------------------------------------------------------
AtBglu30            ----------------------------------------------------------------------------------------------------
AtBglu31            ----------------------------------------------------------------------------------------------------
AtBglu32            ----------------------------------------------------------------------------------------------------
AtBglu33            ----------------------------------------------------------------------------------------------------
AtBglu34            ----------------------------------------------------------------------------------------------------
AtBglu35            ----------------------------------------------------------------------------------------------------
AtBglu36            ----------------------------------------------------------------------------------------------------
AtBglu37            ----------------------------------------------------------------------------------------------------
AtBglu38            ----------------------------------------------------------------------------------------------------
AtBglu39            ----------------------------------------------------------------------------------------------------
AtBglu4             ----------------------------------------------------------------------------------------------------
AtBglu40            ----------------------------------------------------------------------------------------------------
AtBglu41            ----------------------------------------------------------------------------------------------------
AtBglu42            ----------------------------------------------------------------------------------------------------
AtBglu43            ----------------------------------------------------------------------------------------------------
AtBglu44            ----------------------------------------------------------------------------------------------------
AtBglu45            ----------------------------------------------------------------------------------------------------
AtBglu46            ----------------------------------------------------------------------------------------------------
AtBglu47            ----------------------------------------------------------------------------------------------------
```

```
AtBglu5         ----------------------------------------------------------------------------------------------------
AtBglu7         ----------------------------------------------------------------------------------------------------
AtBglu8         ----------------------------------------------------------------------------------------------------
AtBglu9         ----------------------------------------------------------------------------------------------------
BD1G10890       ----------------------------------------------------------------------------------------------------
BD1G10920       ----------------------------------------------------------------------------------------------------
BD1G10930       ----------------------------------------------------------------------------------------------------
BD1G10940       ----------------------------------------------------------------------------------------------------
BD1G19270       ----------------------------------------------------------------------------------------------------
BD1G33040       ----------------------------------------------------------------------------------------------------
BD1G42690       ----------------------------------------------------------------------------------------------------
BD1G70170       ----------------------------------------------------------------------------------------------------
BD2G09190       ----------------------------------------------------------------------------------------------------
BD2G09200       ----------------------------------------------------------------------------------------------------
BD2G27770       ----------------------------------------------------------------------------------------------------
BD2G37450       ----------------------------------------------------------------------------------------------------
BD2G57640       ----------------------------------------------------------------------------------------------------
BD2G59650       ----------------------------------------------------------------------------------------------------
BD2G59660       ----------------------------------------------------------------------------------------------------
BD3G00650       ----------------------------------------------------------------------------------------------------
BD3G40000       ----------------------------------------------------------------------------------------------------
BD3G40010       ----------------------------------------------------------------------------------------------------
BD3G45610       ----------------------------------------------------------------------------------------------------
BD3G45630       ----------------------------------------------------------------------------------------------------
BD3G45640       ----------------------------------------------------------------------------------------------------
BD3G45650       ----------------------------------------------------------------------------------------------------
BD4G08040       ----------------------------------------------------------------------------------------------------
BD4G09920       ----------------------------------------------------------------------------------------------------
BD4G34930       ----------------------------------------------------------------------------------------------------
BD4G34940       ----------------------------------------------------------------------------------------------------
BD4G34950       ----------------------------------------------------------------------------------------------------
BD5G13260       ----------------------------------------------------------------------------------------------------
BD5G13270       ----------------------------------------------------------------------------------------------------
BD5G15530       ----------------------------------------------------------------------------------------------------
BD5G15540       ----------------------------------------------------------------------------------------------------
GRMZM2G012236   ----------------------------------------------------------------------------------------------------
GRMZM2G015804   ----------------------------------------------------------------------------------------------------
GRMZM2G031660   ----------------------------------------------------------------------------------------------------
GRMZM2G055699   ----------------------------------------------------------------------------------------------------
GRMZM2G069024   ----------------------------------------------------------------------------------------------------
GRMZM2G108133   ----------------------------------------------------------------------------------------------------
GRMZM2G110948   ----------------------------------------------------------------------------------------------------
GRMZM2G112704   ----------------------------------------------------------------------------------------------------
GRMZM2G128197   ----------------------------------------------------------------------------------------------------
GRMZM2G148176   ----------------------------------------------------------------------------------------------------
GRMZM2G163544   ----------------------------------------------------------------------------------------------------
GRMZM2G177661   ----------------------------------------------------------------------------------------------------
GRMZM2G376416   ----------------------------------------------------------------------------------------------------
```

```
GRMZM2G457040      ----------------------------------------------------------------------------------------------------
Os10bglu34         ----------------------------------------------------------------------------------------------------
Os11bglu36         ----------------------------------------------------------------------------------------------------
Os12bglu38         ----------------------------------------------------------------------------------------------------
Os1bglu1           ----------------------------------------------------------------------------------------------------
Os1bglu2           ----------------------------------------------------------------------------------------------------
Os1bglu3           ----------------------------------------------------------------------------------------------------
Os1bglu4           ----------------------------------------------------------------------------------------------------
Os1bglu5           ----------------------------------------------------------------------------------------------------
Os3bglu6           ----------------------------------------------------------------------------------------------------
Os3bglu7           ----------------------------------------------------------------------------------------------------
Os3bglu8           ----------------------------------------------------------------------------------------------------
Os4bglu10          ----------------------------------------------------------------------------------------------------
Os4bglu11          ----------------------------------------------------------------------------------------------------
Os4bglu12          ----------------------------------------------------------------------------------------------------
Os4bglu13          ----------------------------------------------------------------------------------------------------
Os4bglu14          ----------------------------------------------------------------------------------------------------
Os4bglu16          ----------------------------------------------------------------------------------------------------
Os4bglu18          ----------------------------------------------------------------------------------------------------
Os4bglu27          ----------------------------------------------------------------------------------------------------
Os4bglu28          ----------------------------------------------------------------------------------------------------
Os4bglu9           ----------------------------------------------------------------------------------------------------
Os5bglu19          ----------------------------------------------------------------------------------------------------
Os5bglu21          ----------------------------------------------------------------------------------------------------
Os5bglu22          ----------------------------------------------------------------------------------------------------
Os5bglu23          ----------------------------------------------------------------------------------------------------
Os5gblu20          ----------------------------------------------------------------------------------------------------
Os6bglu24          ----------------------------------------------------------------------------------------------------
Os6bglu25          ----------------------------------------------------------------------------------------------------
Os7bglu26          ----------------------------------------------------------------------------------------------------
Os9bglu29          ----------------------------------------------------------------------------------------------------
Os9bglu30          ----------------------------------------------------------------------------------------------------
Os9bglu31          ----------------------------------------------------------------------------------------------------
Os9bglu32          ----------------------------------------------------------------------------------------------------
Os9bglu33          ----------------------------------------------------------------------------------------------------
SB01G010825        ----------------------------------------------------------------------------------------------------
SB01G010830        ----------------------------------------------------------------------------------------------------
SB01G013360        ----------------------------------------------------------------------------------------------------
SB01G043030        ----------------------------------------------------------------------------------------------------
SB02G028400        ----------------------------------------------------------------------------------------------------
SB02G029620        ----------------------------------------------------------------------------------------------------
SB02G041550        ----------------------------------------------------------------------------------------------------
SB03G037780        ----------------------------------------------------------------------------------------------------
SB03G042690        ----------------------------------------------------------------------------------------------------
SB06G019830        ----------------------------------------------------------------------------------------------------
SB06G019840        ----------------------------------------------------------------------------------------------------
SB06G019850        ----------------------------------------------------------------------------------------------------
SB06G019860        ----------------------------------------------------------------------------------------------------
```

```
SB06G019880     ----------------------------------------------------------------------------------------------------
SB06G022410     ----------------------------------------------------------------------------------------------------
SB06G022420     LLESLRAMNHADGAHTDNPVPRTVSNLLVHVLGTHVDHVHDIVTRLEMDLDAIELQLDKGERSEVVVGGCFLAGRGRRQSYMAGSGGAQATTPTLPPTGS
SB06G022450     ----------------------------------------------------------------------------------------------------
SB06G022460     ----------------------------------------------------------------------------------------------------
SB06G022490     ----------------------------------------------------------------------------------------------------
SB06G022500     ----------------------------------------------------------------------------------------------------
SB06G022510     ----------------------------------------------------------------------------------------------------
SB08G007586     ----------------------------------------------------------------------------------------------------
SB08G007650     ----------------------------------------------------------------------------------------------------
SB09G018160     ----------------------------------------------------------------------------------------------------
SB10G012220     ----------------------------------------------------------------------------------------------------
SB10G022300     ----------------------------------------------------------------------------------------------------
SB10G027600     ----------------------------------------------------------------------------------------------------
SB10G028060     ----------------------------------------------------------------------------------------------------
SbDhr1          ----------------------------------------------------------------------------------------------------
SbDhr2          ----------------------------------------------------------------------------------------------------
Zm-p60.1        ----------------------------------------------------------------------------------------------------
ZmGLU2          ----------------------------------------------------------------------------------------------------
ZmGLU3          ----------------------------------------------------------------------------------------------------
ZmGLU4          ----------------------------------------------------------------------------------------------------
ZmGLU5          ----------------------------------------------------------------------------------------------------
ZmGLU6          ----------------------------------------------------------------------------------------------------
ZmGLU7          ----------------------------------------------------------------------------------------------------
ZmGLU8          ----------------------------------------------------------------------------------------------------
```

```
                            510       520       530       540       550       560       570       580       590       600
                    ....|....|....|....|....|....|....|....|....|....|....|....|....|....|....|....|....|....|....|....|
AC155376.2_FGT005   ----------------------------------------------------------------------------------------------------
AC211140.2_FGT007   ----------------------------------------------------------------------------------------------------
AC217401.3_FGT001   ----------------------------------------------------------------------------------------------------
AC217687.3_FGT004   ----------------------------------------------------------------------------------------------------
AtBglu1             ----------------------------------------------------------------------------------------------------
AtBglu10            ----------------------------------------------------------------------------------------------------
AtBglu11            ----------------------------------------------------------------------------------------------------
AtBglu12            ----------------------------------------------------------------------------------------------------
AtBglu13            ----------------------------------------------------------------------------------------------------
AtBglu14            ----------------------------------------------------------------------------------------------------
AtBglu15            ----------------------------------------------------------------------------------------------------
AtBglu16            ----------------------------------------------------------------------------------------------------
AtBglu17            ----------------------------------------------------------------------------------------------------
AtBglu18            ----------------------------------------------------------------------------------------------------
AtBglu19            ----------------------------------------------------------------------------------------------------
AtBglu2             ----------------------------------------------------------------------------------------------------
AtBglu20            ----------------------------------------------------------------------------------------------------
AtBglu21            ----------------------------------------------------------------------------------------------------
AtBglu22            ----------------------------------------------------------------------------------------------------
AtBglu23            ----------------------------------------------------------------------------------------------------
AtBglu24            ----------------------------------------------------------------------------------------------------
AtBglu25            ----------------------------------------------------------------------------------------------------
AtBglu26            ----------------------------------------------------------------------------------------------------
AtBglu27            ----------------------------------------------------------------------------------------------------
AtBglu28            ----------------------------------------------------------------------------------------------------
AtBglu29            ----------------------------------------------------------------------------------------------------
AtBglu3             ----------------------------------------------------------------------------------------------------
AtBglu30            ----------------------------------------------------------------------------------------------------
AtBglu31            ----------------------------------------------------------------------------------------------------
AtBglu32            ----------------------------------------------------------------------------------------------------
AtBglu33            ----------------------------------------------------------------------------------------------------
AtBglu34            ----------------------------------------------------------------------------------------------------
AtBglu35            ----------------------------------------------------------------------------------------------------
AtBglu36            ----------------------------------------------------------------------------------------------------
AtBglu37            ----------------------------------------------------------------------------------------------------
AtBglu38            ----------------------------------------------------------------------------------------------------
AtBglu39            ----------------------------------------------------------------------------------------------------
AtBglu4             ----------------------------------------------------------------------------------------------------
AtBglu40            ----------------------------------------------------------------------------------------------------
AtBglu41            ----------------------------------------------------------------------------------------------------
AtBglu42            ----------------------------------------------------------------------------------------------------
AtBglu43            ----------------------------------------------------------------------------------------------------
AtBglu44            ----------------------------------------------------------------------------------------------------
AtBglu45            ----------------------------------------------------------------------------------------------------
AtBglu46            ----------------------------------------------------------------------------------------------------
AtBglu47            ----------------------------------------------------------------------------------------------------
```

```
AtBglu5             ----------------------------------------------------------------------------------------------------
AtBglu7             ----------------------------------------------------------------------------------------------------
AtBglu8             ----------------------------------------------------------------------------------------------------
AtBglu9             ----------------------------------------------------------------------------------------------------
BD1G10890           ----------------------------------------------------------------------------------------------------
BD1G10920           ----------------------------------------------------------------------------------------------------
BD1G10930           ----------------------------------------------------------------------------------------------------
BD1G10940           ----------------------------------------------------------------------------------------------------
BD1G19270           ----------------------------------------------------------------------------------------------------
BD1G33040           ----------------------------------------------------------------------------------------------------
BD1G42690           ----------------------------------------------------------------------------------------------------
BD1G70170           ----------------------------------------------------------------------------------------------------
BD2G09190           ----------------------------------------------------------------------------------------------------
BD2G09200           ----------------------------------------------------------------------------------------------------
BD2G27770           ----------------------------------------------------------------------------------------------------
BD2G37450           ----------------------------------------------------------------------------------------------------
BD2G57640           ----------------------------------------------------------------------------------------------------
BD2G59650           ----------------------------------------------------------------------------------------------------
BD2G59660           ----------------------------------------------------------------------------------------------------
BD3G00650           ----------------------------------------------------------------------------------------------------
BD3G40000           ----------------------------------------------------------------------------------------------------
BD3G40010           ----------------------------------------------------------------------------------------------------
BD3G45610           ----------------------------------------------------------------------------------------------------
BD3G45630           ----------------------------------------------------------------------------------------------------
BD3G45640           ----------------------------------------------------------------------------------------------------
BD3G45650           ----------------------------------------------------------------------------------------------------
BD4G08040           ----------------------------------------------------------------------------------------------------
BD4G09920           ----------------------------------------------------------------------------------------------------
BD4G34930           ----------------------------------------------------------------------------------------------------
BD4G34940           ----------------------------------------------------------------------------------------------------
BD4G34950           ----------------------------------------------------------------------------------------------------
BD5G13260           ----------------------------------------------------------------------------------------------------
BD5G13270           ----------------------------------------------------------------------------------------------------
BD5G15530           ----------------------------------------------------------------------------------------------------
BD5G15540           ----------------------------------------------------------------------------------------------------
GRMZM2G012236       ----------------------------------------------------------------------------------------------------
GRMZM2G015804       ----------------------------------------------------------------------------------------------------
GRMZM2G031660       ----------------------------------------------------------------------------------------------------
GRMZM2G055699       ----------------------------------------------------------------------------------------------------
GRMZM2G069024       ----------------------------------------------------------------------------------------------------
GRMZM2G108133       ----------------------------------------------------------------------------------------------------
GRMZM2G110948       ----------------------------------------------------------------------------------------------------
GRMZM2G112704       ----------------------------------------------------------------------------------------------------
GRMZM2G128197       ----------------------------------------------------------------------------------------------------
GRMZM2G148176       ----------------------------------------------------------------------------------------------------
GRMZM2G163544       ----------------------------------------------------------------------------------------------------
GRMZM2G177661       ----------------------------------------------------------------------------------------------------
GRMZM2G376416       ----------------------------------------------------------------------------------------------------
```

```
GRMZM2G457040   --------------------------------------------------------------------------------------------------------------
Os10bglu34      --------------------------------------------------------------------------------------------------------------
Os11bglu36      --------------------------------------------------------------------------------------------------------------
Os12bglu38      --------------------------------------------------------------------------------------------------------------
Os1bglu1        --------------------------------------------------------------------------------------------------------------
Os1bglu2        --------------------------------------------------------------------------------------------------------------
Os1bglu3        --------------------------------------------------------------------------------------------------------------
Os1bglu4        --------------------------------------------------------------------------------------------------------------
Os1bglu5        --------------------------------------------------------------------------------------------------------------
Os3bglu6        --------------------------------------------------------------------------------------------------------------
Os3bglu7        --------------------------------------------------------------------------------------------------------------
Os3bglu8        --------------------------------------------------------------------------------------------------------------
Os4bglu10       --------------------------------------------------------------------------------------------------------------
Os4bglu11       --------------------------------------------------------------------------------------------------------------
Os4bglu12       --------------------------------------------------------------------------------------------------------------
Os4bglu13       --------------------------------------------------------------------------------------------------------------
Os4bglu14       --------------------------------------------------------------------------------------------------------------
Os4bglu16       --------------------------------------------------------------------------------------------------------------
Os4bglu18       --------------------------------------------------------------------------------------------------------------
Os4bglu27       --------------------------------------------------------------------------------------------------------------
Os4bglu28       --------------------------------------------------------------------------------------------------------------
Os4bglu9        --------------------------------------------------------------------------------------------------------------
Os5bglu19       --------------------------------------------------------------------------------------------------------------
Os5bglu21       --------------------------------------------------------------------------------------------------------------
Os5bglu22       --------------------------------------------------------------------------------------------------------------
Os5bglu23       --------------------------------------------------------------------------------------------------------------
Os5gblu20       --------------------------------------------------------------------------------------------------------------
Os6bglu24       --------------------------------------------------------------------------------------------------------------
Os6bglu25       --------------------------------------------------------------------------------------------------------------
Os7bglu26       --------------------------------------------------------------------------------------------------------------
Os9bglu29       --------------------------------------------------------------------------------------------------------------
Os9bglu30       --------------------------------------------------------------------------------------------------------------
Os9bglu31       --------------------------------------------------------------------------------------------------------------
Os9bglu32       --------------------------------------------------------------------------------------------------------------
Os9bglu33       --------------------------------------------------------------------------------------------------------------
SB01G010825     --------------------------------------------------------------------------------------------------------------
SB01G010830     --------------------------------------------------------------------------------------------------------------
SB01G013360     --------------------------------------------------------------------------------------------------------------
SB01G043030     --------------------------------------------------------------------------------------------------------------
SB02G028400     --------------------------------------------------------------------------------------------------------------
SB02G029620     --------------------------------------------------------------------------------------------------------------
SB02G041550     --------------------------------------------------------------------------------------------------------------
SB03G037780     --------------------------------------------------------------------------------------------------------------
SB03G042690     --------------------------------------------------------------------------------------------------------------
SB06G019830     --------------------------------------------------------------------------------------------------------------
SB06G019840     --------------------------------------------------------------------------------------------------------------
SB06G019850     --------------------------------------------------------------------------------------------------------------
SB06G019860     --------------------------------------------------------------------------------------------------------------
```

```
SB06G019880    ----------------------------------------------------------------------------------------------------
SB06G022410    ----------------------------------------------------------------------------------------------------
SB06G022420    RMTPAAAAPDDGGEGAPPGGAGVGGGSTSQGMARLVRKRQSKRARQSSQATSTTRRPPRRARRRHSSHGRTHRHHADHGRVLLERQPVPEAQEQREQETI
SB06G022450    ----------------------------------------------------------------------------------------------------
SB06G022460    ----------------------------------------------------------------------------------------------------
SB06G022490    ----------------------------------------------------------------------------------------------------
SB06G022500    ----------------------------------------------------------------------------------------------------
SB06G022510    ----------------------------------------------------------------------------------------------------
SB08G007586    ----------------------------------------------------------------------------------------------------
SB08G007650    ----------------------------------------------------------------------------------------------------
SB09G018160    ----------------------------------------------------------------------------------------------------
SB10G012220    ----------------------------------------------------------------------------------------------------
SB10G022300    ----------------------------------------------------------------------------------------------------
SB10G027600    ----------------------------------------------------------------------------------------------------
SB10G028060    ----------------------------------------------------------------------------------------------------
SbDhr1         ----------------------------------------------------------------------------------------------------
SbDhr2         ----------------------------------------------------------------------------------------------------
Zm-p60.1       ----------------------------------------------------------------------------------------------------
ZmGLU2         ----------------------------------------------------------------------------------------------------
ZmGLU3         ----------------------------------------------------------------------------------------------------
ZmGLU4         ----------------------------------------------------------------------------------------------------
ZmGLU5         ----------------------------------------------------------------------------------------------------
ZmGLU6         ----------------------------------------------------------------------------------------------------
ZmGLU7         ----------------------------------------------------------------------------------------------------
ZmGLU8         ----------------------------------------------------------------------------------------------------
```

```
                            610       620       630       640       650       660       670       680       690       700
                    ....|....|....|....|....|....|....|....|....|....|....|....|....|....|....|....|....|....|....|....|
AC155376.2_FGT005   --------VTRANFPD-GF---------------------------------------------------------------------------------
AC211140.2_FGT007   --------LTRGSFPK-GF---------------------------------------------------------------------------------
AC217401.3_FGT001   --------LSRAAFPK-DF---------------------------------------------------------------------------------
AC217687.3_FGT004   --------LSRASFPK-GF---------------------------------------------------------------------------------
AtBglu1             --------YSRSDFPE-GF---------------------------------------------------------------------------------
AtBglu10            --------FTRNNFPK-DF---------------------------------------------------------------------------------
AtBglu11            --------YSRNDFPP-GF---------------------------------------------------------------------------------
AtBglu12            --------LRRSDFPE-DF---------------------------------------------------------------------------------
AtBglu13            --------LRRSDFPK-DF---------------------------------------------------------------------------------
AtBglu14            --------LRKTDFPE-DF---------------------------------------------------------------------------------
AtBglu15            --------LRRSDFPE-DF---------------------------------------------------------------------------------
AtBglu16            --------LRRNDFPQ-DF---------------------------------------------------------------------------------
AtBglu17            --------LQRSSFPQ-DF---------------------------------------------------------------------------------
AtBglu18            --------FSRLNFPE-GF---------------------------------------------------------------------------------
AtBglu19            --------LSRASFPE-GF---------------------------------------------------------------------------------
AtBglu2             ----------------------------------------------------------------------------------------------------
AtBglu20            --------FTRANFPK-GF---------------------------------------------------------------------------------
AtBglu21            --------LSRASFPN-GF---------------------------------------------------------------------------------
AtBglu22            --------LSRASFPN-GF---------------------------------------------------------------------------------
AtBglu23            --------LSRASFPE-GF---------------------------------------------------------------------------------
AtBglu24            --------LSRAHFPK-GF---------------------------------------------------------------------------------
AtBglu25            --------FGRGSFPD-GF---------------------------------------------------------------------------------
AtBglu26            --------KGRASFPK-GF---------------------------------------------------------------------------------
AtBglu27            --------FGRSDFPE-GF---------------------------------------------------------------------------------
AtBglu28            --------FDRHGFPD-NF---------------------------------------------------------------------------------
AtBglu29            --------LDRSSFPD-DF---------------------------------------------------------------------------------
AtBglu3             --------SDKNDFPE-GF---------------------------------------------------------------------------------
AtBglu30            --------LDRHSFPD-DF---------------------------------------------------------------------------------
AtBglu31            --------LNRYSFPP-HF---------------------------------------------------------------------------------
AtBglu32            --------LNRYSFPP-HF---------------------------------------------------------------------------------
AtBglu33            --------IHKQDFPA-DF---------------------------------------------------------------------------------
AtBglu34            --------FNRTGFPR-NF---------------------------------------------------------------------------------
AtBglu35            --------FNRSGFPK-NF---------------------------------------------------------------------------------
AtBglu36            --------QPKWISQK-NF---------------------------------------------------------------------------------
AtBglu37            --------FNKQDFES-DF---------------------------------------------------------------------------------
AtBglu38            --------FNSGNFEK-GF---------------------------------------------------------------------------------
AtBglu39            --------FNRKHFDD-DF---------------------------------------------------------------------------------
AtBglu4             --------FSRSDYPE-GF---------------------------------------------------------------------------------
AtBglu40            --------ISRGSFPK-GF---------------------------------------------------------------------------------
AtBglu41            --------ISRANFPD-GF---------------------------------------------------------------------------------
AtBglu42            --------THRSNFPS-TF---------------------------------------------------------------------------------
AtBglu43            --------LNRKSFPE-GF---------------------------------------------------------------------------------
AtBglu44            --------LSRQSFPK-GF---------------------------------------------------------------------------------
AtBglu45            --------VDSSPFPS-DF---------------------------------------------------------------------------------
AtBglu46            --------DDSSPFPS-DF---------------------------------------------------------------------------------
AtBglu47            --------EETFHFPK-NF---------------------------------------------------------------------------------
```

```
AtBglu5          ----------------------------------------------------------------------------------------------------
AtBglu7          --------FTRNDFPN-DF---------------------------------------------------------------------------------
AtBglu8          --------FTRNDFPE-DF---------------------------------------------------------------------------------
AtBglu9          --------FTRNSFPK-DF---------------------------------------------------------------------------------
BD1G10890        --------LSQASFPK-GF---------------------------------------------------------------------------------
BD1G10920        --------LSRASFPK-GF---------------------------------------------------------------------------------
BD1G10930        --------LSRASFPK-GF---------------------------------------------------------------------------------
BD1G10940        --------LSRAAFPK-GF---------------------------------------------------------------------------------
BD1G19270        --------LSRGAFPE-GF---------------------------------------------------------------------------------
BD1G33040        --------IRRADFPP-GF---------------------------------------------------------------------------------
BD1G42690        --------IKRSQFPP-EF---------------------------------------------------------------------------------
BD1G70170        --------LTRGSFPK-GF---------------------------------------------------------------------------------
BD2G09190        --------ISRDDFPA-GF---------------------------------------------------------------------------------
BD2G09200        --------ISRDDFPA-GF---------------------------------------------------------------------------------
BD2G27770        --------FTRADFPS-DF---------------------------------------------------------------------------------
BD2G37450        ---------------K-KF---------------------------------------------------------------------------------
BD2G57640        --------VTRADFPE-GF---------------------------------------------------------------------------------
BD2G59650        --------FTRTDFPP-DF---------------------------------------------------------------------------------
BD2G59660        --------YTRSDFPR-GF---------------------------------------------------------------------------------
BD3G00650        --------LTRGSFPK-GF---------------------------------------------------------------------------------
BD3G40000        --------FSRYSFPK-DF---------------------------------------------------------------------------------
BD3G40010        --------FNRSSFPE-GF---------------------------------------------------------------------------------
BD3G45610        --------FTRCDFAQ-DF---------------------------------------------------------------------------------
BD3G45630        --------FTRSDFAQ-DF---------------------------------------------------------------------------------
BD3G45640        --------FTRSDFAQ-DF---------------------------------------------------------------------------------
BD3G45650        --------FTRGDFAQ-DF---------------------------------------------------------------------------------
BD4G08040        --------LNRDSFPK-GF---------------------------------------------------------------------------------
BD4G09920        --------AAADSEDD-EF---------------------------------------------------------------------------------
BD4G34930        --------ITKDDFPP-GF---------------------------------------------------------------------------------
BD4G34940        --------LTRRDFPE-GF---------------------------------------------------------------------------------
BD4G34950        --------LTRHDFPD-GF---------------------------------------------------------------------------------
BD5G13260        --------ISRRSFPE-GF---------------------------------------------------------------------------------
BD5G13270        --------WSQRGNIK-WA---------------------------------------------------------------------------------
BD5G15530        --------VDRSQFPSSNF---------------------------------------------------------------------------------
BD5G15540        --------LGRDEFPP-GF---------------------------------------------------------------------------------
GRMZM2G012236    --------LRRSDFPP-SF---------------------------------------------------------------------------------
GRMZM2G015804    --------AHAGELPQ------------------------------------------------------------------------------------
GRMZM2G031660    --------VRRSDFPP-SF---------------------------------------------------------------------------------
GRMZM2G055699    --------FTRQDFPR-AF---------------------------------------------------------------------------------
GRMZM2G069024    --------FTRSEFPE-DF---------------------------------------------------------------------------------
GRMZM2G108133    --------VTRADFPA-GF---------------------------------------------------------------------------------
GRMZM2G110948    --------LSRRSFPK-GF---------------------------------------------------------------------------------
GRMZM2G112704    --------LNRQSFPP-GF---------------------------------------------------------------------------------
GRMZM2G128197    --------LSRDAFPK-GF---------------------------------------------------------------------------------
GRMZM2G148176    --------LSRRAFPD-GF---------------------------------------------------------------------------------
GRMZM2G163544    --------LRRADFPQ-GF---------------------------------------------------------------------------------
GRMZM2G177661    --------LTRGSFPK-GF---------------------------------------------------------------------------------
GRMZM2G376416    ----------------------------------------------------------------------------------------------------
```

```
GRMZM2G457040   --------VDRSEFPP-GF--------------------------------------------------------------------------------
Os10bglu34      --------LTRKSFPN-GF--------------------------------------------------------------------------------
Os11bglu36      --------DADDSEED-NF--------------------------------------------------------------------------------
Os12bglu38      --------LTRETFPA-GF--------------------------------------------------------------------------------
Os1bglu1        --------LSRRSFPA-GF--------------------------------------------------------------------------------
Os1bglu2        --------YTRSDFPR-DF--------------------------------------------------------------------------------
Os1bglu3        --------YTRNDFPA-DF--------------------------------------------------------------------------------
Os1bglu4        --------VTRGDFPD-GF--------------------------------------------------------------------------------
Os1bglu5        --------YTRGDFPE-DF--------------------------------------------------------------------------------
Os3bglu6        --------LTRGSFPE-GF--------------------------------------------------------------------------------
Os3bglu7        --------LSRAAFPK-RF--------------------------------------------------------------------------------
Os3bglu8        --------LSRAAFPK-GF--------------------------------------------------------------------------------
Os4bglu10       --------ISRRSFPK-GF--------------------------------------------------------------------------------
Os4bglu11       --------ISRRSFPK-GF--------------------------------------------------------------------------------
Os4bglu12       --------VSRRSFPK-GF--------------------------------------------------------------------------------
Os4bglu13       --------ISRRSFPE-GF--------------------------------------------------------------------------------
Os4bglu14       --------VDRSQFPP-DF--------------------------------------------------------------------------------
Os4bglu16       --------LRRDDFPP-GF--------------------------------------------------------------------------------
Os4bglu18       --------IHRSDFPA-SF--------------------------------------------------------------------------------
Os4bglu27       --------FNRFSFPE-DF--------------------------------------------------------------------------------
Os4bglu28       --------LNRHSFPE-GF--------------------------------------------------------------------------------
Os4bglu9        --------ISRRSFPK-GF--------------------------------------------------------------------------------
Os5bglu19       --------FTRDDFPD-GF--------------------------------------------------------------------------------
Os5bglu21       --------FTRDDFPH-DF--------------------------------------------------------------------------------
Os5bglu22       --------FTRQDFPG-EF--------------------------------------------------------------------------------
Os5bglu23       --------FTRQDFPG-GL--------------------------------------------------------------------------------
Os5gblu20       --------FRREDFPD-GF--------------------------------------------------------------------------------
Os6bglu24       --------IRRSQFPE-DF--------------------------------------------------------------------------------
Os6bglu25       --------ISRADFPP-GF--------------------------------------------------------------------------------
Os7bglu26       --------LSRRAFPE-GF--------------------------------------------------------------------------------
Os9bglu29       --------FNRYSFPK-DF--------------------------------------------------------------------------------
Os9bglu30       --------FTRYSFPK-DF--------------------------------------------------------------------------------
Os9bglu31       --------ITRADFPP-EF--------------------------------------------------------------------------------
Os9bglu32       --------LTRHDFPE-GF--------------------------------------------------------------------------------
Os9bglu33       --------LTRHDFPE-GF--------------------------------------------------------------------------------
SB01G010825     --------LSRDAFPK-GF--------------------------------------------------------------------------------
SB01G010830     --------LSRPAFPR-GF--------------------------------------------------------------------------------
SB01G013360     ---------------------------------------------------------------------------------------------------
SB01G043030     --------LTRGSFPK-GF--------------------------------------------------------------------------------
SB02G028400     --------FSRYSFPK-DF--------------------------------------------------------------------------------
SB02G029620     --------ITRSDFPA-GF--------------------------------------------------------------------------------
SB02G041550     --------LSRRAFPE-GF--------------------------------------------------------------------------------
SB03G037780     --------FTRSDFPE-DF--------------------------------------------------------------------------------
SB03G042690     --------VTRADFPD-GF--------------------------------------------------------------------------------
SB06G019830     ---------------------------------------------------------------------------------------------------
SB06G019840     --------ISRRSFPE-GF--------------------------------------------------------------------------------
SB06G019850     --------SSRRSFPE-GF--------------------------------------------------------------------------------
SB06G019860     --------ISRRSFPE-GF--------------------------------------------------------------------------------
```

```
SB06G019880     ----------------------------------------------------------------------------------------------------
SB06G022410     --------LRRSDFPP-SF---------------------------------------------------------------------------------
SB06G022420     ASAALCLPLLRAAFSR-SAAAANANAQPDALARPVRQGVQPLLRARASLLLARAAPPCVRRRLRRRGSTVRRPAPVGRGRWRGREGRWGAHAGGDGGVPL
SB06G022450     --------IDRNQFPP-DF---------------------------------------------------------------------------------
SB06G022460     --------IDRNQFPP-DF---------------------------------------------------------------------------------
SB06G022490     --------VDRGEFPP-GF---------------------------------------------------------------------------------
SB06G022500     --------VRRSDFPA-SF---------------------------------------------------------------------------------
SB06G022510     --------VRRSDFPA-SF---------------------------------------------------------------------------------
SB08G007586     --------PRRDWFPP-SF---------------------------------------------------------------------------------
SB08G007650     --------PRKDWFPP-SF---------------------------------------------------------------------------------
SB09G018160     --------FTVDDFPD-GF---------------------------------------------------------------------------------
SB10G012220     --------VSRSQFPK-GF---------------------------------------------------------------------------------
SB10G022300     --------ITRGDFPE-GF---------------------------------------------------------------------------------
SB10G027600     --------LRRADFPQ-GF---------------------------------------------------------------------------------
SB10G028060     --------LSRETFPP-GF---------------------------------------------------------------------------------
SbDhr1          --------PRRDWFPP-SF---------------------------------------------------------------------------------
SbDhr2          --------PKRDWFPP-SF---------------------------------------------------------------------------------
Zm-p60.1        --------PQRDWFPS-DF---------------------------------------------------------------------------------
ZmGLU2          --------PRRDWFPS-DF---------------------------------------------------------------------------------
ZmGLU3          --------PKRDWFPP-SF---------------------------------------------------------------------------------
ZmGLU4          --------SPQNSKP--RC---------------------------------------------------------------------------------
ZmGLU5          --------PKRDWFPS-DF---------------------------------------------------------------------------------
ZmGLU6          --------PKGDWFPS-DF---------------------------------------------------------------------------------
ZmGLU7          --------PRREWFPP-SF---------------------------------------------------------------------------------
ZmGLU8          --------PRREWFPP-SF---------------------------------------------------------------------------------
```

```
                           710       720       730       740       750       760       770       780       790       800
                    ....|....|....|....|....|....|....|....|....|....|....|....|....|....|....|....|....|....|....|....|
AC155376.2_FGT005   -VFG----------------------VATS--------A-------YQ----------------------------------------------------
AC211140.2_FGT007   -VFG----------------------TAAA--------A-------YQ----------------------------------------------------
AC217401.3_FGT001   -VFG----------------------TATS--------A-------YQ----------------------------------------------------
AC217687.3_FGT004   -VFG----------------------TATS--------A-------YQ----------------------------------------------------
AtBglu1             -VFG----------------------AGIS--------A-------YQ----------------------------------------------------
AtBglu10            -LFG----------------------AATS--------A-------YQ----------------------------------------------------
AtBglu11            -VFG----------------------SGTS--------A-------YQ----------------------------------------------------
AtBglu12            -IFG----------------------AATS--------A-------YQ----------------------------------------------------
AtBglu13            -IFG----------------------AATS--------A-------YQ----------------------------------------------------
AtBglu14            -IFG----------------------AATS--------A-------YQ----------------------------------------------------
AtBglu15            -IFG----------------------SATS--------A-------YQ----------------------------------------------------
AtBglu16            -VFG----------------------SATS--------A-------YQ----------------------------------------------------
AtBglu17            -RFG----------------------AASS--------A-------YQ----------------------------------------------------
AtBglu18            -IWG----------------------TATA--------A-------FQ----------------------------------------------------
AtBglu19            -MFG----------------------TATA--------A-------FQ----------------------------------------------------
AtBglu2             ----------------------------------------------------------------------------------------------------
AtBglu20            -IFG----------------------TATA--------A-------FQ----------------------------------------------------
AtBglu21            -LFG----------------------TATA--------A-------FQ----------------------------------------------------
AtBglu22            -VFG----------------------TATA--------A-------FQ----------------------------------------------------
AtBglu23            -LFG----------------------TATA--------A-------YQ----------------------------------------------------
AtBglu24            -LFG----------------------TATA--------A-------YQ----------------------------------------------------
AtBglu25            -LFG----------------------ATTS--------A-------FQ----------------------------------------------------
AtBglu26            -LFG----------------------TASS--------S-------YQ----------------------------------------------------
AtBglu27            -LFG----------------------TASS--------A-------YQ----------------------------------------------------
AtBglu28            -VFG----------------------TAAS--------A-------FQ----------------------------------------------------
AtBglu29            -VFG----------------------TAIS--------A-------FQ----------------------------------------------------
AtBglu3             -IFG----------------------SATS--------A-------YQ----------------------------------------------------
AtBglu30            -IFG----------------------TAAS--------A-------FQ----------------------------------------------------
AtBglu31            -DFG----------------------VASS--------A-------YQ----------------------------------------------------
AtBglu32            -DFG----------------------VASS--------A-------YQ----------------------------------------------------
AtBglu33            -IFG----------------------TSVS--------A-------YQ----------------------------------------------------
AtBglu34            -TFG----------------------AATS--------A-------YQ----------------------------------------------------
AtBglu35            -TFG----------------------AATS--------A-------YQ----------------------------------------------------
AtBglu36            -TFG----------------------AATS--------A-------YQ----------------------------------------------------
AtBglu37            -IFG----------------------VASS--------A-------YQ----------------------------------------------------
AtBglu38            -IFG----------------------VASS--------A-------YQ----------------------------------------------------
AtBglu39            -IFE----------------------G-------------------------------------------------------------------------
AtBglu4             -VFG----------------------AGTS--------A-------YQ----------------------------------------------------
AtBglu40            -VFG----------------------TASS--------A-------FQ----------------------------------------------------
AtBglu41            -VFG----------------------TASS--------A-------YQ----------------------------------------------------
AtBglu42            -TFG----------------------VATS--------A-------YQ----------------------------------------------------
AtBglu43            -LFG----------------------TATS--------A-------YQ----------------------------------------------------
AtBglu44            -VFG----------------------TATS--------A-------YQ----------------------------------------------------
AtBglu45            -LFG----------------------TASS--------A-------YQ----------------------------------------------------
AtBglu46            -LFG----------------------TASS--------A-------FQ----------------------------------------------------
AtBglu47            -LFG----------------------TASS--------A-------YQ----------------------------------------------------
```

```
AtBglu5           --------------------------------------L-------CK----------------------------------------------------
AtBglu7           -LFG----------------------AATS--------A-------YQ----------------------------------------------------
AtBglu8           -LFG----------------------AGTS--------A-------YQ----------------------------------------------------
AtBglu9           -LFG----------------------AATS--------A-------YQ----------------------------------------------------
BD1G10890         -VFG----------------------TATS--------A-------YQ----------------------------------------------------
BD1G10920         -VFG----------------------TATS--------A-------YQ----------------------------------------------------
BD1G10930         -VFG----------------------TATS--------A-------YQ----------------------------------------------------
BD1G10940         -VFG----------------------TAAS--------A-------FQ----------------------------------------------------
BD1G19270         -VFG----------------------TAAS--------A-------YQ----------------------------------------------------
BD1G33040         -TFG----------------------TASS--------A-------YQ----------------------------------------------------
BD1G42690         -MFG----------------------TASS--------A-------YQ----------------------------------------------------
BD1G70170         -VFG----------------------TASA--------A-------YQ----------------------------------------------------
BD2G09190         -VFG----------------------AGTS--------A-------YQ----------------------------------------------------
BD2G09200         -VFG----------------------AGTS--------A-------YQ----------------------------------------------------
BD2G27770         -VFG----------------------AGTS--------A-------YQ----------------------------------------------------
BD2G37450         -WRS----------------------ASWS--------A-------SR----------------------------------------------------
BD2G57640         -IFG----------------------VATS--------A-------YQ----------------------------------------------------
BD2G59650         -VFG----------------------AATS--------S-------YQ----------------------------------------------------
BD2G59660         -VFG----------------------AATS--------A-------YQ----------------------------------------------------
BD3G00650         -VFG----------------------TAAS--------A-------YQ----------------------------------------------------
BD3G40000         -IFG----------------------TGSS--------A-------IQ----------------------------------------------------
BD3G40010         -IFG----------------------TGTS--------A-------YQ----------------------------------------------------
BD3G45610         -VFG----------------------AGTS--------A-------YQ----------------------------------------------------
BD3G45630         -VFG----------------------AGTS--------A-------YQ----------------------------------------------------
BD3G45640         -VFG----------------------AGTS--------A-------YQ----------------------------------------------------
BD3G45650         -VFG----------------------AGTS--------A-------YQ----------------------------------------------------
BD4G08040         -VFG----------------------TASS--------A-------YQ----------------------------------------------------
BD4G09920         -FFG----------------------LATA--------P-------AH----------------------------------------------------
BD4G34930         -VFG----------------------TGSS--------A-------YQ----------------------------------------------------
BD4G34940         -VFG----------------------AGSS--------A-------YQ----------------------------------------------------
BD4G34950         -IFG----------------------AGTS--------A-------YQ----------------------------------------------------
BD5G13260         -VFG----------------------TASS--------S-------YQ----------------------------------------------------
BD5G13270         -KLG----------------------DGNS--------K-------FF----------------------------------------------------
BD5G15530         -LFG----------------------TSTS--------A-------YQGKYFLCWITKGLLLVCARRGELGVWSGRRSSLHAGRRVHSSPGKCELVVASI
BD5G15540         -LFG----------------------AATS--------A-------YQ----------------------------------------------------
GRMZM2G012236     -LFG----------------------AGSS--------S-------YQ----------------------------------------------------
GRMZM2G015804     ----------------------------------------------------------------------------------------------------
GRMZM2G031660     -LFG----------------------TATS--------S-------YQ----------------------------------------------------
GRMZM2G055699     -VFG----------------------AGTS--------A-------YQ----------------------------------------------------
GRMZM2G069024     -VFG----------------------SATS--------A-------YQ----------------------------------------------------
GRMZM2G108133     -VFG----------------------VGSS--------A-------YQ----------------------------------------------------
GRMZM2G110948     -VFG----------------------TAAS--------A-------YQ----------------------------------------------------
GRMZM2G112704     -VFG----------------------TASS--------A-------YQ----------------------------------------------------
GRMZM2G128197     -VFG----------------------TATS--------A-------YQ----------------------------------------------------
GRMZM2G148176     -VFG----------------------TAAS--------A-------YQ----------------------------------------------------
GRMZM2G163544     -VFG----------------------TASS--------A-------YQ----------------------------------------------------
GRMZM2G177661     -VFG----------------------TASS--------A-------YQ----------------------------------------------------
GRMZM2G376416     ----------------------------------------------------------------------------------------------------
```

```
GRMZM2G457040   -LFG----------------------AATS--------A-------YQ----------------------------------------------------
Os10bglu34      -VFG----------------------TASS--------A-------YQ----------------------------------------------------
Os11bglu36      -FFG----------------------LATA--------P-------AH----------------------------------------------------
Os12bglu38      -VFG----------------------TASS--------A-------YQ----------------------------------------------------
Os1bglu1        -VFG----------------------TAAS--------A-------YQ----------------------------------------------------
Os1bglu2        -VFG----------------------AATS--------A-------YQ----------------------------------------------------
Os1bglu3        -VFG----------------------AATS--------A-------YQ----------------------------------------------------
Os1bglu4        -VFG----------------------VATS--------A-------YQ----------------------------------------------------
Os1bglu5        -VFG----------------------SATS--------S-------YQ----------------------------------------------------
Os3bglu6        -VFG----------------------TASA--------A-------YQ----------------------------------------------------
Os3bglu7        -VFG----------------------TATS--------A-------YQ----------------------------------------------------
Os3bglu8        -VFG----------------------TATS--------A-------FQ----------------------------------------------------
Os4bglu10       -IFG----------------------TSSS--------S-------YQ----------------------------------------------------
Os4bglu11       -IFG----------------------TSSS--------S-------YQ----------------------------------------------------
Os4bglu12       -IFG----------------------TASS--------S-------YQ----------------------------------------------------
Os4bglu13       -IFG----------------------TASS--------S-------YQ----------------------------------------------------
Os4bglu14       -LFG----------------------TSSS--------A-------YQ----------------------------------------------------
Os4bglu16       -LFG----------------------AATS--------A-------YQ----------------------------------------------------
Os4bglu18       -LFG----------------------TATS--------S-------YQ----------------------------------------------------
Os4bglu27       -IFG----------------------TGSA--------A-------YQ----------------------------------------------------
Os4bglu28       -LFG----------------------TGTS--------A-------YQ----------------------------------------------------
Os4bglu9        -IFG----------------------TSSA--------S-------YQ----------------------------------------------------
Os5bglu19       -TFG----------------------AGTA--------A-------FQ----------------------------------------------------
Os5bglu21       -AFG----------------------AGTS--------A-------YQ----------------------------------------------------
Os5bglu22       -VFG----------------------AGTS--------A-------YQ----------------------------------------------------
Os5bglu23       -RRRHICLPGFRDADSITFPRDIESLTCGTHMCLDPRGSHTMRGLPVK----------------------------------------------------
Os5gblu20       -AFG----------------------AGTA--------A-------YQ----------------------------------------------------
Os6bglu24       -FFG----------------------TASS--------A-------YQ----------------------------------------------------
Os6bglu25       -IFG----------------------TASS--------A-------YQ----------------------------------------------------
Os7bglu26       -VFG----------------------TAAS--------A-------YQ----------------------------------------------------
Os9bglu29       -IFG----------------------TGSA--------A-------YQ----------------------------------------------------
Os9bglu30       -IFG----------------------TGSA--------A-------YQ----------------------------------------------------
Os9bglu31       -IFG----------------------AGSS--------A-------YQ----------------------------------------------------
Os9bglu32       -VFG----------------------AGTS--------A-------FQ----------------------------------------------------
Os9bglu33       -VFG----------------------AGSS--------A-------FQ----------------------------------------------------
SB01G010825     -IFG----------------------TATS--------A-------FQ----------------------------------------------------
SB01G010830     -VFG----------------------TATS--------A-------YQ----------------------------------------------------
SB01G013360     ----------------------------------------------------------------------------------------------------
SB01G043030     -VFG----------------------TAAA--------A-------YQ----------------------------------------------------
SB02G028400     -VFG----------------------TGSA--------A-------YQ----------------------------------------------------
SB02G029620     -VFG----------------------AGSS--------A-------YQ----------------------------------------------------
SB02G041550     -VFG----------------------TAAS--------A-------YQ----------------------------------------------------
SB03G037780     -VFG----------------------SATS--------A-------YQ----------------------------------------------------
SB03G042690     -VFG----------------------VATS--------A-------YQ----------------------------------------------------
SB06G019830     ----------------------------------------------------------------------------------------------------
SB06G019840     -IFG----------------------TASS--------A-------YQ----------------------------------------------------
SB06G019850     -IFG----------------------TASA--------A-------YQ----------------------------------------------------
SB06G019860     -IFG----------------------TASS--------A-------YQ----------------------------------------------------
```

```
SB06G019880    ----------------------------------------------------------------------------------------------------
SB06G022410    -LFG----------------------AGTS--------S-------YQ----------------------------------------------------
SB06G022420    DVLG----------------------HGVE--------H-------FG----------------------------------------------------
SB06G022450    -LFG----------------------TSTS--------A-------YQ----------------------------------------------------
SB06G022460    -LFG----------------------TSTS--------A-------YQ----------------------------------------------------
SB06G022490    -LFG----------------------AATS--------A-------YQ----------------------------------------------------
SB06G022500    -LFG----------------------TATS--------S-------YQ----------------------------------------------------
SB06G022510    -LFG----------------------TATS--------S-------YQ----------------------------------------------------
SB08G007586    -LFG----------------------AATA--------S-------YQ----------------------------------------------------
SB08G007650    -LVS----------------------AATS--------A-------YH----------------------------------------------------
SB09G018160    -AFG----------------------AGTA--------A-------FQ----------------------------------------------------
SB10G012220    -LFG----------------------TASS--------A-------YQ----------------------------------------------------
SB10G022300    -VFG----------------------AGAS--------A-------YQ----------------------------------------------------
SB10G027600    -VIG----------------------TASS--------A-------YQ----------------------------------------------------
SB10G028060    -VFG----------------------TASS--------A-------YQ----------------------------------------------------
SbDhr1         -LFG----------------------AATS--------A-------YQ----------------------------------------------------
SbDhr2         -TFG----------------------AATS--------A-------FQ----------------------------------------------------
Zm-p60.1       -TFG----------------------AATS--------A-------YQ----------------------------------------------------
ZmGLU2         -IFG----------------------AATS--------A-------YQ----------------------------------------------------
ZmGLU3         -IFG----------------------AATA--------A-------YQ----------------------------------------------------
ZmGLU4         -NLS----------------------FRPR--------A-------ER----------------------------------------------------
ZmGLU5         -IFG----------------------AATS--------A-------YQ----------------------------------------------------
ZmGLU6         -IVG----------------------AATS--------A-------YQ----------------------------------------------------
ZmGLU7         -IFG----------------------AATS--------A-------YQ----------------------------------------------------
ZmGLU8         -IFG----------------------AATS--------A-------YQ----------------------------------------------------
```

```
                            810       820       830       840       850       860       870       880       890       900
                    ....|....|....|....|....|....|....|....|....|....|....|....|....|....|....|....|....|....|....|....|
AC155376.2_FGT005   -----------------------------------------------IEGARRE---------------G----GKGDNI-----WDV------------
AC211140.2_FGT007   -----------------------------------------------YEGAVKT---------------D----GRGQTI-----WDT------------
AC217401.3_FGT001   -----------------------------------------------VEGAAST---------------N----GRGPST-----WDA------------
AC217687.3_FGT004   -----------------------------------------------VEGAAST---------------N----GRGPSI-----WDS------------
AtBglu1             -----------------------------------------------WEGAVDE---------------D----GRKPSV-----WDT------------
AtBglu10            -----------------------------------------------WEGAVAE---------------D----GRTPSV-----WDT------------
AtBglu11            -----------------------------------------------VEGAADE---------------D----GRTPSI-----WDV------------
AtBglu12            -----------------------------------------------VEGAAHE---------------D----GRGPSI-----WDT------------
AtBglu13            -----------------------------------------------VEGAAHE---------------D----GRGPSI-----WDT------------
AtBglu14            -----------------------------------------------VEGAAQE---------------D----GRGPSI-----WDT------------
AtBglu15            -----------------------------------------------VEGGAHE---------------D----GRGPSI-----WDT------------
AtBglu16            -----------------------------------------------CEGAAHE---------------D----GRGPSI-----WDS------------
AtBglu17            -----------------------------------------------SEGAANV---------------D----GREPSI-----WDT------------
AtBglu18            -----------------------------------------------VEGAVNE---------------G----CRGPSM-----WDT------------
AtBglu19            -----------------------------------------------VEGAVNE---------------G----CRGPSL-----WDI------------
AtBglu2             -----------------------------------------------------E----------------------------------------------
AtBglu20            -----------------------------------------------VEGAVNE---------------G----CRGPSM-----WDV------------
AtBglu21            -----------------------------------------------VEGAINE---------------T----CRGPAL-----WDI------------
AtBglu22            -----------------------------------------------VEGAINE---------------T----CRGPAL-----WDI------------
AtBglu23            -----------------------------------------------VEGAINE---------------T----CRGPAL-----WDI------------
AtBglu24            -----------------------------------------------VEGAVNE---------------T----CRGPSV-----WDI------------
AtBglu25            -----------------------------------------------HEGAAEE---------------G----GRGSSI-----WDS------------
AtBglu26            -----------------------------------------------YEGAVNE---------------G----ARGQSV-----WDH------------
AtBglu27            -----------------------------------------------YEGARNE---------------A----PRGESV-----WDT------------
AtBglu28            -----------------------------------------------YEGATSE---------------G----GKSPSI-----WDY------------
AtBglu29            -----------------------------------------------SEGATSE---------------G----GKSPTI-----WDY------------
AtBglu3             -----------------------------------------------WEGAFDE---------------D----GRKPSV-----WDT------------
AtBglu30            -----------------------------------------------YEGATSE---------------G----GKSPTI-----WDH------------
AtBglu31            -----------------------------------------------YEGAVEE---------------G----GRSLSI-----WDN------------
AtBglu32            -----------------------------------------------YEGAVEE---------------G----GRSPSI-----WDN------------
AtBglu33            -----------------------------------------------VEGAKKG---------------S----GRGLTS-----WDE------------
AtBglu34            -----------------------------------------------IEGAA----------------------HRALNG-----WDY------------
AtBglu35            -----------------------------------------------IEGAA----------------------HRALNG-----WDY------------
AtBglu36            -----------------------------------------------VEGAA----------------------HRALNG-----WDY------------
AtBglu37            -----------------------------------------------IEGGR----------------------GRGLNV-----WDG------------
AtBglu38            -----------------------------------------------VEGGR----------------------GRGLNV-----WDS------------
AtBglu39            --------------------------------------------------GK----------------------GRGLNV-----WDG------------
AtBglu4             -----------------------------------------------WEGAAAE---------------D----GRKPSL-----WDT------------
AtBglu40            -----------------------------------------------HEGAVKA---------------E----GRGPTI-----WDT------------
AtBglu41            -----------------------------------------------FEGAVKE---------------G----NKGESI-----WDT------------
AtBglu42            -----------------------------------------------IEGGWNE---------------G----KKGPSI-----WDK------------
AtBglu43            -----------------------------------------------VEGETHQ---------------D----GRGPSI-----WDA------------
AtBglu44            -----------------------------------------------VEGETHQ---------------D----GRGPSI-----WDA------------
AtBglu45            -----------------------------------------------YEGAFLT---------------D----GKSLNN-----WDV------------
AtBglu46            -----------------------------------------------YEGAFLT---------------D----GKGLNN-----WDV------------
AtBglu47            -----------------------------------------------YEGAYLT---------------D----GKTLSN-----WDV------------
```

```
AtBglu5          -----------------------------------------------WEGAAAE---------------D----GRKPSV-----WDT------------
AtBglu7          -----------------------------------------------WEGAFDE---------------D----GKSPSV-----WDT------------
AtBglu8          -----------------------------------------------WEGAANE---------------D----GRTPSV-----WDT------------
AtBglu9          -----------------------------------------------WEGAVAE---------------D----GRTPSV-----WDT------------
BD1G10890        -----------------------------------------------VEGMAFS---------------D----GRGPSV-----WDA------------
BD1G10920        -----------------------------------------------VEGMAFS---------------G----GRGPSV-----WDA------------
BD1G10930        -----------------------------------------------VEGMAAS---------------G----GRGPSI-----WDA------------
BD1G10940        -----------------------------------------------VEGMAAS---------------G----GRGPSI-----WDP------------
BD1G19270        -----------------------------------------------VEGMAKR---------------G----GRGPSI-----WDA------------
BD1G33040        -----------------------------------------------YEGAVNE---------------G----QRGPTI-----WDT------------
BD1G42690        -----------------------------------------------YEGAVRE---------------G----GRGPSI-----WDT------------
BD1G70170        -----------------------------------------------YEGAVKA---------------D----GRGQTI-----WDT------------
BD2G09190        -----------------------------------------------VSSCRDY------YYDDECRLAA----GSGASDIDKRRFDD------------
BD2G09200        -----------------------------------------------WEGAAAE---------------D----GRSPSV-----WDA------------
BD2G27770        -----------------------------------------------YEGATDE---------------D----GRSPSI-----WDT------------
BD2G37450        -----------------------------------------------VGEAAK-------------------------EG--------------------
BD2G57640        -----------------------------------------------IEGARKE---------------G----GKGDSI-----WDV------------
BD2G59650        -----------------------------------------------YEGAVDE---------------D----GRSPGI-----WDT------------
BD2G59660        -----------------------------------------------YEGAVAE---------------D----GRSPSI-----WDT------------
BD3G00650        -----------------------------------------------YEGAVKE---------------D----GRGPAI-----WDK------------
BD3G40000        -----------------------------------------------YEGAFE----------------------RGKTT-----WDT------------
BD3G40010        -----------------------------------------------YEGAVDE---------------------RGRNI-----WDT------------
BD3G45610        -----------------------------------------------YEGAVAE---------------D----GRSPSD-----WDT------------
BD3G45630        -----------------------------------------------YEGAVAE---------------D----GRSPSF-----WDT------------
BD3G45640        -----------------------------------------------YEGAVAE---------------D----GRSPSF-----WDT------------
BD3G45650        -----------------------------------------------YEGAVAE---------------D----GRSPSF-----WDT------------
BD4G08040        -----------------------------------------------VEGNALK---------------Y----GRGPCI-----WDT------------
BD4G09920        -----------------------------------------------VEDRLE-------------------------DA-----WLQ------------
BD4G34930        -----------------------------------------------IEGAVAE---------------D----GRKPSI-----WDT------------
BD4G34940        -----------------------------------------------VEGAASE---------------D----RRKPSI-----WDT------------
BD4G34950        -----------------------------------------------VEGAAAE---------------D----GRKPSI-----WDT------------
BD5G13260        -----------------------------------------------YEGGAME---------------G----GRGPSI-----WDN------------
BD5G13270        -----------------------------------------------HTLVTIQNRRNHIA--------S----LSGPD------GTC------------
BD5G15530        ETEGGTQVALITVPNLMLGLRRYPSGFNNSSKSQAVLEKSLYYVDLWIEGGYLE---------------G----NKGTSN-----WDV------------
BD5G15540        -----------------------------------------------IEGAYLE---------------D----NKSLNN-----WDV------------
GRMZM2G012236    -----------------------------------------------IEGAYLE---------------D----NKGLSN-----WDV------------
GRMZM2G015804    ----------------------------------------------------------------------------GLRL-----RHR------------
GRMZM2G031660    -----------------------------------------------IEGAYLE---------------G----NKSLSN-----WDV------------
GRMZM2G055699    -----------------------------------------------YEGATDE---------------D----GRSPSI-----WDN------------
GRMZM2G069024    -----------------------------------------------YEGAVGE---------------D----GRSPSI-----WDT------------
GRMZM2G108133    -----------------------------------------------VEGAVAE---------------D----GRKPSI-----WDT------------
GRMZM2G110948    -----------------------------------------------VEGMAHK---------------D----GRGPSI-----WDA------------
GRMZM2G112704    -----------------------------------------------VEGNTHR---------------Y----GRGPCI-----WDT------------
GRMZM2G128197    -----------------------------------------------VEGAATS---------------G----GRGPCI-----WDP------------
GRMZM2G148176    -----------------------------------------------VEGMAKH---------------G----GRGPSI-----WDA------------
GRMZM2G163544    -----------------------------------------------YEGAVNE---------------G----QRGPTI-----WDT------------
GRMZM2G177661    -----------------------------------------------YEGAVKE---------------D----GRGKTI-----WDK------------
GRMZM2G376416    ----------------------------------------------------------------------------------------------------
```

```
GRMZM2G457040   -----------------------------------------------IEGAYLE---------------D----GKGLCN-----WDV------------
Os10bglu34      -----------------------------------------------YEGAVKE---------------D----GRGPTI-----WDK------------
Os11bglu36      -----------------------------------------------VEDRLE-------------------------DA-----WLQ------------
Os12bglu38      -----------------------------------------------VEGNALQ---------------Y----GRGPCI-----WDT------------
Os1bglu1        -----------------------------------------------VEGMALK---------------D----GRGPSI-----WDA------------
Os1bglu2        -----------------------------------------------YDGAAAE---------------D----GRSPTI-----WDT------------
Os1bglu3        -----------------------------------------------YEGAAAE---------------D----GRGASI-----WDT------------
Os1bglu4        -----------------------------------------------IEGARRE---------------G----GKGDNI-----WDV------------
Os1bglu5        -----------------------------------------------YEGGFDE---------------D----GRSPSN-----WDI------------
Os3bglu6        -----------------------------------------------YEGAVKE---------------D----GRGQTI-----WDT------------
Os3bglu7        -----------------------------------------------VEGMAAS---------------G----GRGPSI-----WDA------------
Os3bglu8        -----------------------------------------------VEGMAAS---------------G----GRGPSI-----WDP------------
Os4bglu10       -----------------------------------------------FEGAAAK---------------G----GRGPSI-----WDT------------
Os4bglu11       -----------------------------------------------FEGGAVL---------------G----GRGPSI-----WDT------------
Os4bglu12       -----------------------------------------------YEGGAAE---------------G----GRGPSI-----WDT------------
Os4bglu13       -----------------------------------------------YEGGARE---------------G----GRGPSI-----WDT------------
Os4bglu14       -----------------------------------------------VEGGYLE---------------G----NKGLSN-----WDV------------
Os4bglu16       -----------------------------------------------IEGAYLD---------------D----NKGLNN-----WDV------------
Os4bglu18       -----------------------------------------------IEGAYLE---------------G----NKSLSN-----WDV------------
Os4bglu27       -----------------------------------------------YEGAVNE---------------G----GRGPSI-----WDT------------
Os4bglu28       -----------------------------------------------YEGAVDK--------------------RGQNI-----WDT------------
Os4bglu9        -----------------------------------------------CEGGAAE---------------G----GRGPSI-----WDT------------
Os5bglu19       -----------------------------------------------YEGAAAE---------------D----GRTPSI-----WDT------------
Os5bglu21       -----------------------------------------------YEGGAAE---------------D----GRTPSI-----WDT------------
Os5bglu22       -----------------------------------------------YEGATDE---------------D----GRSPSI-----WDT------------
Os5bglu23       -----------------------------------------------TEGPQAS---------------GTLLLIQVCLL-----WDGRVSYMIKKIHIC
Os5gblu20       -----------------------------------------------YEGAAAE---------------D----GRTPSI-----WDT------------
Os6bglu24       -----------------------------------------------YEGAVRE---------------G----GRGPSI-----WDT------------
Os6bglu25       -----------------------------------------------YEGAVNE---------------G----QRGPTI-----WDT------------
Os7bglu26       -----------------------------------------------VEGMAKQ---------------G----GRGPSI-----WDA------------
Os9bglu29       ----------------------------------------------------------------------------------------------------
Os9bglu30       -----------------------------------------------YEGAYKE---------------G----GKGPSV-----WDN------------
Os9bglu31       -----------------------------------------------VEGAFAE---------------D----GRKPSI-----WDT------------
Os9bglu32       -----------------------------------------------VEGAAAE---------------D----GRKPSI-----WDT------------
Os9bglu33       -----------------------------------------------VEGAAAE---------------D----GRKPSI-----WDT------------
SB01G010825     -----------------------------------------------VEGAATS---------------G----GRGPCI-----WDP------------
SB01G010830     -----------------------------------------------VEGAAST---------------N----GRGPST-----WDP------------
SB01G013360     ----------------------------------------------------------------------------------------------------
SB01G043030     -----------------------------------------------YEGAVTT---------------D----GRGRTI-----WDT------------
SB02G028400     -----------------------------------------------YEGAYNE---------------G----GKGPSI-----WDK------------
SB02G029620     -----------------------------------------------VEGAFAE---------------D----GRNASI-----WDT------------
SB02G041550     -----------------------------------------------VEGMAKH---------------G----GRGPSI-----WDA------------
SB03G037780     -----------------------------------------------YEGAVAE---------------D----GRSPSI-----WDT------------
SB03G042690     -----------------------------------------------IEGARRE---------------G----GKGDSI-----WDV------------
SB06G019830     ---------------------------------------------------------------------------EI-----FDK------------
SB06G019840     -----------------------------------------------YEGGAME---------------G----GRGPSI-----WDT------------
SB06G019850     -----------------------------------------------AVHYA-----------------N----GSSNNV--------------------
SB06G019860     -----------------------------------------------YEGGAME---------------G----GRGPSI-----WDT------------
```

```
SB06G019880   ----------------------------------------------------ME---------------G----GRGPSI-----WDT------------
SB06G022410   -----------------------------------------------IEGAYRE---------------D----NKGLSN-----WDV------------
SB06G022420   -----------------------------------------------LEG----------------------------GR-----IDY------------
SB06G022450   -----------------------------------------------IEGGYLE---------------G----NKGLSN-----WDI------------
SB06G022460   -----------------------------------------------IEGGYLE---------------G----NKGLSN-----WDI------------
SB06G022490   -----------------------------------------------IEGAYLE---------------D----GKGLCN-----WDV------------
SB06G022500   -----------------------------------------------IEGAYLE---------------G----NKSLSN-----WDV------------
SB06G022510   -----------------------------------------------IEGAYLE---------------G----NKSLSN-----WDV------------
SB08G007586   -----------------------------------------------IEGAWNE---------------D----GKGPST-----WDH------------
SB08G007650   -----------------------------------------------IEGAWNE---------------D----GKGPST-----WDH------------
SB09G018160   -----------------------------------------------YEGAVDE---------------D----GKSPSI-----WNT------------
SB10G012220   -----------------------------------------------YEGAVRE---------------G----GRGPSI-----WDT------------
SB10G022300   -----------------------------------------------VEGAWAE---------------D----GKKPSI-----WDT------------
SB10G027600   -----------------------------------------------YEGAVNE---------------G----RRGPTI-----WDT------------
SB10G028060   -----------------------------------------------VEGNTRK---------------F----GRGPSI-----WDT------------
SbDhr1        -----------------------------------------------IEGAWNE---------------D----GKGPST-----WDH------------
SbDhr2        -----------------------------------------------IEGGWNE---------------D----GKGPST-----WDH------------
Zm-p60.1      -----------------------------------------------IEGAWNE---------------D----GKGESN-----WDH------------
ZmGLU2        -----------------------------------------------IEGAWNE---------------D----GKGESN-----WDH------------
ZmGLU3        -----------------------------------------------IEGAWNE---------------D----GKGPSN-----WDH------------
ZmGLU4        -----------------------------------------------IEGGWNE---------------D----GKGPSS-----WDY------------
ZmGLU5        -----------------------------------------------IEGGWNE---------------D----GKKPST-----WDH------------
ZmGLU6        -----------------------------------------------IEGGWNE---------------G----GKGPST-----WDH------------
ZmGLU7        -----------------------------------------------IEGAWNE---------------G----GKGPST-----WDD------------
ZmGLU8        -----------------------------------------------IEGAWNE---------------G----GKGPST-----WDD------------
```

```
                           910       920       930       940       950       960       970       980       990      1000
                   ....|....|....|....|....|....|....|....|....|....|....|....|....|....|....|....|....|....|....|....|
AC155376.2_FGT005  --------F--TD---------------------------------------------------------------------------------------
AC211140.2_FGT007  --------F--AH---------------------------------------------------------------------------------------
AC217401.3_FGT001  --------F--VH---------------------------------------------------------------------------------------
AC217687.3_FGT004  --------F--AH---------------------------------------------------------------------------------------
AtBglu1            --------F--LH---------------------------------------------------------------------------------------
AtBglu10           --------F--SH---------------------------------------------------------------------------------------
AtBglu11           --------F--AH---------------------------------------------------------------------------------------
AtBglu12           --------F--SE---------------------------------------------------------------------------------------
AtBglu13           --------F--SE---------------------------------------------------------------------------------------
AtBglu14           --------F--SE---------------------------------------------------------------------------------------
AtBglu15           --------F--SE---------------------------------------------------------------------------------------
AtBglu16           --------F--SE---------------------------------------------------------------------------------------
AtBglu17           --------F--TK---------------------------------------------------------------------------------------
AtBglu18           --------F--TK---------------------------------------------------------------------------------------
AtBglu19           --------Y--TK---------------------------------------------------------------------------------------
AtBglu2            ----------------------------------------------------------------------------------------------------
AtBglu20           --------Y--TK---------------------------------------------------------------------------------------
AtBglu21           --------Y--CR---------------------------------------------------------------------------------------
AtBglu22           --------F--CK---------------------------------------------------------------------------------------
AtBglu23           --------Y--CR---------------------------------------------------------------------------------------
AtBglu24           --------Y--CK---------------------------------------------------------------------------------------
AtBglu25           --------F--TL---------------------------------------------------------------------------------------
AtBglu26           --------F--SN---------------------------------------------------------------------------------------
AtBglu27           --------F--VR---------------------------------------------------------------------------------------
AtBglu28           --------F--SH---------------------------------------------------------------------------------------
AtBglu29           --------F--SH---------------------------------------------------------------------------------------
AtBglu3            --------F--LH---------------------------------------------------------------------------------------
AtBglu30           --------F--SL---------------------------------------------------------------------------------------
AtBglu31           --------F--TH---------------------------------------------------------------------------------------
AtBglu32           --------F--TH---------------------------------------------------------------------------------------
AtBglu33           --------F--TH---------------------------------------------------------------------------------------
AtBglu34           --------F--TH---------------------------------------------------------------------------------------
AtBglu35           --------F--TH---------------------------------------------------------------------------------------
AtBglu36           --------F--TH---------------------------------------------------------------------------------------
AtBglu37           --------F--TH---------------------------------------------------------------------------------------
AtBglu38           --------F--TH---------------------------------------------------------------------------------------
AtBglu39           --------F--TH---------------------------------------------------------------------------------------
AtBglu4            --------L--CH---------------------------------------------------------------------------------------
AtBglu40           --------F--SH---------------------------------------------------------------------------------------
AtBglu41           --------F--TK---------------------------------------------------------------------------------------
AtBglu42           --------F--TH---------------------------------------------------------------------------------------
AtBglu43           --------F--VK---------------------------------------------------------------------------------------
AtBglu44           --------F--VK---------------------------------------------------------------------------------------
AtBglu45           --------F--TH---------------------------------------------------------------------------------------
AtBglu46           --------F--AH---------------------------------------------------------------------------------------
AtBglu47           --------F--TN---------------------------------------------------------------------------------------
```

```
AtBglu5        --------L--CY---------------------------------------------------------------------------------------
AtBglu7        --------T--SH---------------------------------------------------------------------------------------
AtBglu8        --------T--SH---------------------------------------------------------------------------------------
AtBglu9        --------F--SN---------------------------------------------------------------------------------------
BD1G10890      --------F--AH---------------------------------------------------------------------------------------
BD1G10920      --------F--AH---------------------------------------------------------------------------------------
BD1G10930      --------F--SH---------------------------------------------------------------------------------------
BD1G10940      --------F--VH---------------------------------------------------------------------------------------
BD1G19270      --------F--IE---------------------------------------------------------------------------------------
BD1G33040      --------L--TR---------------------------------------------------------------------------------------
BD1G42690      --------F--TH---------------------------------------------------------------------------------------
BD1G70170      --------F--AH---------------------------------------------------------------------------------------
BD2G09190      --------F--SGKARRRRTAGPPACGTPTLAPTLTALHLTSPHRRQISSPPFPSHAPQPRHEVCRADASCEPPPSAAILRATLAVAALHSAAAFEHNRR
BD2G09200      --------F--AR---------------------------------------------------------------------------------------
BD2G27770      --------F--TH---------------------------------------------------------------------------------------
BD2G37450      ----------------------------------------------------------------------------------------------------
BD2G57640      --------F--AD---------------------------------------------------------------------------------------
BD2G59650      --------F--TH---------------------------------------------------------------------------------------
BD2G59660      --------F--TH---------------------------------------------------------------------------------------
BD3G00650      --------F--AH---------------------------------------------------------------------------------------
BD3G40000      --------F--SH---------------------------------------------------------------------------------------
BD3G40010      --------F--SH---------------------------------------------------------------------------------------
BD3G45610      --------F--TH---------------------------------------------------------------------------------------
BD3G45630      --------F--TH---------------------------------------------------------------------------------------
BD3G45640      --------F--TH---------------------------------------------------------------------------------------
BD3G45650      --------F--TH---------------------------------------------------------------------------------------
BD4G08040      --------F--LK---------------------------------------------------------------------------------------
BD4G09920      --------F--AT---------------------------------------------------------------------------------------
BD4G34930      --------F--TH---------------------------------------------------------------------------------------
BD4G34940      --------W--SH---------------------------------------------------------------------------------------
BD4G34950      --------F--TH---------------------------------------------------------------------------------------
BD5G13260      --------F--TH---------------------------------------------------------------------------------------
BD5G13270      --------F--SE---------------------------------------------------------------------------------------
BD5G15530      --------Y--TH---------------------------------------------------------------------------------------
BD5G15540      --------F--TH---------------------------------------------------------------------------------------
GRMZM2G012236  --------F--TH---------------------------------------------------------------------------------------
GRMZM2G015804  --------R--RR---------------------------------------------------------------------------------------
GRMZM2G031660  --------F--SH---------------------------------------------------------------------------------------
GRMZM2G055699  --------F--TH---------------------------------------------------------------------------------------
GRMZM2G069024  --------F--TH---------------------------------------------------------------------------------------
GRMZM2G108133  --------F--TH---------------------------------------------------------------------------------------
GRMZM2G110948  --------F--IK---------------------------------------------------------------------------------------
GRMZM2G112704  --------F--LK---------------------------------------------------------------------------------------
GRMZM2G128197  --------F--VH---------------------------------------------------------------------------------------
GRMZM2G148176  --------F--IE---------------------------------------------------------------------------------------
GRMZM2G163544  --------L--TR---------------------------------------------------------------------------------------
GRMZM2G177661  --------F--AH---------------------------------------------------------------------------------------
GRMZM2G376416  ----------------------------------------------------------------------------------------------------
```

```
GRMZM2G457040    --------F--TH---------------------------------------------------------------------------------------
Os10bglu34       --------F--AH---------------------------------------------------------------------------------------
Os11bglu36       --------F--AT---------------------------------------------------------------------------------------
Os12bglu38       --------F--LM---------------------------------------------------------------------------------------
Os1bglu1         --------F--VK---------------------------------------------------------------------------------------
Os1bglu2         --------F--AH---------------------------------------------------------------------------------------
Os1bglu3         --------F--TH---------------------------------------------------------------------------------------
Os1bglu4         --------F--TE---------------------------------------------------------------------------------------
Os1bglu5         --------F--TH---------------------------------------------------------------------------------------
Os3bglu6         --------F--AH---------------------------------------------------------------------------------------
Os3bglu7         --------F--AH---------------------------------------------------------------------------------------
Os3bglu8         --------F--VH---------------------------------------------------------------------------------------
Os4bglu10        --------F--TH---------------------------------------------------------------------------------------
Os4bglu11        --------F--TH---------------------------------------------------------------------------------------
Os4bglu12        --------F--TH---------------------------------------------------------------------------------------
Os4bglu13        --------F--TH---------------------------------------------------------------------------------------
Os4bglu14        --------F--TH---------------------------------------------------------------------------------------
Os4bglu16        --------F--TH---------------------------------------------------------------------------------------
Os4bglu18        --------F--TH---------------------------------------------------------------------------------------
Os4bglu27        --------Y--AH---------------------------------------------------------------------------------------
Os4bglu28        --------F--SR---------------------------------------------------------------------------------------
Os4bglu9         --------F--TY---------------------------------------------------------------------------------------
Os5bglu19        --------Y--AH---------------------------------------------------------------------------------------
Os5bglu21        --------Y--TH---------------------------------------------------------------------------------------
Os5bglu22        --------F--TH---------------------------------------------------------------------------------------
Os5bglu23        VQQKVYIFYGPKH---------------------------------------------------------------------------------------
Os5gblu20        --------Y--TH---------------------------------------------------------------------------------------
Os6bglu24        --------F--TH---------------------------------------------------------------------------------------
Os6bglu25        --------L--TK---------------------------------------------------------------------------------------
Os7bglu26        --------F--IE---------------------------------------------------------------------------------------
Os9bglu29        ----------------------------------------------------------------------------------------------------
Os9bglu30        --------F--TH---------------------------------------------------------------------------------------
Os9bglu31        --------F--SH---------------------------------------------------------------------------------------
Os9bglu32        --------F--TH---------------------------------------------------------------------------------------
Os9bglu33        --------F--IN---------------------------------------------------------------------------------------
SB01G010825      --------F--VH---------------------------------------------------------------------------------------
SB01G010830      --------F--VH---------------------------------------------------------------------------------------
SB01G013360      ----------------------------------------------------------------------------------------------------
SB01G043030      --------F--AH---------------------------------------------------------------------------------------
SB02G028400      --------F--TH---------------------------------------------------------------------------------------
SB02G029620      --------F--TH---------------------------------------------------------------------------------------
SB02G041550      --------F--IE---------------------------------------------------------------------------------------
SB03G037780      --------F--TH---------------------------------------------------------------------------------------
SB03G042690      --------F--TD---------------------------------------------------------------------------------------
SB06G019830      --------F--LV---------------------------------------------------------------------------------------
SB06G019840      --------F--TH---------------------------------------------------------------------------------------
SB06G019850      ----------------------------------------------------------------------------------------------------
SB06G019860      --------F--TH---------------------------------------------------------------------------------------
```

```
SB06G019880        --------F--TH---------------------------------------------------------------------------------------
SB06G022410        --------F--TH---------------------------------------------------------------------------------------
SB06G022420        --------F--NQ---------------------------------------------------------------------------------------
SB06G022450        --------F--TH---------------------------------------------------------------------------------------
SB06G022460        --------F--TH---------------------------------------------------------------------------------------
SB06G022490        --------F--TH---------------------------------------------------------------------------------------
SB06G022500        --------F--TH---------------------------------------------------------------------------------------
SB06G022510        --------F--TH---------------------------------------------------------------------------------------
SB08G007586        --------F--CH---------------------------------------------------------------------------------------
SB08G007650        --------F--CH---------------------------------------------------------------------------------------
SB09G018160        --------Y--AH---------------------------------------------------------------------------------------
SB10G012220        --------Y--TH---------------------------------------------------------------------------------------
SB10G022300        --------Y--TH---------------------------------------------------------------------------------------
SB10G027600        --------L--TR---------------------------------------------------------------------------------------
SB10G028060        --------F--LK---------------------------------------------------------------------------------------
SbDhr1             --------F--CH---------------------------------------------------------------------------------------
SbDhr2             --------F--CH---------------------------------------------------------------------------------------
Zm-p60.1           --------F--CH---------------------------------------------------------------------------------------
ZmGLU2             --------F--CH---------------------------------------------------------------------------------------
ZmGLU3             --------F--CH---------------------------------------------------------------------------------------
ZmGLU4             --------F--CH---------------------------------------------------------------------------------------
ZmGLU5             --------F--CH---------------------------------------------------------------------------------------
ZmGLU6             --------F--CH---------------------------------------------------------------------------------------
ZmGLU7             --------F--CH---------------------------------------------------------------------------------------
ZmGLU8             --------F--CH---------------------------------------------------------------------------------------
```

```
                                1010      1020      1030      1040      1050      1060      1070      1080      1090      1100
                            ....|....|....|....|....|....|....|....|....|....|....|....|....|....|....|....|....|....|....|....|
AC155376.2_FGT005           -------------------------------------------------D--------------------------------------------------
AC211140.2_FGT007           -------------------------------------------------T--------------------------------------------------
AC217401.3_FGT001           -------------------------------------------------T--------------------------------------------------
AC217687.3_FGT004           -------------------------------------------------V--------------------------------------------------
AtBglu1                     -------------------------------------------------C--------------------------------------------------
AtBglu10                    -------------------------------------------------T--------------------------------------------------
AtBglu11                    -------------------------------------------------A--------------------------------------------------
AtBglu12                    -------------------------------------------------KY-------------------------------------------------
AtBglu13                    -------------------------------------------------KY-------------------------------------------------
AtBglu14                    -------------------------------------------------KY-------------------------------------------------
AtBglu15                    -------------------------------------------------KY-------------------------------------------------
AtBglu16                    -------------------------------------------------KF-------------------------------------------------
AtBglu17                    -------------------------------------------------QY-------------------------------------------------
AtBglu18                    -------------------------------------------------KF-------------------------------------------------
AtBglu19                    -------------------------------------------------KF-------------------------------------------------
AtBglu2                     ----------------------------------------------------------------------------------------------------
AtBglu20                    -------------------------------------------------KF-------------------------------------------------
AtBglu21                    -------------------------------------------------RN-------------------------------------------------
AtBglu22                    -------------------------------------------------RN-------------------------------------------------
AtBglu23                    -------------------------------------------------RY-------------------------------------------------
AtBglu24                    -------------------------------------------------KY-------------------------------------------------
AtBglu25                    -------------------------------------------------KQ-------------------------------------------------
AtBglu26                    -------------------------------------------------RF-------------------------------------------------
AtBglu27                    -------------------------------------------------KY-------------------------------------------------
AtBglu28                    -------------------------------------------------TF-------------------------------------------------
AtBglu29                    -------------------------------------------------TF-------------------------------------------------
AtBglu3                     -------------------------------------------------T--------------------------------------------------
AtBglu30                    -------------------------------------------------TY-------------------------------------------------
AtBglu31                    -------------------------------------------------AF-------------------------------------------------
AtBglu32                    -------------------------------------------------AF-------------------------------------------------
AtBglu33                    -------------------------------------------------MF-------------------------------------------------
AtBglu34                    -------------------------------------------------RY-------------------------------------------------
AtBglu35                    -------------------------------------------------RY-------------------------------------------------
AtBglu36                    -------------------------------------------------RY-------------------------------------------------
AtBglu37                    -------------------------------------------------RY-------------------------------------------------
AtBglu38                    -------------------------------------------------RF-------------------------------------------------
AtBglu39                    -------------------------------------------------RY-------------------------------------------------
AtBglu4                     -------------------------------------------------S--------------------------------------------------
AtBglu40                    -------------------------------------------------T--------------------------------------------------
AtBglu41                    -------------------------------------------------EK-------------------------------------------------
AtBglu42                    -------------------------------------------------I--------------------------------------------------
AtBglu43                    -------------------------------------------------I--------------------------------------------------
AtBglu44                    -------------------------------------------------I--------------------------------------------------
AtBglu45                    -------------------------------------------------KN-------------------------------------------------
AtBglu46                    -------------------------------------------------EN-------------------------------------------------
AtBglu47                    -------------------------------------------------I--------------------------------------------------
```

```
AtBglu5          -------------------------------------------------S--------------------------------------------------
AtBglu7          -------------------------------------------------C--------------------------------------------------
AtBglu8          -------------------------------------------------C--------------------------------------------------
AtBglu9          -------------------------------------------------S--------------------------------------------------
BD1G10890        -------------------------------------------------T--------------------------------------------------
BD1G10920        -------------------------------------------------T--------------------------------------------------
BD1G10930        -------------------------------------------------I--------------------------------------------------
BD1G10940        -------------------------------------------------T--------------------------------------------------
BD1G19270        -------------------------------------------------I--------------------------------------------------
BD1G33040        -------------------------------------------------R--------------------------------------------------
BD1G42690        -------------------------------------------------NH-------------------------------------------------
BD1G70170        -------------------------------------------------T--------------------------------------------------
BD2G09190        ARGCRPPLYLRAATLTRLRAQQMSHDRRPRIYLRHRKKWIVQGLFPSAYKF-------------------------------------------------
BD2G09200        -------------------------------------------------AH-------------------------------------------------
BD2G27770        -------------------------------------------------A--------------------------------------------------
BD2G37450        ----------------------------------------------------------------------AVGGGGPARLVPPPPPLTPRSKGRSCLPPL
BD2G57640        -------------------------------------------------N--------------------------------------------------
BD2G59650        -------------------------------------------------A--------------------------------------------------
BD2G59660        -------------------------------------------------A--------------------------------------------------
BD3G00650        -------------------------------------------------T--------------------------------------------------
BD3G40000        -------------------------------------------------T--------------------------------------------------
BD3G40010        -------------------------------------------------T--------------------------------------------------
BD3G45610        -------------------------------------------------S--------------------------------------------------
BD3G45630        -------------------------------------------------A--------------------------------------------------
BD3G45640        -------------------------------------------------A--------------------------------------------------
BD3G45650        -------------------------------------------------A--------------------------------------------------
BD4G08040        -------------------------------------------------F--------------------------------------------------
BD4G09920        -------------------------------------------------EHSCDDKEAMCDQKPADAAMASAGGDGGS----------------------
BD4G34930        -------------------------------------------------S--------------------------------------------------
BD4G34940        -------------------------------------------------Q--------------------------------------------------
BD4G34950        -------------------------------------------------Q--------------------------------------------------
BD5G13260        -------------------------------------------------QH-------------------------------------------------
BD5G13270        -------------------------------------------------D--------------------------------------------------
BD5G15530        -------------------------------------------------K--------------------------------------------------
BD5G15540        -------------------------------------------------TR-------------------------------------------------
GRMZM2G012236    -------------------------------------------------I--------------------------------------------------
GRMZM2G015804    -------------------------------------------------L--------------------------------------------------
GRMZM2G031660    -------------------------------------------------V--------------------------------------------------
GRMZM2G055699    -------------------------------------------------A--------------------------------------------------
GRMZM2G069024    -------------------------------------------------A--------------------------------------------------
GRMZM2G108133    -------------------------------------------------E--------------------------------------------------
GRMZM2G110948    -------------------------------------------------I--------------------------------------------------
GRMZM2G112704    -------------------------------------------------Y--------------------------------------------------
GRMZM2G128197    -------------------------------------------------T--------------------------------------------------
GRMZM2G148176    -------------------------------------------------V--------------------------------------------------
GRMZM2G163544    -------------------------------------------------R--------------------------------------------------
GRMZM2G177661    -------------------------------------------------T--------------------------------------------------
GRMZM2G376416    ----------------------------------------------------------------------------------------------------
```

```
GRMZM2G457040    -------------------------------------------------TH-------------------------------------------------
Os10bglu34       -------------------------------------------------T--------------------------------------------------
Os11bglu36       -------------------------------------------------ETSCDDNGNVRDQRPVDALMASAAGDGGS----------------------
Os12bglu38       -------------------------------------------------Q--------------------------------------------------
Os1bglu1         -------------------------------------------------T--------------------------------------------------
Os1bglu2         -------------------------------------------------E--------------------------------------------------
Os1bglu3         -------------------------------------------------A--------------------------------------------------
Os1bglu4         -------------------------------------------------N--------------------------------------------------
Os1bglu5         -------------------------------------------------Q--------------------------------------------------
Os3bglu6         -------------------------------------------------T--------------------------------------------------
Os3bglu7         -------------------------------------------------T--------------------------------------------------
Os3bglu8         -------------------------------------------------T--------------------------------------------------
Os4bglu10        -------------------------------------------------QY-------------------------------------------------
Os4bglu11        -------------------------------------------------QS-------------------------------------------------
Os4bglu12        -------------------------------------------------QH-------------------------------------------------
Os4bglu13        -------------------------------------------------QH-------------------------------------------------
Os4bglu14        -------------------------------------------------K--------------------------------------------------
Os4bglu16        -------------------------------------------------TQ-------------------------------------------------
Os4bglu18        -------------------------------------------------L--------------------------------------------------
Os4bglu27        -------------------------------------------------I--------------------------------------------------
Os4bglu28        -------------------------------------------------I--------------------------------------------------
Os4bglu9         -------------------------------------------------QH-------------------------------------------------
Os5bglu19        -------------------------------------------------S--------------------------------------------------
Os5bglu21        -------------------------------------------------S--------------------------------------------------
Os5bglu22        -------------------------------------------------A--------------------------------------------------
Os5bglu23        -------------------------------------------------K--------------------------------------------------
Os5gblu20        -------------------------------------------------S--------------------------------------------------
Os6bglu24        -------------------------------------------------NH-------------------------------------------------
Os6bglu25        -------------------------------------------------R--------------------------------------------------
Os7bglu26        -------------------------------------------------K--------------------------------------------------
Os9bglu29        ----------------------------------------------------------------------------------------------------
Os9bglu30        -------------------------------------------------I--------------------------------------------------
Os9bglu31        -------------------------------------------------S--------------------------------------------------
Os9bglu32        -------------------------------------------------Q--------------------------------------------------
Os9bglu33        -------------------------------------------------Q--------------------------------------------------
SB01G010825      -------------------------------------------------T--------------------------------------------------
SB01G010830      -------------------------------------------------T--------------------------------------------------
SB01G013360      ----------------------------------------------------------------------------------------------------
SB01G043030      -------------------------------------------------T--------------------------------------------------
SB02G028400      -------------------------------------------------I--------------------------------------------------
SB02G029620      -------------------------------------------------E--------------------------------------------------
SB02G041550      -------------------------------------------------V--------------------------------------------------
SB03G037780      -------------------------------------------------A--------------------------------------------------
SB03G042690      -------------------------------------------------D--------------------------------------------------
SB06G019830      -------------------------------------------------L--------------------------------------------------
SB06G019840      -------------------------------------------------QH-------------------------------------------------
SB06G019850      ----------------------------------------------------------------------------------------------------
SB06G019860      -------------------------------------------------QH-------------------------------------------------
```

```
SB06G019880    -------------------------------------------------QH-------------------------------------------------
SB06G022410    -------------------------------------------------I--------------------------------------------------
SB06G022420    -------------------------------------------------K--------------------------------------------------
SB06G022450    -------------------------------------------------K--------------------------------------------------
SB06G022460    -------------------------------------------------T--------------------------------------------------
SB06G022490    -------------------------------------------------TH-------------------------------------------------
SB06G022500    -------------------------------------------------I--------------------------------------------------
SB06G022510    -------------------------------------------------A--------------------------------------------------
SB08G007586    -------------------------------------------------NF-------------------------------------------------
SB08G007650    -------------------------------------------------EY-------------------------------------------------
SB09G018160    -------------------------------------------------S--------------------------------------------------
SB10G012220    -------------------------------------------------TH-------------------------------------------------
SB10G022300    -------------------------------------------------S--------------------------------------------------
SB10G027600    -------------------------------------------------R--------------------------------------------------
SB10G028060    -------------------------------------------------Y--------------------------------------------------
SbDhr1         -------------------------------------------------NF-------------------------------------------------
SbDhr2         -------------------------------------------------TY-------------------------------------------------
Zm-p60.1       -------------------------------------------------NH-------------------------------------------------
ZmGLU2         -------------------------------------------------NF-------------------------------------------------
ZmGLU3         -------------------------------------------------NY-------------------------------------------------
ZmGLU4         -------------------------------------------------NF-------------------------------------------------
ZmGLU5         -------------------------------------------------TF-------------------------------------------------
ZmGLU6         -------------------------------------------------SF-------------------------------------------------
ZmGLU7         -------------------------------------------------KY-------------------------------------------------
ZmGLU8         -------------------------------------------------NH-------------------------------------------------
```

```
                          1110      1120      1130      1140      1150      1160      1170      1180      1190      1200
                    ....|....|....|....|....|....|....|....|....|....|....|....|....|....|....|....|....|....|....|....|
AC155376.2_FGT005   ----------------------------------------------------------K------ER-VL------------------------------
AC211140.2_FGT007   ----------------------------------------------------------F------GK-IS------------------------------
AC217401.3_FGT001   ----------------------------------------------------------P------GN-IV------------------------------
AC217687.3_FGT004   ----------------------------------------------------------P------GN-IA------------------------------
AtBglu1             -----------------------------------------------------------------R----------------------------------
AtBglu10            -----------------------------------------------------------------YN-RG------------------------------
AtBglu11            -----------------------------------------------------------------GH--S------------------------------
AtBglu12            ----------------------------------------------------------P------EK-IK------------------------------
AtBglu13            ----------------------------------------------------------P------EK-IK------------------------------
AtBglu14            ----------------------------------------------------------P------EK-IK------------------------------
AtBglu15            ----------------------------------------------------------P------EK-IK------------------------------
AtBglu16            ----------------------------------------------------------P------EK-IM------------------------------
AtBglu17            ----------------------------------------------------------P------EK-IS------------------------------
AtBglu18            ----------------------------------------------------------P------H--RC------------------------------
AtBglu19            ----------------------------------------------------------P------H--RV------------------------------
AtBglu2             ----------------------------------------------------------------------------------------------------
AtBglu20            ----------------------------------------------------------P------H--KC------------------------------
AtBglu21            ----------------------------------------------------------P------E--RC------------------------------
AtBglu22            ----------------------------------------------------------P------E--RC------------------------------
AtBglu23            ----------------------------------------------------------P------E--RC------------------------------
AtBglu24            ----------------------------------------------------------P------E--KC------------------------------
AtBglu25            ----------------------------------------------------------H------SE-SN------------------------------
AtBglu26            ----------------------------------------------------------P------HR-IS------------------------------
AtBglu27            ----------------------------------------------------------P------E--RN------------------------------
AtBglu28            ----------------------------------------------------------P------E--RT------------------------------
AtBglu29            ----------------------------------------------------------P------E--RT------------------------------
AtBglu3             -----------------------------------------------------------------R----------------------------------
AtBglu30            ----------------------------------------------------------P------E--RT------------------------------
AtBglu31            ----------------------------------------------------------P------E--RT------------------------------
AtBglu32            ----------------------------------------------------------P------E--RT------------------------------
AtBglu33            ----------------------------------------------------------P------EK-VQ------------------------------
AtBglu34            ----------------------------------------------------------P------EK-VP------------------------------
AtBglu35            ----------------------------------------------------------P------EK-VP------------------------------
AtBglu36            ----------------------------------------------------------P------ER-VS------------------------------
AtBglu37            ----------------------------------------------------------P------EKGGA------------------------------
AtBglu38            ----------------------------------------------------------P------EKGGA------------------------------
AtBglu39            ----------------------------------------------------------P------EKGGP------------------------------
AtBglu4             -----------------------------------------------------------------R----------------------------------
AtBglu40            ----------------------------------------------------------F------GK-IT------------------------------
AtBglu41            ----------------------------------------------------------P------GK-IL------------------------------
AtBglu42            ----------------------------------------------------------E------GK-IL------------------------------
AtBglu43            ----------------------------------------------------------P------GK-IA------------------------------
AtBglu44            ----------------------------------------------------------P------GK-IA------------------------------
AtBglu45            ----------------------------------------------------------P------GK-IL------------------------------
AtBglu46            ----------------------------------------------------------P------GK-IV------------------------------
AtBglu47            ----------------------------------------------------------S------GK-IA------------------------------
```

```
AtBglu5        -----------------------------------------------------------------R----------------------------------
AtBglu7        -----------------------------------------------------------------D---S------------------------------
AtBglu8        -----------------------------------------------------------------Y----------------------------------
AtBglu9        ---------------------------------------------------------------------Y------------------------------
BD1G10890      ----------------------------------------------------------P------GA-PKAPIAIAAYYAYHFCCAYCLRESGRFSDKEI
BD1G10920      ----------------------------------------------------------P------VF-TA------------------------------
BD1G10930      ----------------------------------------------------------P------GN-VV------------------------------
BD1G10940      ----------------------------------------------------------P------GN-IA------------------------------
BD1G19270      ----------------------------------------------------------P------GM-IS------------------------------
BD1G33040      ----------------------------------------------------------P------GR-VI------------------------------
BD1G42690      ----------------------------------------------------------P------DK-IA------------------------------
BD1G70170      ----------------------------------------------------------F------GK-IT------------------------------
BD2G09190      ----------------------------------------------------------C------NT-GD------------------------------
BD2G09200      ----------------------------------------------------------A------HA-GD------------------------------
BD2G27770      -----------------------------------------------------------------GK-MP------------------------------
BD2G37450      QPLAITRRSLDEWPKAGSDDVGEWPN-----------------------------PTTP------GA-SK------------------------------
BD2G57640      ----------------------------------------------------------K------EH-IL------------------------------
BD2G59650      -----------------------------------------------------------------GR-LS------------------------------
BD2G59660      -----------------------------------------------------------------GK-TP------------------------------
BD3G00650      ----------------------------------------------------------F------GK-IL------------------------------
BD3G40000      ----------------------------------------------------------P------GK-TA------------------------------
BD3G40010      ----------------------------------------------------------P----------A------------------------------
BD3G45610      -----------------------------------------------------------------GK-MP------------------------------
BD3G45630      -----------------------------------------------------------------GK-MP------------------------------
BD3G45640      -----------------------------------------------------------------GK-MP------------------------------
BD3G45650      -----------------------------------------------------------------GK-MP------------------------------
BD4G08040      ----------------------------------------------------------P------GA-TP------------------------------
BD4G09920      -------------------QPASRTTGVEK--GAVGEQRKPLRVAMEAMLRGFEKFSDG------GE-SS------------------------------
BD4G34930      -----------------------------------------------------------------GY-SI------------------------------
BD4G34940      -----------------------------------------------------------------GY-SF------------------------------
BD4G34950      -----------------------------------------------------------------GY-SY------------------------------
BD5G13260      ----------------------------------------------------------P------DK-IT------------------------------
BD5G13270      ----------------------------------------------------------D------DK-IA------------------------------
BD5G15530      ----------------------------------------------------------Q------GT-IR------------------------------
BD5G15540      ----------------------------------------------------------P------GG-IR------------------------------
GRMZM2G012236  ----------------------------------------------------------K------GN-ID------------------------------
GRMZM2G015804  ----------------------------------------------------------P------GK-IS------------------------------
GRMZM2G031660  ----------------------------------------------------------P------GR-IE------------------------------
GRMZM2G055699  -----------------------------------------------------------------GR-MP------------------------------
GRMZM2G069024  -----------------------------------------------------------------GR-MP------------------------------
GRMZM2G108133  -----------------------------------------------------------------GY-SL------------------------------
GRMZM2G110948  ----------------------------------------------------------P------GE-IA------------------------------
GRMZM2G112704  ----------------------------------------------------------P------GT-TP------------------------------
GRMZM2G128197  ----------------------------------------------------------P------GK-IA------------------------------
GRMZM2G148176  ----------------------------------------------------------P------GT-IP------------------------------
GRMZM2G163544  ----------------------------------------------------------P------GR-VI------------------------------
GRMZM2G177661  ----------------------------------------------------------F------GK-VA------------------------------
GRMZM2G376416  ----------------------------------------------------------------------------------------------------
```

```
GRMZM2G457040   ----------------------------------------------------------S------GG-VM------------------------------
Os10bglu34      ----------------------------------------------------------F------GK-II------------------------------
Os11bglu36      --------------------QQSWRSTGGENIGDR-EQRKPLRVAMEAMLRGFEILAES------GE-SA------------------------------
Os12bglu38      ----------------------------------------------------------P------GV-TP------------------------------
Os1bglu1        ----------------------------------------------------------P------GE-IA------------------------------
Os1bglu2        -----------------------------------------------------------------GK-TK------------------------------
Os1bglu3        -----------------------------------------------------------------GK-MK------------------------------
Os1bglu4        ----------------------------------------------------------K------ER-IL------------------------------
Os1bglu5        -----------------------------------------------------------------GK-MP------------------------------
Os3bglu6        ----------------------------------------------------------F------GK-IT------------------------------
Os3bglu7        ----------------------------------------------------------P------GN-VA------------------------------
Os3bglu8        ----------------------------------------------------------P------GN-IA------------------------------
Os4bglu10       ----------------------------------------------------------P------DK-IT------------------------------
Os4bglu11       ----------------------------------------------------------P------DK-IT------------------------------
Os4bglu12       ----------------------------------------------------------P------EK-IA------------------------------
Os4bglu13       ----------------------------------------------------------P------DK-IA------------------------------
Os4bglu14       ----------------------------------------------------------Q------GT-IE------------------------------
Os4bglu16       ----------------------------------------------------------A------GR-IS------------------------------
Os4bglu18       ----------------------------------------------------------P------GN-IK------------------------------
Os4bglu27       ----------------------------------------------------------P------GK-VE------------------------------
Os4bglu28       ----------------------------------------------------------P------GK-IA------------------------------
Os4bglu9        ----------------------------------------------------------P------DK-IA------------------------------
Os5bglu19       -----------------------------------------------------------------WR-NP------------------------------
Os5bglu21       -----------------------------------------------------------------GR-HP------------------------------
Os5bglu22       -----------------------------------------------------------------GK-MP------------------------------
Os5bglu23       -----------------------------------------------------------------GR-MA------------------------------
Os5gblu20       -----------------------------------------------------------------GR-HP------------------------------
Os6bglu24       ----------------------------------------------------------P------EK-IA------------------------------
Os6bglu25       ----------------------------------------------------------P------GR-VI------------------------------
Os7bglu26       ----------------------------------------------------------P------GT-IP------------------------------
Os9bglu29       ----------------------------------------------------------------------------------------------------
Os9bglu30       ----------------------------------------------------------P------GK-IL------------------------------
Os9bglu31       -----------------------------------------------------------------GY-SV------------------------------
Os9bglu32       -----------------------------------------------------------------GY-SP------------------------------
Os9bglu33       -----------------------------------------------------------------GY-MP------------------------------
SB01G010825     ----------------------------------------------------------P------GK-IA------------------------------
SB01G010830     ----------------------------------------------------------P------GN-IV------------------------------
SB01G013360     ----------------------------------------------------------------------------------------------------
SB01G043030     ----------------------------------------------------------F------GK-IS------------------------------
SB02G028400     ----------------------------------------------------------P------GK-IL------------------------------
SB02G029620     ----------------------------------------------------------------------------------------------------
SB02G041550     ----------------------------------------------------------P------GT-IP------------------------------
SB03G037780     -----------------------------------------------------------------GN-MP------------------------------
SB03G042690     ----------------------------------------------------------K------ER-VL------------------------------
SB06G019830     ----------------------------------------------------------A------DR-IA------------------------------
SB06G019840     ----------------------------------------------------------P------DK-IA------------------------------
SB06G019850     ----------------------------------------------------------D------DK-IA------------------------------
SB06G019860     ----------------------------------------------------------PGRFMIQDK-IA------------------------------
```

```
SB06G019880        ----------------------------------------------------------P------DK-II------------------------------
SB06G022410        ----------------------------------------------------------Q------GK-IV------------------------------
SB06G022420        ----------------------------------------------------------S------GK-IE------------------------------
SB06G022450        ----------------------------------------------------------Q------GT-VE------------------------------
SB06G022460        ----------------------------------------------------------Q------GK-VE------------------------------
SB06G022490        ----------------------------------------------------------T------GA-IM------------------------------
SB06G022500        ----------------------------------------------------------P------GR-IE------------------------------
SB06G022510        ----------------------------------------------------------P------GR-IK------------------------------
SB08G007586        ----------------------------------------------------------P------EW-IV------------------------------
SB08G007650        ----------------------------------------------------------P------ER-IA------------------------------
SB09G018160        -----------------------------------------------------------------AR-NP------------------------------
SB10G012220        ----------------------------------------------------------P------EK-IA------------------------------
SB10G022300        -----------------------------------------------------------------GY-SI------------------------------
SB10G027600        ----------------------------------------------------------P------GR-VI------------------------------
SB10G028060        ----------------------------------------------------------P------GT-TP------------------------------
SbDhr1             ----------------------------------------------------------P------EW-IV------------------------------
SbDhr2             ----------------------------------------------------------P------DF-IA------------------------------
Zm-p60.1           ----------------------------------------------------------P------ER-IL------------------------------
ZmGLU2             ----------------------------------------------------------P------ER-IM------------------------------
ZmGLU3             ----------------------------------------------------------P------DW-IL------------------------------
ZmGLU4             ----------------------------------------------------------P------EW-IL------------------------------
ZmGLU5             ----------------------------------------------------------P------DW-IA------------------------------
ZmGLU6             ----------------------------------------------------------P------SL-IA------------------------------
ZmGLU7             ----------------------------------------------------------P------EW-IA------------------------------
ZmGLU8             ----------------------------------------------------------P------EW-IA------------------------------
```

```
                                 1210      1220      1230      1240      1250      1260      1270      1280      1290      1300
                    ....|....|....|....|....|....|....|....|....|....|....|....|....|....|....|....|....|....|....|....|
AC155376.2_FGT005   --DRS-------------NAEIAV-DHYHRYK----------------EDI-----ELMASLGFSA-Y-RFSISWARIFP------DGL-----GEKVNE
AC211140.2_FGT007   --DFS-------------NADVAV-DQYHRFE----------------EDV-----QLMADMGMDA-Y-RFSIAWSRILP------NGT-----G-QVNQ
AC217401.3_FGT001   --YNQ-------------TADVAV-DQYHRYR----------------EDV-----DLMKSLNFDA-Y-RFSISWSRIFP------DGE-----G-RVNP
AC217687.3_FGT004   --GNQ-------------NGDVAV-DQYHRYK----------------EDV-----DLMKSLNFDA-Y-RFSISWSRIFP------DGE-----G-KVNP
AtBglu1             --KMD-------------NGDIAC-DGYHKYK----------------EDV-----QLMAETGLHT-F-RFSISWSRLIS------NGR-----G-SINP
AtBglu10            --NLG-------------NGDITS-DGYHKYK----------------EDV-----KLMAEMGLES-F-RFSISWSRLIP------NGR-----G-LINP
AtBglu11            --GVA-------------AGNVAC-DQYHKYK----------------EDV-----KLMADMGLEA-Y-RFSISWSRLLP------SGR-----G-PINP
AtBglu12            --DGS-------------NGSIAS-DSYHLYK----------------EDV-----GLLHQIGFDA-Y-RFSISWSRILP------REN---LKG-GINQ
AtBglu13            --DGT-------------NGSIAS-DSYHLYK----------------EDV-----GLLHQIGFGA-Y-RFSISWSRILP------RGN---LKG-GINQ
AtBglu14            --DGS-------------NGSIAD-DSYHLYK----------------EDV-----GLLHQIGFNA-Y-RFSISWSRILP------RGN---LKG-GINQ
AtBglu15            --DGS-------------NGSVAD-NSYHLYK----------------EDV-----ALLHQIGFNA-Y-RFSISWSRILP------RGN---LKG-GINQ
AtBglu16            --DGS-------------NGSIAD-DSYNLYK----------------EDV-----NLLHQIGFDA-Y-RFSISWSRILP------RGT---LKG-GINQ
AtBglu17            --DGS-------------NGDVAD-EFYYRFK----------------EDV-----AHMKEIGLDS-F-RFSISWSRILP------RGT---VAG-GVNQ
AtBglu18            --ENH-------------NADVAV-DFYHRYK----------------EDI-----QLMKDLNTDA-F-RLSIAWPRIFP------HGR---MSK-GISK
AtBglu19            --KNH-------------NADEAV-DFYHRYK----------------EDI-----QLMKKLNTDG-F-RLSISWPRIFP------HGR---MEK-GISK
AtBglu2             ------------------D---------------------------------------------------------------------------------
AtBglu20            --NYH-------------NADVAV-DFYHRYK----------------EDI-----KLMKNLNTDG-F-RFSIAWPRIFP------HGR---MEK-GISK
AtBglu21            --SGD-------------HADVAV-DFFHRYK----------------EDI-----QLMKNLNTDA-F-RLSIAWSRIFP------HGR---KEK-GVSQ
AtBglu22            --SGH-------------NADVAV-DFFHRYK----------------EDI-----QLMKNLNTDA-F-RLSIAWSRIFP------HGR---KEK-GVSQ
AtBglu23            --NND-------------NGDVAV-DFFHRYK----------------EDI-----QLMKNLNTDA-F-RMSIAWPRIFP------HGR---KEK-GVSQ
AtBglu24            --NGD-------------NGTQAV-DFFYRYK----------------EDI-----QLMKNLNTDS-F-RLSISWTRIFP------HGR---EEN-GVSK
AtBglu25            --NNL-------------DGRLGV-DFYHHYK----------------EDV-----QLLKKLNMDA-F-RFSISWSRIFP------HGK---KDK-GVSE
AtBglu26            --DSS-------------DGNVAV-DFYHRYK----------------EDI-----KRMKDINMDS-F-RLSIAWPRVLP------YGK---RDR-GVSE
AtBglu27            --CYS-------------NADQAI-EFYNHYK----------------DDI-----QRMKDINMDA-F-RFSISWPRIFP------LGK---KSK-GVNK
AtBglu28            --RMQ-------------NADVAV-DFYHRYK----------------DDI-----KLMKELNMDA-F-RFSISWARLIP------SGK---VKD-GVNK
AtBglu29            --NMQ-------------NADVAV-DFYHRYK----------------DDI-----KLIEELNVDA-F-RFSISWARLIP------SGK---VKD-GVNK
AtBglu3             --NLS-------------NGDITS-DGYHKYK----------------EDV-----KLMVETGLDA-F-RFSISWSRLIP------NGR-----G-PVNP
AtBglu30            --KMH-------------NADVAI-DFYHRYK----------------DDI-----KLMKELNMDA-F-RFSISWSRLIP------SGK---LKD-GVNK
AtBglu31            --NMD-------------NGDVAV-DFYHRYK----------------EDI-----KLIKEMNMDS-F-RFSLSWSRILP------SGK---LSD-GVNK
AtBglu32            --NMD-------------NGDVAV-DFYHRYK----------------DDI-----KLIKEMNMDS-F-RFSLSWSRILP------SGK---LSD-GVNK
AtBglu33            --QNG-------------DGDEGV-DFYTRYK----------------DDI-----KLMKELNTNG-F-RFSISWTRILP------YGT---IKK-GVNE
AtBglu34            --DRS-------------SGDLAC-DSYDLYK----------------DDV-----KLLKRMNVQA-Y-RLSIAWSRVLP------KGR---LTG-GVDE
AtBglu35            --DRS-------------SADLAC-DSYDLYK----------------DDV-----KLLKRMNVQA-Y-RLSIAWSRVLP------KGR---LTG-GVDE
AtBglu36            --DRS-------------IGDLAC-NSYDLYK----------------DDV-----KLLKRMNVQA-Y-RFSIAWSRVLP------KGR---LIG-GVDE
AtBglu37            --DLG-------------NGDTTC-DSYRTWQ----------------KDL-----DVMEELGVKG-Y-RFSFAWSRILP------KGK---RSR-GINE
AtBglu38            --DLG-------------NGDTTC-DSYTLWQ----------------KDI-----DVMDELNSTG-Y-RFSIAWSRLLP------KGK---RSR-GVNP
AtBglu39            --DLG-------------NGDSTC-GSYEHWQ----------------KDI-----DVMTELGVDG-Y-RFSLAWSRIAP------RE---------SNQ
AtBglu4             --DQG-------------NGDIAC-DGYHKYK----------------DDV-----KLMVDTNLDA-F-RFSISWSRLIP------NGR-----G-PVNQ
AtBglu40            --DFS-------------NADVAV-DQYHRYE----------------EDV-----QLMKNMGMDA-Y-RFSISWTRIFP------NGV-----G-HINE
AtBglu41            --DFS-------------NADTTV-DQYHRFH----------------NDI-----DLMKDLRMDA-Y-RFSISWSRIFP------NGT-----G-EVNP
AtBglu42            --DGS-------------NGDVAV-DHYHRYK----------------EDV-----DLIGQLGFGA-Y-RFSISWSRIFP------DGL-----GTEVNE
AtBglu43            --NNA-------------TAEITV-DQYHRYK----------------EDV-----DLMQNLNIDA-Y-RFSISWSRIFP------EGS-----G-KINS
AtBglu44            --KNA-------------TAEITV-DQYHRYK----------------EDV-----DLMKKLNFDA-Y-RFSISWSRIFP------EGS-----G-KVNW
AtBglu45            --DKN-------------NADRAV-DQYNRFL----------------EDI-----QLMSFLGVNS-Y-RFSISWCRILP------RGR---F-G-EINY
AtBglu46            --DGS-------------NGDIAT-DQYHRYM----------------EDI-----QSMNFLGVNS-Y-RLSISWSRVLP------NGR---F-G-VINY
AtBglu47            --DGS-------------HGKVAV-DHYHRYP----------------GDL-----DLMEDLGVNS-Y-RLSLSWARILP------KGR---F-G-DVNM
```

```
AtBglu5        --NIG-------------NGDVTC-DGYHKYK----------------EDV-----KLMVDTNLDA-F-RFSISWSRLIP------NGR-----G-SVNQ
AtBglu7        --GSN-------------NGDIAC-DGYHKYK----------------EDV-----MLMAEMGLES-F-RFSISWSRLIP------NGR-----G-RINP
AtBglu8        --NGS-------------NGDIAC-DGYHKYK----------------EDV-----KLMAEMGLES-F-RFSISWSRLIP------NGR-----G-RINP
AtBglu9        --DTG-------------NGDVTS-DGYHKYK----------------EDV-----KLMATMGLES-F-RFSISWSRLIP------NGR-----G-LINP
BD1G10890      LWGIK-------------MQTVTT-DQYHHYKNCQERIPGTYVFMLAQEDV-----NLMKGLNFDA-Y-RFSISWSRIFP------DGE-----G-KVNE
BD1G10920      --YES-------------PVDFLISSEDHTFKI------------ADVEDV-----NLMKGLNFDA-Y-RFSISWSRIFP------DGE-----G-KVNE
BD1G10930      --GNT-------------NADVTT-DQYHRYK----------------EDV-----NLMKGLNFDA-Y-RFSISWSRIFP------DGE-----G-KVNE
BD1G10940      --GNG-------------NADVTT-DEYHHYK----------------EDV-----ELMKSLNFDA-Y-RFSISWSRIFP------DGE-----G-RVNE
BD1G19270      --GNG-------------TADVAV-DEYHRYK----------------EDV-----DIMKSMGFDA-Y-RFSISWSRIFP------NGA-----G-KVNQ
BD1G33040      --DFS-------------NADVAV-DHYHRYK----------------EDV-----DLMKDIGVDA-Y-RFSISWSRIFP------NGT-----G-KPNE
BD1G42690      --NGS-------------TGDVAI-DSYHRYK----------------DDV-----SIMKDLGFDA-Y-RFSLSWSRILP------SGK---PSG-GVNI
BD1G70170      --DFS-------------NADVAV-DQYHRFE----------------EDI-----QLMADMGMDA-Y-RFSIAWARILP------NGV-----G-QVNQ
BD2G09190      --DPV-------------NGDVAA-DGYHKYK----------------EDI-----KLMKETGLDA-Y-RFSISWSRLIP------NGR-----G-EVNP
BD2G09200      --DPV-------------DGSVAA-DGYHKYK----------------EDI-----KLMKETGLDA-Y-RFSISWSRLIP------NGR-----G-EVNP
BD2G27770      --DKS-------------TGDLGA-DGYHRYK----------------EDV-----ELMSDTGLEA-Y-RFSISWSRLIP------RGR-----G-PLNP
BD2G37450      --AGCGG--PA-----------SA-KPGEGLR----------------LDL-----STLRSQGRKDQIAFFDKECSKVAE------HVYLGG--------
BD2G57640      --DGT-------------SGEVAV-DHYHRYK----------------EDI-----ELMAKLGFGA-Y-RFSISWSRIFP------DGL-----GKEINE
BD2G59650      --DKS-------------TGDVAS-DGYHRYK----------------DDV-----KLMADTNLEA-Y-RFSISWSRLIP------DGR-----G-TVNP
BD2G59660      --DKS-------------VGDVAA-DGYHKYK----------------DDV-----KLMAETNLEA-Y-RFSISWSRLIP------NGR-----G-AVNP
BD3G00650      --DFS-------------NADVAV-DHYHRFE----------------EDI-----ELMADMGLDA-Y-RFSIAWSRILP------NGT-----G-EVNQ
BD3G40000      --DNG-------------TTDIAN-DFYHRYK----------------EDL-----QLITDMNMDT-F-RFSIAWSRILP------TGT---IAG-GINQ
BD3G40010      --DGG-------------TGDVAN-DFYHRYK----------------EDL-----NFITAMNMDT-F-RFSLAWSRILP------NGT---ISG-GVSK
BD3G45610      --DKS-------------TGDIAA-DGYHKYK----------------QNS-----W---------------------FT------DGR-----G-AVNP
BD3G45630      --DKS-------------TGDIAA-DGYHKYK----------------EDM-----KLISKTGLEA-Y-RFSISWSRLIPTKIAVPDGR-----G-AVNP
BD3G45640      --DKS-------------TGDIAA-DGYHKYK----------------EDL-----KLISETGLEA-Y-RFSISWSRLIP------NGR-----G-AVNP
BD3G45650      --DKS-------------TGDIAA-DGYHKYK----------------EDL-----KLVSETGLEA-Y-RFSISWSRLIP------NGR-----G-AVNP
BD4G08040      --DNA-------------TANVTV-DEYHRYM----------------DDV-----DNMVRVGFDA-Y-RFSISWSRIFP------SGI-----G-RINK
BD4G09920      --GGDNCSHNVAAWHNVPNPQERL-KFWSDPD----------------TEL-----KLAKETGISV-F-RMGVDWTRIMP------KEPTEDLKS-SVNY
BD4G34930      --DGA-------------TADVTA-NQYHKYK----------------EDV-----KLLSEMGVDA-Y-RFSIAWPRLIP------DGR-----G-AVNP
BD4G34940      --DGS-------------TADVSA-DQYHHYK----------------EDV-----KLMHNMGLDA-Y-RFSIAWPRLIP------DGR-----G-QINP
BD4G34950      --DKS-------------TADISA-DQYHHYK----------------DDV-----KLMHEIGLDA-Y-RFSIAWPRLIP------DGR-----G-RINP
BD5G13260      --DRS-------------NGDVAA-DSYHLYK----------------EDV-----RLMKDMGMDA-Y-RFSISWTRILP------YGT---LRG-GVNR
BD5G13270      --DRS-------------NGDVAV-DSYHLYK----------------EDV-----RLMKDMGMDA-Y-RFSISWTRILP------NGT---LRG-GVNT
BD5G15530      --GGS-------------NGDTAA-DHYHRYM----------------EDI-----ELMHSLGVNS-Y-RFSIAWTRILP------RGR---F-G-DVNP
BD5G15540      --DGR-------------NGDVAD-DHYHRYM----------------EDV-----EIMHNLGVNS-Y-RFSISWSRILP------RGR---L-G-GVNS
GRMZM2G012236  --DGS-------------NGDMAT-DHYHRYK----------------DDI-----EMMHSIGLTS-Y-KFSLSWSRILP------KGR---F-G-GINQ
GRMZM2G015804  --DFS-------------NADVAV-DQYHRFE----------------EDV-----QLMADMGMDA-Y-RFSIAWSRILP------NGT-----G-QVNQ
GRMZM2G031660  --DGS-------------TGDVAD-DHYHRYE----------------DDI-----ELMHSLGTNA-Y-RFSISWARILP------RGR---F-G-EVNP
GRMZM2G055699  --DKS-------------TGDLGA-DGYHKYK----------------GDV-----QLMSDTGLEA-Y-RFSISWSRLIP------RGR-----G-AINP
GRMZM2G069024  --DKS-------------NGDVAA-DGYNKYK----------------DDV-----KLIIDNNLEA-Y-RFSISWSRLIP------NGR-----G-AINP
GRMZM2G108133  --DNA-------------TGDVTA-DQYHKYK----------------DDV-----KLLHEMGVDA-Y-RMSIAWPRLIP------DGR-----G-AVNP
GRMZM2G110948  --NNA-------------TADVTV-DEYHRYK----------------EDV-----NIMKNMGFDA-Y-RFSISWSRIFP------NGT-----G-EVNW
GRMZM2G112704  --DNA-------------TADVTV-DEYNRYM----------------DDV-----DNMVRVGFDA-Y-RFSISWSRIFP------SGI-----G-RVNK
GRMZM2G128197  --EDA-------------NADVTT-DEYHRYK----------------EDV-----DLMKSLNFDA-Y-RFSISWSRIFP------DGE-----G-KINE
GRMZM2G148176  --NNA-------------TADVTV-DEYHRYK----------------EDV-----NIMKNMGFDA-Y-RFSISWSRIFP------DGT-----G-KVNQ
GRMZM2G163544  --DFS-------------NADVAV-DHYHRYK----------------EDV-----DLIKDIGMDA-Y-RFSISWSRIFP------NGT-----G-EPNE
GRMZM2G177661  --DLS-------------NADVAV-DQYHRFE----------------EDI-----QLMADMGMDA-Y-RFSIAWSRILP------NGT-----G-QVNQ
GRMZM2G376416  ----------------------------------------------------------------------------HVFV------DGR-----G-AVNP
```

```
GRMZM2G457040   --DGR-------------TGDVAD-DHYHRYV----------------GDL-----EILQSLGVNA-Y-RFSISWARVLP------RGR---V-G-GVNA
Os10bglu34      --DFS-------------NADVAV-DQYHRFE----------------EDI-----QLMADMGMDA-Y-RFSISWSRIFP------NGT-----G-EVNQ
Os11bglu36      --GGDNCSHNVAAWHNVPCPQERL-RFWSDPD----------------AEL-----KLAKETGISV-F-RMGVDWARLMP------EEPTEELKS-SVNF
Os12bglu38      --DNS-------------TANVTV-DEYHRYM----------------DDV-----DNMVRVGFDA-Y-RFSISWSRIFP------SGL-----G-KINK
Os1bglu1        --NNA-------------TADVTV-DEYHRYK----------------EDV-----NIMKSMGFDA-Y-RFSISWSRIFP------TGT-----G-KVNW
Os1bglu2        --DKG-------------TGDVAA-DGYHKYK----------------GDV-----KLMAETGLEA-Y-KFSISWSRLIP------NGR-----G-AVNQ
Os1bglu3        --DKS-------------TGDVAS-DGYHKYK----------------GDV-----KLMTETGLEA-Y-RFSISWSRLIP------SGR-----G-AVNQ
Os1bglu4        --DGS-------------SGEVAV-DHYHRYK----------------EDI-----ELMASLGFRA-Y-RFSISWPRIFP------DGL-----GKNVNE
Os1bglu5        --GRS-------------TADVAA-DGYHKYK----------------DDL-----KLMVDTNLEA-Y-RLSISWSRIIP------NGR-----G-DVNP
Os3bglu6        --DFS-------------NADVAV-DQYHRFE----------------EDI-----QLMADMGMDA-Y-RFSIAWSRIYP------NGV-----G-QVNQ
Os3bglu7        --GNQ-------------NGDVAT-DQYHRYK----------------EDV-----NLMKSLNFDA-Y-RFSISWSRIFP------DGE-----G-RVNQ
Os3bglu8        --GNG-------------NADVTT-DEYHRYK----------------EDV-----DLLKSLNFDA-Y-RFSISWSRIFP------DGE-----G-KVNT
Os4bglu10       --DKS-------------NGDGAC-NSYHLYK----------------EDV-----RIMKEMGMDA-Y-RFSISWSRILP------NGS---LSG-GVNR
Os4bglu11       --DRS-------------NGDVAC-DSYHLYK----------------EDV-----RSMKEMGMDA-Y-RFSISWSRILP------NGS---LSG-GVNR
Os4bglu12       --DRS-------------NGDVAS-DSYHLYK----------------EDV-----RLMKDMGMDA-Y-RFSISWTRILP------NGS---LRG-GVNK
Os4bglu13       --DKS-------------NGDVAA-DSYHLYK----------------EDV-----RIMKDMGVDA-Y-RFSISWTRILP------NGS---LSG-GINR
Os4bglu14       --DGS-------------NGDTAN-DHYHRYM----------------EDI-----ELMHSLGVNS-Y-RFSISWARILP------KGR---F-G-DVNP
Os4bglu16       --DGR-------------NGDVAD-DHYHRYT----------------EDV-----DILHNLGVNS-Y-RFSISWARILP------RGR---L-G-GVNS
Os4bglu18       --DGS-------------NGDIAD-DHYHRYE----------------EDV-----ELMNSLGVNA-Y-RFSISWSRILP------KGR---F-G-GVNP
Os4bglu27       --DGS-------------NGDVAV-DFYHRYK----------------EDL-----NFVTDMNMDA-F-RFSIAWSRILP------NGT---ISG-GINK
Os4bglu28       --DGS-------------NADIAN-DFYHRYK----------------EDL-----NLITAMNMDS-F-RFSIAWSRILP------NGT---ISG-GINK
Os4bglu9        --DKS-------------NGDVAD-NTYHLYK----------------EDV-----HMMKEMGMDA-Y-RFSISWSRILP------NGS---LNG-GVNI
Os5bglu19       --GGE-------------TGDVAC-DGYHKYK----------------EDV-----MLMNETGLEA-Y-RFTISWSRLIP------SGR-----G-AVNP
Os5bglu21       --EDE-------------TGDVAS-DGYHKYK----------------LPKGAIVPQLMSKEGAGA-T-QTRRSHRRHSS------KGG-----R-----
Os5bglu22       --DKS-------------TGDMGA-GGYHKYK----------------EDV-----KLMSDTSLEA-Y-RFSISWSRLIP------RGR-----G-PVNP
Os5bglu23       --DNS-------------TGDRAA-AGYHKYK----------------EDV-----KLMSDTGLEA-Y-RFSISWSRLIP------RGR-----G-PINP
Os5gblu20       --EDG-------------TGDVAS-DGYHKYK----------------EDV-----KLMTEIGLEA-Y-RFTISWSRLIP------SGR-----G-AVNP
Os6bglu24       --NGS-------------NGDIAI-DSYHRYK----------------EDV-----GIMKGLGLNA-Y-RFSVSWPRILP------S-------------
Os6bglu25       --DFS-------------NADVAV-DHYHRYK----------------EDV-----ELMNDIGMDA-Y-RFSISWSRIFP------NGT-----G-EPNE
Os7bglu26       --NNA-------------TADVTV-DEYHRYK----------------EDV-----NIMKNMGFDA-Y-RFSISWSRIFP------NGT-----G-MVNQ
Os9bglu29       ------------------------------------------------EDV-----NLLKDMNMDA-F-RFSISWSRILP------NGT---LSG-GVNK
Os9bglu30       --NND-------------NGDVAN-DFYHRYK----------------EDV-----SLLKDMNMDA-F-RFSIAWTRILP------NGS---LSG-GINK
Os9bglu31       --DGA-------------TGDVTA-DQYHKYK----------------EDV-----KLLQDMGVDA-Y-RMSISWSRLIP------DGR-----G-AVNP
Os9bglu32       --GGA-------------IADVSA-DQYHHYK----------------EDV-----KLMYDMGLDA-Y-RFSIAWPRLIP------DGR-----G-EINP
Os9bglu33       --DGS-------------NADVSA-DQYHHYK----------------EDV-----KLMYDMGLDA-Y-RFSIAWPRLIP------DGR-----G-EINP
SB01G010825     --EDG-------------NADVTT-DEYHRYK----------------EDV-----DLMKSLNFDA-Y-RFSISWSRIFP------DGE-----G-KVNE
SB01G010830     --GNQ-------------TADVAV-DQYHRYR----------------EDV-----DLMKSLNFDA-Y-RFSISWSRIFP------DGE-----G-RVNP
SB01G013360     -------------------------------------------------------------MGMDA-Y-RFSIAWPRIFP------NGT-----G-EVNQ
SB01G043030     --DFS-------------NADVAV-DQYHRFE----------------EDV-----QLMADMGMDA-Y-RFSIAWSRILP------NGT-----G-QVNQ
SB02G028400     --NND-------------TGDVAD-DMYHRYK----------------EDV-----QLLKDMNLDA-F-RFSIAWTRILP------NGS---LSG-GINK
SB02G029620     --------------------------------------------------------------------------------------DGR-----G-AVNP
SB02G041550     --NNA-------------TADVTV-DEYHRYK----------------EDV-----NIMKNMGFDA-Y-RFSISWSRIFP------NGT-----G-KVNQ
SB03G037780     --DKS-------------NGDIAA-DGYNKYK----------------DDV-----KLVIDSNLEA-Y-RFSISWSRLIP------NGR-----G-AINP
SB03G042690     --DKS-------------NAEIAV-DHYHRYK----------------EDI-----ELMASLGFSA-Y-RFSISWARIFP------DGL-----GEKVNE
SB06G019830     --NRS-------------NGDVAV-NSYHLYK----------------EDV-----RLMKDMGMDA-Y-RFSISWTRILP------NGS---LSG-GVNR
SB06G019840     --DRS-------------NGDVAV-DSYHRYK----------------EDV-----RIMKDMGVDA-Y-RFSISWTRILP------NGS---LSG-GVNR
SB06G019850     --NRS-------------NGDVAV-DSYHLYK----------------EDV-----RIMKSMGMDA-Y-RFSISWSRILP------YGS---LSG-GVNR
SB06G019860     --DRS-------------NGDVAV-DSYHLYK----------------EDV-----RIMKGMGMDA-Y-RFSISWSRILP------NGS---LSG-GVNR
```

```
SB06G019880      --DRS-------------NGDVAI-DSYHLYK----------------EDV-----RLLKDMGMDA-Y-RFSISWTRILP------NGS---LSG-GINR
SB06G022410      --DGS-------------NGDVAA-DHYHRYK----------------EDI-----EMMHSVGLDS-Y-RFSLSWSRILP------KGR---F-G-DVNP
SB06G022420      --DGS-------------NGDVAT-DHYHRYK----------------EDI-----EIMHSLGLDC-Y-RFSLSWSRILP------KGR---F-G-GVNP
SB06G022450      --DGT-------------NGDTAD-DHYHRYM----------------EDI-----ELIHSLGVNS-Y-RFSIAWARILP------KGR---F-G-HVNP
SB06G022460      --DGT-------------NGDTAD-DHYHHYM----------------EDI-----ELMHSMGVNS-Y-RFSIAWARILP------KGR---F-G-HVNP
SB06G022490      --DGR-------------TGDVAD-DHYHRYM----------------GDV-----EILQSLGVNA-Y-RFSISWARILP------RGR---L-G-GVNA
SB06G022500      --DGS-------------TGDTAD-DHYHRFE----------------DDV-----ELMHSLGTNA-Y-RFSISWARILP------RGR---F-G-QVNP
SB06G022510      --DRS-------------TGDVAD-DHYHRYE----------------EDI-----ELMHSLGTNA-Y-RFSISWARVLP------KGR---F-G-KVNP
SB08G007586      --DRS-------------NGDVAA-DSYHMYA----------------EDV-----RLLKEMGMDA-Y-RFSISWPRILP------KGT---LAG-GINE
SB08G007650      --DRS-------------NGDVAA-DSYHMYA----------------DDV-----KLLKEMGMDA-Y-RFSISWSRILP------KGT---IAG-GINE
SB09G018160      --NEH-------------SGDFAA-DGYHKYK----------------EDV-----KLMKDIGLKA-Y-RFTISWSRLIP------NGR-----G-AVNP
SB10G012220      --DGS-------------NGDMAI-DSYHRYQ----------------EDV-----KIMKDIGFNA-Y-RFSISWTRILP------NGK---LSG-GVNM
SB10G022300      --DHA-------------TGDVAA-DQYHHYK----------------EDV-----KLMHDMGLDA-Y-RFSIAWSRLIP------DGR-----G-AVNP
SB10G027600      --DFS-------------NADIAV-DHYHRYK----------------EDV-----DLIKDIGMDA-Y-RFSISWSRIFP------NGT-----G-EPNE
SB10G028060      --DNA-------------TADVSV-DEYDRYM----------------DDV-----DNMVRVGFDA-Y-RFSISWSRIFP------SGI-----G-RVNK
SbDhr1           --DRS-------------NGDVAA-DSYHMYA----------------EDV-----RLLKEMGMDA-Y-RFSISWPRILP------KGT---LAG-GINE
SbDhr2           --DKS-------------NGDVAA-DSYHLYE----------------EDV-----KLLKEMGMDA-Y-RFSISWPRILP------NGT---L-S-DINE
Zm-p60.1         --DGS-------------NSDIGA-NSYHMYK----------------TDV-----RLLKEMGMDA-Y-RFSISWPRILP------KGT---KEG-GINP
ZmGLU2           --DGS-------------NADIGA-NSYHMYK----------------TDV-----RLLKEMGMDA-Y-RFSISWPRILP------KGT---VEG-GINQ
ZmGLU3           --DGS-------------NGDTGA-NSYHMYP----------------ADV-----RLLKEIGMDA-Y-RFSISWSRILP------KGT---LEG-GINQ
ZmGLU4           --DKS-------------NGDVAA-DSYHMYP----------------EDV-----RLLKEIGMDA-Y-RFSISWSRILP------NGT---LEG-GINP
ZmGLU5           --DHS-------------NGDVAA-DSYHMYK----------------EDV-----RLLKEIGMDS-Y-RFSISWSRILP------NGT---LEG-GINP
ZmGLU6           --DGS-------------NGDVAA-NSYHMYH----------------EDV-----RLMKEIGLDA-Y-RFSVSWSRILP------KGT---LEG-GINQ
ZmGLU7           --DGS-------------TGDVGA-NSYYLYR----------------EDV-----RLLKEMGMDA-Y-RFSIAWSRILP------KGT---LEG-GINH
ZmGLU8           --DGS-------------SGDVGA-NSYYLYR----------------EDV-----RLLKEMGMDA-Y-RFSISWPRILP------KGT---LEG-GINH
```

```
                    1310      1320      1330      1340      1350      1360      1370      1380      1390      1400
                ....|....|....|....|....|....|....|....|....|....|....|....|....|....|....|....|....|....|....|....|
AC155376.2_FGT005  QGVAFYNDLINFMISKG-------------------IEPYATLYHWDLPNNLQKT---L-GGWIS-D---KIV-------EYFALYAEACFANFGDRVK-
AC211140.2_FGT007  AGIDHYNKVINALLSKG-------------------IQPYVTLYHWDLPQALEDR---Y-NGWLD-R---QIV-------NDFAAYAETCFKAFGDRVK-
AC217401.3_FGT001  EGVAYYNNLINYLLRKG-------------------ITPYANLYHSDLPLALQNK---Y-GGWLN-A---KMA-------KLFTDYADFCFKTFGDRVK-
AC217687.3_FGT004  EGVAYYNNLINYLLQQG-------------------MTPYINLYHYDLPLALEKK---Y-GGWLS-A---KMA-------DLFTDYADFCFKTYGDRVK-
AtBglu1            KGLQFYKNFIQELVKHG-------------------IEPHVTLHHYDFPQYLEDD---Y-GGWTN-R---KII-------KDFTAYADVCFREFGNHVK-
AtBglu10           KGLLFYKNLIKELISHG-------------------IEPHVTLYHYDLPQSLEDE---Y-GGWIN-R---KII-------EDFTAYADVCFREFGEDVK-
AtBglu11           KGLQYYNNLIDELITHG-------------------IQPHVTLHHFDLPQALEDE---Y-GGWLS-Q---EIV-------RDFTAYADTCFKEFGDRVS-
AtBglu12           AGIDYYNNLINELLSKG-------------------IKPFATIFHWDTPQSLEDA---Y-GGFLG-A---EIV-------NDFRDYADICFKNFGDRVK-
AtBglu13           AGIDYYNNLINELLSKG-------------------IKPFATIFHWDTPQSLEDA---Y-GGFFG-A---EIV-------NDFRDYADICFKNFGDRVK-
AtBglu14           AGIDYYNNLINELLSKG-------------------IKPFATIFHWDTPQDLEDA---Y-GGFRG-A---EIV-------NDFRDYADICFKSFGDRVK-
AtBglu15           AGIDYYNNLINELLSKG-------------------IKPFATMFHWDTPQALEDA---Y-GGFRG-A---EIV-------NDFRDYADICFKNFGDRVK-
AtBglu16           AGIEYYNNLINQLISKG-------------------VKPFVTLFHWDLPDALENA---Y-GGLLG-D---EFV-------NDFRDYAELCFQKFGDRVK-
AtBglu17           AGINFYNHLINELISNG-------------------IRPLVTLFHWDTPQALEDE---Y-GGFLN-P---QIV-------KDFVEYVDICFKEFGDRVK-
AtBglu18           VGVQFYHDLIDELLKNN-------------------IIPLVTVFHWDTPQDLEDE---Y-GGFLS-G---RIV-------QDFTEYANFTFHEYGHKVK-
AtBglu19           EGVQFYHDLIDELLKND-------------------ITPLVTVFHWDTPADLEDE---Y-GGFLS-E---RIV-------PDFVEYANFTFHEYGDKVK-
AtBglu2            ----------------------------------------------------------------G-P---IAE-------SYFTAYADVCFREFGNHVK-
AtBglu20           AGVQYYHDLIDELLANG-------------------ITPLVTVFHWDTPQDLEDE---Y-GGFLS-D---RII-------KDFTEYANFTFQEYGDKVK-
AtBglu21           AGVQFYHELIDELLKNG-------------------IVPFVTVFHWDTPQDLEDE---Y-GGFLS-Q---NIV-------KDFREYADYVFTEYGGKVK-
AtBglu22           AGVKFYHDLIDELLKNG-------------------IIPFVTVFHWDTPQDLEDE---Y-GGFLS-E---NIV-------KDFREYADYVFTEYGGKVK-
AtBglu23           AGVQFYHDLIDELIKNG-------------------ITPFVTVFHWDTPQDLEDE---Y-GGFLS-E---RIV-------KDFREYADFVFQEYGGKVK-
AtBglu24           SGVQFYHDLIDELKRNG-------------------IIPFVTVFHWDTPQTLENE---Y-GGFLS-A---HIV-------KDFREYAEFVFKEYGGKVK-
AtBglu25           TGVKFYNDLINELIANG-------------------VTPLVTLFQWDVPQALEDE---Y-GGFLS-D---RIL-------EDFRDFAQFAFNKYGDRVK-
AtBglu26           EGIKFYNDVIDELLANE-------------------ITPLVTIFHWDIPQDLEDE---Y-GGFLS-E---QII-------DDFRDYASLCFERFGDRVS-
AtBglu27           EGIQFYNDLIDELLANG-------------------ITPLATLFHWDTPQALEDE---Y-SGFLS-E---EAV-------DDFKDFAALCFEEFGDRVK-
AtBglu28           EGVEFYKALIDELVANG-------------------IEPSMTLYHWDHPQSLEDE---Y-GGFLS-P---QIV-------EDFRDFSRVCFEEFGDKVK-
AtBglu29           EGVQFYKALIDELIANG-------------------IQPSVTLYHWDHPQALEDE---Y-GGFLN-P---QII-------EDFRNFARVCFENFGDKVK-
AtBglu3            KGLQFYKNFIQELVSHG-------------------IEPHVTLFHYDHPQYLEDE---Y-GGWIN-R---RII-------QDFTAYANVCFREFGHHVK-
AtBglu30           EGVQFYKDLIDELLAND-------------------IQPSMTLYHWDHPQSLEDE---Y-GGFLS-P---KIV-------EDFRDFARICFEEFGDKVK-
AtBglu31           EGVQFYKNLIDELIENG-------------------IKPFVTIYHWDIPQALDDE---Y-GSFLS-P---RII-------DDFRNYARFCFQEFGDKVS-
AtBglu32           EGVQFYKNLIDELIKNG-------------------IKPFVTIYHWDIPQALDDE---Y-GSFLS-P---RII-------DDFRNFARFCFQEFGDKVS-
AtBglu33           EGVKFYNDLINELLANG-------------------IQPSVTLFHWESPLALEME---Y-GGFLN-E---RIV-------EDFREFANFCFKEFGDRVK-
AtBglu34           NGITYYNNLINELKANG-------------------IEPYVTIFHWDVPQTLEDE---Y-GGFLS-T---RIV-------EDYTNYAELLFQRFGDRVK-
AtBglu35           NGITYYNNLINELKANG-------------------IEPYVTIFHWDVPQTLEDE---Y-GGFLS-T---RIV-------EDYTNYAELLFQRFGDRVK-
AtBglu36           NGITYYNNLINELKANG-------------------IEPFVTIFHWDVPQDFRRR---I-WRLLK-P---T-Y-------SDFKNYAELLFQRFGDRVK-
AtBglu37           DGINYYSGLIDGLIARN-------------------ITPFVTLFHWDLPQSLQDE---Y-EGFLD-R---TII-------DDFKDYADLCFERFGDRVK-
AtBglu38           GAIKYYNGLIDGLVAKN-------------------MTPFVTLFHWDLPQTLQDE---Y-NGFLN-K---TIV-------DDFKDYADLCFELFGDRVK-
AtBglu39           AGVKYYNDLIDGLLAKN-------------------ITPFVTLFHWDLPQVLQDE---Y-EGFLN-H---EII-------DDFKDYANLCFKIFGDRVK-
AtBglu4            KGLQFYKNLIQELVSHG-------------------IEPHVTLYHYDHPQSLEDE---Y-GGWLN-H---RMI-------KDFTTYADVCFREFGNHVK-
AtBglu40           AGIDHYNKLINALLAKG-------------------IEPYVTLYHWDLPQALHDR---Y-LGWLN-P---QII-------NDFAAYAEVCFQRFGDRVK-
AtBglu41           DGVKYYNSLIDALLAKG-------------------IKPYVTLYHWDLPQALEDR---Y-EGWLS-R---EVV-------DDFEHYAFTCFKAFGDRVK-
AtBglu42           EGIAFYNDLINTLLEKG-------------------IQPYVTLYHWDLPSHLQEA---I-GGWTN-R---KIV-------DYFGLYADACFANFGDRVK-
AtBglu43           NGVAYYNRLIDYLIEKG-------------------ITPYANLYHYDLPLALEQK---Y-QGLLS-K---Q---------GRFCGLRRVLFQTFGDRVK-
AtBglu44           KGVAYYNRLIDYMVQKG-------------------ITPYANLYHYDLPLALENK---Y-KGLLG-R---QVV-------KDFADYAEFCYKTFGDRVK-
AtBglu45           LGIKYYNIFIDALISRG-------------------IKPFVTLNHVDYPQELEDR---F-QSWLN-P---EMQ-------KEFGYLADICFKHFGNRVK-
AtBglu46           KGIKYYNNLIDALIKKG-------------------ITPFVTLNHFDYPQELENR---F-KSWLS-S---EMQ-------KDFGYLADICFKHFGDRVK-
AtBglu47           GGIDHYNRMINDILKTG-------------------IEPFVTLTHYDIPQELEYR---Y-GSWLN-P---QIR-------EDFEHYANICFRHFGDRVK-
```

AtBglu5	KGLQFYKNLISELITHG-------------------IEPHVTLYHYDHPQYLEDE---Y-GGWVN-N---MMI-------KDFTAYVDVCFREFGNYVK-
AtBglu7	KGLLFYKNLIKELRSHG-------------------IEPQVTLYHYDLPQSLEDE---Y-GGWIN-R---KII-------EDFTAFADVCFREFGEDVK-
AtBglu8	KGLLFYKNLIKELRSHG-------------------IEPHVTLYHYDLPQSLEDE---Y-GGWIN-H---KII-------EDFTAFADVCFREFGEDVK-
AtBglu9	KGLLFYNNLIKDLKSHG-------------------IEPHVTLYHYDLPQSLEDE---Y-GGWIN-R---KII-------EDFTAYADVCFREFGEDVK-
BD1G10890	EGVAYYNNLIDYLLQKG-------------------ITPYINLYHYDLPLALEKK---Y-GGWLN-A---KTV-------ELFADYADFCFKTFGNRVK-
BD1G10920	EGVAYYNNLIDYLLQKG-------------------ITPYINLYHYDLPLALEKK---Y-GGWLN-A---KTV-------ELFADYADFCFKTFGNRVK-
BD1G10930	EGVAYYNNLINYLLQKG-------------------ITPYINLYHADLPLALEKK---Y-GGWLS-A---KTV-------ELFADYADFCFKTFGNRVK-
BD1G10940	EGVAYYNNLIDYVIKKG-------------------LIPYVNLNHYDIPLALQKK---Y-DGWLS-P---KIV-------NIFSDYAEFCFKTYGDRVQ-
BD1G19270	EGVDYYNRLIDYMLQQG-------------------ITPYANLYHYDLPLALHQQ---Y-LGWLS-P---KIV-------EAFADYADFCFKVFGDRVK-
BD1G33040	EGLSYYNSLIDVLLDKG-------------------IQPYVTLFHWDLPQALEDK---Y-GGWLN-S---QIV-------EDFVHYASTCFEEFGDRVK-
BD1G42690	EGIKYYNNLIDKLISKG-------------------IEPFVTLFHWDSPQVLEQQ---Y-GGFLS-H---LIV-------EDFHDYANICFREFGDRVK-
BD1G70170	AGIDHYNKLINALLAKG-------------------IQPYVTLYHWDLPQALEDK---Y-NGWLN-R---QIV-------DDFAGYAETCFAAFGDRVK-
BD2G09190	KGLAYYNNLINELLDHG-------------------IQPHVTMFHYDLPQILEDE---Y-DGWLS-P---QII-------GDFTAYADVCFREFGDRVT-
BD2G09200	KGLEYYNNLINELLDHG-------------------IQPHVTMFQYDLPLILEDE---Y-DGWLS-P---QII-------DDFTAYADVCFREFGDRVT-
BD2G27770	KGLEYYNNLINELTKRG-------------------IEIHVTLYHLDFPQILEDE---Y-HGWLS-P---RVV-------ADFTAFADACFREFGDRVR-
BD2G37450	---DAVAKNREILRKNG-------------------ITHVLNCVGFVCPE-YFKSDLVYRTLWLQ-D---SPT-------EDITSILYDVFDYFEDVREQ
BD2G57640	QGVAFYNNLIDFMIEKG-------------------IQPYATLYHWDLPHNLQQT---M-GGWLS-D---KIV-------EYFALYAEACFANFGDRVK-
BD2G59650	KGLEYYNNLIDELVKHG-------------------IQVHVMLHHLDFPQVLEDV---Y-GGWLS-P---RIV-------EDFTTFADVCFREFGDRVS-
BD2G59660	KGLEYYNNLIDELVKHG-------------------IQIHVMLHQLDYPQILDDE---Y-GGWLS-T---RIV-------EDFTAFADVCFGEFGDRVS-
BD3G00650	AGIDHYNKVINALIAKG-------------------IEPYVTLYHWDLPQALEDK---Y-MGLLD-R---QII-------NDYLAYAETCFEAFGDRVK-
BD3G40000	KGVDFYNSLIKEVLSRG-------------------LVPFVTIFHFDTPQALEDK---Y-GSFLS-D---KII-------KDYVEYADLVFGLFGDRIK-
BD3G40010	TGVAFYNSLIDEVVARG-------------------LTPFVTISHFDTPQALEDK---Y-GGFLS-E---NLV-------KDYVEYADLCFSLFGDRVK-
BD3G45610	KGLDYYNNIIDELVKRG-------------------IQTHITLHHIDLPQILEDE---Y-GGWLS-P---RIIFAPAKCSEDFTAYADVCFREFGDRVK-
BD3G45630	KGLKYYNNIINELVKHG-------------------IQIHITLHHIDLPQILEDE---Y-GGWLS-P---RII-------EDFTAYAGVCFREFGDRVK-
BD3G45640	KGLEYYNNIIDELVK------------------------------HDLPQILEDE---Y-GGWLS-S---RII-------EDFTAYADVCFREFGDRVK-
BD3G45650	KGLEYYNNIIDELVKHG-------------------IQIHITLHHVDLPQILEDE---Y-GGWLS-P---RII-------EDFTAYADICFREFGDRVT-
BD4G08040	DGVDYYHRLINYMLANK-------------------ITPYVVLYHYDLPEVLNNQ---Y-NGWLS-P---RVV-------SDFGNFADFCFKTYGDRVK-
BD4G09920	AALERYRWIIQQVRDHG-------------------MKVMLTLFHHSLPP-WAGE---Y-GGWKM-E---KTV-------NYFMDFVRLVVDRVSDLVD-
BD4G34930	KGLEYYNNLIDELLSYG-------------------IQPHVTIYHFDFPQALQDE---Y-KGMLS-R---RFI-------EDYTAYAEVCFKNFGDRVK-
BD4G34940	KGLEYYNSLIDELILNG-------------------IQPHVTIYHFDLPQVLQDE---Y-GGLLS-P---KFI-------EDYTSYAEVCFKSFGDRVK-
BD4G34950	KGLKYYNNLIDELIRHD-------------------IQPHVTIYHLDFPQSLQDE---Y-KGLLS-P---RFV-------DDYTAYADACFKSFGDRVK-
BD5G13260	EGIKYYNNLINELLSKA-------------------IC---HPFHWDSPQALEDK---Y-GGFLN-P---NII-------NDYKDYTEVCFREFGDRVK-
BD5G13270	EGIKYYNSLINELLCKG-------------------VQPFVTLFHWDSPQALEDK---Y-GGFLN-P---NII-------NDYKDYAEVCFREFGDRVK-
BD5G15530	DGVAFYNAIIDALWQKG-------------------IQPFVTIFHYDIPHELDER---Y-GGWLS-P---EIQ-------KDFGYFAEVCFKMFGDRVK-
BD5G15540	AGIAFYDRLIAALLQKG-------------------IEPFVTLNHFDVPHEMETR---Y-GSWLG-A---GIR-------EEFDYYADVCFRAFGDRVK-
GRMZM2G012236	AGIKFYNNLINGLLEKG-------------------IQPLVTINHYDIPEELQER---Y-NSWLN-P---EIQ-------EDFTYFAELCFKMFGDRVK-
GRMZM2G015804	AGIDHYNKVINALLSKG-------------------IQPYVTLYHWDLPQALEDR---Y-NGWLD-R---QIV-------NDFAAYAETCFKAFGDRVK-
GRMZM2G031660	AGIAFYNRLIDSLLLKG-------------------IEPFVTLSHYDIPQELEDR---Y-GAWLGGA---EAR-------RDFGHLADVCFAAFGDRVR-
GRMZM2G055699	KGLEYYNNLINELVKRG-------------------IEIHVTLSHLDYPQILEDE---Y-HGWLS-P---RMV-------DDFEAYADVCFREFGDRVR-
GRMZM2G069024	KGIEYYNNLIDELVTHG-------------------VQVHVMIYQLDLPQILEDE---Y-GGWLS-P---MVV-------EDFTAYADVCFREFGDRVS-
GRMZM2G108133	KGLEYYNNLIDELLSYG-------------------IQPHVTIYHFDFPQALQDE---Y-SGLIS-P---RFI-------EDFTAYADVCFSNFGDRVK-
GRMZM2G110948	KGVAYYNRLINYMVKKG-------------------ITPYANLYHYDLPEALEVR---Y-GGLLS-R---EVV-------RSFADYADFCFGAFGDRVK-
GRMZM2G112704	DGVDYYHRLINYLLANH-------------------ITPYVVLYHYDLPQVLQDQ---Y-NGWLS-P---RIL-------GDFTAFADFCFKTYGDRVK-
GRMZM2G128197	EGVQYYNNLIDYMIKQG-------------------LTPYANLNHYDLPLALQKK---Y-QGWLG-P---KIV-------DIFADYADFCFKTFGNRVK-
GRMZM2G148176	EGVDYYNRLIDYMLQQG-------------------IAPYANLYHYDLPLALHEQ---Y-LGWLS-P---KIV-------EAFADYAEFCFHAFGDRVK-
GRMZM2G163544	EGLNYYNSLINTLLDKG-------------------IQPYVTLFHWDLPQALEDR---Y-GGWLN-S---QIV-------DDFVHYASTCFKEFGDRVK-
GRMZM2G177661	AGVDHYNRFIDALLSKG-------------------IEPYVTLYHWDLPQALEDR---Y-NGWLD-R---QIV-------YDFAEYAETCFEAFGDRVR-
GRMZM2G376416	KGLEYYNSLIDELLRYG-------------------IQPHVTIYHFDLPQALQDE---Y-NGLLS-P---RII-------DDFTAYADVCFRSFGDRVK-

```
GRMZM2G457040   GGVAFYNRLIDALLQKG-------------------IQPFVTLNHFDMPRELEVR---Y-GGWLD-A---GIR-------EEYEHYADVCFGAFGDRVR-
Os10bglu34      AGIDHYNKLINALLAKG-------------------IEPYVTLYHWDLPQALEDK---Y-TGWLD-R---QII-------NDYAVYAETCFQAFGDRVK-
Os11bglu36      AALERYRWIIQRVREYG-------------------MKVMLTLFHHSLPP-WAGK---Y-GGWKM-E---KTV-------TYFMDFVRLVVDRVSNLVD-
Os12bglu38      DGVDYYHRLIDYMLANN-------------------IIPYVVLYHYDLPQVLHDQ---Y-KGWLH-P---RIV-------RDFVRFADFCFKTYGHKVK-
Os1bglu1        KGVAYYNRLINYMLKIG-------------------ITPYANLYHYDLPEALEVQ---Y-GGLLN-R---KIV-------EAFADYAEFCFKTFGDRVK-
Os1bglu2        EGLKYYNNVIDELAKRG-------------------IQPHIMLCHLDLPQALEDE---Y-DGWLS-P---RIV-------DDFTAYADVCFREFGDRVL-
Os1bglu3        QGLKYYNNIIDELTKRG-------------------IQVHVMLYHLDLPQALEDE---Y-AGWLS-P---RIV-------EDFTAYADVCFREFGDRVS-
Os1bglu4        QGVAFYNDLINFMIEKG-------------------IEPYATLYHWDLPHNLQQT---V-GGWLS-D---KIV-------EYFALYAEACFANFGDRVK-
Os1bglu5        KGLQYYNDIIDGLVKNG-------------------IQVHIMLYQLDLPQVLEDE---Y-DGWLS-P---RIL-------EDFKAYADVCFKEFGDRVA-
Os3bglu6        AGIDHYNKLIDALLAKG-------------------IQPYVTLYHWDLPQALEDK---Y-KGWLD-R---QIV-------DDFAAYAETCFREFGDRVK-
Os3bglu7        EGVAYYNNLINYLLQKG-------------------ITPYVNLYHYDLPLALEKK---Y-GGWLN-A---KMA-------DLFTEYADFCFKTFGNRVK-
Os3bglu8        EGVAYYNNLIDYVIKQG-------------------LIPYVNLNHYDLPLALQKK---Y-EGWLS-P---KIV-------GVFSDYAEFCFKTYGDRVK-
Os4bglu10       EGINYYNNLINELLSKE-------------------VQPFATLFHFDTPQALEDK---Y-KGFLS-P---NII-------NDYKDYAEICFKEFGDRVK-
Os4bglu11       EGISYYNNLINELLSKG-------------------VQPFVTLFHWDSPQALEDK---Y-KGFLS-P---NII-------NDYKEYAETCFKEFGDRVK-
Os4bglu12       EGIKYYNNLINELLSKG-------------------VQPFITLFHWDSPQALEDK---Y-NGFLS-P---NII-------NDFKDYAEICFKEFGDRVK-
Os4bglu13       EGISYYNNLINELLLKG-------------------VQPFVTLFHWDSPQALEDK---Y-NGFLS-P---NII-------NDYKEYAETCFKEFGDRVK-
Os4bglu14       DGVAFYNALIDGLVQKG-------------------IQPFVTICHYDIPHELDER---Y-GGWLS-P---EIQ-------KDFSYFAEVCFKLFGDRIK-
Os4bglu16       AGIAFYNRLINALLQKG-------------------IQPFVTLNHFDIPHELETR---Y-GGWLG-A---AIR-------EEFEYYSDVCFNAFGDRVR-
Os4bglu18       AGIDFYNKLIDSILLKG-------------------IQPFVTLTHYDIPQELEDR---Y-GAWLN-A---EIQ-------SDFGHFADVCFGAFGDRVK-
Os4bglu27       EGIAFYNSLINEVISRG-------------------LKPFVTIFHFDTPQALEDK---Y-RSFLS-E---NIV-------KDFVDYADVCFREFGDRVK-
Os4bglu28       EGVEFYNSLINEVIAKG-------------------LKPFVTIFHFDTPQALEDK---Y-GGFLS-E---NIV-------KDYVDYADLCFSLFGDRVK-
Os4bglu9        EGINYYNNLINELLLKG-------------------VQSFVTLFHYDTPQALEDK---Y-NGFLS-P---NII-------NDYKDYAEICFKEFGDRVK-
Os5bglu19       KGLQFYNSMINELVKAG-------------------IQIHAVLYHIDLPQSLQDE---Y-GGWVS-P---KVV-------DDFAAYADVCFREFGDRVA-
Os5bglu21       --------RSDVSWWDG-------------------IQIHVVMYHMDLPQSLQDE---Y-GGWIS-P---KIV-------DDFTAYADVCFREFGDRVV-
Os5bglu22       KGLEYYNSLIDELVERG-------------------IEIHVTLYHLDFPQILEDE---Y-HGWLS-P---RVI-------DDFTAYADVCFREFGDRVR-
Os5bglu23       KGLEYYNDLIDKLVKRGAQIFCAIPKKGEICDCSMGIEIHVTLYHLDFPQALQDE---Y-NGWLS-P---RII-------EDFTAYADVCFREFGDLVR-
Os5gblu20       KGLQFYNNMINELVKAG-------------------IQIQVALYHSDLPQSLQDE---Y-GGWIN-P---KIV-------DDFTAYADVCFREFGDRVA-
Os6bglu24       ------------------------------------VEPFVTLFHWDSPQALEQQ---Y-GGFLS-N---LIV-------EDFRDYADICFREFGDRVK-
Os6bglu25       EGLSYYNSLIDALLDKG-------------------IEPYVTLFHWDLPQALEDR---Y-GGWLN-S---EII-------EDFVQYAFTCFKEFGDRVK-
Os7bglu26       EGVDYYNRLIDYMVKKG-------------------IKPYANLYHYDLPLALHEQ---Y-LGWLS-P---NIV-------EAFADYADFCFQTFGDRVK-
Os9bglu29       EGVAFYNNLINEIIAKG-------------------MKPFVTIFHWDTPQALESK---Y-GGFLS-E---NII-------KDYVDFAEVCFREFGDRVK-
Os9bglu30       EGVAFYNSLINDVIAKG-------------------MIPFVTIFHWDTPLALESK---Y-GGFLS-E---DIV-------KEYVDFAEVCFREFGDRVK-
Os9bglu31       KGLEYYNNLIDELLSHG-------------------IQPHVTIYHFDFPQALQDE---Y-NGILS-P---RFV-------EDFTAYADVCFKNFGDRVK-
Os9bglu32       KGLEYYNNLIDELIMHG-------------------IQPHVTIYHFDLPQALQDE---Y-GGILS-P---RFI-------EDYTAYAEVCFKNFGDRVK-
Os9bglu33       KGLEYYNNLIDELIMHG-------------------IQPHVTIYHFDLPQALQDE---Y-GGILS-P---RFI-------EDYSAYAEVCFKNFGDRVK-
SB01G010825     EGVQYYNDLIDYMIKQG-------------------LTPYANLNHYDLPLALQKK---Y-QGWLG-P---KIV-------DIFADYADFCFKTFGDRVK-
SB01G010830     EGVAYYKNLISYLLQKG-------------------ITPYANLYHSDLPLALQNK---Y-GGWLN-P---KMA-------KLFTDYADFCFKSFGDHVK-
SB01G013360     AGIDHYNNLINALLAKG-------------------IEPYVTLYHWDLPQALEDK---Y-TGWLD-R---QII-------NDYAVYAETCFKAFGDRVK-
SB01G043030     AGIDHYNKVINALLSKG-------------------IQPYVTLYHWDLPQALEDR---Y-NGWLD-R---QIV-------NDFAAYAETCFKAFGDRVK-
SB02G028400     EGVAFYNNLINEVIAKG-------------------LKPFVTIFHWDTPLALEDK---Y-GGFLS-E---NII-------KDYVDFAEVCFKEFGDRVK-
SB02G029620     KGLEYYNNLIDELLSYG-------------------IQPHVTIYHFDFPQALQDE---Y-NGLIS-R---KFI-------EDFTAYADVCFKNFGNRVK-
SB02G041550     EGVDYYNRLIDYMLQQG-------------------ITPYANLYHYDLPLALHEQ---Y-LGWLS-P---KIV-------EAFADYAEFCFQTFGDRVK-
SB03G037780     KGLEYYNNLIDELATHG-------------------VQVHVMISQLDPPQILEDE---Y-GGWLS-P---KIV-------EDFTAYADVCFREFGDRVS-
SB03G042690     QGVAFYNDLINFMISKG-------------------IEPYATLYHWDLPNNLQKT---L-GGWIS-D---KIV-------EYFALYAEACFANFGDRVK-
SB06G019830     EGVRYYNNLINELLSKG-------------------LQPFVTLFHWDSPQALEDK---Y-GGFLS-P---NII-------NDYKDYSEVCFKEFGDRVK-
SB06G019840     EGIRYYNNLIDELLLKG-------------------VQPFVTLFHWDSPQALEDK---Y-GGFLS-P---NII-------NDYKDYAEVCIKEFGDRVK-
SB06G019850     EGIRYYNNLIDELLLKG-------------------IQPFVTLFHWDSPQALEDK---Y-GGFLS-P---NII-------NDYKDYAEVCFKEFGDRVK-
SB06G019860     EGIRYYNNLIDELLLKG-------------------IQPFVTLFHWDTPQALEDK---Y-GGFLS-S---SII-------NDYKDYAEVCFKEFGDRVK-
```

```
SB06G019880      EGIRYYNNLINELMSKG-------------------LQPFVTLFHWDSPQALEDK---Y-GGFLS-P---NMI-------NDYKDYAEVCFKEFGDRVK-
SB06G022410      AGVKFYNSLINGMLQKG-------------------IEPFVTINHYDIPEELQQR---Y-GSWLS-P---EIQ-------EDFTYFAEICFKMFGDRVK-
SB06G022420      AGVKFYNSLINGLLGKG-------------------IQPFVTINHYDIPQELQER---Y-GSWLS-P---EIQ-------EDFTYFAELCFKMFGDRVK-
SB06G022450      DGVAFYNALIDALLQRG-------------------IEPFVTISHYDIPYELEKR---Y-GGWLS-P---KIR-------RDFGYLADVCFRMFGDRVK-
SB06G022460      DGVALYNALIDALLQRG-------------------IEPFVTISHFDIPYELEER---Y-GGWLS-P---KIR-------RDFGYLADVCFRMFGDRVK-
SB06G022490      GGIAFYNRLIDALLQKG-------------------IQPFVTLNHFDMPHELDVR---Y-VGWLG-A---GIR-------DEFEYYADVCFAAFGDRVR-
SB06G022500      EGIAFYNKLIDSLLLKG-------------------IEPFVTLAHYDIPQELVDR---Y-GAWLS-T---EVQ-------RDFGYLADVCFAAFGDRVK-
SB06G022510      AGIAFYNKLIDSLLLKG-------------------IEPFVTLTHYDTPQELEDR---Y-GAWLS-A---EAR-------RDFGHLADVCFAAFGDRVK-
SB08G007586      KGVEYYNKLIDLLLENG-------------------MEPYITIFHWDAPQALVDT---Y-GGFLD-E---RII-------KDYTDFAKVCFEKFGKKVK-
SB08G007650      KGVEYYNKLIDLLLENG-------------------IEPYITIFHWDTPQALVDA---Y-GGFLD-D---RII-------TDYTDFAKVCFQKFGTKVK-
SB09G018160      KGLQFYNDMINELVKEG-------------------IQVHAALYHLDLPQILEDE---Y-NGWLS-P---RIV-------DDFTAYADVCFREFGDRVA-
SB10G012220      EGIKYYNNFINKLISEG-------------------IQPFVTLFHWDSPQALEQQ---Y-GGFLS-Q---LIV-------EDFRDYANICFREFGDRVK-
SB10G022300      KGLEYYNSLINELLRYG-------------------IQPHVTIYHFDLPQALQDE---Y-NGLLS-P---RII-------DDFTAYADVCFRSFGDRVK-
SB10G027600      EGLNYYNSLIDVLLDKG-------------------IQPYVTLFHWDLPQALEDR---Y-GGWLN-S---QIV-------DDFVHYASTCFKEFGDRVK-
SB10G028060      DGVDYYHRLIDYMLANH-------------------ITPYVVLYHYDLPQVLQDQ---Y-NGWLS-P---RIV-------PDFTAFADFCFKTYGDRVK-
SbDhr1           KGVEYYNKLIDLLLENG-------------------IEPYITIFHWDTPQALVDA---Y-GGFLD-E---RII-------KDYTDFAKVCFEKFGKKVK-
SbDhr2           KGIAYYNNLINLLIDNG-------------------IEPYVTIFHWDTPQALVDD---Y-GGFLD-K---RII-------KDYTDFAGLCFERFGDRVN-
Zm-p60.1         DGIKYYRNLINLLLENG-------------------IEPYVTIFHWDVPQALEEK---Y-GGFLD-KSHKSIV-------EDYTYFAKVCFDNFGDKVK-
ZmGLU2           DGIDYYKRLINLLLENG-------------------IEPYVTIFHWDVPQALEEK---Y-GGFLD-KTHKRIV-------NDYKNFAKVCFDNFGDKVK-
ZmGLU3           AGIKYYKKLINLLIENG-------------------IEPFVTIFHWDVPQALEDK---Y-GGFLG-D---RIV-------KDYTDFAKVCFENFGDKVK-
ZmGLU4           DGIKYYKNLINLLLENG-------------------IEPFVTLFHWDTPQALMDK---Y-GGFLD-K---SIV-------KDYTDFAKVCFDNFGDKVK-
ZmGLU5           YGIKYYKNLINLLVENG-------------------IEPFVTIFHWDTPQALVDK---Y-GGFLD-E---RIV-------KDYTDFAKVCFENFGDKVN-
ZmGLU6           DGINYYKKLINLLLAEG-------------------IEPFITIFHWDTPQALVDK---Y-GGFLD-R---RIV-------KDYTDFAMVCFENFGDKVK-
ZmGLU7           KGIEYYKRLINLLKENG-------------------IEPYVTLFHWDTPQALVDS---Y-GGFLD-D---RIV-------KDYTDFAKVCFVHFGDTVK-
ZmGLU8           KGIEYYKKLINLLKENG-------------------IEPYVTLFHWDTPQALVDS---Y-GGFLD-D---RIV-------KDYTDFAKVCFVHFGDVVK-
```

```
                       1410      1420      1430      1440      1450      1460      1470      1480      1490      1500
                 ....|....|....|....|....|....|....|....|....|....|....|....|....|....|....|....|....|....|....|....|
AC155376.2_FGT005 --------------------RWI------TINEPLQTAINGYGI-GIFAPGGCQG---------------------------ETA-------RCY-LAAH
AC211140.2_FGT007 --------------------HWI------TLNEPHTVAVQGYDA-GLHAPGRCSVLLH--------L--YC-R--T----GNSGT-------EPY-IVAH
AC217401.3_FGT001 --------------------HWF------TFNEPRIVALLGYDA-GSIPPQRCTKC----------------SA-G----GNSAT-------EPY-IVAH
AC217687.3_FGT004 --------------------HWF------TFNEPRIVALLGYDT-GSNPPQSCKIPYE-----------ISGIS-I----LRTSE-------VP---LKN
AtBglu1           --------------------FWT------TINEANIFTIGGYND-GNSPPGRCSFP-G--------R--NC--T-L----GNSST-------ETY-IVGH
AtBglu10          --------------------LWT------TINEATIFAIGSYDQ-GISPPGHCSPNKF--------I--NC--T-S----GNSST-------EPY-LAGH
AtBglu11          --------------------HWT------TINEVNVFALGGYDQ-GITPPARCSPPFG--------L--NC--T-K----GNSSI-------EPY-IAVH
AtBglu12          --------------------HWM------TLNEPLTVVQQGYVA-GVMAPGRCSKFT--------NP--NC--T-A----GNGAT-------EPY-IVGH
AtBglu13          --------------------HWM------TLNEPLTVVQQGYVA-GVMAPGRCSKFT--------NP--NC--T-A----GNGAT-------EPY-IVGH
AtBglu14          --------------------HWI------TLNEPLTVVQQGYVA-GVMAPGRCSKFT--------NP--NC--T-A----GNGAT-------EPY-IVGH
AtBglu15          --------------------HWM------TLNEPLTVVQQGYVA-GVMAPGRCSKFT--------NP--NC--T-D----GNGAT-------EPY-IVGH
AtBglu16          --------------------QWT------TLNEPYTMVHEGYIT-GQKAPGRCSNFY--------KP--DC--L-G----GDAAT-------EPY-IVGH
AtBglu17          --------------------EWI------TINEPNMFAVLGYNV-GNIAPGRCSSYV---------Q--NC--T-V----GNSAT-------EPY-LVAH
AtBglu18          --------------------HWI------TFNEPWVFSRAGYDN-GKKAPGRCSPYIPG--Y---GQ--HC--Q-D----GRSGY-------EAY-QVSH
AtBglu19          --------------------NWI------TFNEPWVFSRSGYDV-GKKAPGRCSPYVKE--F---GK--LC--Q-D----GRSGF-------EPY-VVSH
AtBglu2           --------------------FWT------TINEANVFTIGGYND-GTSPPGRCS---------------NC--S-S----GNSST-------ETY-IVGH
AtBglu20          --------------------HWI------TFNEPWVFSRAGYDI-GNKAPGRCSKYIKE--H---GE--MC--H-D----GRSGH-------EAY-IVSH
AtBglu21          --------------------NWI------TFNEPWVFAHAGYDL-GKKAPGRCSRYVP-------GC--ED--R-E----GQSGK-------EAY-LVSH
AtBglu22          --------------------NWI------TFNEPWVFAHAGYDV-GKKAPGRCSRYLK-------GC--ED--R-D----GRSGY-------EAY-LVSH
AtBglu23          --------------------HWI------TFNEPWVFSHAGYDV-GKKAPGRCSSYVN--------A--KC--Q-D----GRSGY-------EAY-LVTH
AtBglu24          --------------------HWI------TFNEPWVFAHAGYDV-GKKAPGRCSPYAKDETV---KG--DC--L-G----GRSGY-------EAY-LVSH
AtBglu25          --------------------HWV------TINEPYEFSRGGYET-GEKAPGRCSKYVN--------E--KC--V-A----GKSGH-------EVY-TVSH
AtBglu26          --------------------LWC------TMNEPWVYSVAGYDT-GRKAPGRCSKYVN--------G--AS--V-A----GMSGY-------EAY-IVSH
AtBglu27          --------------------LWV------TLNEPWVYSIGGYDT-GRKAPGRASKYMN--------E--AA--V-A----GESGL-------EVY-TVSH
AtBglu28          --------------------MWT------TINEPYVITVAGYDT-GNKAVGRCSKWVN--------S--KC--Q-G----GDSGT-------EPY-IASH
AtBglu29          --------------------MWT------TINEPYVISVAGYDT-GIKAVGRCSKWVN--------S--RC--Q-A----GDSAI-------EPY-IVSH
AtBglu3           --------------------FWT------TINEANIFTIGGYND-GITPPGRCSSP-G--------R--NC--S-S----GNSST-------EPY-IVGH
AtBglu30          --------------------MWT------TINEPYIMTVAGYDQ-GNKAAGRCSKWVN--------E--KC--Q-A----GDSST-------EPY-IVSH
AtBglu31          --------------------MWT------TFNEPYVYSVSGYDA-GNKAMGRCSKWVN--------S--LC--I-A----GDSGT-------EPY-LVSH
AtBglu32          --------------------MWT------TFNEPYVYSVSGYDA-GNKAIGRCSKWVN--------S--LC--I-A----GDSGT-------EPY-LVSH
AtBglu33          --------------------NWA------TFNEPSVYSVAGYSK-GKKAPGRCSKWQA--------P--KC--P-T----GDSSE-------EPY-IVAH
AtBglu34          --------------------FWI------TLNQPFSLATKGYGD-GSYPPGRCTGCE----------------L-G----GDSGV-------EPY-TVAH
AtBglu35          --------------------FWI------TLNQPLSLALKGYGN-GSYPPGRCTGCE----------------L-G----GDSGV-------EPY-TVAH
AtBglu36          --------------------FWI------TLNQPYSLAVKGYGD-GQYPPGRCTDCE----------------F-G----GDSGT-------EPY-IVGH
AtBglu37          --------------------HWI------TINQLFTVPTRGYAL-GTDAPGRCSQWVD--------K--RC--Y-G----GDSST-------EPY-IVAH
AtBglu38          --------------------NWI------TINQLYTVPTRGYAL-GTDAPGRCSPKID--------V--RC--P-G----GNSST-------EPY-IVAH
AtBglu39          --------------------KWI------TINQLYTVPTRGYAM-GTDAP------------------------------------------EPY-IVAH
AtBglu4           --------------------LWT------TINEANIFSIGGYND-GDTPPGRCSKP-S--------K--NC--S-S----GNSSI-------EPY-IVGH
AtBglu40          --------------------HWI------TFNEPHTFAIQGYDV-GLQAPGRCTILFK--------L--TC-R--E----GNSST-------EPY-IVGH
AtBglu41          --------------------YWI------TFNEPHGVSIQGYDT-GIQAPGRCSLLGH--------W--FC-K--K----GKSSV-------EPY-IVAH
AtBglu42          --------------------HWI------TLNEPLQTSVNGHCI-GIFAPGRNEK---------------------------PLI-------EPY-LVSH
AtBglu43          --------------------NWM------TFNEPRVVAALGYDN-GIFAPGRCSEAFG-----------NC-T--D----GNSAT-------EPY-IVAH
AtBglu44          --------------------NWM------TFNEPRVVAALGYDN-GIFAPGRCSKAFG-----------NC-T--E----GNSAT-------EPY-IVTH
AtBglu45          --------------------YWT------TLNEPNQQLILGYLT-GKFPPSRCSSPYG-----------NC--S-Q----GNSET-------EPF-IAAH
AtBglu46          --------------------HWI------TINEPNQHISLAYRS-GLFPPARCSMPYG-----------NC--T-H----GNSET-------EPF-IAAH
AtBglu47          --------------------FWS------TFNEPNVQVILGYRT-GTYPPSRCSKPFG-----------NC--S-C----GDSYI-------EPL-VAAH
```

```
AtBglu5         --------------------FWT------TINEANVFTIGGYND-GDTPPGRCSLP-G--------K--NC--L-L----GNSST-------ETY-IVGH
AtBglu7         --------------------LWT------KINEATLFAIGSYGD-GMR-YGHCPPM-------------NY--S-T----ANVCT-------ETY-IAGH
AtBglu8         --------------------LWT------TINEATIFAFAFYGK-DVR-YG------------------NC--T-T----GNYCM-------ETY-IAGH
AtBglu9         --------------------LWT------TINEATIFAIGSYDQ-GTAPPGHCSPNKF--------V--NC--S-T----GNSST-------EPY-IAGH
BD1G10890       --------------------HWF------TFNEPRIVVLGGYDV-GSNPPQRCTKC----------------AA-G----GNSAT-------EPY-IVAH
BD1G10920       --------------------HWF------TFNEPRIVVLGGYDV-GSNPPQRCTKC----------------AA-G----GNSAT-------EPY-IVAH
BD1G10930       --------------------HWF------TLNEPRIACLLGYDV-GSTPPQRCTKC----------------AA-G----GNSAT-------EPY-IVAH
BD1G10940       --------------------NWF------TFNEPRIVAALGFDT-GIDPPNRCTKC----------------AA-G----GNSAT-------EPY-TVVH
BD1G19270       --------------------NWF------TFNEPRCVAALGYDN-GYHAPGRCSQ---------------C-TA-G----GNSMT-------EPY-LVAH
BD1G33040       --------------------HWI------TVNEPHNFAIDGYDF-GIQAPGRCSIMSH--------L--FC-K--D----GRSST-------EPY-IVAH
BD1G42690       --------------------YWI------TLNEPWSFSVGGYSS-GILAPGRCSSRQ--------KS--GC--S-M----GDSGK-------EPY-IVAH
BD1G70170       --------------------HWI------TVNEPHTVSVQGYDA-GLQAPGRCSVLLH--------L--YC-K--S----GNSGT-------EPY-IVAH
BD2G09190       --------------------NWT------TLNEPNALVALGYDS-GIGPPGRCSKPFG-----------DC--S-R----GNSVD-------EPY-IVAH
BD2G09200       --------------------NWT------TLNEPNALVSLGYDA-GIGPPGRCSKPFG-----------DC--S-C----GNSVD-------EPY-IVAH
BD2G27770       --------------------HWT------TMDEPNVISIAAYDS-GAFPPCRCSAPFG--------I--NC--T-V----GDSTV-------EPY-VVAH
BD2G37450       SGRVFVHCCQGVSRSTSLVIAYLMWREGQSFDDAFQFVKA--AR-GIANPNMGFAC-Q-------L------LQ-C-------------------QKRVH
BD2G57640       --------------------HWM------TINEPLQTSVNGYGI-GIFAPGVCEG---------------------------AAA-------EPF-LAAH
BD2G59650       --------------------YWT------TIDEPNVGVIGSYDT-GIFAPGHCSDPFG-------LI--EC--I-V----GDSTV-------EPY-VAAH
BD2G59660       --------------------YWT------TIDEPNVAAVGSYDT-AQIAPGRCSDPFG-------ST--KC--T-A----GDSTV-------EPY-VAAH
BD3G00650       --------------------HWI------TFNEPHTVTVQGYDS-GIHAPGRCSVLRH--------L--YC-K--Q----GSSGT-------EPY-IVAH
BD3G40000       --------------------LWN------TFNEPMIFCSGGYAT-GIAAPGRCSPYV--------SK--TC--G-A----GNSAT-------EPY-IAGH
BD3G40010       --------------------LWN------TFNEPTVFCMNGYGT-GIMAPGRCSDA----------S--SC--A-A----GDSGT-------EPY-TAAH
BD3G45610       --------------------YWT------TVNEPNIGAIASYSI-GHLPPGRCSDPFG-------IT--KC--T-V----GNSST-------EPY-IAVH
BD3G45630       --------------------YWT------TVNEPNIGAIAAYGS-GQLPPGRCSDPFG-------IT--KC--T-A----GNSSI-------EPY-IAVH
BD3G45640       --------------------YWT------TVNEPNIGAIAAYGS-GQLPPGRCSDPFG-------IT--KC--T-A----GNSST-------EPY-IAVH
BD3G45650       --------------------YWT------TVNELNGCAIASYGS-GQFPPGRCSDPFG-------IT--QC--A-G----GNSST-------EPY-IAVH
BD4G08040       --------------------NWF------TINEPRMMASHGYGD-GFFAPGRCTGC----------------RF-G----GNSAT-------EPY-ITGH
BD4G09920       --------------------YWV------VFNEPHVFVMLTYCA-GSWPGGDPNAL-E-------V------AT-S-----ALPT-------GVYNQALH
BD4G34930       --------------------YWS------TVNEPNVEPIGGYDQ-GILPPRRCSFPFGT-------L--SC--D-Q----GNSTT-------EPY-IVAH
BD4G34940       --------------------HWV------TVNEPNIEPIGGYDT-GFQPPRRCSYPFG--------V--DC--A-G----GNSST-------EPY-IAAH
BD4G34950       --------------------HWV------TVNEPNIETIGSFDS-GELPPRRCSYPFG--------V--NC--T-G----GNSTT-------EPY-IAAH
BD5G13260       --------------------HWI------TFNEPWSFCVTGYAR-GVFPPGRCSPWE--------KG--NC--S-S----GDSGR-------EPY-TVCH
BD5G13270       --------------------HWI------TFNEPWAFSVGAYAM-GVLAPGRCSPWE--------LG--KC--D-A----GDSGR-------EPY-IAAH
BD5G15530       --------------------FWT------TINEPNLLTKFSYMD-GWYPPCRCSKPFG-----------NC--A-F----GNSSI-------EPY-IVAH
BD5G15540       --------------------YWT------TFNEPNLFTKFAYLL-GEYPPNHCSPPFG-----------AC--N-S----GNSRR-------EPY-VAAH
GRMZM2G012236   --------------------HWV------TFNEPNLLAKLEYFI-GGFPPNRCSEPNG-----------KC--D-Y----GNSST-------EPY-IAAH
GRMZM2G015804   --------------------HWI------TLNEPHTVAVQGYDA-GLHAPGRCSVLLH--------L--YC-R--T----GNSGT-------EPY-IVAH
GRMZM2G031660   --------------------HWA------TFNEPNVAVTRGYML-GTYPPGRCSR--------------SC--A-R----GNSDA-------EPY-VAAH
GRMZM2G055699   --------------------HWT------TMDEPNVNSIAAYDN-GAFPPGRCSPPFGF------GT--NC--TAG----GNSSV-------EPY-VVTH
GRMZM2G069024   --------------------HWT------TLDEVNVAAIGSYDN-GQIAPGRCSDPFG-------TK--KC--T-V----GNSSV-------EPY-IAAH
GRMZM2G108133   --------------------YWS------TVNEPNVETIGGYDQ-GILPPRRCSFPFG--------F--GC--E-E----GNSTT-------EPY-VAAH
GRMZM2G110948   --------------------NWL------TFNEPRVVAALGYDD-GRFAPGRCTG---------------C-EA-G----GDSGT-------EPY-VVAH
GRMZM2G112704   --------------------NWF------TINEPRMMAQHGYGD-GFFPPARCTGC----------------QF-G----GNSAT-------EPY-IAGH
GRMZM2G128197   --------------------NWF------TLNEPRIVAFLGYDK-GLNPPNRCTQC----------------TA-G----GNSST-------EPY-IVVH
GRMZM2G148176   --------------------NWF------TFNEPRCVAALGYDN-GLHAPGRCSG---------------C-PA-G----GNSTT-------EPY-LVAH
GRMZM2G163544   --------------------HWI------TFNEPHNFAIEGYDL-GIQAPGRCSILSH--------I--FC-R--E----GKSST-------EPY-VVAH
GRMZM2G177661   --------------------HWV------TLNEPHTVAVQGYDA-GLQAPGRCSLLLH--------L--YC-R--S----GDSAT-------EPY-VVAH
GRMZM2G376416   --------------------HWI------TVNEPNIEPIGGYDQ-GYLPPRRCSYPFGL------GV--TC--T-H----GNSTT-------EPY-AVAH
```

```
GRMZM2G457040  ---------------------LWT------TFNEPNLLVKFQYML-GAYPPSRCSPPFG-----------SC--G-S----GDSRR-------EPY-AAAH
Os10bglu34     ---------------------HWI------TFNEPHTVAVQAYDS-GMHAPGRCSVLLH--------L--YC-K--K----GNSGT-------EPY-IVAH
Os11bglu36     ---------------------YWV------IFNEPHVFVMLTYCA-GAWPGGDPNAI-E-------V------AT-S-----TLPT-------GVYNQALH
Os12bglu38     ---------------------NWF------TINEPRMMANHGYGD-GFFPPGRCTGC----------------QP-G----GNSAT-------EPY-IAAH
Os1bglu1       ---------------------NWM------TFNEPRVVAALGYDD-GNFAPGRCTK---------------C-T--A----GNSAT-------EPY-IVAH
Os1bglu2       ---------------------HWT------TLAEPNIAALGGYDT-GVLSPGHCSDPFG-------LT--EC--T-V----GNSTV-------EPY-ITAH
Os1bglu3       ---------------------HWT------ILAEPNVAALGGYDT-GEFAPGRCSDPFG-------VT--KC--T-V----GNSSV-------EPY-VAAH
Os1bglu4       ---------------------HWI------TINEPLQTAVNGYGI-GHFAPGGCEG---------------------------ETA-------RCY-LAAH
Os1bglu5       ---------------------HWI------TIDEPNVASIGSYDS-GQLAPGRCSDPFG-------IR--KC--T-V----GNSSV-------EPY-IAVH
Os3bglu6       ---------------------HWI------TLNEPHTVAIQGYDA-GLQAPGRCSVLLH--------L--YC-K--A----GNSGT-------EPY-VVAH
Os3bglu7       ---------------------HWF------TFNEPRIVALLGYDQ-GTNPPKRCTKC----------------AA-G----GNSAT-------EPY-IVAH
Os3bglu8       ---------------------NWF------TFNEPRIVAALGHDT-GTDPPNRCTKC----------------AA-G----GNSAT-------EPY-IVAH
Os4bglu10      ---------------------HWI------TFNEPWNFCSMGYAS-GTMAPGRCSSWE--------KG--KC--R-V----GDSGR-------EPY-TACH
Os4bglu11      ---------------------HWI------TFNEPWTFCSMGYAS-GIMAPGRCSSWE--------ARRRNC--I-LHVTYRDSSRQVAARQKLPA--ALC
Os4bglu12      ---------------------NWI------TFNEPWTFCSNGYAT-GLFAPGRCSPWE--------KG--NC--S-V----GDSGR-------EPY-TACH
Os4bglu13      ---------------------HWI------TFNEPLSFCVAGYASGGMFAPGRCSPWE---------G--NC--S-A----GDSGR-------EPY-TACH
Os4bglu14      ---------------------FWT------TFNQPNLSIKFSYMD-GFYSPGRCSEPFG-----------KC--A-L----GNSSI-------EPY-VAGH
Os4bglu16      ---------------------FWT------TFNEPNLSTRHQYIL-GEFPPNHCSPPFG-----------NC--S-S----GDSRR-------EPY-AAAH
Os4bglu18      ---------------------YWT------TFNEPNVAVRHGYML-GTYPPSRCSPPFG-----------HC-AR-G----GDSHA-------EPY-VAAH
Os4bglu27      ---------------------SWN------TFNEPMIFCAGGYGS-GTKAPGRCSPYV--------SK--KC--A-P----GDSGN-------EPY-VAGH
Os4bglu28      ---------------------LWN------TFNEPTIFCMNGYAT-GIMAPGRCSPYA--------SA--SC--A-AG---GDSGR-------EPY-VAGH
Os4bglu9       ---------------------HWI------TFNEPWIFCSKAYAS-GTYAPGRCSPWE--------MG--KC--S-V----GDSGR-------EPY-TACH
Os5bglu19      ---------------------HWT------TSIEPNVMAQSGYDD-GYLPPNRCSYPFG-------RS--NC--T-L----GNSTV-------EPY-LFIH
Os5bglu21      ---------------------HWT------TVLEPNAMAQAGYDM-GILPPNRCSYPFG--------S--NC--T-A----GNSSV-------EPY-LFIH
Os5bglu22      ---------------------HWT------TMDEPNVLSIAAYDS-GAFPPCRCSPPFG--------A--NC--T-A----GNSTV-------EPY-VVAH
Os5bglu23      ---------------------HWT------TVGEPNVLSIAGYDS-GVIPPCRCSPPFG--------T--SC--A-A----GDSTV-------EPY-FAAH
Os5gblu20      ---------------------HWT------TVLEPNVMAQGCYDT-GILPPNHCSYPFG--------N--NC--T-G----GNSTV-------EPY-LFIH
Os6bglu24      ---------------------YWI------TFNEPWSFSIGGYSN-GILAPGRCSSQG--------KS--GC--S-K----GDSGR-------EPY-IVAH
Os6bglu25      ---------------------HWI------TFNEPYNFAIDGYDL-GIQAPGRCSILSH--------V--FC-R--E----GKSST-------EPY-IVAH
Os7bglu26      ---------------------DWF------TFNEPRCVAALGYDN-GFHAPGRCSG---------------C-DA-G----GNSTT-------EPY-LAAH
Os9bglu29      ---------------------FWA------TFNEPWTYCSQGYGT-GIHALGRCSPYV--------ST--SC--A-G----GDSSR-------EPY-LAAH
Os9bglu30      ---------------------YWT------TFNEPFTYSAYGYGK-GVFAPGRCSSYV--------SK--SC--G-V----GDSSR-------EPY-LVAH
Os9bglu31      ---------------------HWS------TVNEPNIEPIGGYDQ-GILPPRRCSFPFGV-------L--SC--D-N----GNSTT-------EPY-IVAH
Os9bglu32      ---------------------HWV------TVNEPNIEPIGGYDA-GVQPPRRCSYPFG--------T--NC--T-G----GDSST-------EPY-IVAH
Os9bglu33      ---------------------HWA------TFNQPNIEPIGGFDA-GDRPPRRCSYPFG--------T--NC--T-G----GDSST-------EPY-IVAH
SB01G010825    ---------------------NWF------TLNEPRIVSFLGYDK-GIDPPNRCTQC----------------TA-G----GNSST-------EPY-IVVH
SB01G010830    ---------------------HWF------TFNEPRIVALLGYDG-GSIPPQRCTKC----------------AA-G----GNSAT-------EPY-IVAH
SB01G013360    ---------------------HWI------TFNEPHTVTVQGYDS-GLQAPGRCSLILH--------L--YC-K--E----GNSGT-------EPY-IVAH
SB01G043030    ---------------------HWI------TLNEPHTVAIQGYDA-GLHAPGRCSVLLH--------L--YC-K--S----GNSGT-------EPY-IVAH
SB02G028400    ---------------------AWT------TFNEPWTYSYQGYAV-GKSAPGRCSSYV--------NK--NC--F-P----GDSAR-------EPY-TVTH
SB02G029620    ---------------------YWT------TVNEPNIETVGGYDE-GILPPRRCSSPFG--------F--PC--N-G----GNSTT-------EPY-IAAH
SB02G041550    ---------------------NWF------TFNEPRCVAALGYDN-GLHAPGRCSE---------------C-AA-G----GNSTT-------EPY-LAAH
SB03G037780    ---------------------HWT------TLDEVNVAALGSYDI-GQIAPGRCSDPFGF--G---TK--KC--T-V----GNSSV-------EPY-IAAH
SB03G042690    ---------------------RWI------TINEPLQTAINGYGI-GIFAPGGCQG---------------------------ETA-------RCY-LAAH
SB06G019830    ---------------------HWI------TFNEPWTFCSVGYAS-GTFPPARCSSWE--------EG--KC--N-V----GDSGR-------EPY-TACH
SB06G019840    ---------------------HWI------TFNEPLSFCSTGYAW-GTFAPGRCSPWE--------QG--KC--S-I----GDSGR-------EPY-TACH
SB06G019850    ---------------------HWI------TFNEPWSFCSGGYAS-GTFAPGRCSPWE--------QG--KC--S-A----GDSGT-------EPY-TVCH
SB06G019860    ---------------------HWI------TFNEPWSFCSSGYAS-GTIAPGRCSPWE--------QG--KC--S-A----GDSGT-------EPY-TVCH
```

```
SB06G019880    --------------------HWI------TFNEPAQFCSIGYAS-GIGAPGRCSPWE--------QG--KC--S-A----GDSGR-------EPY-TVCH
SB06G022410    --------------------HWA------TFNEPNLMAKLAYFN-GKFPPSHCSKPFG-----------KC--N-S----GNSST-------EPY-IAAH
SB06G022420    --------------------HWA------TFNEANFLTKLKYSM-GKFPPSHCSEPYG-----------KC--N-S----GNSST-------EPY-IAAH
SB06G022450    --------------------FWI------TFNEPNIFAKLSYIY-GRYPPGHCSRPFG-----------NC--T-S----GNSST-------EPY-IVGH
SB06G022460    --------------------FWI------TFNEPNMFTKLGYIY-GRFPPGHCSRPFG-----------NC--T-F----GNSST-------EPY-IAGH
SB06G022490    --------------------FWT------TFNEPNLSTKFQYML-GVYPPRHCSPPFG-----------SC--N-S----GNSHR-------EPY-VAAH
SB06G022500    --------------------HWA------TFNEPNVAMTKGYML-GTYPPGRCSPPFG-----------SC--A-Q----GNSDA-------EPY-VATH
SB06G022510    --------------------YWS------TFNEPNVVVTRGYMV-GTYPPERCSPPLG-----------SC--A-R----GNSDA-------EPY-VATH
SB08G007586    --------------------NWF------TFNEPETFCSVSYGT-GVLAPGRCSPGV--------SC--AV--P-T----GNSLT-------EPY-IVAH
SB08G007650    --------------------NWF------TFNEPETFCSVSYGT-GVLAPGRCSPGV--------NC--AV--P-T----GNSLT-------EPY-TVAH
SB09G018160    --------------------HWT------TMMEPNIIAQGSYDI-GIVAPGRCSYPFG--------H--DC--T-A----GNSTV-------EPY-LFLH
SB10G012220    --------------------HWI------TFNEPWSFSINGYAS-GILAPGRCSAWE--------NS--GC--S-T----GDSGR-------EPY-IVAH
SB10G022300    --------------------HWI------TVNEPNIEPLGGYDQ-GYLPPRRCSAPFGL--A---GV--PC--T-H----GNSTT-------EPY-VVAH
SB10G027600    --------------------HWI------TFNEPHNFAIDGYDL-GIQAPGRCSILSH--------I--FC-R--E----GKSST-------EPY-VVAH
SB10G028060    --------------------FWF------TINEPQMVASHGYGD-AFFPPGRCTGC----------------YF-G----GNSAT-------EPY-IAGH
SbDhr1         --------------------NWL------TFNEPETFCSVSYGT-GVLAPGRCSPGV--------SC--AV--P-T----GNSLS-------EPY-IVAH
SbDhr2         --------------------NWL------TFNEPHTFTCLSYGT-GILAPGRCSPGM--------KC--PD--P-T----GDSIR-------EPY-LVGH
Zm-p60.1       --------------------NWL------TFNEPQTFTSFSYGT-GVFAPGRCSPGL--------DC--AY--P-T----GNSLV-------EPY-TAGH
ZmGLU2         --------------------NWL------TFNEPQTFTSFSYGT-GVFAPGRCSPGL--------DC--AI--P-T----GNSLV-------EPY-IAGH
ZmGLU3         --------------------NWL------TFNEPQTFTTFSYGT-GVFAPGRCSPGE--------KC--AQ--P-I----ANSLT-------EPY-IAGH
ZmGLU4         --------------------NWF------TFNEPETFCTFSHGT-GQCAPGRCSPGIIT-PTGSTSC--AN--P-I----GNSLT-------EPY-IVGH
ZmGLU5         --------------------NWL------TFNEPQTFSSFSYGT-GLCAPGRCTPGQ--------KC--AN--P-I----GNSLT-------EPY-TVGH
ZmGLU6         --------------------NWL------TFNEPQTFSSFSYGI-GLCAPGRCSPGQ--------KC--AN--P-I----GNSLI-------EPY-IVGH
ZmGLU7         --------------------NWF------TFNEPQTFSSFSYGT-GIFAPGRCSPGQ--------KC--AN--P-T----GNSLT-------EPY-IVAH
ZmGLU8         --------------------NWF------TFNEPQTFSSFSYGT-GICAPGRCSPGQ--------KC--AN--P-V----GNSLT-------EPY-IVGH
```

```
                         1510      1520      1530      1540      1550      1560      1570      1580      1590      1600
                  ....|....|....|....|....|....|....|....|....|....|....|....|....|....|....|....|....|....|....|....|
AC155376.2_FGT005 HQILAHAAAVD-VYR-R--------------------------------------------------------------KFK---------------A--
AC211140.2_FGT007 NFILAHATVSD-MYR-R--------------------------------------------------------------KYK---------------A--
AC217401.3_FGT001 NFLLSHAAAVS-RYR-N--------------------------------------------------------------KYQ---------------A--
AC217687.3_FGT004 NITLALLG-------------------------------------------------------------------------V---------------A--
AtBglu1           NLLLAHASVSR-LYK-Q--------------------------------------------------------------KYK---------------D--
AtBglu10          NILLAHASASK-LYK-L--------------------------------------------------------------KYK---------------S--
AtBglu11          NMLLAHASATI-LYK-Q--------------------------------------------------------------QYK---------------VLL
AtBglu12          NLILAHGEAVK-VYR-E--------------------------------------------------------------KYK---------------A--
AtBglu13          NLILAHGEAVK-VYR-E--------------------------------------------------------------KYK---------------A--
AtBglu14          NLILAHGEAIK-VYR-K--------------------------------------------------------------KYK---------------A--
AtBglu15          NLILSHGAAVQ-VYR-E--------------------------------------------------------------KYK---------------A--
AtBglu16          NLLLAHGVAVK-VYR-E--------------------------------------------------------------KYQ---------------A--
AtBglu17          YLILSHAATVQ-LYR-E--------------------------------------------------------------KYQ---------------S--
AtBglu18          NLLLSHAYAVD-AFR-N--------------------------------------------------------------CK----------------Q--
AtBglu19          NLLVGHAEAVD-AFR-K--------------------------------------------------------------CE----------------K--
AtBglu2           NLLLAHASVSR-LYQ-Q--------------------------------------------------------------KYK---------------D--
AtBglu20          NMLLAHADAVD-AFR-K--------------------------------------------------------------CD----------------K--
AtBglu21          NLLNAHAEAVE-VFR-Q--------------------------------------------------------------KV-------------------
AtBglu22          NLLNAHAEAVE-VFR-Q--------------------------------------------------------------KV-------------------
AtBglu23          NLLISHAEAVE-AYR-K--------------------------------------------------------------CE----------------K--
AtBglu24          NLLNAHAEAVE-AFR-Q--------------------------------------------------------------CE----------------K--
AtBglu25          NLLLAHAEAVE-EFR-K--------------------------------------------------------------CG----------------K--
AtBglu26          NMLLAHAEAVE-VFR-K--------------------------------------------------------------CD----------------H--
AtBglu27          NLLLAHAEAVE-VFR-N--------------------------------------------------------------NP----------------K--
AtBglu28          HLLLAHAAAVQ-EFR-K--------------------------------------------------------------C-N---------------K--
AtBglu29          HLLLSHAAAVQ-EFR-N--------------------------------------------------------------CNK---------------T--
AtBglu3           NLLLAHASASR-LYK-Q--------------------------------------------------------------KYK---------------D--
AtBglu30          HTLLAHAAAVE-EFR-K--------------------------------------------------------------CEK---------------T--
AtBglu31          HLLLAHAAAVE-EFR-K--------------------------------------------------------------CDK---------------I--
AtBglu32          NLLLAHAAAVE-EFR-K--------------------------------------------------------------CDK---------------I--
AtBglu33          NQILAHLAAVD-EFR-N--------------------------------------------------------------CKK---------------V--
AtBglu34          NQLLAHAKTVS-LYR-K--------------------------------------------------------------RYQ---------------K--
AtBglu35          NQLLAHAKTVS-LYR-K--------------------------------------------------------------RYQ---------------K--
AtBglu36          HELLAHMEAVS-LYR-K--------------------------------------------------------------RYQ---------------K--
AtBglu37          NQLLAHATVVD-LYR-T--------------------------------------------------------------RYK------------------
AtBglu38          NQLLAHAAAVD-VYR-T--------------------------------------------------------------KYK---------------D--
AtBglu39          NQLLAHAKVVH-LYR-K--------------------------------------------------------------KYK---------------P--
AtBglu4           NLLLAHASVSR-RYK-Q--------------------------------------------------------------KYK---------------D--
AtBglu40          NVILTHATVSD-IYR-K--------------------------------------------------------------KYK---------------A--
AtBglu41          NILLSHAAAYH-TYQ-R--------------------------------------------------------------NFK---------------E--
AtBglu42          HQVLAHATAVS-IYR-S--------------------------------------------------------------KYK---------------E--
AtBglu43          HLILAHAAAVQ-RYR-Q--------------------------------------------------------------NYQ---------------E--
AtBglu44          HLILAHAAAVQ-RYR-K--------------------------------------------------------------YYQ---------------A--
AtBglu45          NMILAHAKAVN-IYK-T--------------------------------------------------------------KYQ---------------K--
AtBglu46          NMILAHAKAIQ-IYR-T--------------------------------------------------------------KYQ---------------R--
AtBglu47          NIILSHLAAVN-LYR-T--------------------------------------------------------------KFQ---------------E--
```

```
AtBglu5         NLLLAHASASR-LYK-Q--------------------------------------------------------------KYK---------------D--
AtBglu7         NMLLAHSSASN-LYK-L--------------------------------------------------------------KYK---------------T--
AtBglu8         NMLLAHASASN-LYK-L--------------------------------------------------------------KYK---------------S--
AtBglu9         NILLAHASASK-LYK-L--------------------------------------------------------------KYK---------------S--
BD1G10890       NFILAHGYAVA-RYR-N--------------------------------------------------------------KYK---------------A--
BD1G10920       NFILAHGYAVA-RYR-N--------------------------------------------------------------KYK---------------A--
BD1G10930       NFLLAHGYAVA-RYR-N--------------------------------------------------------------KYQ---------------A--
BD1G10940       NILLSHATAVA-RYR-N--------------------------------------------------------------KYQ---------------A--
BD1G19270       HLILSHAAAVK-RYR-E--------------------------------------------------------------KYQ---------------H--
BD1G33040       NILLAHAGAFH-TYK-Q--------------------------------------------------------------HFK---------------K--
BD1G42690       NQLLAHASAVQ-VYR-D--------------------------------------------------------------KYQ---------------M--
BD1G70170       NFILAHATVSR-IYR-N--------------------------------------------------------------KYR---------------A--
BD2G09190       NCLLAHSSAVS-LYK-R--------------------------------------------------------------KYQ---------------A--
BD2G09200       NCLLAHSSAVS-LYR-R--------------------------------------------------------------KYQ---------------A--
BD2G27770       HSILAHASVVR-LYH-Q--------------------------------------------------------------KYR---------------A--
BD2G37450       AIPLSPNSVLR-MYR-MAPHSPYAPLHLVPKMLNEPSPAALDSRGAFIVHIASLIYVWVGLRCDQVMEKDARAAAFQVVRYE---------------K--
BD2G57640       HQILAHAASVD-VYR-R--------------------------------------------------------------KFK---------------A--
BD2G59650       NMILAHASATR-LYR-K--------------------------------------------------------------KYQ---------------A--
BD2G59660       NMILAHASATR-LYR-G--------------------------------------------------------------KYQ---------------A--
BD3G00650       NIILAHATVSD-MYR-T--------------------------------------------------------------KYK---------------A--
BD3G40000       NLLLAHAEAVE-LYR-T--------------------------------------------------------------KYQ---------------K--
BD3G40010       TLLLAHAQAVK-LYR-T--------------------------------------------------------------KYQ---------------Q--
BD3G45610       TTLLAHASVFK-LYR-E--------------------------------------------------------------KYK---------------A--
BD3G45630       TTLLAHTSVVK-LYR-E--------------------------------------------------------------KYKRYRLQVDSLKKLLEQA--
BD3G45640       TTLLAHASVVK-LYR-E--------------------------------------------------------------KYK---------------A--
BD3G45650       TTLLAHASVVK-LYR-E--------------------------------------------------------------KYK---------------A--
BD4G08040       HLLLSHAAAVK-IYR-D--------------------------------------------------------------KYQ---------------A--
BD4G09920       WMAVAHAEAYD-YIH-S--------------------------------------------------------------ESN---------------N--
BD4G34930       HLLLAHASAAS-LYK-E--------------------------------------------------------------KYQ---------------A--
BD4G34940       HLLLAHASAVS-LYR-E--------------------------------------------------------------KYK---------------E--
BD4G34950       RLLLAHASAVS-LYR-D--------------------------------------------------------------KYQ---------------G--
BD5G13260       HQLLAHAATAR-LYK-A--------------------------------------------------------------KYE---------------A--
BD5G13270       HQILAHASAVR-IYK-E--------------------------------------------------------------KYQ---------------A--
BD5G15530       NMILSHANAVS-IYR-N--------------------------------------------------------------NYQ---------------G--
BD5G15540       NILLSHAAAVN-NYK-K--------------------------------------------------------------NYQ---------------A--
GRMZM2G012236   NMILAHAKTNN-IYR-K--------------------------------------------------------------NYK---------------S--
GRMZM2G015804   NFILAHATVSD-MYR-R--------------------------------------------------------------KYK---------------A--
GRMZM2G031660   NVVLAHAAAVQ-IYK-T--------------------------------------------------------------KYQ---------------S--
GRMZM2G055699   NCILAHAAVAA-LYT-R--------------------------------------------------------------SYR---------------A--
GRMZM2G069024   NMLLAHASATR-LYR-E--------------------------------------------------------------KYQ---------------A--
GRMZM2G108133   HLLLAHASAVS-LYR-D--------------------------------------------------------------RYQ---------------A--
GRMZM2G110948   HLILSHAAAVQ-RYR-R--------------------------------------------------------------RHQ---------------P--
GRMZM2G112704   HLLLAHAAAVK-LYR-S--------------------------------------------------------------KYK---------------V--
GRMZM2G128197   NILLSHATAVA-RYR-N--------------------------------------------------------------KYQ---------------A--
GRMZM2G148176   HLILSHAAAVR-RYR-D--------------------------------------------------------------KYQ---------------L--
GRMZM2G163544   NILLAHAGAFH-TYK-Q--------------------------------------------------------------HFK---------------K--
GRMZM2G177661   NFILAHAKVSD-VYR-K--------------------------------------------------------------KYK---------------A--
GRMZM2G376416   HLLLAHASAVS-LYR-R--------------------------------------------------------------KYQ---------------G--
```

```
GRMZM2G457040    NIIMSHAAAVR-AYR-E--------------------------------------------------------------KYQ---------------A--
Os10bglu34       NMILSHATVSD-IYR-K--------------------------------------------------------------KYK---------------A--
Os11bglu36       WMAIAHSEAYD-YIH-S--------------------------------------------------------------KSK---------------N--
Os12bglu38       NLLLSHAAAVR-TYR-D--------------------------------------------------------------KYQ---------------A--
Os1bglu1         HLILSHASAVQ-RYR-H--------------------------------------------------------------KYQ---------------H--
Os1bglu2         NMILTHAAVVR-LYR-E--------------------------------------------------------------KYQ---------------A--
Os1bglu3         NMILTHAAVVR-LYR-E--------------------------------------------------------------KYQ---------------T--
Os1bglu4         YQILAHAAAVD-VYR-R--------------------------------------------------------------KFK---------------A--
Os1bglu5         NMLLAHASVTK-LYR-E--------------------------------------------------------------KYQ---------------V--
Os3bglu6         HFILAHAAAAS-IYR-T--------------------------------------------------------------KYK---------------A--
Os3bglu7         NFLLSHAAAVA-RYR-T--------------------------------------------------------------KYQ---------------A--
Os3bglu8         NIILSHATAVD-RYR-N--------------------------------------------------------------KFQ---------------A--
Os4bglu10        HQLLAHAETVR-LYK-E--------------------------------------------------------------KYQ---------------F--
Os4bglu11        NLCLDQTAILCNVRLVA--------------------------------------------------------------FQQ---------------A--
Os4bglu12        HQLLAHAETVR-LYK-A--------------------------------------------------------------KYQ---------------A--
Os4bglu13        HQLLAHAETVR-LYK-E--------------------------------------------------------------KYQ---------------V--
Os4bglu14        NIILSHANAVS-VYR-N--------------------------------------------------------------KYQ---------------G--
Os4bglu16        NILLSHAAAVH-NYK-T--------------------------------------------------------------NYQ---------------P--
Os4bglu18        NVILSHATAIE-IYK-R--------------------------------------------------------------KYQ---------------S--
Os4bglu27        NLLLAHAEAVR-LYR-Q--------------------------------------------------------------KYQ---------------A--
Os4bglu28        HLLVAHAEAVR-LYR-A--------------------------------------------------------------RYR---------------A--
Os4bglu9         HQLLAHAETVR-LYR-E--------------------------------------------------------------KYQ---------------F--
Os5bglu19        HTLLAHASAVR-LYR-E--------------------------------------------------------------KHQ---------------A--
Os5bglu21        HSLLAHASAVR-LYR-E--------------------------------------------------------------KYK---------------V--
Os5bglu22        NSILAHASVTR-LYR-D--------------------------------------------------------------KYQ---------------A--
Os5bglu23        NSILAHASAVR-LYW-D--------------------------------------------------------------KYQ---------------A--
Os5gblu20        HNLLAHASAVR-LYR-E--------------------------------------------------------------KYQ---------------V--
Os6bglu24        NQLLAHAAVVQ-IYR-E--------------------------------------------------------------KYQ---------------G--
Os6bglu25        NILLAHAGAFR-AYE-Q--------------------------------------------------------------HFK---------------N--
Os7bglu26        HLILSHAAAVK-RYR-E--------------------------------------------------------------KYQ---------------L--
Os9bglu29        HVILAHATAVH-LYR-T--------------------------------------------------------------KYQ---------------P--
Os9bglu30        HIHLSHAAAVQ-LYR-T--------------------------------------------------------------KYQ---------------P--
Os9bglu31        HLLLAHSSAVS-LYR-E--------------------------------------------------------------KYQ---------------A--
Os9bglu32        HLLLAHASAVS-IYR-Q--------------------------------------------------------------KYQ---------------A--
Os9bglu33        HLLLAHASAVS-IYR-Q--------------------------------------------------------------KYQ---------------A--
SB01G010825      NILLSHATAVA-RYR-N--------------------------------------------------------------KYQ---------------A--
SB01G010830      NFLLSHAAAVA-RYR-N--------------------------------------------------------------KYQ---------------A--
SB01G013360      NIILAHATVAD-IYM-N--------------------------------------------------------------KYK---------------A--
SB01G043030      NFILAHATVSD-IYR-R--------------------------------------------------------------KYK---------------A--
SB02G028400      NIILAHAEAVA-LYN-A--------------------------------------------------------------KYK---------------P--
SB02G029620      HLLLAHASAVS-LYR-E--------------------------------------------------------------KYQ------------------
SB02G041550      HLILSHAAAVR-RYR-D--------------------------------------------------------------KYQ---------------L--
SB03G037780      NMLLAHASATR-LYR-E--------------------------------------------------------------KYQ---------------A--
SB03G042690      HQILAHAAAVD-VYR-R--------------------------------------------------------------KFK---------------A--
SB06G019830      YQMLAHAETVR-LYK-E--------------------------------------------------------------KYQ---------------G--
SB06G019840      HQILAHAETVR-LYK-Q--------------------------------------------------------------KYQ---------------A--
SB06G019850      HQILAHAETVR-LYK-E--------------------------------------------------------------KYK---------------V--
SB06G019860      HQILAHAETVR-LYK-E--------------------------------------------------------------KYQ---------------V--
```

```
SB06G019880    HQLLAHGEAVR-LYK-E--------------------------------------------------------------KYQ---------------A--
SB06G022410    NMILAHAKTVN-IYK-K--------------------------------------------------------------NYK---------------T--
SB06G022420    NMILAHAMAVN-IYR-K--------------------------------------------------------------NYK---------------V--
SB06G022450    NMVLSHANVVS-IYK-E--------------------------------------------------------------KYQ---------------G--
SB06G022460    NIILSHANVVN-IYK-K--------------------------------------------------------------KYQ---------------G--
SB06G022490    NIIMSHAAAVR-NYK-E--------------------------------------------------------------SYQ---------------A--
SB06G022500    NVVLSHATAVE-IYK-R--------------------------------------------------------------KYQ---------------R--
SB06G022510    NVVLAHATAVE-IYK-R--------------------------------------------------------------KYQ---------------S--
SB08G007586    NLLRAHAETVD-LYN-K--------------------------------------------------------------YHK------------------
SB08G007650    HLLLAHAETVD-LYN-K--------------------------------------------------------------HHK------------------
SB09G018160    YNLLAHSSVVR-LYR-E--------------------------------------------------------------KYQ---------------A--
SB10G012220    NQLLAHAAAVQ-VYK-G--------------------------------------------------------------KYQ---------------E--
SB10G022300    HLLLAHASAVS-LYR-R--------------------------------------------------------------KYQ---------------G--
SB10G027600    NILLAHAGAFH-SYK-Q--------------------------------------------------------------HFK---------------K--
SB10G028060    HLLLSHAAAVK-LYR-E--------------------------------------------------------------KYK---------------V--
SbDhr1         NLLRAHAETVD-IYN-K--------------------------------------------------------------YHK------------------
SbDhr2         NFLLAHAETVD-LYN-K--------------------------------------------------------------FHR------------------
Zm-p60.1       NILLAHAEAVD-LYN-K--------------------------------------------------------------HYK---------------R--
ZmGLU2         NILLAHAEAVD-LYN-K--------------------------------------------------------------YYK---------------G--
ZmGLU3         NILRAHAMTVD-LYN-K--------------------------------------------------------------NYK---------------G--
ZmGLU4         NLLRAHAEVVD-LYN-K--------------------------------------------------------------HYK---------------I--
ZmGLU5         NLLRAHAEAVD-LYN-K--------------------------------------------------------------YYK---------------G--
ZmGLU6         NLLLAHAEAVD-LYN-K--------------------------------------------------------------HYK---------------D--
ZmGLU7         NLLRAHAETVH-EYN-K--------------------------------------------------------------HYR---------------G--
ZmGLU8         NLLRAHAETVH-QYN-K--------------------------------------------------------------YYR---------------G--
```

```
                                        1610      1620      1630      1640      1650      1660      1670      1680      1690      1700
                                ....|....|....|....|....|....|....|....|....|....|....|....|....|....|....|....|....|....|....|....|
AC155376.2_FGT005    ---------------------A---QSGEV-----GLVVDCEWAEPFS-E------K--L-EDQIAAQRRIDFQLGWYLDPIYFGDYPESMRQRL-GSDL
AC211140.2_FGT007    ---------------------A---QNGEL-----GIAFDVIWYEPMT-N------S--T-IDIEATKRAQEFQLGWFADPFFFGDYPATMRARV-GERL
AC217401.3_FGT001    ---------------------A---QKGKV-----GIVLDFNWYEALT-N------S--T-EDQAAAQRARDFHVGWFADPLINGHYPQIMQDIV-KERL
AC217687.3_FGT004    ---------------------A---QKGKV-----GIVLDFNWYEALT-N------S--P-DDQAAAQRARDFHIGWFVDPLINGHYPQIMQDLV-KERL
AtBglu1              ---------------------I---QGGSV-----GFSLFAMNFTPST-N------S--K-DDEIATKRANDFYLGWMLEPLIYGDYPDVMKRTI-GSRL
AtBglu10             ---------------------T---QKGSI-----GLSIFAFGLSPYT-N------S--K-DDEIATQRAKAFFYGWMLKPLVFGDYPDEMKRTV-GSRL
AtBglu11             SASLPSSICIAFCYVLFITQYK---QHGSV-----GISVYTYGAVPLT-N------S--V-KDKQATARVNDFYIGWILHPLVFGDYPETMKTNV-GSRL
AtBglu12             ---------------------S---QKGQV-----GIALNAGWNLPYS-E------S--A-EDRLAAARAMAFTFDYFMEPLVTGKYPIDMVNYVKGGRL
AtBglu13             ---------------------S---QKGQV-----GIALNAGWNLPYT-E------S--A-EDRLAAARAMAFTFDYFMEPLVTGKYPVDMVNNVKDGRL
AtBglu14             ---------------------S---QKGQV-----GIALNAGWNLPYT-E------S--A-EDRLAAARAMAFTFDYFMEPLVTGKYPVDMVNNVKGGRL
AtBglu15             ---------------------S---QQGQV-----GIALNAGWNLPYT-E------S--P-KDRLAAARAMAFTFDYFMEPLVTGKYPVDMVNNV-KGRL
AtBglu16             ---------------------T---QKGEI-----GIALNTAWHYPYS-D------S--Y-ADRLAATRATAFTFDYFMEPIVYGRYPIEMVSHVKDGRL
AtBglu17             ---------------------F---HGGTI-----GMTIQTYWMIPKY-N------T--P-ACREAAKRALDFFFGWFADPITYGDYPKTMRELV-GNRL
AtBglu18             ---------------------C---AGGKI-----GIAHSPAWFEPQD--L-----E--H-VG-GSIERVLDFILGWHLAPTTYGDYPQSMKDRV-GHRL
AtBglu19             ---------------------C---KGGKI-----GIAHSPAWFEPED--V-----E--G-GQ-ATVNRVLDFVIGWHLDPTTFGDYPQSMKDAV-GSRL
AtBglu2              ---------------------K---QGGSV-----GFSLYAFEFIPQT-S------S--SKDDEIAIQRAKDFFYGWILGPLTFGDYPDEMKRAV-GSRL
AtBglu20             ---------------------C---KGGKI-----GIAHSPAWFEAHE-LS-----D--E-EHETPVTGLIDFILGWHLHPTTYGDYPQSMKDHI-GHRL
AtBglu21             -------------------------KGGKI-----GIAHSPAWFEPHD-LK-----D--S-NDAPTVSRVLDFMLGWHLEPTTSGDYPQIMKDLL-GYRL
AtBglu22             -------------------------KGGKI-----GIAHSPAWFEPHD-LK-----D--S-NDVPTVSRVLDFMLGWHLDPTTFGDYPQIMKDLL-GHRL
AtBglu23             ---------------------C---KGGKI-----GIAHSPAWFEAHD-LA-----D--S-QDGASIDRALDFILGWHLDTTTFGDYPQIMKDIV-GHRL
AtBglu24             ---------------------C---KGGKI-----GIAHSPAWFEPHD-FK-----D--E-QSGATIDRALDFIMGWHLDTTMFGDYPQTMKDIV-GHRL
AtBglu25             ---------------------C---TGGKI-----GIVQSPMWFEPYD-KK---STS--S-PSEEIVKRAMDFTLGWHMEPITHGDYPQAMKDVV-GSRL
AtBglu26             ---------------------I---KNGQI-----GIAHNPLWYEPYD-PS-----D--P-DDVEGCNRAMDFMLGWHQHPTACGDYPETMKKSV-GDRL
AtBglu27             ---------------------C---KDGKI-----GIAHCPVWFEPYD-SN-----C--P-KDIEACERAMEFMFGWHMDPTVYGDYPAVMKKSI-GKRL
AtBglu28             ---------------------T---QDGQI-----GIVLSPLWFEPYD-SA-----S--P-ADNEAVKRALATELDWHLDPVIHGDYPEMMKKLA-GNRL
AtBglu29             ---------------------L---QDGKI-----GIVISPWWLEPYD-ST-----S--S-ADKEAVERGLPLELEWHLNPVIYGDYPETMKKHV-GNRL
AtBglu3              ---------------------M---QGGSV-----GFSLFSLGFTPST-S------S--K-DDDIAVQRAKDFYFGWMLEPFIFGDYPDEMKRTV-GSRL
AtBglu30             ---------------------S---HDGQI-----GIVLSPRWFEPYH-SD-----S--T-DDKEAAERALAFEIGWHLDPVIHGDYPEIVKKYA-GNKL
AtBglu31             ---------------------S---QDSKI-----GIVLSPYWFEPYD-SA-----S--N-ADKEAVERALAFNIGWHLSPLVFGDYPETIKISA-GNRL
AtBglu32             ---------------------S---QDAKI-----GIVLSPYWFEPYD-ID-----S--E-SDKEAVERALVFNIGWHLSPLVFGDYPETIKTTA-GNRL
AtBglu33             ---------------------E---GGGKI-----GIVLVSHWFEPKD-PN-----S--S-EDVKAARRSLEYQLGWFLRPLTYGQYPAEMLEDV-NIRL
AtBglu34             ---------------------F---QGGKI-----GTTLIGRWFAPLN-EF-----S--E-LDKAAAKRAFDFFVGWFLDPLVYGKYPTIMREMV-GDRL
AtBglu35             ---------------------F---QGGKI-----GTTLIGRWFVPLN-EF-----S--E-LDKAAAKRAFDFFVGWFLDPLVYGKYPTIMREMV-GDRL
AtBglu36             ---------------------F---QGGKI-----GTTLIGRWFIPLN-ET-----N--D-LDKAAAKREFDFSVLGSTG--V--RTISKDNERL-GDRL
AtBglu37             ---------------------Y---QGGKI-----GPVMITRWFLPYD-DT--------L-ESKQATWRAKEFFLGWFMEPLTKGKYPYIMRKLV-GNRL
AtBglu38             ---------------------D---QKGMI-----GPVMITRWFLPFD-HS--------Q-ESKDATERAKIFFHGWFMGPLTEGKYPDIMREYV-GDRL
AtBglu39             ---------------------K---QRGQI-----GVVMITRWFVPYD-ST--------Q-ANIDATERNKEFFLGWFMEPLTKGKYPDIMRKLV-GRRL
AtBglu4              ---------------------K---QGGSI-----GFSLFILGLIPTT-S------S--K-DDATATQRAQDFYVGWFLRPLLFGDYPDTMKRTI-GSRL
AtBglu40             ---------------------K---QGGSL-----GIAFDVMWFEPES-N------K--T-EDIEAAQRAQDFQLGWFLDPLMFGDYPSSMRSRV-GSRL
AtBglu41             ---------------------K---QRGQI-----GISLDAKWYEPMS-D------C--D-EDKDAARRAMDFGLGWFMDPLINGDYPASMKSLV-EERL
AtBglu42             ---------------------S---QGGQI-----GLSVDCEWAEPNS-E------K--P-EDKVAADRRIDFQLGWFLDPLFFGDYPASMRQKL-GDNL
AtBglu43             ---------------------K---QKGRV-----GILLDFVWFEPLT-S------S--Q-ADNDAAQRARDFHVGWFIHPIVYGEYPNTLQNIV-KERL
AtBglu44             ---------------------K---QKGRV-----GILLDFVWYEPLT-R------S--K-ADNLAAQRARDFHIGWFIHPLVYGEYPKTMQNIV-KERL
AtBglu45             ---------------------E---QKGSI-----GIVVQTSWFEPIS-D------S--N-ADKEAAERAQSFYSNWILDPVIYGKYPKEMVDIL-GPAL
AtBglu46             ---------------------E---QKGII-----GIVVQTSWFEPIS-D------S--I-ADKNAAERAQSFYSNWILDPVVYGKYPEEMVNLL-GSAL
AtBglu47             ---------------------Q---QRGQI-----GIVMNTIWFEPIS-D------S--L-ADRLAADRAQAFYLTWFLDPVVFGRYPREMREIL-GDDL
```

```
AtBglu5        ---------------------K---QGGSI-----GFGLYLMGLTPST-S------S--K-DDAIATQRAKDFYFGWFLGPLIFGDYPDTMKRTI-GSRL
AtBglu7        ---------------------K---QRGSV-----GLSIYAYGLSPYT-D------S--K-DDETATERAEAFLFGWMLKPLVVGDYPDIMKRTL-GSRL
AtBglu8        ---------------------K---QRGSI-----GLSIFALGLTPYT-N------S--K-DDEIATQRAKAFLYGWMLKPLVFGDYPDEMKRTL-GSRL
AtBglu9        ---------------------K---QKGSI-----GLSIFAFGLSPYT-N------S--K-DDEIATQRAKTFLYGWMLKPLVFGDYPDEMKKTV-GSRL
BD1G10890      ---------------------A---QQGKV-----GIVLDFNWYEALT-N------S--T-DDEAAAQRARDFHVGWFVDPLINGHYPQIMQDLV-KERL
BD1G10920      ---------------------A---QQGKV-----GIVLDFNWYEALT-N------S--T-DDEAAAQRARDFHVGWFVDPLINGHYPQIMQDLV-KERL
BD1G10930      ---------------------A---QQGKI-----GIVLDFNWYEALT-N------S--A-EDEAAAQRARDFHVGWFVDPLINGHYPQIMQDLV-KERL
BD1G10940      ---------------------S---QKGKI-----GIVLDFNWYEPLT-N------S--T-EDQAAAQRARDFHVGWFLDPLVNGQYPKTMQDIV-KERL
BD1G19270      ---------------------H---QKGKI-----GILLDFVWYEPLS-K------S--K-ADQAAAQRARDFHLGWFLDPIVHGQYPESMLKIV-EGRL
BD1G33040      ---------------------E---QGGLI-----GIALDSKWYEPLS-D------V--D-EDREAAARAMDFELGWFLDPLMFGHYPPSMQKLV-GSRL
BD1G42690      ---------------------E---QKGKI-----GITIVSNWITPYS-N------S--K-EDNDATKRAMDFMYGWFMDPLTKGDYPLSMKTLV-GSRL
BD1G70170      ---------------------T---QKGQL-----GMAFDVMWYEPMT-S------R--A-VDVEAAKRAQEFQLGWFADPFFFGDYPETMRKRV-GERL
BD2G09190      ---------------------K---QKGLI-----GINLYIYNILPFT-N------S--T-EDIAATKRARAFYTGWFLDPLYHGDYPLLMKENT-GSKL
BD2G09200      ---------------------K---QKGLI-----GMNIFIYDILPFT-N------S--T-EDKAAAKRAQAFYTGWFLDPLYFGDYPLVMKENT-GSKL
BD2G27770      ---------------------A---QKGVV-----GMNIYSFWNYPFS-D------S--P-ADVAATQRSLDFMIGWILDPLVYGDYPEIMKKKA-GSRI
BD2G37450      ---------------------V---Q-GHIKVVREGLEQPEFW-EAFS-SAPLHSDSNVK-LSKEQIDSASRTGLGSRRVESYDSDFELVYKAIT-GGVV
BD2G57640      ---------------------V---QGGQV-----GFVIDCEWAEPFS-D------K--M-EDQAAAARRIDFQLGWYLDPIYFGDYPESMRQRL-GDHL
BD2G59650      ---------------------T---QKGLV-----GINVYSFWTYPLT-N------S--T-ADLQATERYQDFVFGWVLGPLVFGDYPQVMKKNV-GSRL
BD2G59660      ---------------------L---QKGVV-----GINIYTFWTYPLT-N------S--T-ADIEATKRCRDFMFNWILEPLVFGDYPQVMKKIV-GSRL
BD3G00650      ---------------------K---QNGEL-----GMSLDVIWYEPAS-N------S--T-ADVEATKRAQEFQLGWFADPFFFGDYPVTMRSRV-GVRL
BD3G40000      ---------------------T---QGGKI-----GITQVSNWFEPYD-PK-----S--L-ADVRAQERSLDFMLGWFQHPVTFGEYPATMRGLV-GSRL
BD3G40010      ---------------------S---QQGQI-----GITQVSHWFVPYD-PS-----S--D-ADLHAQKRALDFMFGWFMHPIVYGEYPGTMRRLV-GARL
BD3G45610      ---------------------E---QKGVI-----GINIYSYWSYPFT-N------S--T-VDLEATQRSKDFMFGWILEPLVSGDYPEVMKNIV-GSRL
BD3G45630      ---------------------E---QKGVV-----GINIYSYWSYPFT-N------S--T-VDLEASQRSKDFMFGWILEPLVSGDYPEVMKKNV-GSRL
BD3G45640      ---------------------E---QKGVV-----GINIYSFWSYPLT-N------S--S-VDLKATQRAKDFIFGWMLEPLVFGDYPEVMKNIV-GSRL
BD3G45650      ---------------------E---QKGAV-----GINIYSFWSYPFK-N------S--S-VDLEATQRAKDFMFGWILEPLVSGDYPEVMKKIV-GSRL
BD4G08040      ---------------------A---QKGKI-----GILLDFVWYEPYN-Y------T--I-EDEYAAHRAREFTLGWFLHPITYGHYPETMQKIV-GDRL
BD4G09920      ---------------------A---MMPIV-----GVSHHVSFTRPYG-L------------------------------------FD-VAAVTI-ANSM
BD4G34930      ---------------------K---QGGHI-----GLTLLGWWYEPAT-Q------T--P-EDIAAAGRMNDFHIGWYMHPLVHGDYPPVMRKNV-GSRL
BD4G34940      ---------------------T---QGGQI-----GITLLGWWHEPAT-N------T--P-QDAAAARRMTEFHIGWFMHPLVYGDYPPVMRSRV-GARL
BD4G34950      ---------------------T---QRGQI-----GITLLGWWHEPAT-K------A--S-RDAAAATRMNDFHIGWFMHPLVYGDYPPVMRRRV-GARL
BD5G13260      ---------------------L---QKGKI-----GISLVSNWFLPLS-R------S--K-SNGDAAIRSVEFMLGWFMDPLIRGDYPLSMRRLV-GNRL
BD5G13270      ---------------------L---QKGKI-----GVSLVSHWFVPFS-C------S--N-SDNDAARRAIDFMLGWFMDPLTSGNYPMSMRGLV-GNRL
BD5G15530      ---------------------K---QGGQI-----GITVSARWYEPFR-N------T--T-VDLLAVQRAISFNAPWFLDPIILGDYPPEMRQML-GANL
BD5G15540      ---------------------K---QGGSI-----GIVVAMKWYEPLT-N------R--T-EDIRAARRALSFEVEWFLDPIFFGDYPREMREML-SANL
GRMZM2G012236  ---------------------K---QGGSV-----GITIHMRWYEPLR-N------I--T-EDHLAVSRALSFEAPWFLDPLFFGDYPHQMRQIL-GPNL
GRMZM2G015804  ---------------------A---QNGEL-----GIAFDVIWYEPMT-N------S--T-IDIEATKRAQEFQLGWFADPFFFGDYPATMRARV-GERL
GRMZM2G031660  ---------------------K---QKGLI-----GIVMSTVWFVPLT-D------A--P-ADRLATERALAFDVPWFLDPIIYGDYPPEMRRLL-GSRL
GRMZM2G055699  ---------------------E---QQGVV-----GINIYTFWNYPFS-P------A--P-ADVQATQRSLDFMIGWMVNPLVYGDYPQVMKRIV-GSRL
GRMZM2G069024  ---------------------V---QKGVV-----GINIYTMWSYPLT-N------S--T-ADLEATQRFLDFYSGWILEPLVFGDYPSVMKKNV-GSRL
GRMZM2G108133  ---------------------A---QGGRI-----GLTLLGWWYEPGT-Q------T--P-DDVAAAARMNDFHIGWFMHPMVFGDYPPVMRRNV-GSRL
GRMZM2G110948  ---------------------T---QRGRV-----GILLDFVWYEPLTAD------S--A-ADRAAAQRSRDFHVGWFLHPIVYGEYPKSVRRSV-KGRL
GRMZM2G112704  ---------------------R---QAGKI-----GILLDFVWYEPLT-K------S--V-EDEYAAHRARMFTLGWFLHPITYGHYPETMQKIV-MGRL
GRMZM2G128197  ---------------------T---QKGKV-----GIVLDFNWYEPFT-N------S--T-EDQKAAQRARDFHIGWFLDPLINGQYPKIMQDIV-KDRL
GRMZM2G148176  ---------------------H---QKGKI-----GILLDFVWYEPFS-D------S--N-ADQAAAQRARDFHLGWFLDPIVHGRYPYSMQEIA-KDRL
GRMZM2G163544  ---------------------E---QGGII-----GIALDSKWYEPLS-D------V--D-EDTEAAARAMDFELGWFLDPLMFGHYPPSMQKLV-GDRL
GRMZM2G177661  ---------------------A---QNGEL-----GIAFDVMWFEPMT-N------T--T-ADIEAAKRGQEFQLGWFADPFFFGDYPASMRSRV-GDRL
GRMZM2G376416  ---------------------E---QGGRI-----GLTLLAWWYEPAT-Q------K--P-EDVEAAARANDFSLGWFMHPLVYGDYPPVMKRNV-GARL
```

```
GRMZM2G457040   ---------------------T---QGGSV-----GIVAAMKWYEPLT-N------S--T-DDILAARRAQAFETDWFLEPIFLGDYPGAMREIL-GSDL
Os10bglu34      ---------------------S---QNGEL-----GISFDVIWYEPMS-N------S--T-ADIEAAKRAQEFQLGWFADPFFFGDYPATMRSRV-GSRL
Os11bglu36      ---------------------E---RKPIV-----GVAHHVSFTRPYG-L------------------------------------FD-VAAVAL-ANSL
Os12bglu38      ---------------------I---QKGKI-----GILLDFVWYEPLT-D------K--E-EDHAAAHRAREFTLGWYLHPITYGHYPETMQNAV-KERL
Os1bglu1        ---------------------I---QKGKI-----GILLDFVWYEGLT-N------S--T-ADQAAAQRSRDFHVGWFLHPIIYGEYPKSLQVIV-KERL
Os1bglu2        ---------------------L---QKGIV-----GINMFSLWSYPLT-N------S--I-ADLQAAQRYKDFSYGWILHPLVFGDYPQVMKKTI-DSRL
Os1bglu3        ---------------------L---QKGIV-----GINVLSLWSYPLT-D------S--T-ADLQAAQRYKDFTYGWILHPLVFGDYPQVMKKAI-GSRL
Os1bglu4        ---------------------V---QGGEV-----GLVVDCEWAEPFS-E------K--T-EDQVAAERRLDFQLGWYLDPIYFGDYPESMRQRL-GDDL
Os1bglu5        ---------------------A---GKGII-----GISVYTFWAYPLT-N------S--T-VDLEATKRCQDFIVHWVLRPLVFGDYPQVMKNIV-GSRL
Os3bglu6        ---------------------T---QNGQL-----GIAFDVMWFEPMS-N------T--T-IDIEAAKRAQEFQLGWFADPFFFGDYPATMRARV-GERL
Os3bglu7        ---------------------A---QQGKV-----GIVLDFNWYEALS-N------S--T-EDQAAAQRARDFHIGWYLDPLINGHYPQIMQDLV-KDRL
Os3bglu8        ---------------------S---QKGKI-----GIVLDFNWYEPLT-N------S--T-EDQAAAQRARDFHVGWFLDPLINGQYPKNMRDIV-KERL
Os4bglu10       ---------------------T---EEAIR-------------QSPFI-------------RDNNL-----NRRSAKFMDPLIRGDYPLSMRELV-GNRL
Os4bglu11       ---------------------L---QKGKI-----GIILNADWFVPLS-Q------S--K-SSSDAARRALDFMLGWFMDPLIRGDYPLSMRELV-GNRL
Os4bglu12       ---------------------L---QKGKI-----GITLVSHWFVPFS-R------S--K-SNDDAAKRAIDFMFGWFMDPLIRGDYPLSMRGLV-GNRL
Os4bglu13       ---------------------L---QKGKI-----GITLVSNWFVPFS-R------S--K-SNIDAARRALDFMLGWFMDPLIRGEYPLSMRELV-RNRL
Os4bglu14       ---------------------K---QGGQI-----GIALSITWYEPFR-N------T--T-IDLLAVKRALSFGASWFLDPILLGDYPTEMREVL-GQSL
Os4bglu16       ---------------------C------------------------------------------------------RFLDPIFFGDYPREMREIL-SSNL
Os4bglu18       ---------------------K---QRGMI-----GMVLYSTWYEPLR-D------V--P-EDRLATERALAFETPWFLDPLVYGDYPPEMRQIL-GGRL
Os4bglu27       ---------------------T---QKGQI-----GITQVSHWFVPYS-D------A--A-ADKHAVRRSLDFMYGWFMDPIVFGDYPGTMRKLV-GDRL
Os4bglu28       ---------------------A---HGGEV-----GITQVSHWFEPYD-AG-----S--A-ADRRARRRALDFMLGWFMHPVAHGEYPPAMRRLV-GGRL
Os4bglu9        ---------------------T---EEVVR---------------------------------------QS-----------------------------
Os5bglu19       ---------------------A---QKGVV-----GMNIYSMWFYPLT-E------S--T-EDIAATERVKDFMYGWILHPLVFGDYPETMKKAA-GSRL
Os5bglu21       ---------------------A---QKGII-----GINIYSMWFYPFT-D------S--A-EEIGATERAKKFIYGWILHPLVFGDYPDTMKKAA-GSRL
Os5bglu22       ---------------------T---QEGFV-----GMNIYSFWNYPFS-S------S--S-ADIAATQRALDFMVGWILDPLVYGDYPEIMKKKA-GSRI
Os5bglu23       ---------------------K---QKGVV-----GTNIYSFWPYPLS-R------S--C-ADIDAVQRVLDFTIGWILDPLVYGDYPEIMKKQA-GSRI
Os5gblu20       ---------------------A---QKGII-----GINMYSLWFYPLT-D------S--A-EDIGATERAKQFMYGWILHPLVFGDYPETIKKVV-GSRL
Os6bglu24       ---------------------G---QKGKI-----GIAIVSNWMIPYE-D------S--K-EDKHATKRALDFMYGWFMDPLTKGDYPVSMRTLV-GNRL
Os6bglu25       ---------------------E---QGGLI-----GIALNSRWYEPFS-N------A--D-EDTEAAARAMDFELGWFLDPLMFGHYPPSMQKLA-GDRL
Os7bglu26       ---------------------Y---QKGRI-----GILLDFVWYEPFS-D------S--N-ADRAAAQRARDFHLGWFLDPIIHGRYPYSMLEIV-KDRM
Os9bglu29       ---------------------T---QHGQI-----GITAVSHWFVPYN-D------T--A-ADRRVVQRSLDFMYGWFLDPIVHGDYPGTMRGWL-GARL
Os9bglu30       ---------------------T---QKGQI-----GMVVVTHWFVPYD-N------S--D-ADRGAVQRSLDFIYGWFMDPIVHGDYPGTMRGWL-GNRL
Os9bglu31       ---------------------T---QGGQI-----GLTLLGWWYEPGT-Q------D--P-EDVAAAARMNDFHIGWYMHPLVYGDYPPVMRKNV-GSRL
Os9bglu32       ---------------------I---QGGQI-----GITLLGWWYEPYT-D------A--V-ADAAAAIRMNEFHIGWFMNPLVHGDYPPVMRSRV-GARL
Os9bglu33       ---------------------I---QGGQI-----GITLMVRWHEPYT-D------K--T-ADAAAAIRMNEFHIGWFLHPLVHGDYPPVMRSRV-GVRL
SB01G010825     ---------------------T---QKGKV-----GIVLDFNWYEPLT-N------S--T-EDQAAAQRARDFHIGWFLDPLINGQYPKTMQDIV-KDRL
SB01G010830     ---------------------A---QKGKV-----GIVLDFNWYEPLT-N------S--T-EDQAAAQRARDFHVGWFVDPLINGHYPQMMQDIV-KERL
SB01G013360     ---------------------T---QNGQL-----GISFDVIWYEPMS-N------S--T-ADVEATKRAQEFQLGWFADPFFFGDYPEIMRSRV-GKRL
SB01G043030     ---------------------A---QNGEL-----GIAFDVIWYEPMT-N------S--T-IDIEATKRAQEFQLGWFAEPFFFGDYPATMRTRV-GERL
SB02G028400     ---------------------A---QRGQI-----GITVVSNWYVPTN-AS-----S--A-ADVKAVQRSLDFMYGWFLDPIVHGEYPGTMLGYL-GDRL
SB02G029620     -------------------------------------------FEPAT-Q------T--P-DDAAAAERMKEFHIGWFMHPIVYGDYPPVMQKNV-GPRL
SB02G041550     ---------------------Y---QKGRI-----GILLDFVWYEPFS-D------S--N-ADQAAAQRARDFHLGWFLDPIINGRYPYSMQEIV-KDRL
SB03G037780     ---------------------V---QKGVV-----GINIYTMWAYPLT-N------S--T-ADLEASQRFLDFYCGWILEPLVFGDYPSVVKKNV-GSRL
SB03G042690     ---------------------A---QGGEV-----GFVVDCEWAEPFS-E------K--A-EDQIAAQRRIDFQLGWYLDPIYFGDYPESMRQRL-GSDL
SB06G019830     ---------------------V---QKGKI-----GITIVSHWFTPFS-Q------S--R-SDIDAARRAVDFMLGWSLDPLIRGDYPLSMKRLV-GNRL
SB06G019840     ---------------------V---QKGKI-----GITLVTNWFVPFS-R------S--R-SNDDAARRALDFMFGWFMDPLIRGHYPLSMRRLV-RNRL
SB06G019850     ---------------------E---QKGNI-----GITLVSSWFVPFS-H------S--K-SNDDAARRAIDFMLGWFMDPLTRGEYPLSMRALV-GNRL
SB06G019860     ---------------------E---QKGNI-----GITLVSQ----------------------------------WFMDPLTRGEYPLSMRALV-GNRL
```

```
SB06G019880    ---------------------V---QRGKI-----GVTLVSLWFLPLS-P------S--K-SNEDAVTRALDFMLGWFMDPLVGGDYPLSMRRLV-GNRL
SB06G022410    ---------------------K---QGGSV-----GITVYMRWYEPLR-N------I--T-DDHLAVSRAQSFEAPWFLDPLFFGDYPHQMRQIL-GPNL
SB06G022420    ---------------------K---QGGSI-----GISLYMRWYEPLR-N------I--T-EDHLAVSRALSFQAPWFLDPLFFGDYPHQMRQIL-GPNL
SB06G022450    ---------------------K---QGGYI-----GITVLSRWYEPFR-N------I--P-TDILAVDRGLSFGAPWFLDPIILGDYPSPMRKML-GPNL
SB06G022460    ---------------------K---QGGRI-----GITVQSRWYEPFR-N------T--P-TDILGVERALSFGAPWFLDPIILGHYPSAMRKIL-GPNL
SB06G022490    ---------------------K---QGGSI-----GIVTAMKWYEPLT-N------T--T-EDILAARRAQSFETEWFLDPIFFGDYPRAMREIL-QSNL
SB06G022500    ---------------------K---QKGMI-----GIVMAAFWFVPLT-D------T--P-VDRMATERALAFSVPWYLDPIVYGDYPPEMRQLL-GSKL
SB06G022510    ---------------------K---QKGMI-----GIVMSALWLVPLT-D------T--P-VDRLATERALAFDAPWFLDPIIYGDYPPEMRQLL-GSKL
SB08G007586    ---------------------G---ADGRI-----GLALNVFGRVPYT-NT---------F-LDQQAQEMSMDKCLGWFLEPVLRGDYPFSMRVSA-RDRL
SB08G007650    -----------------------------------------------------------------AQERSMDNCLGWFLEPVVRGDYPFSMRASA-KDRV
SB09G018160    ---------------------V---RKGVV-----GINLYSLCIYSLT-D------L--A-EDIQATERANDFLFGSILNPFLFGDYPESMKKAA-GARL
SB10G012220    ---------------------K---QKGSI-----GITLVSNWMIPYT-N------S--K-GDKDAAKRALEFMYGWFMDPLTKGDYPLSMRTLV-GNRL
SB10G022300    ---------------------E---QGGRI-----GLTLLAYWYEPAT-H------K--P-EDVQAAARANDFTLGWFMHPLVHGDYPPVMRRNA-GSRL
SB10G027600    ---------------------D---QGGII-----GIALDSKWYEPLS-D------V--D-EDTEAAARAMDFELGWFLDPLMFGHYPPSMQKLA-GDRL
SB10G028060    ---------------------H---QGGKI-----GILLDFVWYEPLT-K------S--I-EDEFAAHRARMFTLGWFLHPITYGHYPETMEKIV-MGRL
SbDhr1         ---------------------G---ADGRI-----GLALNVFGRVPYT-NT---------F-LDQQAQERSMDKCLGWFLEPVVRGDYPFSMRVSA-RDRV
SbDhr2         ---------------------G---EKGRI-----GLALNVMGTVPYG-ST---------F-LDEQAHERCMDYNLGWYLEPVVRGDYPHSMRSSV-RDRL
Zm-p60.1       -------------------------DDTRI-----GLAFDVMGRVPYG-TS---------F-LDKQAEERSWDINLGWFLEPVVRGDYPFSMRSLA-RERL
ZmGLU2         -------------------------ENGRI-----GLAFDVMGRVPYG-TS---------F-LDEQAKERSMDINLGWFLEPVVRGDYPFSMRSLA-RERL
ZmGLU3         -------------------------TDGRI-----GLAFDVMGRVPYG-NT---------F-LDEQAQERSLDQNLGWFLEPVVRGDYPFSMRSLA-RKRL
ZmGLU4         ---------------------DYKGENGRI-----GIVFDVMGRVPFE-KS------A--F-IDQQAEERSWDINLGWFLEPVVRGDYPFSMRSLV-RDRL
ZmGLU5         -------------------------ENGRI-----GLAFDVMGRVPYE-KS------A--F-TDQQAEQRSWDINLGWFLEPVVRGDYPFSMRSLA-RERL
ZmGLU6         -------------------------ENGRI-----GIAFDVMGRVPYE-KS------A--F-VDQQAQERSWDINLGWFLEPLVRGDYPFSMRSLV-RDRL
ZmGLU7         ---------------------N---KDAQI-----GIAFDVMGRVPYD-NM---------F-LDDQAQERSIDYNLGWFMEPVVRGDYPFSMRSLI-KDRL
ZmGLU8         ---------------------N---KEGHI-----GMAFDVMGRVPYE-KM---------F-LDDQAQERSIDYNLGWFVEPVVRGDYPFSMRSLV-KDRL
```

```
                            1710      1720      1730      1740      1750      1760      1770      1780      1790      1800
                    ....|....|....|....|....|....|....|....|....|....|....|....|....|....|....|....|....|....|....|....|
AC155376.2_FGT005   PTFSEKDKK-FI---------------K-NKIDFIGLNH-YT-SRLIAH-------------HQNPDD---V--YF--YKV-QQME-RV---EKWS----
AC211140.2_FGT007   PKFTADEAA-LV---------------K-GALDFMGINH-YT-TFYTRH-------------NDTNIIG--RLLND--TLA-DTGTISL---PFDK----
AC217401.3_FGT001   PKFTPEQAK-LV---------------K-GSADYIGINQ-YT-ASYVKG-------------QKLLQ-Q---KPTS--YSA-DWQV-QY---VLER----
AC217687.3_FGT004   PRFTPEQAK-LV---------------K-GSADYIGINE-YT-SSYMKG-------------QKLVQ-L---APSS--YSA-DWQV-QY---VFAR----
AtBglu1             PVFSKEESE-QV---------------K-GSSDFIGVIH-YL-TALVTN-------------IDINPSL--SGIPD--FNS-DM----------------
AtBglu10            PVFSEEESE-QL---------------K-GSSDFIGIIH-YT-TFYVTN-------------KPSPS-IFPSMNEG--FFK-DMGVYM----IS------
AtBglu11            PAFTEEESE-QV---------------K-GAFDFVGVIN-YM-ALYVKD-------------NSSSLK--P-NLQD--FNT-DIAVEM----TL------
AtBglu12            PTFTAKQSK-ML---------------K-GSYDFIGRNY-YS-SSYAKD-------------VPCSSE----N-VT--LFS-DPCAS-V---TGER----
AtBglu13            PTFTAKQSK-ML---------------K-GSYDFIGINY-YS-SSYAKD-------------VPCSSE----N-VT--LFS-DPCAS-V---TGER----
AtBglu14            PTFTSKQSN-ML---------------K-GSYDFIGINY-YS-SSYAKD-------------VPCSSE----N-VT--MFS-DPCAS-V---TGER----
AtBglu15            PIFTAQQSK-ML---------------K-GSYDFIGINY-YS-STYAKD-------------VPCSTK----D-VT--MFS-DPCAS-V---TGER----
AtBglu16            PTFTPEESE-ML---------------K-GSYDFIGVNY-YS-SLYAKD-------------VPCATE----N-IT--MTT-DSCVS-L---VGER----
AtBglu17            PKFTKKQSK-MV---------------R-GSFDFFGLNY-YT-SRYVED-------------VMFYAN---TN-LS--YTT-DSRVN-Q---TTEK----
AtBglu18            PKFTEAEKK-LL---------------K-GSTDYVGMNY-YT-SVFAKE-------------ISPD-P---KS-PS--WTT-DSLVDWD---SKSV----
AtBglu19            PRFTKAQKA-KL---------------K-DSTDFVGINY-YT-SFFAKA-------------DQKVDS---RN-PT--WAT-DALVEFE---PKTV----
AtBglu2             PIFSKEESE-QV---------------K-GSSDFIGIMH-YF-PALVEN-------------IKLKPSL--SRNTD--FYS-DMGVSL----TY------
AtBglu20            PKFTEAQKE-KL---------------K-NSADFVGINY-YT-SVFALH-------------DEEPDP---SQ-PS--WQS-DSLVDWE---PRYV----
AtBglu21            PQFTAAQKA-KL---------------K-DSTDFVGLNY-YT-STFSNY-------------NEKPDP---SK-PS--WKQ-DSLVSWE---PKNV----
AtBglu22            PKFTSSQKA-KL---------------K-DSTDFVGLNY-YT-STFSNH-------------NEKPDP---ST-PS--WKQ-DSLVAWE---PKNV----
AtBglu23            PKFTTEQKA-KL---------------K-ASTDFVGLNY-YT-SVFSNH-------------LEKPDP---SK-PR--WMQ-DSLITWE---SKNA----
AtBglu24            PKFTTEQIA-KL---------------K-NSADFVGINY-YT-STFSKH-------------LEKPNH---AE-PK--FKQ-DSLVEWK---NKNV----
AtBglu25            PSFTPEQKE-KL---------------K-GSYDFVGINY-FT-STFVAH-------------TDNVNP---EK-PS--WEA-DSRLQLH---SNNV----
AtBglu26            PSFTPEQSK-KL---------------I-GSCDYVGINY-YS-SLFVKS-------------IKHVDP---TQ-PT--WRT-DQGVDWM---KTNI----
AtBglu27            PSFTAAQSK-KL---------------R-GSFDFVGVNY-YS-AFYVKN-------------IDEVNH---DK-PN--WRS-DARIEWR---KENN----
AtBglu28            PSFTPEQSK-ML---------------K-NSSDFIGINY-YT-ARYVAH-------------IPQADP---AR-PR--FVT-DHQLQWR---VTNH----
AtBglu29            PAFTPEQSK-ML---------------I-NSSDFIGVNY-YS-IHFTAH-------------LPHIDH---TR-PR--FRT-DHHFEKK---LINR----
AtBglu3             PVFSKEESE-QV---------------K-GSSDFIGIIH-YL-AASVTS-------------IKIKPSI--SGNPD--FYS-DMGVSM----TWT-----
AtBglu30            PSFTVEQSK-ML---------------Q-NSSDFVGINY-YT-ARFAAH-------------LPHIDP---EK-PR--FKT-DHHVEWK---LTNH----
AtBglu31            PSFTKEQSM-MV---------------K-NSFDFIGVNY-YT-ARFVAH-------------DLNVDI---SR-PR--FMT-DQHLQYK---LTNR----
AtBglu32            PSFTKEQSM-ML---------------Q-NSFDFIGINY-YT-ARFVAH-------------DLHVDL---SR-PR--FTT-DQHLQYK---LTNR----
AtBglu33            REFTPEESE-KL---------------R-KSLDFVGLNY-YG-AFFSTP-------------LAKVNS---SQ-LN--YET-DLRVNWT---V-IT----
AtBglu34            PEFTPEQSA-LV---------------K-GSLDFLGLNY-YV-TQYATD-------------APPPT-----Q-LN--AIT-DARVTLG----FYR----
AtBglu35            PEFTPEESA-LV---------------K-GSLDFLGLNY-YV-SQYATD-------------APPPT-----Q-PN--AIT-DARVTLG----FYR----
AtBglu36            PKFTPKQSA-LL---------------K-GSLDFLGLNY-YV-TRYATY-------------RPPPMP---TQ-HS--VLT-DSGVTIG----FER----
AtBglu37            PKFNSTEAR-LL---------------K-GSYDFLGLNY-YV-TQYAHA-------------LDPSPP---EK-LT--AMT-DSLANLT---SLDA----
AtBglu38            PEFSETEAA-LV---------------K-GSYDFLGLNY-YV-TQYAQN-------------NQTIVP---SDVHT--ALM-DSRTTLT---SKNA----
AtBglu39            PKFNKKEAK-LV---------------K-GSYDFLGINY-YQ-TQYVYA-------------IPANPP---NR-LT--VLN-DSLSAFS----YEN----
AtBglu4             PVFSEKESE-QV---------------K-GSCDFVGVIH-YH-AASVTN-------------IKSKPSL--SGNPD--FYS-YMET--------------
AtBglu40            PVFTGSQSS-LV---------------K-GSLDFVGINH-YT-TYYARN-------------NATNLIG--TLLHD--AVS-DSGTV-T---LPFK----
AtBglu41            PKITPEMYK-TI---------------K-GAFDYVGINH-YT-TLYARN-------------DRTRIRK--LILQD--ASS-DSAV-IT---SSFR----
AtBglu42            PRFTPEEKEFML---------------Q-NSWDFLGLNH-YT-SRLISH-------------VSNKEAE--S--NF--YQA-QELE-RI---VELE----
AtBglu43            PKFTEEEVK-MV---------------K-GSIDFVGINQ-YT-TYFMSD-------------PKISTTP---KDLG--YQQ-DWNV-TF---NFAK----
AtBglu44            PKFTEKEVK-MV---------------K-GSIDFVGINQ-YT-TYYMSE-------------PHPTTKP---KDLG--YQQ-DWNV-EF---GFAK----
AtBglu45            PQFSSNEVK-NL---------------EKSRADFVGINH-YT-SYFIQD-------------CLTSAC----NTGHGAFKA-EGYA-LK---LDR-----
AtBglu46            PKFSSNEMN-SL---------------MSYKSDFLGINH-YT-SYFIQD-------------CLITAC----NSGDGASKS-EGLA-LK---LDR-----
AtBglu47            PEFTKDDLK--S---------------SKNALDFIGINQ-YT-SRYAKD-------------CLHSVC----EPGKGGSRA-EGFV-YA---NAL-----
```

```
AtBglu5       PVFSEEESE-QF---------------A-ASVTNIKF-----------------------------KPSI--SGNPD--FYS-D-----------------
AtBglu7       PVFSEEESK-QV---------------K-GSSDFVGVVH-YN-TFYVTN-------------RPAPSLV-TSINKL--FFA-DIGAYL----IA------
AtBglu8       PVFSEEESE-QV---------------K-GSSDFVGIIH-YT-TVYVTN-------------QPAPYIFPSSTNKD--FFT-DMGAYI----IS------
AtBglu9       PVFSEEESE-QV---------------K-GSSDFIGIIH-YT-TFYVTN-------------HQPSASLFPSMGEG--FFK-DMGVYI----IP------
BD1G10890     PRFTPDETK-LV---------------N-GSADYIGINQ-YT-ANYIKG-------------QKLVP-Q---KPTS--YSA-DWQV-TY---ASDR----
BD1G10920     PRFTPDETK-LV---------------N-GSADYIGINQ-YT-ANYIKG-------------QKLVP-Q---KPTS--YSA-DWQV-TY---ASDR----
BD1G10930     PRFTSDEVK-IV---------------K-GSADYIGINQ-YT-ASYIKG-------------QKLVQ-Q---APTS--YSA-DWQV-TY---ASLR----
BD1G10940     PSFTSEQSK-LV---------------K-GSADYFGINQ-YT-ASYMAD-------------QPTPQ-Q---APTS--YSS-DWHV-SF---IFQR----
BD1G19270     PTFSHEESR-MV---------------K-GSMDYVGINH-YT-SYYMKD-------------PGAWN-L---TPVS--YQD-DWHV-GF---VYER----
BD1G33040     PQFSKQESQ-SV---------------S-GSLDFVGINH-YT-TLYARN-------------DRMRVRK--LVMND--AST-DAAV-IP---TAYR----
BD1G42690     PKFTKEQAR-AL---------------N-GSFDFIGLNY-YS-ARYAQN-------------TKHNCK---IN-KS--YST-DSRAN-Q---RVER----
BD1G70170     PRFTPEEAE-LV---------------K-GALDFVGINH-YT-TYYTRQ-------------NDTNIIG--KLFND--TLA-DTGTI-S---LPFR----
BD2G09190     PIFSQNQSE-QL---------------I-NSVDFLGINY-YK-IIYVKD-------------DPQNGP---INKSD--YVA-DMSAKA----ILAS----
BD2G09200     PKFSENQSE-QL---------------I-NSVDFLGINY-YA-IMHVKD-------------NPHDAP---SNRRD--FMA-DMSAKA----IFPS----
BD2G27770     PAFTEEQSE-LI---------------R-GSIDFVGINH-YT-SVYVSD-------------GKSSAD---AGLRD--YNA-DLSATF----RLSK----
BD2G37450     PAFSSS--G-AGDETHLPARESSWSLLR-HKFVTRSLSRVYSDSALIRDLDPRVQHLAAEASISPPFL----SPSS--FSS-DSSIS-S---KYSS----
BD2G57640     PKFSEKERE-LI---------------R-NKIDFIGLNH-YT-SRFIAH-------------QQDPQA---I--HF--YQV-QQME-RI---EKWN----
BD2G59650     PSFTKFQSE-AI---------------K-GAIDFIGINH-YF-SIYVND-------------RPLDE-----GPRD--YEA-DMSVYQ----RGSR----
BD2G59660     PSFTKVQSE-AV---------------K-GSVDFIGINH-YY-TLYVND-------------SPLQK-----GVRD--FAL-DMSSAY----RGSK----
BD3G00650     PRFTTKEAD-LV---------------K-GSLDFMGINH-YT-TFYTKD-------------DNSTYIK--KFLND--TLA-DSGSI-S---LPFR----
BD3G40000     PEFTPEQKK-KL---------------A-GSFDFIGINY-YT-SNYAKH-------------APAPNA---LT-PA--YGT-DNNAN-Q---TGYR----
BD3G40010     PEFTTEQKE-LL---------------K-GSFDFIGLNY-YT-SNYAKA-------------APAPNKL--EK-PS--YGT-DNRVN-Q---TGFR----
BD3G45610     PSFTMVQSG-LV---------------K-DSFDFFGINH-YY-SFYVSD-------------RPMET-----GVRD--FYG-DMSISY----RASR----
BD3G45630     PSFTKIQSG-LI---------------K-NSFDFIGINH-YF-SVYVND-------------RPIER-----GARD--FNG-DMSVYY------------
BD3G45640     PSFTKVQSV-LI---------------K-DSFDFFGINH-YY-SLYVND-------------RPIEI-----DVRD--FNA-DMSIYY----RASR----
BD3G45650     PSFTKVQSG-LI---------------K-DSFDFFGINH-YY-SLYVSD-------------RPIET-----GVRD--FYG-DMSISY----RASR----
BD4G08040     PSFSPEQTA-LV---------------Q-GSADYIGINH-YT-SYYVKH-------------YVNL-T-----HMS--YAN-DWQA-KI---SYDR----
BD4G09920     TLFPYI--D-SI---------------C-DKLDFIGINY-YG-QEVISG-------------AGLKLV----DNDE--YSE-SGRG--------------
BD4G34930     PSFTAEELK-RV---------------H-GSFDFVGFNH-YI-AIYVKA-------------DLSKLD---QPLRD--YMG-DAAVAYDMPFLNSK----
BD4G34940     PVLTAPVSK-KV---------------R-RSFDFIGFNH-YI-IMRIRS-------------IDTNSS---QQPRD--YYV-DAAVQN-----PA-----
BD4G34950     PYLTAEQSK-NL---------------S-GSFDFVGFNH-YL-VVRAQS-------------DERAFD---RKQRD--YYN-DAAAIA----NPF-----
BD5G13260     PRFTKEQSE-LV---------------K-GAFDFIGINY-YT-TSYADN-------------LPPSNG---LK-NS--YNT-DARAN-L---TGVR----
BD5G13270     PQFTKEQSR-LV---------------K-GAFDFIGINY-YS-ANYADD-------------LPPSNG---LN-IS--YNT-DARVN-L---TGVR----
BD5G15530     PNFTSKEKR-KL---------------QATKLDFIGLNH-YT-TVYLKD-------------CIFSP----CAVDP--IDG-DARV-VS---LAER----
BD5G15540     PKFTPEEKK-LM---------------Q-NKVDFIGINQ-YT-AIYARD-------------CISLP----CNIMT--YEG-NAMV-LA---TGER----
GRMZM2G012236 PKFTAGEEK-LL---------------KN-QIDFIGVNH-YQ-TFYVKD-------------CIYSL----CDIDP--YTS-EALV-SE---STER----
GRMZM2G015804 PKFTADEAA-LV---------------K-GALDFMGINH-YT-TFYTRH-------------NDTNIIG--RLLND--TLA-DTGTISL---PFDK----
GRMZM2G031660 PTFSPEERR-TL----------------GYGLDFIGINH-YT-TLYARD-------------CMISP----GYCPSGQEFH-QSLA-AY---TGER----
GRMZM2G055699 PRFTKRQSE-MV---------------R-GTADFIGINH-YT-SVYVSD-------------RPNDAAADTTGPRD--YNA-DLSATF----RFSR----
GRMZM2G069024 PSFSKVQSE-AI---------------R-GTLDFIGINH-YY-SFYVND-------------RPLEK-----GIRD--FSL-DIAADY----RGSR----
GRMZM2G108133 PTFTDEEAA-RV---------------R-GSFDFVGFNH-YI-VVYVKA-------------DLGRLD---DQVRD--YMG-DAAVKYDMPFLKSR----
GRMZM2G110948 PKFTAEEAG-LV---------------R-GSIDYVGVNQ-YT-AYYVRD-------------RRPNATA---APPS--YSS-DWHA-EF---VYER----
GRMZM2G112704 PNFTFEQSA-MV---------------K-GSADYVAINH-YT-TYYASN-------------FVNA-T-----ETN--YRN-DWNA-KI---SYER----
GRMZM2G128197 PSFTPEQAK-LV---------------K-GSSDYFGINQ-YT-TYYIAD-------------QQTPP-Q---GPPS--YSS-DWGV-QY---YFQR----
GRMZM2G148176 PLFSDEEAR-MV---------------K-GSIDYVGINH-YT-SFYMKD-------------PGTWN-L---TPVS--YQD-DWHV-GF---VYER----
GRMZM2G163544 PQFSARASM-LV---------------S-GSLDFVGINH-YT-TLYVRN-------------DRMRIRK--LVMND--AST-DAAV-IP---TAYR----
GRMZM2G177661 PRFTAAEAA-LV---------------K-GALDFVGINH-YT-TYYTKH-------------NSTDLVG--RLLHN--TLA-DTGTI-S---LPFR----
GRMZM2G376416 PSLTARDSA-MV---------------R-GSLDFVGINQ-YG-AILVEA-------------DLGQLD---RDLRD--YYG-DMATNFVTV--PFE----
```

```
GRMZM2G457040   PTFTAEEKA-LL---------------LRYKADFIGLNH-YT-AIYARD-------------CLRSP----CNLGS--YEG-NAFV-SA---TGER----
Os10bglu34      PKFTEKEAA-LV---------------N-GSLDFMGINH-YT-TFYTKD-------------DQSTVIE--KLLNN--TLA-DTATI-S---VPFR----
Os11bglu36      TLFPYV--D-SI---------------C-DKLDFIGINY-YG-QEVISG-------------PGLKLV----DNDE--YSE-SGRG--------------
Os12bglu38      PNFTREQSE-MI---------------K-GSADYIAINH-YT-TYYVSH-------------HVNK-T-----SIS--YLN-DWDV-KI---SYER----
Os1bglu1        PKFTADEVH-MV---------------K-GSIDYVGINQ-YT-AYYVRD-------------QQPNA-T---TLPS--YSS-DWHA-AP---IYER----
Os1bglu2        PSFSQVQTE-LI---------------K-GAIDFIGINH-YY-SAYVNY-------------RPLVE-----GVRD--YVA-DRSVSA----RVYK----
Os1bglu3        PSFSKVQTE-LV---------------K-GTLDFIGVNH-YF-SLYVSD-------------LPLAK-----GVRD--FIA-DRSVSC----RGLL----
Os1bglu4        PTFSEKDKE-FI---------------R-NKIDFVGINH-YT-SRFIAH-------------HQDPED---I--YF--YRV-QQVE-RI---EKWN----
Os1bglu5        PSFTKAQSE-DV---------------K-GSLDFIGMNH-YY-SLYVND-------------RPLGK-----GTRD--FVA-DISIYY----RGSK----
Os3bglu6        PRFTADEAA-VV---------------K-GALDFVGINH-YT-TYYTRH-------------NNTNIIG--TLLNN--TLA-DTGTV-S---LPFK----
Os3bglu7        PKFTPEQAR-LV---------------K-GSADYIGINQ-YT-ASYMKG-------------QQLMQ-Q---TPTS--YSA-DWQV-TY---VFAK----
Os3bglu8        PTFTPEQAK-LV---------------K-GSADYFGINQ-YT-ANYMAD-------------QPAPQ-Q---AATS--YSS-DWHV-SF---IFQR----
Os4bglu10       PEFSKEQSE-MV---------------K-GAFDFIGLNY-YA-SSYADN-------------DPPSYG---HN-NS--YNT-DSHAK-I---TGSR----
Os4bglu11       PEFSKEQSG-MV---------------K-GAFDFIGLNY-YT-SSYADN-------------DPPSHG---HN-NS--YNT-DAHAK-I---TGSR----
Os4bglu12       PQFTKEQSK-LV---------------K-GAFDFIGLNY-YT-ANYADN-------------LPPSNG---LN-NS--YTT-DSRAN-L---TGVR----
Os4bglu13       PQFTKEQSE-LI---------------K-GSFDFIGLNY-YT-SNYAGS-------------LPPSNG---LN-NS--YST-DARAN-L---TAVR----
Os4bglu14       PKFTSKQKN-RL---------------QSTKLDFIGLNH-YT-TCYVKD-------------CIFSP----CEIDP--VNA-DARV-FS---LYER----
Os4bglu16       PKFTPEEKK-LL---------------QNNKVDFIGINH-YT-AIYAKD-------------CIYSP----CTLDT--YEG-NALV-YA---IGRR----
Os4bglu18       PSFSPEDRR-KL----------------RYKLDFIGVNH-YT-TLYARD-------------CMFSD----CPQGQ--ETQ-HALA-AV---TGES----
Os4bglu27       PKFTAEQSE-LV---------------K-GSYDFIGLNY-YT-TNYAKS-------------VLRRPSK--LK-PA--YAT-DNWVN-Q---TAYR----
Os4bglu28       PAFTAEQSE-ML---------------R-GSFDFIGLNY-YT-SNYAVA-------------APPPNKL---H-PS--YLT-DNWVN-A---TGYR----
Os4bglu9        --------------------------------QFIH----------------------------------------------------------------
Os5bglu19       PLFSDYESE-LV---------------T-NAFDFIGLNH-YT-SNYVSD-------------NSNAVK---APLQD--VTD-DISSLF----WASK----
Os5bglu21       PIFSNHESE-MA---------------V-KWFCLLLLKQ-FV----------------------------------------------------------
Os5bglu22       PSFTEEQSE-LI---------------R-GSADFIGINH-YT-SVYISD-------------ASNGET---VGPRD--YSA-DMAATF----RISR----
Os5bglu23       PSFTKEQSE-LI---------------R-GSADFIGINH-YK-SLYVSD-------------GSNREK---AGLRD--YNA-DMAAHF----RVSR----
Os5gblu20       PFFSNHESE-LV---------------T-NAFDFIGLNH-YS-SVYTSN-------------NNNVVK---APLQD--LTA-DIATLF----RATK----
Os6bglu24       PRFTKEQSK-AI---------------N-GSFDFIGLNY-YT-ARYIQG-------------TKQDSN---SH-KS--YST-DSLTN-E---RVER----
Os6bglu25       PQFSTHASK-LV---------------S-GSLDFVGINH-YT-TLYARN-------------DRLRIRK--LVMDD--AST-DSAV-IP---TAYR----
Os7bglu26       PTFSDEESR-MV---------------K-DSIDYVGINH-YT-SFYMKD-------------PGPWN-L---TPTS--YQD-DWHV-GF---AYER----
Os9bglu29       PAFTAEQAA-AV---------------R-GSYDFIGVNY-YT-TYYAKS-------------VPLPSS---NR-LS--YDT-DIRAN-T---TGFR----
Os9bglu30       PEFTPEQSA-MV---------------K-GSYDFIGVNY-YT-TYYAKS-------------IPPPNS---NE-LS--YDL-DNRAN-T---TGFR----
Os9bglu31       PSFTAEESK-RV---------------L-ESYDFVGFNH-YV-AIFVRA-------------DLSKLD---QSLRD--YMG-DAAVKYDLPFLKSN----
Os9bglu32       PSITASDSE-KI---------------R-GSFDFIGINH-YF-VIFVQS-------------SDANHD---QKLRD--YYV-DAGVQE----NGG-----
Os9bglu33       PSITASDSE-KI---------------R-GSFDFIGINH-YY-VIFVQS-------------IDANE----QKLRD--YYI-DAGVQG------------
SB01G010825     PSFTPEQAK-LV---------------K-GSSDYFGINQ-YT-TNYISN-------------QQTTQ-Q---GPPS--YSS-DWGV-QY---NFER----
SB01G010830     PKFTPGQAK-LV---------------K-GSADYIGINQ-YT-ASYIKG-------------QKLLQ-Q---KPTS--YSA-DWQV-QY---ALER----
SB01G013360     PKFTAEEAA-LV---------------K-GSLDFMGINH-YT-TFYVQD-------------DESSVVG--TLLNN--TLA-DTGTI-S---LPFR----
SB01G043030     PKFTADEAT-LV---------------K-GALDFMGINH-YT-TFYTRH-------------NETNIIG--RLLND--TLA-DTGTISL---PFDK----
SB02G028400     PRFTAAQAK-LI---------------K-GSYDFIGVNY-YT-AYFASA-------------KPAPNG---ME-QS--YDG-DIRAN-T---SGYR----
SB02G029620     PSFTDEERK-KV---------------K-GSFDFVGFNH-YI-VVHVRA-------------DLNRLK---QKLRD--YMG-DAAVKF----DSNQ----
SB02G041550     PLFSDEESR-MV---------------K-GSIDYVGINH-YT-SFYMKD-------------PGTWN-L---TPVS--YQD-DWHV-GF---VYER----
SB03G037780     PSFRKVQSE-AI---------------R-GTIDFIGINH-YL-SVYVND-------------HPLEK-----GIRD--FVL-DVAADY----RVSR----
SB03G042690     PTFSEKDKE-FI---------------R-NKIDFIGLNH-YT-SRLIAH-------------HQNPDD---V--YF--YQV-QQME-RI---EKWN----
SB06G019830     PQFTKEQSK-LV---------------K-GAFDFIGLNY-YT-GYYTED-------------VPPSL-----N-KS--YNT-DAQAN-T---TGVR----
SB06G019840     PQFTSEQSK-LV---------------K-GAFDFIGLNY-YT-TNYAAN-------------LPPSNG---LN-LS--YST-DSQAN-L---TGVR----
SB06G019850     PQFTKEQSE-LV---------------K-GAFDFIGLNY-YT-TNYADN-------------LPQSNG---LN-VS--SRT-DARVN-L---TGKYVLKS
SB06G019860     PQFTKEQSE-LV---------------K-GAFDFIGLNY-YT-TNYADN-------------LPQSNG---LN-VS--YST-DARFR-------------
```

```
SB06G019880    PRFTKEQSK-LL---------------K-GAFDFIGLNY-YT-TYYAAS-------------LPPSSN---G--------------L-Y---SSIR----
SB06G022410    PEFTEGEKQ-LM---------------KN-QIDFIGVNH-YK-TLYVKD-------------CVYSL----CDLDT--YAG-DALV-SE---SAER----
SB06G022420    PKFTKGEKQ-LL---------------KN-QIDFIGINH-YE-TLYIKD-------------CIHSL----CDLDT--YAG-DALV-TE---SAER----
SB06G022450    PEFTSKQKK-IL---------------QPSKLDFIGLNH-YS-TSYLKD-------------CIYSS-P--CELDP--FDG-DAQI-ST---SIDR----
SB06G022460    PEFTLKQKK-IL---------------QTSKLDFIGLNH-YS-TNYLKD-------------SISSSSP--CELDQ--YDG-DAQI-ST---SAER----
SB06G022490    PTFTAEEKK-LL---------------LQYKPDFIGLNH-YT-AIYAKD-------------CIHSP----CNLQT--YEG-NAFV-LA---TGEK----
SB06G022500    PTFSPVEKR-KL----------------GYKLDFIGINH-YT-TLYVKD-------------CMFTS----GCPSG-QDIQ-YALA-AY---TGER----
SB06G022510    PTFSPEERR-KL----------------GYKLDFIGINH-YT-TLYAKD-------------CMFSS----GCPSSGQEIH-HALA-AF---TGER----
SB08G007586    PYFKEKEQE-KL---------------V-GSYDMIGINY-YT-STFSKH-------------NDISANY---S-PV--LNTDDAYASQK---TQGP----
SB08G007650    PYFKEIEQE-KL---------------T-GN---------------------------------------------------------------------
SB09G018160    PSFSSYESE-LV---------------T-GAFDFIGLNH-YS-SIYASN-------------NPDASK---MPVRD--QAA-DVGALF----RDTR----
SB10G012220    PRFTKEQSK-AI---------------H-GSFDFIGLNY-YT-ARYVQN-------------TKHSNN---GN-RS--YNT-DSRTN-Q---SVER----
SB10G022300    PVLTAQESA-MV---------------R-GSFDFVGINQ-YG-ALLVEA-------------DLGQLK---RELRD--YYG-DTAVNFITL--PFE----
SB10G027600    PKFSTQASK-LV---------------S-GSLDFVGINH-YT-TLYVRN-------------DRMRIRK--LVMND--AST-DAAI-IP---TAYR----
SB10G028060    PNFTFEQSA-MV---------------K-GSADYIAINH-YT-TYYASN-------------FG------------------------------------
SbDhr1         PYFKEKEQE-KL---------------V-GSYDMIGINY-YT-STFSKH-------------IDLSPNN---S-PV--LNTDDAYASQE---TKGP----
SbDhr2         PHFTEKEQQ-KL---------------V-GSYDMIGINY-YS-SRFAKH-------------VDITENF---S-PE--LNTHDCCATEE---ITGP----
Zm-p60.1       PFFKDEQKE-KL---------------A-GSYNMLGLNY-YT-SRFSKN-------------IDISPNY---S-PV--LNTDDAYASQE---VNGP----
ZmGLU2         PFFSDKQQE-KL---------------V-GSYNMLGINY-YT-SIFSKH-------------IDISPKY---S-PV--LNTDDAYASQE---TYGP----
ZmGLU3         PFFTDNEQA-ML---------------A-GSYDILGINY-YT-SRFSKH-------------VDFSEDY---S-PK--LNADDAYATAE---IFGP----
ZmGLU4         PFFTNEERE-KL---------------V-GSYDMLGLNY-YT-SRFSKH-------------IDITQHN---T-LR--LNTDDAYASQE---TKGP----
ZmGLU5         PFFTDKEQE-KL---------------V-GSYDMLGLNY-YT-SRFSKN-------------IDISPNY---S-PV--LNTDDAYASQE---TNGP----
ZmGLU6         PFFTVEEQE-RL---------------V-GSYDMLGLNY-YT-ARFSKH-------------IDISPNY---S-PA--LNTDDAYASQE---TYGP----
ZmGLU7         PYFTDDEKE-KL---------------V-GSYDIMGINY-YT-SRFSKH-------------IDISPKY---S-PV--LNTDDAYAAQE---TKGP----
ZmGLU8         PYFTDEEKE-KL---------------V-GSYDIMGINY-YT-SRFSKH-------------VDISTGY---T-PV--LNTDDAYATQE---TKGP----
```

```
                         1810      1820      1830      1840      1850      1860      1870      1880      1890      1900
                  ....|....|....|....|....|....|....|....|....|....|....|....|....|....|....|....|....|....|....|....|
AC155376.2_FGT005 ---------------S-GES-IGE--R-AASEW-L-----------VIVPWGLHKLL---NYIVKKYNN-------------------------------
AC211140.2_FGT007 ---------------N-GKP-IGD--R-ANSIW-L-----------YIVPSGIRKLM---NYVKERDGR-------------------------------
AC217401.3_FGT001 ---------------N-GKP-IGP--Q-ANSKW-L-----------YIVPEGMYGCV---NYLKHKYGN-------------------------------
AC217687.3_FGT004 ---------------N-GKP-IGP--Q-ANSKW-L-----------YIAPTGMYGCV---NYLKEKYGN-------------------------------
AtBglu1           ------------------------------------------------------GIL---EYIKQSYGN-------------------------------
AtBglu10          ---------------------AAN--S--SFLL-W-----------EATPWGLEGIL---EYIKQSYNN-------------------------------
AtBglu11          ---------------------VGN--TS-IENE-Y-----------ANTPWSLQQIL---LYVKETYGN-------------------------------
AtBglu12          ---------------E-GVP-IGP--K-AASDW-L-----------LIYPKGIRDLL---LYAKYKFKD-------------------------------
AtBglu13          ---------------E-GVP-IGP--K-AASDW-L-----------LIYPKGIRDLL---LYAKYKFKD-------------------------------
AtBglu14          ---------------D----------------------------------GGIRDLI---LYAKYKFKD-------------------------------
AtBglu15          ---------------D-GVP-IGP--K-AASDW-L-----------LIYPKGIRDLV---LYAKYKFKD-------------------------------
AtBglu16          ---------------N-GVP-IGP--A-AGSDW-L-----------LIYPKGIRDLL---LHAKFRYND-------------------------------
AtBglu17          ---------------N-GVP-VGE--P-TSADW-L-----------FICPEGFQDVL---LYIKSKFQN-------------------------------
AtBglu18          ---------------D-GYK-IGS--K-PFNGK-L-----------DVYSKGLRYLL---KYIKDNYGD-------------------------------
AtBglu19          ---------------DGSIK-IGS--Q-PNTAK-M-----------AVYAKGLRKLM---KYIKDRYNS-------------------------------
AtBglu2           ---------------------LGN--F--SGFG-Y-----------DVFPWAMESVL---EYIKQTYGN-------------------------------
AtBglu20          ---------------DKFNA-FAN--K-PDVAK-V-----------EVYAKGLRSLL---KYIKDKYGN-------------------------------
AtBglu21          ---------------D-HSA-IGS--M-PLTAA-L-----------PVYAKGFRKLL---KYIKDKYAN-------------------------------
AtBglu22          ---------------D-HSA-IGS--Q-PLTAA-L-----------PVYAKGFRSLL---KYIKDKYAN-------------------------------
AtBglu23          ---------------Q-NYA-IGS--K-PLTAA-L-----------NVYSRGFRSLL---KYIKDKYAN-------------------------------
AtBglu24          ---------------N-NIT-IGS--K-PETGP-L-----------PVYSTGFRKVL---KYVKDKYAN-------------------------------
AtBglu25          ---------------D-GFK-IGS--Q-PATAK-Y-----------PVCADGLRKVL---KYIKENYND-------------------------------
AtBglu26          ---------------D-GKQ-IAK--Q-GGSEW-S-----------FTYPTGLRNIL---KYVKKTYGN-------------------------------
AtBglu27          ---------------A-GQT-LGV--R-GGSEW-D-----------FLYPQGLRKFL---NYAKNKYES-------------------------------
AtBglu28          ---------------S-NHQ-FGP--G-EDRGI-L-----------QSHPEGLRKVL---NYIKDKYNN-------------------------------
AtBglu29          ---------------S-NHE-TGP--G-DDRGK-I-----------HSHPEGLRRVL---NYIKDKYNN-------------------------------
AtBglu3           -------------------V-LGN--F--SAFE-Y-----------AVAPWAMESVL---EYIKQSYGN-------------------------------
AtBglu30          ---------------S-GHI-IGP--G-EERGF-L-----------FSHPEGLRKVL---NYIKERYNN-------------------------------
AtBglu31          ---------------T-GDT-ISL--ESDGTKI-L-----------WSYPEGLRKIL---NYIKNKYNN-------------------------------
AtBglu32          ---------------S-GDH-ISS--ESDGTKI-L-----------WSYPEGLRKLL---NYIKNKYNN-------------------------------
AtBglu33          ---------------N-NLS-LPD--LQTTSMG-I-----------VIYPAGLKNIL---KHIKDEYMD-------------------------------
AtBglu34          ---------------N-GVP-IGV--V-APS---F-----------VYYPPGFRQIL---NYIKDNYKN-------------------------------
AtBglu35          ---------------N-GSP-IGV--V-ASS---F-----------VYYPPGFRQIL---NYIKDNYKN-------------------------------
AtBglu36          ---------------N-GVS-IGV--K-ASI---N-----------FDV-KDLRHLVDFFLFVELLLLS-------------------------------
AtBglu37          ---------------N-GQP-PGP--P-FSK-G-S-----------YYHPRGMLNVM---EHFKTKYGD-------------------------------
AtBglu38          ---------------T-GHA-PGP--P-FNA-A-S-----------YYYPKGIYYVM---DYFKTTYGD-------------------------------
AtBglu39          ---------------K-DGP-IGP--W-FNA-D-S-----------YYHPRGILNVL---EHFKTKYGN-------------------------------
AtBglu4           -------------------D-FGK--S--LDFQ-Y-----------ANTPWAMEVVL---EYIKQSYGN-------------------------------
AtBglu40          ---------------G-LST-IGD--R-ASSIW-L-----------YIVPRGMRSLM---NYIKHRYGN-------------------------------
AtBglu41          ---------------G-GVA-IGE--R-AGSSW-L-----------HIVPWGIRKLA---VYVKDIYGN-------------------------------
AtBglu42          ---------------N-GDL-IGE--R-AASDW-L-----------YAVPWGIRKTL---NYMSKKYNH-------------------------------
AtBglu43          ---------------N-GTP-IGP--R-AHSEW-L-----------YNVPWGMYKAL---MYIEERYGN-------------------------------
AtBglu44          ---------------L-GKP-IGP--R-AYSSW-L-----------YNVPWGMYKAL---MYMKERYGN-------------------------------
AtBglu45          --------------KG-NVT-IGE--L-TDVNW-Q-----------HIDPTGFHKML---NYLKDRYPN-------------------------------
AtBglu46          --------------KG-NVS-IGE--L-TDVNW-Q-----------HIDPNGFRKML---NYLKNRYHN-------------------------------
AtBglu47          --------------KD-GLR-LG-------------------------EPVGMEEML---MYATERYKN-------------------------------
```

```
AtBglu5        -----------------------------MGAY-Y-----------PVAPWTMEAVL---EYIKQSYDN-------------------------------
AtBglu7        ---------------------AGN--A--SLFE-F-----------DAVPWGLEGIL---QHIKQSYNN-------------------------------
AtBglu8        ---------------------TGN--S--SSFV-F-----------DAVPWGLEGVL---QHIKHRYNN-------------------------------
AtBglu9        ---------------------TGN--S--SFLV-W-----------EATPWGLEGIL---EYIKQSYNN-------------------------------
BD1G10890      ---------------N-GIP-IGP--K-GE-VW--------------------KSSS---RHNRKRYRY-------------------------------
BD1G10920      ---------------N-GIP-IGP--K-ANSNW-L-----------YIVPTGMYGCV---NYLREKYGN-------------------------------
BD1G10930      ---------------N-GKP-IGP--K-ANSDW-L-----------YIVPTGMYGCV---NYLRVKYGN-------------------------------
BD1G10940      ---------------N-GKP-IGP--L-ANSNW-L-----------YIVPTGMYGCV---NYIKEKYKN-------------------------------
BD1G19270      ---------------N-GVP-IGA--R-ANSYW-L-----------YIVPWGINKAV---TYVKERYGN-------------------------------
BD1G33040      ---------------H-GKR-IGE--T-AASSW-L-----------HIVPWGMFKLM---KHVKEKYGN-------------------------------
BD1G42690      ---------------N-GTY-IGP--K-AGSSW-L-----------YIYPKGIEELL---LYTKETYNN-------------------------------
BD1G70170      ---------------N-GKA-IGD--R-ANSIW-L-----------YIVPRGMRSLM---NYVKDRYNS-------------------------------
BD2G09190      --------------D-----------------------------------STTGEEL---EYLKQSYGN-------------------------------
BD2G09200      --------------N--------------STTG-F-----------YVPGFGLQEVL---EYLKQSYGN-------------------------------
BD2G27770      ---------------N-DSG-TGQ--F--IPIN-M-----------PDDPQGLQCML---QYLTDKYQN-------------------------------
BD2G37450      ---------------D-SPS-LSP--S-ASSPPSLGLSPASSNLPHALVPSSRSPLR---QSSSEEPSKPVLGSLHSPSKVSSIAERRGGFSPLKLPSLP
BD2G57640      ---------------T-GEG-IGE--R-AASEW-L-----------LIVPWGLRKAI---NYIVKKYNN-------------------------------
BD2G59650      ---------------T-DPP-SGQ--F--NPED-F-----------PNDPDGLQFVL---QYLTEAYGG-------------------------------
BD2G59660      ---------------T-DPP-VGK--Y--APTA-I-----------PNDPEGLQLMM---LYLKETYGD-------------------------------
BD3G00650      ---------------D-GKP-IGD--K-ANSIW-L-----------YIVPGSMRSLM---NYVKDRYNT-------------------------------
BD3G40000      ---------------N-GVP-IGP--P-AFTPI-F-----------FNYPPGLRELL---LYIKRTYKD-------------------------------
BD3G40010      ---------------D-GVP-IGP--P-AYTPI-F-----------YNYPPGLRELL---LYAKKRYNN-------------------------------
BD3G45610      ---------------T-DPP-AGQ--G--VPTN-V-----------PSDPDGLHLVL---EYLKETYGN-------------------------------
BD3G45630      ----------------------------------------------------------------------------------------------------
BD3G45640      ---------------T-GPP-AGQ--D--ERMS-L-----------F
BD3G45650      ---------------T-GPP-AGQ--G--APTN-V-----------PSDPKGLQLVL---EYLKEAYGN-------------------------------
BD4G08040      ---------------N-GVL-IGK--Q-AFSNW-L-----------YVVPWGFYKAV---MHVKDKFRN-------------------------------
BD4G09920      -----------------------------------------------VYPDGLFRVL---IKFNERYKSL------------------------------
BD4G34930      --------------NK-PFL-FG---L--KSDI-M-----------TSTPWALKKML---GHLQLKYKN-------------------------------
BD4G34940      ----------------------D---NI-SKVQ-V-----------ETAPWSLSKLL---EHLKLNYGN-------------------------------
BD4G34950      ----------------------K---DI-QEGH-L-----------ESAPWALGKLL---DHLRLKYRN-------------------------------
BD5G13260      ---------------N-GVA-IGP--Q-AASPW-L-----------YVYPPGFRELL---LYVKKNYGN-------------------------------
BD5G13270      ---------------N-GVP-IGP--Q-YASPG-L-----------YVYPQGLRDLL---LYVKGHYGN-------------------------------
BD5G15530      ---------------D-GVL-IGE--P-TGTPY-F-----------YDVPHGMEKVV---MHYKQRYNN-------------------------------
BD5G15540      ---------------D-GVL-IGK--P-TAFKG-Y-----------YDVPQGMEQAV---KYVNERYEN-------------------------------
GRMZM2G012236  ---------------N-GIP-IGK--L-TQDAN-T-----------YVVPSSMEKLV---MYLKERYNN-------------------------------
GRMZM2G015804  ---------------N-GKP-IGD--R-ANSIW-L-----------YIVPSGIRKLM---NYVKERYNS-------------------------------
GRMZM2G031660  ---------------D-GIP-IGP--P-TAMPT-F-----------YVVPDGIEKMV---TYIMNRYSN-------------------------------
GRMZM2G055699  ---------------D-DPA-TGQ--F--VPIN-M-----------PSDPQGLQCML---EYLSQTYNN-------------------------------
GRMZM2G069024  ---------------T-DPP-IGQ--H--APTS-I-----------PADPRGLQLLV---EYLSEAYGN-------------------------------
GRMZM2G108133  ---------------N-QFP-FG---AL-TSDF-M-----------TSTPWALKKML---RHLRVTYKN-------------------------------
GRMZM2G110948  ---------------D-GVP-IGP--R-ANSDW-L-----------YIVPWGLYKAV---TYVKEKYGN-------------------------------
GRMZM2G112704  ---------------D-GVP-IGK--R-AYSDW-L-----------YVVPWGLYKAL---IWTKEKFNS-------------------------------
GRMZM2G128197  ---------------N-GVQ-IGQ--M-AHSIW-L-----------YIVPSGMYGVV---NYLKEKYHN-------------------------------
GRMZM2G148176  ---------------N-GVP-IGA--H-ANSYW-L-----------YIVPWGINKAV---SYVKETYKN-------------------------------
GRMZM2G163544  ---------------H-GKK-IGE--T-AASGW-L-----------HIVPWGMFKLM---KHIKEKYGN-------------------------------
GRMZM2G177661  ---------------N-GKA-IGD--R-ANSIW-L-----------YIVPSGMRSLM---NYVKERYNS-------------------------------
GRMZM2G376416  ---------------S-TVT-RNQQVPR-LGLR-N-----------HEAPWALSKLL---EHLQTHYGN-------------------------------
```

```
GRMZM2G457040   --------------DD-GVK-IGG--D-TALAG-F----------FDVPEAIELAI---QYVNGRYKG------------------------------
Os10bglu34      ---------------N-GQP-IGD--R-ANSIW-L----------YIVPRSMRILM---NYVKDRYNK------------------------------
Os11bglu36      ----------------------------------------------VYPDGLFRIL---IQFNERYKRL-----------------------------
Os12bglu38      ---------------N-GVP-IGK--Q-AYSNW-L----------YVVPWGIYKAV---MHVKEKYKD------------------------------
Os1bglu1        ---------------D-GVP-IGP--R-ANSDW-L----------YIVPWGLYKAV---TYVKEKYGN------------------------------
Os1bglu2        ---------------T-DPP-TEK--Y--EPTE-Y----------PNDPKGLQLAL---EYLRESYGD------------------------------
Os1bglu3        ---------------Q-GVRFIAQ-TMQ-APTR-S----------MGDPHGLQLML---QHLKESYGD------------------------------
Os1bglu4        ---------------T-GEK-IGE--R-AASEW-L----------FIVPWGLRKLL---NYAAKRYGN------------------------------
Os1bglu5        ---------------T-DPP-PGK--A--APTS-I----------GPDPQGLRLMV---QYLQETYGN------------------------------
Os3bglu6        ---------------N-GKP-IGD--R-ANSIW-L----------YIVPRGMRSLM---NYVKERYNS------------------------------
Os3bglu7        ---------------N-GKP-IGP--Q-ANSNW-L----------YIVPWGMYGCV---NYIKQKYGN------------------------------
Os3bglu8        ---------------N-GVP-IGQ--Q-ANSNW-L----------YIVPTGMYGAV---NYIKEKYNN------------------------------
Os4bglu10       ---------------N-GIP-IGP--Q-AASFW-F----------YIYPEGLRELL---LHIKENYGN------------------------------
Os4bglu11       ---------------N-GIP-IGP--Q-AASFW-F----------HIYPEGICEML---LYVKENYGN------------------------------
Os4bglu12       ---------------N-GIP-IGP--Q-AASPW-L----------YVYPQGFRDLL---LYVKENYGN------------------------------
Os4bglu13       ---------------N-GIP-IGP--Q-AASPW-L----------YIYPQGFRELV---LYVKENYGN------------------------------
Os4bglu14       ---------------D-GVP-IGK--A-TGAPF-F----------HDVPRGMEEAV---TYYKQRYNN------------------------------
Os4bglu16       ---------------N-GKI-IGK--P-TALHG-Y----------FVVPEAMEKVV---MYVNDRYRN------------------------------
Os4bglu18       ---------------N-GLP-IGT--P-TAMPT-F----------YVVPDGIEKMV---KYFMRRYNN------------------------------
Os4bglu27       ---------------N-GVP-IGP--P-AFTKI-F----------FTYAPGLRELL---LYTKRKYND------------------------------
Os4bglu28       ---------------N-SIP-IGP--P-AYTPI-F----------FNYPPGLRELL---LYVKRRYNN------------------------------
Os4bglu9        --------------------------------------------------------------------------------------------------
Os5bglu19       ---------------N-STP-TRE---F--LPG-T----------SLDPRGLELAL---EYLQEKYGN------------------------------
Os5bglu21       -------------------------------PG-T----------IVDPRGLEHAL---KYIREKYGN------------------------------
Os5bglu22       ---------------N-DTP-SGQ--F--VPTR-L----------PRDPKGLQCML---EYLRDTYQG------------------------------
Os5bglu23       ---------------N-DTP-SDK--C--LSIY-Q---------------------------------------------------------------
Os5gblu20       ---------------N-DTP-TPE---F--LPG-N----------TVDPQGLENAL---EYIRENYGN------------------------------
Os6bglu24       ---------------N-GTD-IGP--K-AGSSW-L----------YIYPKGIEELL---LYTKRTYNN------------------------------
Os6bglu25       ---------------H-GKK-IGE--T-AASSW-L----------HIVPWGMFKLM---KHVKEKYGN------------------------------
Os7bglu26       ---------------N-GVP-IGA--Q-ANSYW-L----------YIVPWGINKAV---TYVKETYGN------------------------------
Os9bglu29       ---------------N-GKP-IGP--Q-EFTPI-F----------FNYPPGLRELL---LYTKRRYNN------------------------------
Os9bglu30       ---------------N-GKP-IGP--Q-EFTPI-F----------FNYPPGLRELL---LYTKRRYNN------------------------------
Os9bglu31       ---------------N-EFP-LG---L--TSDF-M----------TSTPWALKKML---NHLQEKYKN------------------------------
Os9bglu32       ----------------------G---GF-DKEH-Y----------QLHPWALGKML---HHLKLKYGN------------------------------
Os9bglu33       --------------------------ED-DTEN-I----------QCHSWSLGKVL---NHLKLEYGN------------------------------
SB01G010825     ---------------N-GVQ-IGQ--L-AHSVW-L----------YIVPTGMYGVV---TYLKEKYQN------------------------------
SB01G010830     ---------------N-GKP-IGP--Q-ANSNW-L----------YIVPSGMYGCV---NYLKQKYGN------------------------------
SB01G013360     ---------------N-GKP-IGD--R-ANSIW-L----------YIVPGSMRSLM---NYVKDRYNT------------------------------
SB01G043030     ---------------N-GKP-IGD--R-ANSIW-L----------YIVPSGIRKLM---NYVKERYNS------------------------------
SB02G028400     ---------------D-GVP-IGT--P-EFVPI-F----------FEYPQGLRELL---LYTSRRYGS------------------------------
SB02G029620     ---------------F-PIR-LN---SL-TIDF-K----------TSKPWALKKLL---RHIRVKYKN------------------------------
SB02G041550     ---------------N-SIP-IGA--H-ANSYW-L----------YIVPWGINKAV---NYVKETYEN------------------------------
SB03G037780     ---------------T-DPP-VGQ--H--APTS-I----------PADPRGLQLMV---EYLSEAYGN------------------------------
SB03G042690     ---------------S-GEK-IGE--R-AASEW-L----------FIVPWGLRKVL---NYIVKKYNN------------------------------
SB06G019830     ---------------G-GLP-IGR--Q-AASPS-L----------YIYPQGFLELL---LHVKENYGN------------------------------
SB06G019840     ---------------N-GVP-IGP--Q-AASSW-L----------YIYPQGFRDLL---LYVKENYGN------------------------------
SB06G019850     RINILLLFIYQVFGMV-SQL-VLR--L-HRSPW-L----------FIYPRGFRELL---LYVKENYGN------------------------------
SB06G019860     ---------------N-GVP-IGP--Q-AASSW-L----------FIYPRGFRELL---LYVKENYGN------------------------------
```

```
SB06G019880     ---------------N-GVP-IGP--Q-AASSW-L-----------FMYPQGFRELL---LYMKKNYGN-------------------------------
SB06G022410     ---------------N-GIP-IGK--P-TPVAN-N-----------YVVPSSMEKLV---MYLTQRYKS-------------------------------
SB06G022420     ---------------N-GIL-IGK--P-TPVAN-T-----------CVVPSSMEKLV---MYLKQRYKS-------------------------------
SB06G022450     ---------------D-GIL-IGE--R-TGSPY-L-----------NVVPYGMEKVV---MYFKRRYNN-------------------------------
SB06G022460     ---------------D-GIL-IGE--R-TGSPY-I-----------NTVPYGIEKVV---TYLNTRYNN-------------------------------
SB06G022490     ---------------D-GVK-IGR--D-TALSG-F-----------YDVPEAIEPAI---MFVNGRYKD-------------------------------
SB06G022500     ---------------N-GIP-IGP--P-TAMPL-F-----------FDVPDGIEKMV---TYIMKRYNN-------------------------------
SB06G022510     ---------------N-GIP-IGP--P-TAMPK-F-----------YFVPDGIEKMV---TYIMKRYNN-------------------------------
SB08G007586     ---------------D-GNA-IGP--P-TGNAW-I-----------NMYPKGLHDIL---MTMKNKYGN-------------------------------
SB08G007650     -------------------------------AW-I-----------NMYPKGLHDIL---MTMKNKYGN-------------------------------
SB09G018160     ---------------D-GPA-AIQ---Y--PAG-T-----------MVDPQGLEHVL---KYIREKYGN-------------------------------
SB10G012220     ---------------N-GTV-IGP--K-AGSPW-L-----------YIYPKGIEELL---LYTKKKYNN-------------------------------
SB10G022300     ---------------S-TVR---NQEPQ-LGLR-N-----------KEAPWALNKVL---EHLQIQYGN-------------------------------
SB10G027600     ---------------H-GKK-IGD--T-AASGW-L-----------HIVPWGMFKLM---KHIKEKYGN-------------------------------
SB10G028060     ----------------------------YSDW-L-----------YVVPWGLYKAL---IWTKEKFNN-------------------------------
SbDhr1          ---------------D-GNA-IGP--P-TGNAW-I-----------NMYPKGLHDIL---MTMKNKYGN-------------------------------
SbDhr2          ---------------N-GNT-IGP--A-TGNAW-V-----------YMYPKGLKDIL---MIMKKRYGN-------------------------------
Zm-p60.1        ---------------D-GKP-IGP--P-MGNPW-I-----------YMYPEGLKDLL---MIMKNKYGN-------------------------------
ZmGLU2          ---------------D-GKP-IGP--P-MGNPW-I-----------YLYPEGLKDIL---MIMKNKYGN-------------------------------
ZmGLU3          ---------------D-GNS-IGP--P-MGNPW-I-----------YMYPKGLKDLL---MIMKNKYGN-------------------------------
ZmGLU4          ---------------D-GEP-IGP--P-MG-NW-I-----------YLYPQGLKDLL---KIMKEKYGN-------------------------------
ZmGLU5          ---------------D-GNP-IGP--W-MGNSW-I-----------YLYPEGLKDLL---MIMKNKYGN-------------------------------
ZmGLU6          ---------------D-DKP-IGP--W-MGNPW-I-----------YMYPDGLKDLL---MIMKNKYGN-------------------------------
ZmGLU7          ---------------D-GNT-IGP--S-MGNPW-I-----------YMYPNGLKDIL---MFMKNKYGN-------------------------------
ZmGLU8          ---------------D-GNT-IGP--S-MGNSW-I-----------YMYPNGLKDIL---MVMKNKYGN-------------------------------
```

```
                                   1910      1920      1930      1940      1950      1960      1970      1980      1990      2000
                              ....|....|....|....|....|....|....|....|....|....|....|....|....|....|....|....|....|....|....|....|
AC155376.2_FGT005             ---------P--VIY-V--------TENGM---------------DDE-DDQ--------------S--A-T-I-----------------DQ-------
AC211140.2_FGT007             ---------RQQPFH-LP-------PERPQ---------------GQQ-EDQ--------------V--P-QRLPQQRCCLHKVLNITVPPHV-------
AC217401.3_FGT001             ---------P--TVF-I--------TENGM---------------DQP-GN-----------------L-T-R-----------------GQ-------
AC217687.3_FGT004             ---------P--TIY-I--------TENGM---------------DQP-GN-----------------L-T-R-----------------DQ-------
AtBglu1                       ---------P--PVY-I--------LENGK---------------T---------------------MNQD--L-----------------EL-------
AtBglu10                      ---------P--PIY-I--------LENGM---------------P---------------------MGRD-----------------------S-------
AtBglu11                      ---------P--PVY-I--------LENGQ---------------M---------------------TPHS-----------------------S-------
AtBglu12                      ---------P--VMY-I--------TENGR---------------DEA-STG---------------------------------------KI-------
AtBglu13                      ---------P--VMY-I--------TENGR---------------DEA-STG---------------------------------------KI-------
AtBglu14                      ---------P--VMY-I--------TENGR---------------DEA-STG---------------------------------------KI-------
AtBglu15                      ---------P--VMY-I--------TENGR---------------DEF-STN---------------------------------------KI-------
AtBglu16                      ---------P--VLY-I--------TENGV---------------DEA-NIG---------------------------------------KI-------
AtBglu17                      ---------P--VIL-V--------TENGM---------------PSE-NDK--------------S--L-S-V-----------------NI-------
AtBglu18                      ---------P--EVI-I--------AENGY---------------GEDLGEK--------------HN---D-V-----------------NF-------
AtBglu19                      ---------P--EII-I--------TENGY---------------GEDLGDK--------------DT---D-L-----------------SV-------
AtBglu2                       ---------P--PVY-I--------LENGT---------------P---------------------MKPD--L-----------------EL-------
AtBglu20                      ---------P--EIM-I--------TENGY---------------GEDLGEQ--------------DT---S-L-----------------VV-------
AtBglu21                      ---------P--EIM-I--------MENGY---------------GDK-LGT--------------TD---S-V-----------------DV-------
AtBglu22                      ---------P--EIM-I--------MENGY---------------GDK-LKD--------------KD---S-V-----------------EV-------
AtBglu23                      ---------P--EIM-I--------MENGY---------------GEE-LGA--------------SD---S-V-----------------AV-------
AtBglu24                      ---------P--EII-I--------MENGY---------------GEN-LKE--------------ND---S-V-----------------EN-------
AtBglu25                      ---------P--EII-V--------TGNGY---------------KET-LEE--------------KD---V-L-----------------PD-------
AtBglu26                      ---------P--PIL-I--------TENGY---------------GEV-AEQ--------------SQSL-Y-M-----------------YN-------
AtBglu27                      ---------P--KFM-I--------TENGH---------------CDI-DYE--------------KK---P-K-----------------LS-------
AtBglu28                      ---------P--IVY-I--------KENGI---------------NDY-DDG--------------TK---S-R-----------------EE-------
AtBglu29                      ---------P--IVY-V--------KENGI---------------DHY-DDG--------------TK---S-R-----------------ET-------
AtBglu3                       ---------P--PIY-I--------LENGT---------------P---------------------MKQD--L-----------------QL-------
AtBglu30                      ---------M--PVY-I--------KENGI---------------NDN-DDG--------------TK---P-R-----------------EE-------
AtBglu31                      ---------P--TIY-I--------TENGF---------------DDY-ENG--------------TV---T-R-----------------EE-------
AtBglu32                      ---------P--TIY-I--------TENGF---------------DDY-ENG--------------SV---T-R-----------------EE-------
AtBglu33                      ---------P--EIY-I--------MENGM---------------DEI-DYG--------------TK---N-I-----------------TE-------
AtBglu34                      ---------P--LTY-I--------TENGV---------------ADL-DLG--------------NV---T-L-----------------AT-------
AtBglu35                      ---------P--LTY-I--------TENGV---------------ADL-DLG--------------NV---T-L-----------------AT-------
AtBglu36                      ---------T--R-I-P--------SDSKS---------------HQK-QEL--------------LM---L-I-----------------AN-------
AtBglu37                      ---------P--LIY-V--------TENGF---------------STS-GG---------------PI---P-F-----------------TE-------
AtBglu38                      ---------P--LIY-V--------TENGF---------------STP-GD----------------E---D-F-----------------EK-------
AtBglu39                      ---------P--LVY-I--------TENGE---------------L------------------------------------------------------
AtBglu4                       ---------P--PVY-I--------LESAK---------------FTF-QW---------QQIGTP-MKQD--S-----------------QL-------
AtBglu40                      ---------P--PVF-I--------TENGM---------------DDP-NSI--------------L--I-S-R-----------------KD-------
AtBglu41                      ---------P--PVF-I--------TENGM---------------DEK-NSP--------------F--I-D-M-----------------EK-------
AtBglu42                      ---------P--PIF-I--------TENGM---------------DDE-DDG--------------S--A-S-I-----------------HD-------
AtBglu43                      ---------P--TMI-L--------SENGM---------------DDP-GN-----------------I-T-L-----------------TQ-------
AtBglu44                      ---------P--TMI-L--------SENGM---------------DDP-GN-----------------V-T-L-----------------AQ-------
AtBglu45                      ---------M--PMF-I--------TENGF---------------GDL-QKP--------------E--T-T-D-----------------KE-------
AtBglu46                      ---------I--PMY-I--------TENGF---------------GQL-QKP--------------E--T-T-V-----------------EE-------
AtBglu47                      ---------I--TLY-V--------TENGF---------------GEN-NTG----------------------------------------V-------
```

```
AtBglu5        ---------P--PVY-I--------LENGT--------------P---------------------M------------------------T-------
AtBglu7        ---------P--PIY-I--------LENGK--------------P---------------------MKHG---------------------S-------
AtBglu8        ---------P--PIY-I--------LENGS--------------P---------------------MKHD---------------------S-------
AtBglu9        ---------P--PVY-I--------LENGM--------------P---------------------MVRD---------------------S-------
BD1G10890      ---------C--SMY-L--------RM-----------------DQP-GN------------------L-T-R-----------------DE-------
BD1G10920      ---------P--AVV-I--------TENGM--------------DQP-GN------------------L-T-R-----------------DE-------
BD1G10930      ---------P--AIV-I--------TENGM--------------DQP-GN------------------L-T-R-----------------DE-------
BD1G10940      ---------P--TII-I--------SENGM--------------DQP-GN------------------L-T-R-----------------EE-------
BD1G19270      ---------P--TMF-L--------SENGM--------------DQP-GN------------------V-S-I-----------------TE-------
BD1G33040      ---------P--PVF-I--------TENGM--------------DDA-NNR--------------F--S-R-L-----------------EN-------
BD1G42690      ---------P--TIY-I--------TENGV--------------DEI-NNE--------------N--L-P-L-----------------QE-------
BD1G70170      ---------P--PVY-I--------TENGM--------------DDG-NSP--------------F--I-S-L-----------------KD-------
BD2G09190      ---------P--PIC-I--------HENGS--------------RAA-RP--------------E--APA--A-----------------AHDLASVLM
BD2G09200      ---------P--PIC-I--------HENGK--------------YFT----------------------------------------------------
BD2G27770      ---------I--PIY-V--------QENGY--------------GQF-F-----------------------------------------ND-------
BD2G37450      KDLVLPPRAP--PSSHTAGEVMDKSNTNGVKQLAGVPHPEKCTGNNST-T-------------------------------------------------
BD2G57640      ---------P--VIY-V--------TENGM--------------DDE-DDQ--------------S--A-P-L-----------------DQ-------
BD2G59650      ---------L--PIY-V--------HENGD--------------AS--------------------------D-----------------ND-------
BD2G59660      ---------I--PIY-V--------QESGH--------------GS--------------------------G-----------------ND-------
BD3G00650      ---------P--TVY-I--------TENGM--------------DDS-NSP--------------F--I-S-L-----------------KK-------
BD3G40000      ---------P--AIY-I--------TENGT--------------DEA-NNS--------------T--I-P-I-----------------KE-------
BD3G40010      ---------P--AIY-I--------TENGT--------------DEA-NNS--------------T--I-P-I-----------------EE-------
BD3G45610      ---------P--PLY-V--------HENGM--------------GS--------------------------P-----------------KD-------
BD3G45630      ---------------------------RGV--------------GS--------------------------P-----------------ND-------
BD3G45640      --------------------------GAGL--------------GS--------------------------A-----------------ND-------
BD3G45650      ---------P--PLY-V--------HENGV--------------GS--------------------------P-----------------ND-------
BD4G08040      ---------P--VIV-I--------GENGI--------------DQA-GN------------------D-T-L-----------------PH-------
BD4G09920      --------DI--PFI-I--------TENGV--------------SDE-T--------------------------------------------------
BD4G34930      ---------P--VVM-I--------HENGK--------------LTN-ST------------------VN--L-----------------KH-------
BD4G34940      ---------P--PVW-I--------HENGY--------------GSA-AP--------------G--ALS--K-----------------TE-------
BD4G34950      ---------P--PVM-I--------HENGF--------------ADA-PK------------------TP--S-----------------KI-------
BD5G13260      ---------P--IVY-I--------TENGV--------------YEA-NNK--------------S--L-P-L-----------------KE-------
BD5G13270      ---------P--DIY-I--------TENGF--------------NEV-NNK--------------S--L-P-L-----------------QE-------
BD5G15530      ---------T--PTY-I--------TENGY--------------AQA-SNH--------------S--M-A-A-----------------ND-------
BD5G15540      ---------T--PVY-V--------TENGY--------------SQQ-SDN-------------------S-V-----------------ED-------
GRMZM2G012236  ---------I--PLY-I--------TENGY--------------AQI-PNI--------------S--T-T-V-----------------EE-------
GRMZM2G015804  ---------P--TVY-I--------TENGM--------------DDG-NSP--------------F--T-S-L-----------------QN-------
GRMZM2G031660  ---------L--PMF-I--------TENGY--------------AQG-GDG--------------Y--TTR-A-----------------ED-------
GRMZM2G055699  ---------I--PVY-V--------QENGY--------------GAL-F-----------------------------------------ND-------
GRMZM2G069024  ---------L--PIY-I--------QETGY--------------AT--------------------------T-----------------NG-------
GRMZM2G108133  ---------P--AVM-I--------HENGA--------------AGQ-PD------------------PS--G-----------------VN-------
GRMZM2G110948  ---------P--TML-L--------SENGM--------------DDP-GN------------------V-T-V-----------------GQ-------
GRMZM2G112704  ---------P--VML-I--------GENGI--------------DQP-GN------------------E-T-L-----------------PF-------
GRMZM2G128197  ---------P--III-I--------SENGM--------------DQP-GN------------------L-T-R-----------------EE-------
GRMZM2G148176  ---------P--TMI-L--------AENGM--------------DQP-GD------------------V-S-I-----------------TQ-------
GRMZM2G163544  ---------P--PVI-I--------TENGM--------------DDA-NNR--------------F--S-K-L-----------------ED-------
GRMZM2G177661  ---------P--PIY-V--------TENGM--------------DDG-NSP--------------F--T-S-I-----------------KD-------
GRMZM2G376416  ---------P--PVM-I--------HENGA--------------GHE-PD------------------PS--G-----------------G--------
```

```
GRMZM2G457040   ---------T--PVY-I--------TENGY---------------SQW-SDA-------------------S-R-----------------EE-------
Os10bglu34      ---------P--TVY-I--------TENGM---------------DDG-NSP--------------F--I-S-L-----------------KN-------
Os11bglu36      --------NI--PFV-I--------TENGV---------------SDE-T--------------------------------------------------
Os12bglu38      ---------P--III-I--------GENGI---------------DQP-GN------------------E-T-L-----------------PG-------
Os1bglu1        ---------P--TMF-L--------SENGM---------------DDP-GN------------------V-T-I-----------------AQ-------
Os1bglu2        ---------F--PFY-I--------EENGK---------------GS--------------------------T-----------------ND-------
Os1bglu3        ---------L--PIY-V--------QENGK---------------LKL-DL---------LPVFSG--KAS--S-----------------ND-------
Os1bglu4        ---------P--VIY-V--------TENGM---------------DEE-DDQ--------------S--A-T-L-----------------DQ-------
Os1bglu5        ---------L--PIY-I--------LENGY---------------GS--------------------------S-----------------ND-------
Os3bglu6        ---------P--PVY-I--------TENGM---------------DDS-NNP--------------F--I-S-I-----------------KD-------
Os3bglu7        ---------P--TVV-I--------TENGM---------------DQP-AN------------------L-S-R-----------------DQ-------
Os3bglu8        ---------P--TII-I--------SENGM---------------DQS-GN------------------L-T-R-----------------EE-------
Os4bglu10       ---------P--TIY-I--------TENGV---------------DEI-NNK--------------T--M-R-L-----------------KE-------
Os4bglu11       ---------P--TIY-I--------TENGV---------------DEV-NNK--------------T--M-P-L-----------------EE-------
Os4bglu12       ---------P--TVY-I--------TENGV---------------DEF-NNK--------------T--L-P-L-----------------QE-------
Os4bglu13       ---------P--TIY-I--------TENGV---------------DEF-NNK--------------T--L-P-L-----------------QE-------
Os4bglu14       ---------T--PTY-I--------TENGY---------------SQA-SNS--------------N--M-T-A-----------------KD-------
Os4bglu16       ---------T--TIY-I--------TENGY---------------SQH-SDT-------------------S-M-----------------ED-------
Os4bglu18       ---------L--PMF-I--------TENGY---------------AQG-GDS--------------Y--T-D-A-----------------ED-------
Os4bglu27       ---------P--DIY-I--------AENGT---------------DEA-NNS--------------T--I-P-I-----------------AE-------
Os4bglu28       ---------P--TIY-I--------TENGT---------------DEA-NNS--------------T--I-P-I-----------------SE-------
Os4bglu9        --------------------------D-------------------------------------------------------------------------
Os5bglu19       ---------L--LFY-I--------QENGS---------------GS--------------------------------------------NA-------
Os5bglu21       ---------L--PIY-I--------QENGS---------------GS--------------------------S-----------------SE-------
Os5bglu22       ---------I--PVY-I--------QENGF---------------GHF-G--------------------K--D-----------------DD-------
Os5bglu23       ----------------I----------LGF---------------GQF----------------------D--K-----------------ED-------
Os5gblu20       ---------L--TIY-I--------QENGS---------------GA--------------------------P-----------------DG-------
Os6bglu24       ---------P--TIY-I--------TENGV---------------DEV-NNE--------------N--L-S-L-----------------KE-------
Os6bglu25       ---------P--PVV-I--------TENGM---------------DDA-NHP--------------F--S-R-L-----------------ED-------
Os7bglu26       ---------P--TMI-L--------SENGM---------------DQP-GN------------------V-S-I-----------------TQ-------
Os9bglu29       ---------P--IIY-V--------TENGI---------------AEG-NNK--------------S--L-P-I-----------------TE-------
Os9bglu30       ---------P--TIY-V--------TENGI---------------DEG-NNS--------------T------L-----------------PE-------
Os9bglu31       ---------P--IVM-I--------HENGA---------------AGQ-PD------------------PS--G-----------------GN-------
Os9bglu32       ---------P--PVM-I--------HENGD---------------ADS-PE------------------TP--G-----------------KI-------
Os9bglu33       ---------P--PVM-I--------HENGY---------------SDS-PD------------------IF--G-----------------KI-------
SB01G010825     ---------P--TII-I--------SENGM---------------DQP-GD------------------L-T-R-----------------EE-------
SB01G010830     ---------P--TVF-I--------TENGM---------------DQP-GN------------------L-T-R-----------------EQ-------
SB01G013360     ---------P--PVY-I--------TENGK---------------KQE-TNGNFPVDLGMDDSNSPF--I-S-L-----------------KD-------
SB01G043030     ---------P--TVY-I--------TENGM---------------DDG-NSP--------------F--T-S-I-----------------QD-------
SB02G028400     ---------P--VLY-V--------TENGI---------------AEE-NNR--------------T--I-P-L-----------------EV-------
SB02G029620     ---------P--PVM-I--------HENGA---------------ADR-PD------------------PS--G-----------------GN-------
SB02G041550     ---------P--TMI-L--------AENGM---------------DQP-GD------------------I-S-I-----------------TQ-------
SB03G037780     ---------L--PIY-I--------QETGY---------------AT--------------------------R-----------------NG-------
SB03G042690     ---------P--VIY-I--------TENGM---------------DDE-DDQ--------------S--A-T-L-----------------DQ-------
SB06G019830     ---------P--TIY-I--------TENGV---------------DEA-TNN--------------S--L-P-L-----------------QE-------
SB06G019840     ---------P--TVY-I--------TENGV---------------DEV-NNK--------------S--L-P-L-----------------QE-------
SB06G019850     ---------P--TVY-I--------TENGV---------------DEA-NNK--------------S--L-S-L-----------------EE-------
SB06G019860     ---------P--TVY-I--------TENGV---------------DEA-NNK--------------N--L-S-L-----------------EE-------
```

```
SB06G019880    ---------P--AIY-I--------TENGF---------------DEA-NNK--------------S--L-P-L-----------------QE-------
SB06G022410    ---------I--PLY-I--------TENGY---------------AQI-GNS--------------S--T-T-T-----------------EE-------
SB06G022420    ---------I--PLY-I--------TENGY---------------AQI-GNS--------------S--T-T-T-----------------EE-------
SB06G022450    ---------T--PMY-I--------TENGY---------------AQA-SNS--------------N--M-S-A-----------------KD-------
SB06G022460    ---------T--PIY-V--------TENGY---------------AQA-SNS--------------S--M-S-A-----------------KD-------
SB06G022490    ---------T--PVY-I--------TENGF---------------SQW-SDA-------------------N-R-----------------EG-------
SB06G022500    ---------L--PMF-I--------TENGY---------------AQG-GVG--------------Y--N-Q-V-----------------ED-------
SB06G022510    ---------L--PMF-I--------TENGY---------------AQG-GDS--------------Y--T-N-V-----------------ED-------
SB08G007586    ---------P--PIY-I--------TENGI---------------GDI-DKG--------------D--L-P-K-----------------AV-------
SB08G007650    ---------P--PMY-I--------TENGI---------------GDI-DKG--------------D--L-P-K-----------------AL-------
SB09G018160    ---------I--SIY-I--------QENGR---------------PD-------------------------------------------D-------
SB10G012220    ---------P--TIY-I--------TENGV---------------DEI-NNV--------------N--I-P-L-----------------QE-------
SB10G022300    ---------P--PVM-I--------HENGA---------------GHE-PD------------------PS--G-----------------A--------
SB10G027600    ---------P--PVI-I--------TENGM---------------DDA-NNR--------------F--S-R-L-----------------EN-------
SB10G028060    ---------P--VML-I--------GENGI---------------DQS-GN------------------E-T-L-----------------PH-------
SbDhr1         ---------P--PMY-I--------TENGM---------------GDI-DKG--------------D--L-P-K-----------------PV-------
SbDhr2         ---------P--PVY-I--------TENGM---------------GDI-DNG--------------D--L-S-M-----------------EA-------
Zm-p60.1       ---------P--PIY-I--------TENGI---------------GDV-DTK--------------ETPL-P-M-----------------ED-------
ZmGLU2         ---------P--PIY-I--------TENGI---------------GDV-DTK--------------EKPL-P-M-----------------EA-------
ZmGLU3         ---------P--PIY-I--------TENGI---------------GDV-DTK--------------DNPL-S-M-----------------QD-------
ZmGLU4         ---------P--PMY-I--------TENGM---------------AEV-D---------------------L-----------------GH-------
ZmGLU5         ---------P--PIY-I--------TENGM---------------GDV-DHG--------------D--L-P-M-----------------EV-------
ZmGLU6         ---------P--PIY-I--------TENGM---------------GDV-DNG--------------D--L-P-M-----------------ED-------
ZmGLU7         ---------P--PVY-I--------TENGI---------------GDV-DTK--------------DKPL-P-I-----------------KD-------
ZmGLU8         ---------P--PIY-I--------TENGI---------------GDI-DSK--------------EKPL-P-V-----------------KD-------
```

```
                          2010      2020      2030      2040      2050      2060      2070      2080      2090      2100
                    ....|....|....|....|....|....|....|....|....|....|....|....|....|....|....|....|....|....|....|....|
AC155376.2_FGT005   ----------------------------------------------------VLN------D--TKRVGYFKG---------------------------
AC211140.2_FGT007   ----------------------------------------------------LIS------Q--FDFLPSLAA---------------------------
AC217401.3_FGT001   ----------------------------------------------------YLH------D--TTRVQFYKG---------------------------
AC217687.3_FGT004   ----------------------------------------------------YLR------D--ATRVRFYRS---------------------------
AtBglu1             ----------------------------------------------------QQK------D--TPRIEYLDA---------------------------
AtBglu10            ----------------------------------------------------TLQ------D--TQRIEFIQA---------------------------
AtBglu11            ----------------------------------------------------SLV------D--TTRVKYLSS---------------------------
AtBglu12            ----------------------------------------------------DLK------D--SERIDYYAQ---------------------------
AtBglu13            ----------------------------------------------------DLK------D--SERIDYYAQ---------------------------
AtBglu14            ----------------------------------------------------LLK------D--GDRIDYYAR---------------------------
AtBglu15            ----------------------------------------------------FLK------D--GDRIDYYAR---------------------------
AtBglu16            ----------------------------------------------------FLN------D--DLRIDYYAH---------------------------
AtBglu17            ----------------------------------------------------ALN------D--EAKIKYHQL---------------------------
AtBglu18            ----------------------------------------------------GTQ------D--HNRKYYIQR---------------------------
AtBglu19            ----------------------------------------------------ALN------D--HNRKYYLQR---------------------------
AtBglu2             ----------------------------------------------------QQK------D--TRRIEYLQA---------------------------
AtBglu20            ----------------------------------------------------ALS------D--QHRTYYIQK---------------------------
AtBglu21            ----------------------------------------------------GTA------D--HNRKYYLQR---------------------------
AtBglu22            ----------------------------------------------------GTA------D--YNRKYYLQR---------------------------
AtBglu23            ----------------------------------------------------GTA------D--HNRKYYLQR---------------------------
AtBglu24            ----------------------------------------------------GTA------D--YNRESYLKK---------------------------
AtBglu25            ----------------------------------------------------ALS------D--SNRKYYHMR---------------------------
AtBglu26            ----------------------------------------------------PSI------D--TERLEYIEG---------------------------
AtBglu27            ----------------------------------------------------NLM------D--LQRTEYHKK---------------------------
AtBglu28            ----------------------------------------------------ILN------D--TFRISYHED---------------------------
AtBglu29            ----------------------------------------------------ILK------D--TFRISYHQD---------------------------
AtBglu3             ----------------------------------------------------QQK------D--TPRIEYLHA---------------------------
AtBglu30            ----------------------------------------------------IVK------D--TFRIEYHKT---------------------------
AtBglu31            ----------------------------------------------------ILE------D--TKRIEYHQK---------------------------
AtBglu32            ----------------------------------------------------IIE------D--TKRIEYHQN---------------------------
AtBglu33            ----------------------------------------------------ATN------D--YGRKEFIKS---------------------------
AtBglu34            ----------------------------------------------------ALA------D--NGRIQNHCS---------------------------
AtBglu35            ----------------------------------------------------ALA------D--NGRIQNHCS---------------------------
AtBglu36            ----------------------------------------------------ALA------D--NGRIQFQCS---------------------------
AtBglu37            ----------------------------------------------------AFH------D--YNRIDYLCS---------------------------
AtBglu38            ----------------------------------------------------ATA------D--YKRIDYLCS---------------------------
AtBglu39            ----------------------------------------------------------------------------------------------------
AtBglu4             ----------------------------------------------------KQK------D--IPRVEYLHA---------------------------
AtBglu40            ----------------------------------------------------ALK------D--AKRIKYHHD---------------------------
AtBglu41            ----------------------------------------------------ALK------D--DKRIGFHRD---------------------------
AtBglu42            ----------------------------------------------------MLD------D--KRRVDYFKS---------------------------
AtBglu43            ----------------------------------------------------GLN------D--TTRVKYYRD---------------------------
AtBglu44            ----------------------------------------------------GLH------D--TTRIKYYKD---------------------------
AtBglu45            ----------------------------------------------------LLN------D--TKRIQYMSG---------------------------
AtBglu46            ----------------------------------------------------LLH------D--TKRIQYLSG---------------------------
AtBglu47            ----------------------------------------------------LLN------D--YQRVKFMSN---------------------------
```

```
AtBglu5        ----------------------------------------------------QHK------D--THRVEYMNA---------------------------
AtBglu7        ----------------------------------------------------TLQ------D--TPRAEFIQA---------------------------
AtBglu8        ----------------------------------------------------MLQ------D--TPRVEYIQA---------------------------
AtBglu9        ----------------------------------------------------TLQ------D--TQRIEYIQA---------------------------
BD1G10890      ----------------------------------------------------YLH------D--ITRIRYYRS---------------------------
BD1G10920      ----------------------------------------------------YLH------D--ITRIRYYRS---------------------------
BD1G10930      ----------------------------------------------------YLR------D--VTRVRFYRS---------------------------
BD1G10940      ----------------------------------------------------FLH------D--TVRVEFYKN---------------------------
BD1G19270      ----------------------------------------------------GVH------D--TVRVRYYRE---------------------------
BD1G33040      ----------------------------------------------------VLQ------D--DERIQYHND---------------------------
BD1G42690      ----------------------------------------------------ALA------D--NTRIEFYRQ---------------------------
BD1G70170      ----------------------------------------------------ALK------D--SKRIKYHND---------------------------
BD2G09190      VSSRPAMSGPAPSKMITLQCFQRQKAVIMINDEVPFRDAIGPHQSTDISPAVGMR------E--TDRIQER-----------------------------
BD2G09200      ----------------------------------------------------------------------------------------------------
BD2G27770      ----------------------------------------------------SVN------D--HNRVEYLSG---------------------------
BD2G37450      -------------------------------------------------------------DGETRLVEYTDSNSEACSCVQLLVYRWPCMEKLTTFARK
BD2G57640      ----------------------------------------------------VLN------D--TKRVGFFKG---------------------------
BD2G59650      ----------------------------------------------------VLD------D--TDRLEYLKS---------------------------
BD2G59660      ----------------------------------------------------TID------D--TDRVEYLKT---------------------------
BD3G00650      ----------------------------------------------------ALK------D--RKRIKYHND---------------------------
BD3G40000      ----------------------------------------------------ALK------D--NTRIMFHYK---------------------------
BD3G40010      ----------------------------------------------------ALK------D--ETRIMFHYN---------------------------
BD3G45610      ----------------------------------------------------SLN------D--TYRVDCLSS---------------------------
BD3G45630      ----------------------------------------------------SLN------D--TDRVVYLSS---------------------------
BD3G45640      ----------------------------------------------------DLD------D--TDRVDYLSS---------------------------
BD3G45650      ----------------------------------------------------SLN------D--NDRVEYLSS---------------------------
BD4G08040      ----------------------------------------------------ALY------D--HFRIDYFDQ---------------------------
BD4G09920      -------------------------------------------------------------D--LIRKPYILE---------------------------
BD4G34930      ----------------------------------------------------ASSQEISNIS--LQKIKYTTR---------------------------
BD4G34940      ---------------------------------------------------YDYD------D--ANRTEFLQD---------------------------
BD4G34950      ----------------------------------------------------EFD------D--DYRSEYLQD---------------------------
BD5G13260      ----------------------------------------------------ALK------D--DARIEYHHK---------------------------
BD5G13270      ----------------------------------------------------SLM------D--DARVEYHHK---------------------------
BD5G15530      ----------------------------------------------------FIN------D--AGRIHYIRG---------------------------
BD5G15540      ----------------------------------------------------LIN------D--VGRVNYLQG---------------------------
GRMZM2G012236  ----------------------------------------------------IIN------D--TERINYIRD---------------------------
GRMZM2G015804  ----------------------------------------------------ALK------D--SKRIKYHNG---------------------------
GRMZM2G031660  ----------------------------------------------------WLD------D--QGRIQYLEG---------------------------
GRMZM2G055699  ----------------------------------------------------SIH------D--HERAEYLSA---------------------------
GRMZM2G069024  ----------------------------------------------------SLH------D--TDRVDYMKT---------------------------
GRMZM2G108133  ----------------------------------------------------SYD------D--EFRSQFLQD---------------------------
GRMZM2G110948  ----------------------------------------------------GVR------D--AARVAYYRS---------------------------
GRMZM2G112704  ----------------------------------------------------ALY------D--KFRIDYFEK---------------------------
GRMZM2G128197  ----------------------------------------------------YVH------D--AVRIDFYKN---------------------------
GRMZM2G148176  ----------------------------------------------------GVH------D--TVRIRYYRD---------------------------
GRMZM2G163544  ----------------------------------------------------DLQ------D--DKRIQYHKD---------------------------
GRMZM2G177661  ----------------------------------------------------ALK------D--SKRVKYHND---------------------------
GRMZM2G376416  ---------------------------------------------------FLYD------D--EFRAHFLRV---------------------------
```

```
GRMZM2G457040    ---------------------------------------------------LID------D--VRRKNYLQG---------------------------
Os10bglu34       ---------------------------------------------------ALK------D--DKRTKYHND---------------------------
Os11bglu36       ---------------------------------------------------         D--LIRKPYILE---------------------------
Os12bglu38       ---------------------------------------------------ALY------D--FFRIQYFDQ---------------------------
Os1bglu1         ---------------------------------------------------GVH------D--TTRVAYYRS---------------------------
Os1bglu2         ---------------------------------------------------SLD------D--PDRVDYIKG---------------------------
Os1bglu3         ---------------------------------------------------SLD------D--TDRVDYIKG---------------------------
Os1bglu4         ---------------------------------------------------VLN------D--TTRVGYFKG---------------------------
Os1bglu5         ---------------------------------------------------TVH------D--NDRVDYLKS---------------------------
Os3bglu6         ---------------------------------------------------ALK------D--SKRIKYHND---------------------------
Os3bglu7         ---------------------------------------------------YLR------D--TTRVHFYRS---------------------------
Os3bglu8         ---------------------------------------------------FLH------D--TERIEFYKN---------------------------
Os4bglu10        ---------------------------------------------------ALK------D--DIRIEYYHK---------------------------
Os4bglu11        ---------------------------------------------------ALK------D--DTRIEYYHK---------------------------
Os4bglu12        ---------------------------------------------------ALK------D--DARIEYYHK---------------------------
Os4bglu13        ---------------------------------------------------ALK------D--DTRIDYYHK---------------------------
Os4bglu14        ---------------------------------------------------FTN------D--TGRITYIQG---------------------------
Os4bglu16        ---------------------------------------------------LIN------D--VERVNYMHD---------------------------
Os4bglu18        ---------------------------------------------------WID------D--EDRIEYLEG---------------------------
Os4bglu27        ---------------------------------------------------ALK------D--DNRISFHYQ---------------------------
Os4bglu28        ---------------------------------------------------ALK------D--ETRIGFHYK---------------------------
Os4bglu9         ---------------------------------------------------------------------------------------------------
Os5bglu19        ---------------------------------------------------TLD------D--VGRIDCLTQ---------------------------
Os5bglu21        ---------------------------------------------------TLD------D--VERINYLAK---------------------------
Os5bglu22        ---------------------------------------------------SLN------D--TDRVDYLSS---------------------------
Os5bglu23        ---------------------------------------------------SLN------D--TERVEYLSS---------------------------
Os5gblu20        ---------------------------------------------------TLD------D--VERINYLQK---------------------------
Os6bglu24        ---------------------------------------------------ALI------D--TTRIEFYRQ---------------------------
Os6bglu25        ---------------------------------------------------VLQ------D--DKRIQYHND---------------------------
Os7bglu26        ---------------------------------------------------GVH------D--TVRIRYYRN---------------------------
Os9bglu29        ---------------------------------------------------ALK------D--GHRIEFHSK---------------------------
Os9bglu30        ---------------------------------------------------ALK------D--GHRIEFHSK---------------------------
Os9bglu31        ---------------------------------------------------TYD------D--DFRSQYLQD---------------------------
Os9bglu32        ---------------------------------------------------DYD------D--DFRSDFLQS---------------------------
Os9bglu33        ---------------------------------------------------NYN------D--DFRSAFLQG---------------------------
SB01G010825      ---------------------------------------------------YLH------D--TVRIDFYKN---------------------------
SB01G010830      ---------------------------------------------------YLH------D--TTRVQFYKG---------------------------
SB01G013360      ---------------------------------------------------AVK------D--DKRISYHNE---------------------------
SB01G043030      ---------------------------------------------------ALK------D--KKRIKYHND---------------------------
SB02G028400      ---------------------------------------------------ALR------D--GHRIRFHSQ---------------------------
SB02G029620      ---------------------------------------------------SYD------D--EFRSQFLQD---------------------------
SB02G041550      ---------------------------------------------------GVH------D--TIRIRYYRD---------------------------
SB03G037780      ---------------------------------------------------SLH------D--TDRVDYMKN---------------------------
SB03G042690      ---------------------------------------------------VLN------D--TTRVGYFKG---------------------------
SB06G019830      ---------------------------------------------------ALK------D--DIRIEYYHK---------------------------
SB06G019840      ---------------------------------------------------ALK------D--STRIEYYHK---------------------------
SB06G019850      ---------------------------------------------------ALK------D--DTRIEFHHA---------------------------
SB06G019860      ---------------------------------------------------ALK------D--NTRIEFYHT---------------------------
```

```
SB06G019880        ----------------------------------------------------ALK------D--DTRIEYHHK---------------------------
SB06G022410        ----------------------------------------------------LIN------D--TERSSYIRD---------------------------
SB06G022420        ----------------------------------------------------LIN------D--TERSSYIHD---------------------------
SB06G022450        ----------------------------------------------------FTN------D--TGRVDYLQG---------------------------
SB06G022460        ----------------------------------------------------FTN------D--TGRVNYLQG---------------------------
SB06G022490        ----------------------------------------------------LIN------D--VARKNYLQG---------------------------
SB06G022500        ----------------------------------------------------WLD------D--QSRIQYLDG---------------------------
SB06G022510        ----------------------------------------------------WLD------D--QGRIQYLDG---------------------------
SB08G007586        ----------------------------------------------------ALE------D--HTRLDYIQR---------------------------
SB08G007650        ----------------------------------------------------ALE------D--HTRLDYIQR---------------------------
SB09G018160        ----------------------------------------------------SLM------D--VDRIDFLKV---------------------------
SB10G012220        ----------------------------------------------------ALV------D--NTRIEFYRQ---------------------------
SB10G022300        ---------------------------------------------------FLYD------D--EFRAHFLQV---------------------------
SB10G027600        ----------------------------------------------------DLQ------D--DKRIQYHND---------------------------
SB10G028060        ----------------------------------------------------ALY------D--KFRIDYFQK---------------------------
SbDhr1             ----------------------------------------------------ALE------D--HTRLDYIQR---------------------------
SbDhr2             ----------------------------------------------------ALD------D--HIRLDYLQR---------------------------
Zm-p60.1           ----------------------------------------------------ALN------D--YKRLDYIQR---------------------------
ZmGLU2             ----------------------------------------------------ALN------D--YKRLDYIQR---------------------------
ZmGLU3             ----------------------------------------------------ALE------D--YKRLDYLQR---------------------------
ZmGLU4             ----------------------------------------------------NLN------D--QKRIDYLQS---------------------------
ZmGLU5             ----------------------------------------------------ALD------D--HKRVHYLQR---------------------------
ZmGLU6             ----------------------------------------------------ALN------D--QKRINYLQR---------------------------
ZmGLU7             ----------------------------------------------------ALN------D--HVRLDYLQR---------------------------
ZmGLU8             ----------------------------------------------------ALN------D--HARLDYLQR---------------------------
```

```
                          2110      2120      2130      2140      2150      2160      2170      2180      2190      2200
                      ....|....|....|....|....|....|....|....|....|....|....|....|....|....|....|....|....|....|....|....|
AC155376.2_FGT005     -------YLNSV--AQAI-K-DGADVR------------------------------------------------------GYFAWSFLDNFEWAM-GYT
AC211140.2_FGT007     -------YMPDIWERDAVRREDGCDVR------------------------------------------------------GYFAWSLLDNWEWAA-GYT
AC217401.3_FGT001     -------YLAEL--RKAI-D-DGADVA------------------------------------------------------GYFAWSLLDNFEWQS-GYT
AC217687.3_FGT004     -------YIGQL--KKAI-D-QGANVA------------------------------------------------------GYFAWSLLDNFEWLA-GYS
AtBglu1               -------YIGAV--LKAV-R-NGSDTR------------------------------------------------------GYFVWSFMDLYELLN-GYK
AtBglu10              -------YIGAM--LNAI-K-NGSDTR------------------------------------------------------GYFVWSMIDLYELLS-GYT
AtBglu11              -------YIKAV--LHSLSR-KGSDVK------------------------------------------------------GYFQWSLMDVFELFG-GYE
AtBglu12              -------HLKMV--QDAI-S-IGANVK------------------------------------------------------GFFAWSLLDNFEWAT-GYA
AtBglu13              -------HLKMV--QDAI-S-IGANVK------------------------------------------------------GFFAWSLLDNFEWAT-GYS
AtBglu14              -------HLKMV--QDAI-L-IGANVK------------------------------------------------------GFFAWSLLDNFEWAS-GYT
AtBglu15              -------HLEMV--QDAI-S-VGANVK------------------------------------------------------GFFAWSLLDNFEWAM-GYT
AtBglu16              -------HLKMV--SDAI-S-IGVNVK------------------------------------------------------GYFAWSLMDNFEWSE-GYT
AtBglu17              -------HLTAL--LEAV-S-QGADVR------------------------------------------------------GYYIWSLMDDFEWEF-GYK
AtBglu18              -------HLLSM--HDAI-CKDKVNVT------------------------------------------------------GYFVWSLMDNFEWQD-GYK
AtBglu19              -------HLLAL--NEAI-CEDKVNVT------------------------------------------------------SYFLWSLMDNFEWQD-GYT
AtBglu2               -------YIGAV--LKAV-R-NGSDTR------------------------------------------------------GYF----------------
AtBglu20              -------HLLSL--HEAI-CDDKVNVT------------------------------------------------------GYFHWSLMDNFEWQD-GYK
AtBglu21              -------HLLAM--NEAI-CIDKVRVT------------------------------------------------------GYFVWSLLDNFEWQD-GYK
AtBglu22              -------HLLAM--NEAI-CIDKVRVT------------------------------------------------------GYFVWSLLDNFEWQD-GYN
AtBglu23              -------HLLSM--QEAV-CIDKVNVT------------------------------------------------------GYFVWSLLDNFEWQD-GYK
AtBglu24              -------HLWSM--HKAI-CEDKVNVT------------------------------------------------------GYFVWSLMDNFEWQD-GFK
AtBglu25              -------HLMAL--HGAV-CEDKVNVK------------------------------------------------------GYFVSSLMDGLEWED-GYK
AtBglu26              -------HIHAI--HQAI-HEDGVRVE------------------------------------------------------GYYVWSLLDNFEWNS-GYG
AtBglu27              -------HLQSI--QQAI-QEDGVVVE------------------------------------------------------GYFAWSLLDNCEWNA-GYG
AtBglu28              -------HLQQL--QKAI-IEDGCDVR------------------------------------------------------GYYVWSLLDNFEWEH-GYS
AtBglu29              -------HLKQV--HKAI-IEDGCDVR------------------------------------------------------GYYVWSLFDNFEWEH-GYN
AtBglu3               -------YIAAV--LKSI-R-NGSDTR------------------------------------------------------GYFIWSFMDLYELVK-GYE
AtBglu30              -------HFEEL--HKAI-VEDGCDVR------------------------------------------------------GYYAWSLMDNFEWEH-GYT
AtBglu31              -------HLQEL--QKAI-TEDGCDVK------------------------------------------------------GYFTWSLLDNFEWEH-GYA
AtBglu32              -------HLQQL--QKAI-TEDGCNVK------------------------------------------------------GYFTWSLLDNFEWEH-GYA
AtBglu33              -------HILIM--GKSI-RMDKVRLK------------------------------------------------------GYYIWSLMDNFEWDK-GYK
AtBglu34              -------HLSCL--KCAM-K-DGCNVA------------------------------------------------------GYFAWSLMDNYEFGN-GYT
AtBglu35              -------HLSCL--KCAM-K-DGCNVA------------------------------------------------------GYFAWSLMDNYEFGN-GYT
AtBglu36              -------HLSCL--KCAI-E-DGCNVA------------------------------------------------------GYFAWSLMDNYEFGN-GYT
AtBglu37              -------HLCFL--RKAI-KEKRVNVK------------------------------------------------------GYFVWSLGDNYEFCN-GYT
AtBglu38              -------HLCFL--SKVI-KEKNVNVK------------------------------------------------------GYFAWSLGDNYEFCN-GFT
AtBglu39              ----------------LI-L-SGCNVK------------------------------------------------------GYFAWCLGDNYELWP-SRS
AtBglu4               -------YIGGV--LKSI-R-NGSDTR------------------------------------------------------GYFVWSFMDLYELLG-GYE
AtBglu40              -------YLSSL--QASI-KEDGCNVK------------------------------------------------------GYFVWSLLDNWEWAA-GYS
AtBglu41              -------YLSNL--SAAI-RNDECDVR------------------------------------------------------GYFVWSLLDNWEWNS-GYT
AtBglu42              -------YLANV--SQAI-E-DGVDIK------------------------------------------------------GYFAWSLLDNFEWAQ-GYT
AtBglu43              -------YLVQL--KKAV-D-DGANLT------------------------------------------------------GYFAWSLLDNFEWLS-GYT
AtBglu44              -------YLTNL--KKAR-D-DGANVV------------------------------------------------------GYFAWSLLDNFEWLS-GYT
AtBglu45              -------YLEAL--QAAM-R-DGANVK------------------------------------------------------GYFVWSLLDNFEWLF-GYK
AtBglu46              -------YLDAL--KAAM-R-DGANVK------------------------------------------------------GYFAWSLLDNFEWLY-GYK
AtBglu47              -------YLDAL--KRAM-R-KGADVR------------------------------------------------------GYFAWSLLDNFEWIS-GYT
```

```
AtBglu5        -------YIGGV--LKSI-R-NGSDTR-------------------------------------------------------GYFVWSFMDLFELIG-RYD
AtBglu7        -------YIGAV--HNAI-T-NGSDTR-------------------------------------------------------GYFVWSMIDLYELIG-RYM
AtBglu8        -------YIGAV--LNAI-K-SGSDTR-------------------------------------------------------GYFVWSLIDLFEVQV-GYK
AtBglu9        -------YIDAV--LNAM-K-NGSDTR-------------------------------------------------------GYFVWSMVDVYEILS-GYT
BD1G10890      -------YLAEL--KRAI-D-GGANVL-------------------------------------------------------GYFAWSLLDNFN-------
BD1G10920      -------YLAEL--KRAI-D-GGANVL-------------------------------------------------------GYFAWSLLDNFEWIS-GYT
BD1G10930      -------YLAEL--KKAI-D-GGANVL-------------------------------------------------------GYFAWSLLDNFEWGS-GYT
BD1G10940      -------YLSEL--KKAI-D-DGANVV-------------------------------------------------------AYFAWSLLDNFEWLS-GYT
BD1G19270      -------YITEL--KKVI-D-DGARVI-------------------------------------------------------GYFAWSLLDNFEWRL-GYT
BD1G33040      -------YMSNL--LDAI-RKEGCNVH-------------------------------------------------------GYFVWSLLDNWEWNS-GYT
BD1G42690      -------HIFHV--LRAL-R-EGVDVR-------------------------------------------------------GYFAWSLFDNFEWMD-GYS
BD1G70170      -------YLTNL--AASI-KEDGCDVR-------------------------------------------------------GYFAWSLLDNWEWAA-GYS
BD2G09190      --------I-------------SCQ--RIGSLHWCKTYPIFVHLSAWPTCRSLMRTNGGSHWRTSYFLHPSFVQTPPNGQMGALPWRCPKGPAYVS-NT-
BD2G09200      -----------------------------------------------------------------------------------------------------
BD2G27770      -------YIGST--LTAL-R-NGANVK-------------------------------------------------------GYFVWSFLDVFELLA-GYY
BD2G37450      DLHPETVFIFVT--PDAS-S-SAEAVR-------------------------------------------------------TLCIW--------IGGEYE
BD2G57640      -------YVGAV--AQAI-K-DGADIR-------------------------------------------------------GYFAWSFLDNFEWAM-GYT
BD2G59650      -------YIGSA--LAAV-R-NGANLK-------------------------------------------------------GYFVWSFLDLFEFLG-GYN
BD2G59660      -------FIEST--LDAI-K-DGANVK-------------------------------------------------------GYFVWSFLDVFEVLS-GYK
BD3G00650      -------YMTNL--ADSI-REDGCDVR-------------------------------------------------------GYFVWSLLDNWEWTA-GYT
BD3G40000      -------HLEFV--YRAI-R-YGG----------------------------------------------------------------------------R
BD3G40010      -------HLKFV--HKAI-Q-YGG----------------------------------------------------------------------------R
BD3G45610      -------YMGST--LDAI-R-NGVNVR-------------------------------------------------------GYFTWAFMDLFELRA-GYQ
BD3G45630      -------YMGST--LDAI-R-NGVNVR-------------------------------------------------------GYFAWAFMDLFELLA-GYQ
BD3G45640      -------YMGST--LDAI-R-NGVNVR-------------------------------------------------------GYFTWAFMDLFELLA-GYQ
BD3G45650      -------YMRST--LDAI-R-NGVNVR-------------------------------------------------------GYFVWAFKDLFELLA-GYQ
BD4G08040      -------YLHEL--KRAI-D-DGARVV-------------------------------------------------------GYFAWSLLDNFEWRM-GFT
BD4G09920      -------HLLAI--YAAI-L-VGVRVL-------------------------------------------------------GYLFWTTSDNWEWAD-GYG
BD4G34930      -------HLIRS----------------------------------------------------------------------------WCSYQELRA-CLT
BD4G34940      -------YLEVL--QLST-R-NGSNAR-------------------------------------------------------GYFVWSFLDVFEFLF-GYQ
BD4G34950      -------YLEVL--YQSI-R-NGSDAR-------------------------------------------------------GYFVWSFLDVFELLF-GYA
BD5G13260      -------HLLAL--LSAI-R-DGANVK-------------------------------------------------------GYFAWSLLDNFEWAS-GYT
BD5G13270      -------HLDIL--QSAI-R-DGANVK-------------------------------------------------------GYFAWSLLDNFEWVY-GYT
BD5G15530      -------YLTFL--ASAI-R-KGADIR-------------------------------------------------------GYFVWSLLDDFEWTS-GYT
BD5G15540      -------YLTSI--SSAV-R-KGANVR-------------------------------------------------------GYFVWSLIDNFEWGF-GYT
GRMZM2G012236  -------YLTYL--SFAI-R-KGADVR-------------------------------------------------------GYFVWSLMDSFEWIS-GYT
GRMZM2G015804  -------YLNNV--AASI-KEDGCDVR-------------------------------------------------------GYFAWSLLDNWEWAA-GYT
GRMZM2G031660  -------YLAKL--AKVI-S-DGADVR-------------------------------------------------------GYFIWSLIDNFEWLY-GYT
GRMZM2G055699  -------YMGSA--LAAL-R-NGANVK-------------------------------------------------------GYFVWSFLDVFELLA-GYY
GRMZM2G069024  -------HISST--LAAL-R-NGANVK-------------------------------------------------------GYFAWCFLDVFEYLS-GFM
GRMZM2G108133  -------YIEAT--LQSI-R-NGSNVQ-------------------------------------------------------GYFVWSFLDVFEYLF-GYR
GRMZM2G110948  -------YVAEL--KAAI-D-GGANCV-------------------------------------------------------GYFAWSLLDNFEWKL-GYT
GRMZM2G112704  -------YLYEL--QCAI-R-DGANVF-------------------------------------------------------GYFAWSLLDNFEWRL-GFT
GRMZM2G128197  -------YLTEL--KRGI-D-GGANVI-------------------------------------------------------GYFAWSLLDNFEWLS-GYT
GRMZM2G148176  -------YITEL--KKAI-D-DGARVI-------------------------------------------------------GYFAWSLLDNFEWRL-GYT
GRMZM2G163544  -------YMSNL--LDAI-RKEGCNVH-------------------------------------------------------GYFVWSLLDNWEWNS-GYT
GRMZM2G177661  -------YLTNL--AASI-KDDACDVR-------------------------------------------------------GYFAWSLLDNWEWTA-GYS
GRMZM2G376416  -------YVEAA--LASV-R-NGSDLR-------------------------------------------------------GYFVWSFMDVFEFLF-SYR
```

```
GRMZM2G457040   -------YITYL--SKAV-R-NGANVR------------------------------------------------------GYFVWTLLDNFEWAF-GYR
Os10bglu34      -------YLTNL--ADSI-REDGCDVR------------------------------------------------------GYFAWSLLDNWEWAA-GYT
Os11bglu36      -------HLLAT--YAAI-I-MGVRVL------------------------------------------------------GYLFWTTSDNWEWAD-GYG
Os12bglu38      -------YLHEL--KRAI-K-DGARVT------------------------------------------------------GYFAWSLLDNFEWRL-GFT
Os1bglu1        -------YITKL--KEAI-D-DGANCI------------------------------------------------------GYFAWSLLDNFEWKL-GYT
Os1bglu2        -------YIGGV--LDAI-R-NGVDVR------------------------------------------------------GYFVWSFVDVYELLE-GYQ
Os1bglu3        -------YIEGV--LNAT-R-NGVNAR------------------------------------------------------GYFAWFFVDMFELLS-GYQ
Os1bglu4        -------YLASV--AQAI-K-DGADVR------------------------------------------------------GYFAWSFLDNFEWAM-GYT
Os1bglu5        -------YIGSI--LTAL-R-NGANVK------------------------------------------------------GYFVWSFVDVFEYLT-GYG
Os3bglu6        -------YLTNL--AASI-KEDGCDVR------------------------------------------------------GYFAWSLLDNWEWAA-GYS
Os3bglu7        -------YLTQL--KKAI-D-EGANVA------------------------------------------------------GYFAWSLLDNFEWLS-GYT
Os3bglu8        -------YLTEL--KKAI-D-DGANVV------------------------------------------------------AYFAWSLLDNFEWLS-GYT
Os4bglu10       -------HLLAL--LSAM-R-DGANVK------------------------------------------------------GYFAWSLLDNFEWSE-GYT
Os4bglu11       -------HLLAL--LSAM-R-DGANVK------------------------------------------------------GYFAWSLLDNFEWAE-GYT
Os4bglu12       -------HLLSL--LSAI-R-DGANVK------------------------------------------------------GYFAWSLLDNFEWSN-GYT
Os4bglu13       -------HLLSL--LSAI-R-DGANVK------------------------------------------------------GYFAWSLLDNFEWSN-GYT
Os4bglu14       -------YLISL--ASAI-R-KGADVR------------------------------------------------------GYFVWSLLDDFEWNF-GYT
Os4bglu16       -------YLKYL--SSAI-R-KGANVG------------------------------------------------------GYFAWSIVDNFEWVY-GYT
Os4bglu18       -------YLTKL--AKVI-R-DGADVR------------------------------------------------------GYFAWSVVDNFEWLF-GYT
Os4bglu27       -------HLRFT--QLAI-K-EGVKVK------------------------------------------------------GYFTWTFMDDFEWGD-GYT
Os4bglu28       -------HLQFV--HKAI-Q-EGVKVK------------------------------------------------------GYFTWTFMDCFEFGD-GFK
Os4bglu9        ----------------------------------------------------------------------------------------------------
Os5bglu19       -------YIAAT--LRSI-R-NGANVK------------------------------------------------------GYCVWSFMDQYEMFG-DYK
Os5bglu21       -------YIAAT--LKAI-R-SGANVK------------------------------------------------------GYSMWSFVDLYELFG-GYS
Os5bglu22       -------YMGST--LAAL-R-NGANVK------------------------------------------------------GYFVWSFLDVFELLA-GYH
Os5bglu23       -------YMGGT--LAAL-R-NGANVK------------------------------------------------------GYFVWSFLDVFELFA-GYH
Os5gblu20       -------YIAAT--LKAI-R-NGANVK------------------------------------------------------GYSMWSFIDIYEIFG-GYN
Os6bglu24       -------HLFHV--QRAL-R-QGVDVR------------------------------------------------------GYFAWSLFDNFEWMD-GYS
Os6bglu25       -------YMSNL--LDAI-RKEGCNVH------------------------------------------------------GYFVWSLLDNWEWNS-GYT
Os7bglu26       -------YITEL--KKAI-D-DGAKVI------------------------------------------------------GYFAWSLLDNFEWRL-GYT
Os9bglu29       -------HLQFV--NHAI-K-NGVNVK------------------------------------------------------GYFTWTFMDCFEWGD-GYL
Os9bglu30       -------HLQFV--NHAI-K-NGVNVK------------------------------------------------------GYFTWTFMDCFEWGD-GYL
Os9bglu31       -------YIEAT--LQSI-R-NGSNVQ------------------------------------------------------GYFVWSFLDVFEYLF-GYR
Os9bglu32       -------YLEVL--HLSI-R-CAHSPCLLLSIPYCKERIEHAGILRVVTA-RRVRV--PLR-------------------LRQPLWPVLRRLHRAG-EDE
Os9bglu33       -------YLEAL--YLSV-R-NGSNTR------------------------------------------------------GYFVWSMFDMFEFLY-GYR
SB01G010825     -------YLTEL--KKGI-D-DGANVV------------------------------------------------------GYFAWSLLDNFEWLS-GYT
SB01G010830     -------YLAEL--KKAI-D-DGANVA------------------------------------------------------GYFAWSLLDNFEWQS-GYT
SB01G013360     -------YLTNL--AASI-REDGCDVR------------------------------------------------------GYFVWSLLDNWEWTA-GYT
SB01G043030     -------YLSNV--AASI-KEDGCDVR------------------------------------------------------GYFAWSLLDNWEWAA-GYS
SB02G028400     -------HLQFV--NHAI-R-DGVNVK------------------------------------------------------GYFTWTFMDCFEWGD-GYL
SB02G029620     -------YIEAT--LESI-R-DGSNVR------------------------------------------------------GYFVWSFLDMFEFLF-GYR
SB02G041550     -------YITEL--KKAI-D-DGARVI------------------------------------------------------GYFAWSLLDNFEWRL-GYT
SB03G037780     -------HINST--LTAL-R-NGANVK------------------------------------------------------GYFAWCFLDVFEYLT-GFS
SB03G042690     -------YLNSV--AQAI-K-DGADIR------------------------------------------------------GYFAWSFLDNFEWAM-GYT
SB06G019830     -------HLLAL--SSAI-R-AGANVK------------------------------------------------------GYFAWSLLDNFEWRD-AFT
SB06G019840     -------HLLAL--QSAI-S-DGANVK------------------------------------------------------GYFAWSLLDNFEWVN-GYT
SB06G019850     -------HFLAL--QSAI-R-DGANVK------------------------------------------------------GYFAWSLLDNFEWAS-GYT
SB06G019860     -------HLLAL--QSAI-R-DGANVK------------------------------------------------------GYFPWSLLDNFEWAN-GYT
```

```
SB06G019880    -------HLLAL--LSAI-R-DGANVK------------------------------------------------------AYFAWSLMDNFEWVN-GYT
SB06G022410    -------YLTYL--SFAI-R-KGADVR------------------------------------------------------GYFVWSLMDNFEWLS-GYT
SB06G022420    -------YLTYL--SLAI-R-KGADVR------------------------------------------------------GYFVWSLMDNFEWLS-GYT
SB06G022450    -------YLTFL--ASAI-R-KGADVR------------------------------------------------------GYFVWSLLDNFEWNS-GYT
SB06G022460    -------YLTSL--ASAI-R-KGADVH------------------------------------------------------GYFVWSLLDNFEWNN-GYT
SB06G022490    -------YVTCL--SKAV-R-NGANVR------------------------------------------------------GYFVWTLLDNFEWTF-GYT
SB06G022500    -------YLTKL--AKVI-R-DGADVR------------------------------------------------------GYFIWSLIDNFEWTY-GYT
SB06G022510    -------YLTKL--AKVI-R-DGADVR------------------------------------------------------GYFVWSLIDNFEWLY-GYT
SB08G007586    -------HLSVL--KQSI-D-LGADVR------------------------------------------------------GYFAWSLLDNFEWSS-GYT
SB08G007650    -------HLSVL--KQSI-D-LGANVR------------------------------------------------------GYFAWSLLDNFEWSS-GYT
SB09G018160    -------YIAST--LKAI-R-DGADVK------------------------------------------------------GYSVWSLLDLYEMFG-GYK
SB10G012220    -------HLFHI--KRAL----------------------------------------------------------------------------------
SB10G022300    -------YIRAA--LGSV-K-NGSDVR------------------------------------------------------GYFVWSFMDVFEYLF-AYR
SB10G027600    -------YMSNL--LDAI-RKEGCNVH------------------------------------------------------GYFVWSLLDNWEWNS-GYT
SB10G028060    -------YLQEL--QYAI-R-DGANVF------------------------------------------------------GYFVWSLLDNFEWRL-GYT
SbDhr1         -------HLSVL--KQSI-D-LGADVR------------------------------------------------------GYFAWSLLDNFEWSS-GYT
SbDhr2         -------HISVL--KDSI-D-SGANVR------------------------------------------------------GHFTWSLLDNFEWSS-GYT
Zm-p60.1       -------HIATL--KESI-D-LGSNVQ------------------------------------------------------GYFAWSLLDNFEWFA-GFT
ZmGLU2         -------HISTL--KESI-D-LGANVH------------------------------------------------------GYFAWSLLDNFEWYA-GYT
ZmGLU3         -------HISVI--KESI-D-LGADVR------------------------------------------------------GHFTWSLLDNFEWSS-GYT
ZmGLU4         -------HLAAL--QDSV-E-SGANVK------------------------------------------------------GYFLWSLLDNFEWFC-GYT
ZmGLU5         -------HIATL--KESR-D-LGANVQ------------------------------------------------------GYFAWSLLDNFEWFS-GYT
ZmGLU6         -------HIAVI--KDSM-E-LGADVR------------------------------------------------------GYFAWSLVDNFEWTA-GYT
ZmGLU7         -------HISVL--KDAI-D-LGADVR------------------------------------------------------GHFTWSLLDNFEWSA-GYT
ZmGLU8         -------HISVL--KDAI-D-LGADVR------------------------------------------------------GHFTWSLLDNFEWCG-GYT
```

```
                        2210      2220      2230      2240      2250      2260      2270      2280      2290      2300
                   ....|....|....|....|....|....|....|....|....|....|....|....|....|....|....|....|....|....|....|....|
AC155376.2_FGT005  K-RFGIVYVDYKD---------------------------------------------------------------------------------------
AC211140.2_FGT007  S-RFGLYFVDYKD---------------------------------------------------------------------------------------
AC217401.3_FGT001  S-KFGIVYVDFSTP--------------------------------------------------------------------------------------
AC217687.3_FGT004  S-KFGIVYVDFNT---------------------------------------------------------------------------------------
AtBglu1            S-SFGLYSVNFSDP--------------------------------------------------------------------------------------
AtBglu10           T-SFGMYYVNFSDP--------------------------------------------------------------------------------------
AtBglu11           R-SFGLLYVDFKDP--------------------------------------------------------------------------------------
AtBglu12           V-RFGLVYVDFNG---------------------------------------------------------------------------------------
AtBglu13           V-RFGLVYVDFND---------------------------------------------------------------------------------------
AtBglu14           V-RFGLVYVDFND---------------------------------------------------------------------------------------
AtBglu15           V-RFGLVYVDFKD---------------------------------------------------------------------------------------
AtBglu16           V-RFGLVFVDFED---------------------------------------------------------------------------------------
AtBglu17           Y-RYGLVYVDFQD---------------------------------------------------------------------------------------
AtBglu18           A-RFGLYYIDFQN---------------------------------------------------------------------------------------
AtBglu19           A-RFGVYYIDFKN---------------------------------------------------------------------------------------
AtBglu2            ----------------------------------------------------------------------------------------------------
AtBglu20           A-RFGLYYVDYKN---------------------------------------------------------------------------------------
AtBglu21           N-RFGLYYVDFKN---------------------------------------------------------------------------------------
AtBglu22           N-RFGLYYVDFKN---------------------------------------------------------------------------------------
AtBglu23           N-RFGLYYVDFKN---------------------------------------------------------------------------------------
AtBglu24           N-RFGLYYIDYKN---------------------------------------------------------------------------------------
AtBglu25           T-RSGLYYVDYGH---------------------------------------------------------------------------------------
AtBglu26           V-RYGLYYIDYKD---------------------------------------------------------------------------------------
AtBglu27           V-RYGLFYVDYNN---------------------------------------------------------------------------------------
AtBglu28           T-RFGVYYVDYDN---------------------------------------------------------------------------------------
AtBglu29           S-RFGMYYVDFKN---------------------------------------------------------------------------------------
AtBglu3            F-SFGLYSVNFSDP--------------------------------------------------------------------------------------
AtBglu30           A-RFGLYYVDFVN---------------------------------------------------------------------------------------
AtBglu31           V-RFGLYYVDYKN---------------------------------------------------------------------------------------
AtBglu32           V-RFGLYYVDYKN---------------------------------------------------------------------------------------
AtBglu33           V-RFGLYYVDYND---------------------------------------------------------------------------------------
AtBglu34           L-RFGMNWVNFTN---------------------------------------------------------------------------------------
AtBglu35           L-RFGMNWVNFTN---------------------------------------------------------------------------------------
AtBglu36           L-RFDMNWVNFTN---------------------------------------------------------------------------------------
AtBglu37           V-RFGLSYVDFNNV--------------------------------------------------------------------------------------
AtBglu38           V-RFGLSYVDFANI--------------------------------------------------------------------------------------
AtBglu39           F-HVSPFYLLHRKD--------------------------------------------------------------------------------------
AtBglu4            V-GFGLYTVNFSDP--------------------------------------------------------------------------------------
AtBglu40           S-RFGLYFVDYRD---------------------------------------------------------------------------------------
AtBglu41           V-RFGIYYVDYKN---------------------------------------------------------------------------------------
AtBglu42           K-RFGLVYVDYKN---------------------------------------------------------------------------------------
AtBglu43           S-RFGIVYVDYKD---------------------------------------------------------------------------------------
AtBglu44           S-RFGIVYVDYKT---------------------------------------------------------------------------------------
AtBglu45           V-RFGLFHVDLTT---------------------------------------------------------------------------------------
AtBglu46           V-RFGLFHVDFTT---------------------------------------------------------------------------------------
AtBglu47           I-RFGMYHVDFST---------------------------------------------------------------------------------------
```

```
AtBglu5         Y-GYGLYSVNFSDP--------------------------------------------------------------------------------------
AtBglu7         T-SYGMYYVNFSDP--------------------------------------------------------------------------------------
AtBglu8         S-SFGMYYVNFSDP--------------------------------------------------------------------------------------
AtBglu9         T-SFGMYHVNFSDP--------------------------------------------------------------------------------------
BD1G10890       ------------ST--------------------------------------------------------------------------------------
BD1G10920       S-KFGIVYVDFNST--------------------------------------------------------------------------------------
BD1G10930       S-KFGIVYVDFNST--------------------------------------------------------------------------------------
BD1G10940       S-KFGIVYVDFTT---------------------------------------------------------------------------------------
BD1G19270       S-RFGIVYVDYKT---------------------------------------------------------------------------------------
BD1G33040       V-RFGLYYIDYNN---------------------------------------------------------------------------------------
BD1G42690       V-RFGLNYVNYKD---------------------------------------------------------------------------------------
BD1G70170       S-RFGLYFVDYND---------------------------------------------------------------------------------------
BD2G09190       T-SVGEGLSDRSRT-AEC---APRPNSVAQAQS---RGWISIQPPVLQECQDSVANRHLGRCREYPMLILRELHVQPMTRPKWVGAHPPDPPDLEANCHP
BD2G09200       ----------------------------------------------------------------------------------------------------
BD2G27770       S-RYGLHYVDFKDP--------------------------------------------------------------------------------------
BD2G37450       S-SKGVDTIDWQQ---------------------------------------------------------------------------------------
BD2G57640       K-RFGLVYVDYKD---------------------------------------------------------------------------------------
BD2G59650       S-GYGLYRVEFGDK--------------------------------------------------------------------------------------
BD2G59660       S-RYGLYRVDFDNE--------------------------------------------------------------------------------------
BD3G00650       S-RFGLYYVDYRD---------------------------------------------------------------------------------------
BD3G40000       E-REGVLHVDVH----------------------------------------------------------------------------------------
BD3G40010       E-REGLLHVDVP----------------------------------------------------------------------------------------
BD3G45610       S-KYGLYRVDFDDM--------------------------------------------------------------------------------------
BD3G45630       S-KYGLYHIDFDDM--------------------------------------------------------------------------------------
BD3G45640       S-KYGLYRVDFDDV--------------------------------------------------------------------------------------
BD3G45650       S-KYGLYRVDFDDV--------------------------------------------------------------------------------------
BD4G08040       S-KFGLVYVDRKT---------------------------------------------------------------------------------------
BD4G09920       P-KFGLVAVDRAN---------------------------------------------------------------------------------------
BD4G34930       P-RAGTPTTTSSGRSTCRTTSKPRL-------NPSGTGRTC---------------------------------------------------RATSCGR-
BD4G34940       L-RFGLCGVDMSDP--------------------------------------------------------------------------------------
BD4G34950       S-RFGLCGVDMNAV--------------------------------------------------------------------------------------
BD5G13260       V-RFGLNFVDYCD---------------------------------------------------------------------------------------
BD5G13270       M-RFGLHFVDYDD---------------------------------------------------------------------------------------
BD5G15530       Q-RYGLYHVDFKT---------------------------------------------------------------------------------------
BD5G15540       V-RFGLYHVDFET---------------------------------------------------------------------------------------
GRMZM2G012236   I-KYGLFHVNFKS---------------------------------------------------------------------------------------
GRMZM2G015804   S-RFGLYFVDYKD---------------------------------------------------------------------------------------
GRMZM2G031660   L-RFGLHYVDYQT---------------------------------------------------------------------------------------
GRMZM2G055699   S-RYGLYHVDFQDP--------------------------------------------------------------------------------------
GRMZM2G069024   S-QYGLYRVDFEDE--------------------------------------------------------------------------------------
GRMZM2G108133   L-RFGVYGVEFNST--------------------------------------------------------------------------------------
GRMZM2G110948   S-RFGLVYVDFRT---------------------------------------------------------------------------------------
GRMZM2G112704   S-KFGIVYVDRNT---------------------------------------------------------------------------------------
GRMZM2G128197   S-KFGIVYVDFAT---------------------------------------------------------------------------------------
GRMZM2G148176   S-RFGLVYVDYKT---------------------------------------------------------------------------------------
GRMZM2G163544   V-RFGLYYIDYNN---------------------------------------------------------------------------------------
GRMZM2G177661   S-RFGLYFVDYKD---------------------------------------------------------------------------------------
GRMZM2G376416   F-RFGLYGVDFAAD--------------------------------------------------------------------------------------
```

```
GRMZM2G457040    L-KYGLYHVDFDT---------------------------------------------------------------------------------------
Os10bglu34       S-RFGLYYVDYKN---------------------------------------------------------------------------------------
Os11bglu36       P-KFGLVAVDRAN---------------------------------------------------------------------------------------
Os12bglu38       S-KFGIVYVDRST---------------------------------------------------------------------------------------
Os1bglu1         S-RFGLVYVDFRT---------------------------------------------------------------------------------------
Os1bglu2         S-RSGLYRVDFDDG--------------------------------------------------------------------------------------
Os1bglu3         T-RYGLYRVDFDDA--------------------------------------------------------------------------------------
Os1bglu4         K-RFGIVYVDYKN---------------------------------------------------------------------------------------
Os1bglu5         Q-SYGLYRVDFADE--------------------------------------------------------------------------------------
Os3bglu6         S-RFGLYFVDYKD---------------------------------------------------------------------------------------
Os3bglu7         S-KFGIVYVDFNT---------------------------------------------------------------------------------------
Os3bglu8         S-KFGIVYVDFTT---------------------------------------------------------------------------------------
Os4bglu10        V-RFGINFVDYDN---------------------------------------------------------------------------------------
Os4bglu11        V-RFGINFVDYDD---------------------------------------------------------------------------------------
Os4bglu12        V-RFGINFVDYND---------------------------------------------------------------------------------------
Os4bglu13        V-RFGINFVDYND---------------------------------------------------------------------------------------
Os4bglu14        L-RFGLYHVHYKT---------------------------------------------------------------------------------------
Os4bglu16        V-KFGLYQVDFDT---------------------------------------------------------------------------------------
Os4bglu18        L-RFGLYYIDYRT---------------------------------------------------------------------------------------
Os4bglu27        G-RFGLIYVDRE----------------------------------------------------------------------------------------
Os4bglu28        D-RFGLIYVDRA----------------------------------------------------------------------------------------
Os4bglu9         -----------ND---------------------------------------------------------------------------------------
Os5bglu19        A-HFGIVAVDFGSE--------------------------------------------------------------------------------------
Os5bglu21        TWHFGLVAVDFDSE--------------------------------------------------------------------------------------
Os5bglu22        S-PFGLHYVDFEDP--------------------------------------------------------------------------------------
Os5bglu23        S-PFGLHHVDFEDP--------------------------------------------------------------------------------------
Os5gblu20        SWHYGLVAVDFGST--------------------------------------------------------------------------------------
Os6bglu24        V-RFGINYIDYKD---------------------------------------------------------------------------------------
Os6bglu25        V-RFGLYYIDYKN---------------------------------------------------------------------------------------
Os7bglu26        S-RFGIVYVDYKT---------------------------------------------------------------------------------------
Os9bglu29        D-RFGLIYIDRLN---------------------------------------------------------------------------------------
Os9bglu30        D-RFGLIYVDRK----------------------------------------------------------------------------------------
Os9bglu31        L-RFGLYGVDFASP--------------------------------------------------------------------------------------
Os9bglu32        V-RQELGTVVLRLP--------------------------------------------------------------------------------------
Os9bglu33        L-RFGLCGVDFTAA--------------------------------------------------------------------------------------
SB01G010825      S-KFGIVYVDFTT---------------------------------------------------------------------------------------
SB01G010830      S-KFGIVYVDFSTP--------------------------------------------------------------------------------------
SB01G013360      S-RFGLYFVDYNN---------------------------------------------------------------------------------------
SB01G043030      S-RFGLYFVDYKD---------------------------------------------------------------------------------------
SB02G028400      D-RFGLIFIDRLN---------------------------------------------------------------------------------------
SB02G029620      L-RFGLYGVDFSSS--------------------------------------------------------------------------------------
SB02G041550      S-RFGLVYVDYKT---------------------------------------------------------------------------------------
SB03G037780      S-QYGLYRVDFEDE--------------------------------------------------------------------------------------
SB03G042690      K-RFGIVYVDYKN---------------------------------------------------------------------------------------
SB06G019830      V-RFGINFVDYND---------------------------------------------------------------------------------------
SB06G019840      V-RFGIYFVDYSD---------------------------------------------------------------------------------------
SB06G019850      V-RFGIYFVDYND---------------------------------------------------------------------------------------
SB06G019860      V-RFGINFVEYND---------------------------------------------------------------------------------------
```

```
SB06G019880    V-RFGLNYVDYND---------------------------------------------------------------------------------------
SB06G022410    I-KYGLYHVDFKS---------------------------------------------------------------------------------------
SB06G022420    T-KYGLYYVDFKS---------------------------------------------------------------------------------------
SB06G022450    Q-RFGLYHVDFKT---------------------------------------------------------------------------------------
SB06G022460    Q-RFGLYYVDYNT---------------------------------------------------------------------------------------
SB06G022490    V-RFGLYHVDYDT---------------------------------------------------------------------------------------
SB06G022500    L-RFGLHYVDYQT---------------------------------------------------------------------------------------
SB06G022510    L-RFGLHYVDYQT---------------------------------------------------------------------------------------
SB08G007586    E-RYGIVYLDREN---------------------------------------------------------------------------------------
SB08G007650    E-RFGIVYVDRDN---------------------------------------------------------------------------------------
SB09G018160    A-HFGLISVDFNDL--------------------------------------------------------------------------------------
SB10G012220    ----------------------------------------------------------------------------------------------------
SB10G022300    F-RFGLYGVDFAAD--------------------------------------------------------------------------------------
SB10G027600    V-RFGLYYIDYNN---------------------------------------------------------------------------------------
SB10G028060    S-RFGIVHVDRST---------------------------------------------------------------------------------------
SbDhr1         E-RFGIVYVDREN---------------------------------------------------------------------------------------
SbDhr2         E-RFGIVYVDREN---------------------------------------------------------------------------------------
Zm-p60.1       E-RYGIVYVDRNN---------------------------------------------------------------------------------------
ZmGLU2         E-RYGIVYVDRKN---------------------------------------------------------------------------------------
ZmGLU3         E-RYGIIYVDRDD---------------------------------------------------------------------------------------
ZmGLU4         Q-PYGIVYVDRND---------------------------------------------------------------------------------------
ZmGLU5         E-RYGIVYVDRND---------------------------------------------------------------------------------------
ZmGLU6         E-RYGIVYVDRND---------------------------------------------------------------------------------------
ZmGLU7         E-RYGIVYVDRGN---------------------------------------------------------------------------------------
ZmGLU8         E-RYGIVYVDRSN---------------------------------------------------------------------------------------
```

```
                      2310      2320      2330      2340      2350      2360      2370      2380      2390      2400
                ....|....|....|....|....|....|....|....|....|....|....|....|....|....|....|....|....|....|....|....|
AC155376.2_FGT005 ----------------------------------------GLSRHP-------KA-SALWFS---------------------RL---------------
AC211140.2_FGT007 ----------------------------------------NLKRHP-------KN-SVQWFK---------------------TL---------------
AC217401.3_FGT001 ----------------------------------------KLERHP-------KA-SAYWFR---------------------DM---------------
AC217687.3_FGT004 -----------------------------------------LERHP-------KA-SAYWFR---------------------DM---------------
AtBglu1           ----------------------------------------HRKRSP-------KL-SAHWYS---------------------GF---------------
AtBglu10          ----------------------------------------GRKRTP-------KL-SASWYT---------------------GF---------------
AtBglu11          ----------------------------------------SLKRSP-------KL-SAHWYS---------------------SF---------------
AtBglu12          ----------------------------------------GRKRYP-------KK-SAKWFK---------------------KL---------------
AtBglu13          ----------------------------------------GRKRYP-------KK-SAKWFR---------------------KL---------------
AtBglu14          ----------------------------------------RRKRYL-------KK-SAHWFR---------------------HL---------------
AtBglu15          ----------------------------------------GCKRYP-------KK-SAEWFR---------------------KL---------------
AtBglu16          ----------------------------------------GRKRYL-------KK-SAKWFR---------------------RL---------------
AtBglu17          ----------------------------------------GLKRHL-------KS-SALWYH---------------------HF---------------
AtBglu18          ----------------------------------------NLTRHQ-------KV-SGKWYS---------------------EF---------------
AtBglu19          ----------------------------------------NLTRME-------KE-SAKWLS---------------------EF---------------
AtBglu2           ----------------------------------------------------------------------------------------------------
AtBglu20          ----------------------------------------NLTRHE-------KL-SAQWYS---------------------SF---------------
AtBglu21          ----------------------------------------NLTRYE-------KE-SAKYYK---------------------DF---------------
AtBglu22          ----------------------------------------NLTRYE-------KE-SAKYYK---------------------DF---------------
AtBglu23          ----------------------------------------NLTRYE-------KE-SGKYYK---------------------DF---------------
AtBglu24          ----------------------------------------NLTRHE-------KV-SGKYYR---------------------EF---------------
AtBglu25          ----------------------------------------NMGRHE-------KQ-SAKWLS---------------------KL---------------
AtBglu26          ----------------------------------------GLRRYP-------KM-SALWLK---------------------EF---------------
AtBglu27          ----------------------------------------GLKRFP-------KM-SAMWFK---------------------EF---------------
AtBglu28          ----------------------------------------DLTRIP-------KD-SVNWFK---------------------QF---------------
AtBglu29          ----------------------------------------NLQRYP-------KD-SVNWFK---------------------KF---------------
AtBglu3           ----------------------------------------HRTRSP-------KL-SAHWYS---------------------AF---------------
AtBglu30          ----------------------------------------GLKRYP-------KD-SVKWFK---------------------RF---------------
AtBglu31          ----------------------------------------GLQRHA-------KH-SAMWFK---------------------HF---------------
AtBglu32          ----------------------------------------GLSRHA-------KN-SAKWFK---------------------HF---------------
AtBglu33          ----------------------------------------NMKRYI-------RS-SGKWLS---------------------EF---------------
AtBglu34          ----------------------------------------PADRKE-------KA-SGKWFS---------------------KF---------------
AtBglu35          ----------------------------------------PADRKE-------KA-SGKWFS---------------------KF---------------
AtBglu36          ----------------------------------------PADRRE-------KA-SGKWFS---------------------RF---------------
AtBglu37          ----------------------------------------TADRDL-------KA-SGLWYQ---------------------SF---------------
AtBglu38          ----------------------------------------TGDRDL-------KA-SGKWFQ---------------------KF---------------
AtBglu39          -----------------------------------------KGAFP
AtBglu4           ----------------------------------------HRKRSP-------KL-SAYWYS---------------------DF---------------
AtBglu40          ----------------------------------------NLKRYP-------KD-SVHWFT---------------------SF---------------
AtBglu41          ----------------------------------------NLTRIP-------KA-SARWFQ---------------------TI---------------
AtBglu42          ----------------------------------------GLTRHP-------KS-SAYWFM---------------------KF---------------
AtBglu43          -----------------------------------------LKRYP-------KM-SALWFK---------------------QL---------------
AtBglu44          -----------------------------------------LKRYP-------KM-SAQWFK---------------------QL---------------
AtBglu45          -----------------------------------------LKRSP-------KQ-SASWYK---------------------NY---------------
AtBglu46          -----------------------------------------LKRTP-------KQ-SATWYK---------------------NF---------------
AtBglu47          -----------------------------------------QERTP-------RL-SASWYK---------------------NF---------------
```

```
AtBglu5          ----------------------------------------HRKRSP-------RL-SAHWYS---------------------DF---------------
AtBglu7          ----------------------------------------GRKRSP-------KL-SASWYT---------------------GF---------------
AtBglu8          ----------------------------------------GRKRSP-------KL-SASWYT---------------------GF---------------
AtBglu9          ----------------------------------------GRKRTP-------KL-SASWYT---------------------GF---------------
BD1G10890        ----------------------------------------ELERHP-------KA-LAYWFR---------------------DM---------------
BD1G10920        ----------------------------------------KLERHP-------KA-SAYWFR---------------------DM---------------
BD1G10930        ----------------------------------------KLERHP-------KA-SAYWFR---------------------DM---------------
BD1G10940        -----------------------------------------LKRYP-------KD-SAYWFK---------------------DM---------------
BD1G19270        -----------------------------------------LKRYP-------KD-SAFWFK---------------------NM---------------
BD1G33040        ----------------------------------------NLTRIP-------KA-SVKWFS---------------------QV---------------
BD1G42690        ----------------------------------------GLKRYP-------KR-SSQWFQ---------------------KF---------------
BD1G70170        ----------------------------------------NLKRYP-------KN-SVQWFK---------------------SL---------------
BD2G09190        SEVWDA--QTSKFGWEAHPTAANVAMAASR----------SDQRNP-------W----RTFS---------------------TC---------------
BD2G09200        ----------------------------------------------------------------------------------------------------
BD2G27770        ----------------------------------------GLPRLP-------KL-SAKWYS---------------------KF---------------
BD2G37450        ----------------------------------------VAGDFLNQKGFSNSL-PVKIFK---------------------EH---------------
BD2G57640        ----------------------------------------GLSRHP-------KA-SAMWFS---------------------RF---------------
BD2G59650        ----------------------------------------ALPRQA-------RL-SARWYS---------------------DF---------------
BD2G59660        ----------------------------------------ALPRQA-------RL-SACWYS---------------------GF---------------
BD3G00650        ----------------------------------------NLKRYP-------KN-SVQWFK---------------------DL---------------
BD3G40000        ----------------------------------------GLLRVR------GRVQGPVWAH--LRGPGHARQVPQEVQLLAR-GIPQETPLIDELLAGH
BD3G40010        ----------------------------------------RLLRVR-------GR-VQGPLRPHLRRPRHAQALPQEVQLLARRFPQEMTRIL---LE-P
BD3G45610        ----------------------------------------RRPRQA-------RL-SARWYS---------------------GF---------------
BD3G45630        ----------------------------------------RRPRQA-------RL-SARWYS---------------------GF---------------
BD3G45640        ----------------------------------------RRPRQP-------RL-SARWYS---------------------VF---------------
BD3G45650        ----------------------------------------RRPRQA-------RL-SARWYS---------------------GF---------------
BD4G08040        -----------------------------------------FTRYP-------KD-STRWFR---------------------KM---------------
BD4G09920        ----------------------------------------NLARKP-------RP-SYYLFS---------------------KI---------------
BD4G34930        --SWTCSSTSSATVWASASTASTSAPRRGRGTRGTPPSGSPASSAA-------AS-SGRWRCPARPTPSDS------------HL---------------
BD4G34940        ----------------------------------------GRTRYV-------RN-SARWYS---------------------GF---------------
BD4G34950        ----------------------------------------ERTRYM-------RN-SARWYS---------------------SF---------------
BD5G13260        ----------------------------------------GQKRYP-------KN-SARWFR---------------------NF---------------
BD5G13270        ----------------------------------------ERKRYP-------KH-SARWFK---------------------KF---------------
BD5G15530        -----------------------------------------QKRTP-------KL-SAGWYR---------------------KF---------------
BD5G15540        -----------------------------------------QKRIP-------KT-SAKWYR---------------------GF---------------
GRMZM2G012236    -----------------------------------------LKRTP-------KL-SAKWYN---------------------KF---------------
GRMZM2G015804    ----------------------------------------NLKRHP-------KN-SVQWFK---------------------TL---------------
GRMZM2G031660    -----------------------------------------QERKP-------KS-SALWYK---------------------RF---------------
GRMZM2G055699    ----------------------------------------ELPRTP-------KL-SALWYS---------------------KF---------------
GRMZM2G069024    ----------------------------------------ALPRQA-------RL-SARWYS---------------------KF---------------
GRMZM2G108133    ----------------------------------------ARTRYQ-------RH-SAKWYA---------------------SF---------------
GRMZM2G110948    -----------------------------------------LRRYP-------KS-SAYWFR---------------------DV---------------
GRMZM2G112704    -----------------------------------------FVRYP-------KD-SARWFR---------------------KV---------------
GRMZM2G128197    -----------------------------------------LKRYP-------KD-SAYWFR---------------------DM---------------
GRMZM2G148176    -----------------------------------------LKRYP-------KD-SAFWFK---------------------HM---------------
GRMZM2G163544    ----------------------------------------NLTRIP-------KA-SVEWFR---------------------QV---------------
GRMZM2G177661    ----------------------------------------NLKRYP-------KS-SVQWFK---------------------NL---------------
GRMZM2G376416    ----------------------------------------NRTRYA-------RR-SARWYA---------------------GF---------------
```

```
GRMZM2G457040    -----------------------------------------QERTP-------RM-SARWYQ---------------------GF---------------
Os10bglu34       -----------------------------------------RKRYP-------KN-SVQWFK---------------------NL---------------
Os11bglu36       ----------------------------------------NLARKP-------RP-SYFLFS---------------------RV---------------
Os12bglu38       -----------------------------------------FTRYP-------KD-STRWFR---------------------KM---------------
Os1bglu1         -----------------------------------------LRRYP-------KM-SAYWFR---------------------DL---------------
Os1bglu2         ----------------------------------------ARPRRA-------RR-SARWYS---------------------DF---------------
Os1bglu3         ----------------------------------------ALPRRA-------KR-SARWYR---------------------DF---------------
Os1bglu4         ----------------------------------------GLSRHP-------KA-SARWFS---------------------RF---------------
Os1bglu5         ----------------------------------------SRPRQA-------RL-SARWYS---------------------GF---------------
Os3bglu6         ----------------------------------------NLKRYP-------KN-SVQWFK---------------------AL---------------
Os3bglu7         -----------------------------------------LERHP-------KA-SAYWFR---------------------DM---------------
Os3bglu8         -----------------------------------------LKRYP-------KD-SANWFK---------------------NM---------------
Os4bglu10        ----------------------------------------GMKRYP-------KN-SARWFK---------------------KF---------------
Os4bglu11        ----------------------------------------GMKRYP-------KN-SARWFK---------------------KF---------------
Os4bglu12        ----------------------------------------GRKRYP-------KN-SAHWFK---------------------KF---------------
Os4bglu13        ----------------------------------------GAKRYP-------KM-SAHWFK---------------------EF---------------
Os4bglu14        -----------------------------------------LKRTP-------KL-SVDWYR---------------------KF---------------
Os4bglu16        -----------------------------------------QERIP-------RM-SAKWYR---------------------DF---------------
Os4bglu18        -----------------------------------------QERSP-------KL-SALWYK---------------------EF---------------
Os4bglu27        ----------------------------------------TLKRYR-------KK-SSYWFA---------------------DF---------------
Os4bglu28        ----------------------------------------TLARFR-------KK-SSYWFA---------------------DF---------------
Os4bglu9         ----------------------------------------LHRRSA-------KL-SFIIQN---------------------YL---------------
Os5bglu19        ----------------------------------------ELTRQP-------RR-SARWYS---------------------DF---------------
Os5bglu21        ----------------------------------------KRRRQP-------RR-SASWYS---------------------EF---------------
Os5bglu22        ----------------------------------------NLPRQP-------KL-SAHWYS---------------------KF---------------
Os5bglu23        ----------------------------------------SLPRQP-------KL-SAQWYS---------------------KF---------------
Os5gblu20        ----------------------------------------ERRRQP-------RR-SASWYS---------------------DF---------------
Os6bglu24        ----------------------------------------GLKRYP-------KR-SSQWLQ---------------------NF---------------
Os6bglu25        ----------------------------------------NLTRIP-------KA-SVQWFS---------------------QV---------------
Os7bglu26        -----------------------------------------LKRYP-------KD-SAFWFK---------------------NM---------------
Os9bglu29        ----------------------------------------NLKRYH-------KQ-SSYWIA---------------------NF---------------
Os9bglu30        ----------------------------------------TLKRYR-------KE-SSYWIE---------------------DF---------------
Os9bglu31        ----------------------------------------ERTRYQ-------RH-SARWYA---------------------GF---------------
Os9bglu32        ----------------------------------------QRRRAP-------A---GETLR---------------------RI---------------
Os9bglu33        ----------------------------------------ARTRYL-------KN-SARWYS---------------------GF---------------
SB01G010825      -----------------------------------------LKRYP-------KD-SAYWFR---------------------DM---------------
SB01G010830      ----------------------------------------KLERHP-------KD-SAYWFR---------------------DM---------------
SB01G013360      ----------------------------------------NLKRYP-------KN-SVLWFK---------------------NL---------------
SB01G043030      ----------------------------------------NLKRYP-------KN-SVQWFK---------------------TL---------------
SB02G028400      ----------------------------------------GLKRYR-------KE-SSYWIQ---------------------NF---------------
SB02G029620      ----------------------------------------ARTRYQ-------RH-SARWYS---------------------SF---------------
SB02G041550      -----------------------------------------LKRYP-------KD-SAFWFK---------------------HM---------------
SB03G037780      ----------------------------------------ALPRQA-------RL-SARWYS---------------------KF---------------
SB03G042690      ----------------------------------------GLSRHP-------KA-SALWFS---------------------RF---------------
SB06G019830      ----------------------------------------GLKRYP-------KN-SAHWFR---------------------EI---------------
SB06G019840      ----------------------------------------GLKRYP-------KS-SAHWFK---------------------KF---------------
SB06G019850      ----------------------------------------GLKRYP-------KS-SAHWFT---------------------EF---------------
SB06G019860      ----------------------------------------GLKRYP-------KS-SAHWFT---------------------EF---------------
```

```
SB06G019880   ----------------------------------------GLKRYP-------KN-SAHWFK---------------------AF---------------
SB06G022410   -----------------------------------------LKRTP-------KL-SAKWYS---------------------NF---------------
SB06G022420   -----------------------------------------LKRTP-------KL-SAKWYS---------------------KF---------------
SB06G022450   -----------------------------------------QKRTP-------KL-SAKWYS---------------------EF---------------
SB06G022460   -----------------------------------------QKRTP-------KL-STKWYR---------------------EF---------------
SB06G022490   -----------------------------------------QERTP-------RM-SATWYQ---------------------GF---------------
SB06G022500   -----------------------------------------QERKP-------KS-SALWYK---------------------RF---------------
SB06G022510   -----------------------------------------QERKP-------KS-SALWYK---------------------RF---------------
SB08G007586   ----------------------------------------GCERTM-------KR-SARWFQ---------------------EF---------------
SB08G007650   ----------------------------------------GCERTM-------KR-SAWWLQ---------------------EF---------------
SB09G018160   ----------------------------------------RRQRQP-------RL-SAYWYS---------------------DF---------------
SB10G012220   ----------------------------------------------------------------------------------------------------
SB10G022300   ----------------------------------------DRTRYA-------RS-SARWYA---------------------GF---------------
SB10G027600   ----------------------------------------NLTRIP-------KA-SVEWFK---------------------QV---------------
SB10G028060   -----------------------------------------FVRYP-------KD-SARWFR---------------------KV---------------
SbDhr1        ----------------------------------------GCERTM-------KR-SARWLQ---------------------EF---------------
SbDhr2        ----------------------------------------GCKRTL-------KR-SARWLK---------------------EF---------------
Zm-p60.1      ----------------------------------------NCTRYM-------KE-SAKWLK---------------------QF---------------
ZmGLU2        ----------------------------------------NYTRYM-------KE-SAKWLK---------------------EF---------------
ZmGLU3        ----------------------------------------GYRRYL-------KR-SAKWLR---------------------EF---------------
ZmGLU4        ----------------------------------------GCKRYM-------KQ-SAKWYK---------------------SF---------------
ZmGLU5        ----------------------------------------GCKRYM-------KR-SAKWFK---------------------EF---------------
ZmGLU6        ----------------------------------------GYKRYM-------KK-SAKWLK---------------------EF---------------
ZmGLU7        ----------------------------------------GCKRRM-------KR-SAKWLK---------------------KF---------------
ZmGLU8        ----------------------------------------GCKRRM-------KR-SAKWLK---------------------KF---------------
```

```
                          2410      2420      2430      2440      2450      2460      2470      2480      2490      2500
                   ....|....|....|....|....|....|....|....|....|....|....|....|....|....|....|....|....|....|....|....|
AC155376.2_FGT005  --------LKGE-AA-------------------------------------------------------------------------------------
AC211140.2_FGT007  --------LS------------------------------------------------------------------------------------------
AC217401.3_FGT001  --------LQK-----------------------------------------------------------------------------------------
AC217687.3_FGT004  --------LQK-----------------------------------------------------------------------------------------
AtBglu1            --------LKGK-PTFLGS--QG-----------------I-------T---------------------------------------------------
AtBglu10           --------LNGT-IDVATQ--D------------------T-------I---------------------------------------------------
AtBglu11           --------LKGT-LHH------------------------------------------------------------------------------------
AtBglu12           --------LNEK----------------------------------------------------------------------------------------
AtBglu13           --------LSEK----------------------------------------------------------------------------------------
AtBglu14           --------LNGK----------------------------------------------------------------------------------------
AtBglu15           --------LNEK----------------------------------------------------------------------------------------
AtBglu16           --------LKGA-HGGTNE---------------------------------------------------------------------------------
AtBglu17           --------LSNS-S--------------------------------------------------------------------------------------
AtBglu18           --------LKPQ-FPT------------------------------------------------------------------------------------
AtBglu19           --------LKPG-LKPSK----------------------------------------------------------------------------------
AtBglu2            ----------------------------------------------------------------------------------------------------
AtBglu20           --------LHDG-SKEFEI--E------------------------------------------------------------------------------
AtBglu21           --------LAQG-VRPS-----------------------------------------------------------------------------------
AtBglu22           --------LGQG-VRPS-----------------------------------------------------------------------------------
AtBglu23           --------LSQG-VRPS-----------------------------------------------------------------------------------
AtBglu24           --------LSEG-VRPS-----------------------------------------------------------------------------------
AtBglu25           --------LEKV-PDTIQS--KV-----------------------------------------------------------------------------
AtBglu26           --------LRFD-QEDDSS--TS-------------------------KK-EE--KKES---YGKQLL--HSV-------------------QDSQFVHS
AtBglu27           --------LKRE-EEIEDS--EE-------------------------EE-YV--LKSTM--NKKRFL--LA----------------------------
AtBglu28           --------LDVK-NKEIWD--VS-------------------------HK-ER---------YNKTFDDVESFEASVGSILYLMTNNISRRE---EEERD
AtBglu29           --------LSRP-VVRSEE--TE-------------------------DE-KVCNVSRKEEKINKALDVSEGFKTSVDSIVNLIKNGSRIEEEDDEEERD
AtBglu3            --------LKGN-TTFLGS--QG-----------------I-------M---------------------------------------------------
AtBglu30           --------LKKS-VVGESN--KE-------------------------EV-EE--MSRAEG--NKTF---KGFEESAGFFASFMAMNQSRRD---E-ENN
AtBglu31           --------LERS-GKPMPM--DL-------------------------FK-S------------------------------------------------
AtBglu32           --------LQRS-GKPMPL--DL-------------------------FK-S------------------------------------------------
AtBglu33           --------LDSK-ETLHKC--YFEGHREKGYAPKLFDVEYL-------EP-E------------------------------------------------
AtBglu34           --------LA------------------------------------------------------------------------------------------
AtBglu35           --------LA------------------------------------------------------------------------------------------
AtBglu36           --------IA------------------------------------------------------------------------------------------
AtBglu37           --------LRDT-TKNQD---------------------IL-------RS-SL-----------------------------------------------
AtBglu38           --------INVT-DEDSTN--QD----------------LL-------RS-SV-----------------------------------------------
AtBglu39           ----------------------------------------------------------------------------------------------------
AtBglu4            --------LKGE-SAFLDS--QG-----------------I-------K---------------------------------------------------
AtBglu40           --------LNS-----------------------------------------------------------------------------------------
AtBglu41           --------LSGS-SSTSDS--SK-----------------------------------------------------------------------------
AtBglu42           --------LKGD-EE-------------------------------------------------------------------------------------
AtBglu43           --------LKR-----------------------------------------------------------------------------------------
AtBglu44           --------LKR-----------------------------------------------------------------------------------------
AtBglu45           --------IEEH-VNRR-----------------------------------------------------------------------------------
AtBglu46           --------IEQN-VNIE-----------------------------------------------------------------------------------
AtBglu47           --------IFQH-RALSKD--DW-------------------------CL-K------------------------------------------------
```

```
AtBglu5         --------LKGK-TSFLDS--KG-----------------I-------K---------------------------------------------------
AtBglu7         --------LNGT-IDVASQ--D------------------T-------I---------------------------------------------------
AtBglu8         --------LNGT-IDVASQ--D------------------M-------T---------------------------------------------------
AtBglu9         --------LNGT-IDVASQ--D------------------T-------I---------------------------------------------------
BD1G10890       --------LK------------------------------------------------------------------------------------------
BD1G10920       --------LK------------------------------------------------------------------------------------------
BD1G10930       --------LK------------------------------------------------------------------------------------------
BD1G10940       --------LHAS-GTGTTK--DG-------------------------TDLS-----------------------------NSTQAGTGVSSKNSAAGSA
BD1G19270       --------LSK-----------------------------------------------------------------------------------------
BD1G33040       --------LAQK-T--------------------------------------------------------------------------------------
BD1G42690       --------LH------------------------------------------------------------------------------------------
BD1G70170       --------LS------------------------------------------------------------------------------------------
BD2G09190       --------FS------------------------------------------------------------------------------------------
BD2G09200       ----------------------------------------------------------------------------------------------------
BD2G27770       --------LRSE-IGINIE--DK-----------------V-------S---------------------------------------------------
BD2G37450       -------------E--------------------------------------------------------------------------------------
BD2G57640       --------LNGE-AAD------------------------------------------------------------------------------------
BD2G59650       --------LKKK-KDSGSVHVQN-------------------------E---------------------------------------------------
BD2G59660       --------LRKN-NGTHV---QS-------------------------V---------------------------------------------------
BD3G00650       --------LA------------------------------------------------------------------------------------------
BD3G40000       DGPHSPRTAW------------------------------------------------------------------------------------------
BD3G40010       RTVYTYICMY------------------------------------------------------------------------------------------
BD3G45610       --------LKKN-GSSLLI--SR-------------------------T---------------------------------------------------
BD3G45630       --------LKKN-GSSLLV--SR-------------------------T---------------------------------------------------
BD3G45640       --------LKKN-GSSPLV--SG-------------------------T---------------------------------------------------
BD3G45650       --------LNKN-GSSLLL--LT-----------------------------------------------------------------------------
BD4G08040       --------IKN-----------------------------------------------------------------------------------------
BD4G09920       --------VATG-KVTRQD--R------------------M-------SAWRELQQAAIQKKTRPFYRDVDKHGRMYAGGLDRPVQRNFVLRDWRFGHYE
BD4G34930       --------LRNG-WRL------------------------------------------------------------------------------------
BD4G34940       --------LHGG-ELRPVA--Q------------------------------------------------------------------------------
BD4G34950       --------LKGG-ELRPGS--P------------------------------------------------------------------------------
BD5G13260       --------LK------------------------------------------------------------------------------------------
BD5G13270       --------LK------------------------------------------------------------------------------------------
BD5G15530       --------LKGS-LLTRKS--RN-------------------------G---------------------------------------------------
BD5G15540       --------LAGA-GPVD-----D-----------------------------------------------------------------------------
GRMZM2G012236   --------IKGY-EQIEIA--SE-----------------------------------------------------------------------------
GRMZM2G015804   --------LS------------------------------------------------------------------------------------------
GRMZM2G031660   --------LQSS----------------------------------------------------------------------------------------
GRMZM2G055699   --------LKNE-IDIIEN--VV-----------------S-------D---------------------------------------------------
GRMZM2G069024   --------LENK-GIH-V---ED-------------------------E---------------------------------------------------
GRMZM2G108133   --------LRGG-ELRPVA--L------------------------------------------------------------------------------
GRMZM2G110948   --------IAG-----------------------------------------------------------------------------------------
GRMZM2G112704   --------IKN-----------------------------------------------------------------------------------------
GRMZM2G128197   --------LSGT-GSKAAT--P--------------------------QTGS-----------------------------GGRPAGSA---------SA
GRMZM2G148176   --------LSK-----------------------------------------------------------------------------------------
GRMZM2G163544   --------LAQK-TAGLEY---------------------------------------------------------------------------------
GRMZM2G177661   --------LA------------------------------------------------------------------------------------------
GRMZM2G376416   --------LRGG-AATSRP--PL-----------------FQRGHTAPP---------------------------------------------------
```

```
GRMZM2G457040    --------LTAR-TSQR-----D-------------------------E---------------------------------------------------
Os10bglu34       --------LAS-----------------------------------------------------------------------------------------
Os11bglu36       --------VTTG-KITRQD--R------------------M-------SAWRELQQAAVQKKTRPFFRAVDKHGRMYAGGLDRPIQRPFILRDWRFGHYK
Os12bglu38       --------IKS-----------------------------------------------------------------------------------------
Os1bglu1         --------VSS-----------------------------------------------------------------------------------------
Os1bglu2         --------LKGK-KDP------------------------------------------------------------------------------------
Os1bglu3         --------LKSK-RQP------------------------------------------------------------------------------------
Os1bglu4         --------LKGD-DAE------------------------------------------------------------------------------------
Os1bglu5         --------LKNR-EMDVDQ--SE-----------------L-------A---------------------------------------------------
Os3bglu6         --------LK------------------------------------------------------------------------------------------
Os3bglu7         --------LK------------------------------------------------------------------------------------------
Os3bglu8         --------LQAS-GPGSKS--G--------------------------SGTS-----------------------------DS-QVGSATSASH-PVGSA
Os4bglu10        --------LR------------------------------------------------------------------------------------------
Os4bglu11        --------LQKS-NRDGNK---------------------------------------------------------------------------------
Os4bglu12        --------LL------------------------------------------------------------------------------------------
Os4bglu13        --------LQ------------------------------------------------------------------------------------------
Os4bglu14        --------LTGS-LLRRKF--RD-------------------------E---------------------------------------------------
Os4bglu16        --------LTSS-SLTD-----G-------------------------L---------------------------------------------------
Os4bglu18        --------LQNL----------------------------------------------------------------------------------------
Os4bglu27        --------LK------------------------------------------------------------------------------------------
Os4bglu28        --------LR------------------------------------------------------------------------------------------
Os4bglu9         --------LLGIHFQPG-----------------------------------------------------------------------------------
Os5bglu19        --------LKNN-AVIKVD--DG-------------------------S---------------------------------------------------
Os5bglu21        --------LKNN-SVIRVE--ED-------------------------G---------------------------------------------------
Os5bglu22        --------LRGE-IGINIE--ST-----------------I-------S---------------------------------------------------
Os5bglu23        --------LRSE-IGIN-----------------------------------------------------------------------------------
Os5gblu20        --------LKNN-APIRVE--DG-------------------------S---------------------------------------------------
Os6bglu24        --------LH------------------------------------------------------------------------------------------
Os6bglu25        --------LAQK-T--------------------------------------------------------------------------------------
Os7bglu26        --------LSSK----------------------------------------------------------------------------------------
Os9bglu29        --------LKR-----------------------------------------------------------------------------------------
Os9bglu30        --------LKR-----------------------------------------------------------------------------------------
Os9bglu31        --------LRGG-ELRPAA--A------------------------------------------------------------------------------
Os9bglu32        --------MN------------------------------------------------------------------------------------------
Os9bglu33        --------LRGG-ELR------------------------------------------------------------------------------------
SB01G010825      --------LSGT-GSKAAA--AP-------------------------QTGS-----------------------------GTRPAVSA---------SA
SB01G010830      --------LQK-----------------------------------------------------------------------------------------
SB01G013360      --------LASS-CKFGGP--F------------------------------------------------------------------------------
SB01G043030      --------LS------------------------------------------------------------------------------------------
SB02G028400      --------LK------------------------------------------------------------------------------------------
SB02G029620      --------LHGG-ELRPVA---------------------------------------------------------------------------------
SB02G041550      --------LSK-----------------------------------------------------------------------------------------
SB03G037780      --------LKKG-IR--V---ED-------------------------E---------------------------------------------------
SB03G042690      --------LKGE-AAE------------------------------------------------------------------------------------
SB06G019830      --------LQKN----------------------------------------------------------------------------------------
SB06G019840      --------LK------------------------------------------------------------------------------------------
SB06G019850      --------LK------------------------------------------------------------------------------------------
SB06G019860      --------LK------------------------------------------------------------------------------------------
```

```
SB06G019880     --------LQ------------------------------------------------------------------------------------------
SB06G022410     --------IKGY-EQIEMA--SE-----------------------------------------------------------------------------
SB06G022420     --------IKGN-EHIEMA--SD-----------------------------------------------------------------------------
SB06G022450     --------LKGS-PLRTRP--QN-----------------------------------------------------------------------------
SB06G022460     --------LMGS-TLRTSP--QN-------------------------GN-S------------------------------------------------
SB06G022490     --------LTAG-NTSLVT--HE-------------------------D---------------------------------------------------
SB06G022500     --------LQSL----------------------------------------------------------------------------------------
SB06G022510     --------LQSF----------------------------------------------------------------------------------------
SB08G007586     --------NGAA-KKVEN----------------------------------------------------------------------------------
SB08G007650     --------NGAA-MKVQN----------------------------------------------------------------------------------
SB09G018160     --------LKNN-VAIQVD--NG-------------------------E---------------------------------------------------
SB10G012220     ----------------------------------------------------------------------------------------------------
SB10G022300     --------LRRG-GGDLTP--PA-----------------P-------P---------------------------------------------------
SB10G027600     --------LAQK-TANLEY---------------------------------------------------------------------------------
SB10G028060     --------IKN-----------------------------------------------------------------------------------------
SbDhr1          --------NGAA-KKVEN----------------------------------------------------------------------------------
SbDhr2          --------NGAA-KRPGNL---------------------I-------KP-NF-----------------------------------------------
Zm-p60.1        --------NAAK-KP-SK----------------------------------------------------------------------------------
ZmGLU2          --------NTAK-KP-SK----------------------------------------------------------------------------------
ZmGLU3          --------NGAA-KKAEK----------------------------------------------------------------------------------
ZmGLU4          --------IAAN----------------------------------------------------------------------------------------
ZmGLU5          --------NAAK-KAAAK----------------------------------------------------------------------------------
ZmGLU6          --------NTEK----------------------------------------------------------------------------------------
ZmGLU7          --------NRAA-HTKKK----------------------------------------------------------------------------------
ZmGLU8          --------NRAA-HSKKK----------------------------------------------------------------------------------
```

```
                             2510      2520      2530      2540
                     ....|....|....|....|....|....|....|....|....
AC155376.2_FGT005    ---------------------------------------ENSYS
AC211140.2_FGT007    ------------------------------------------SS
AC217401.3_FGT001    ------------------------------------------HL
AC217687.3_FGT004    -------------------------------------------H
AtBglu1              ---------------------------Q----L--H-SNFSSSR
AtBglu10             ---------------------------Q----LQSN-ISGSSSL
AtBglu11             --------------------------------------PSYASS
AtBglu12             -----------------------------------------KKN
AtBglu13             -----------------------------------------KRN
AtBglu14             -----------------------------------------KEN
AtBglu15             -----------------------------------------KND
AtBglu16             ---------------------------------------QVAVI
AtBglu17             ---------------------------------------SYQMD
AtBglu18             -------------------------------------SKLREEL
AtBglu19             -----------------------------------S-SKLHEEL
AtBglu2              -------------------------------------------V
AtBglu20             -----------------------------------H-EFEHDEL
AtBglu21             -------------------------------------ALKRDEL
AtBglu22             -------------------------------------ALKKDEL
AtBglu23             -------------------------------------ALKKDEL
AtBglu24             -------------------------------------AIKKDEL
AtBglu25             -----------------------------------D-SDSRKEL
AtBglu26             IKDSGA---LPAVLGSLFVVSATV---G----T-SL-FFKGANN
AtBglu27             ---TGS---S----ASCFI--PKM---S----E-SS-KALELLF
AtBglu28             QCAFGNLNDQ----SGLLL---ES---Y----N-SF-GFLENVW
AtBglu29             FCAFKNHNDQ----LGFFL----------------K-LQNSLGF
AtBglu3              ---------------------------Q----MQSN-FSSSASS
AtBglu30             RCSFDFPHTH----FG-------V---L----Q-GI-ENPSSFY
AtBglu31             ---------------------------V----K-RW-WSTLQMI
AtBglu32             ---------------------------V----K-NW-WSAIPMI
AtBglu33             ---------------------------N----S-QL-SYRSDFM
AtBglu34             -------------------------------------------K
AtBglu35             -------------------------------------------K
AtBglu36             -------------------------------------------K
AtBglu37             ---------------------------P----F-KN-GDRKSLT
AtBglu38             ----------------------SS---K----N-RD-RKSLADA
AtBglu39             ----------------------------------------SFEA
AtBglu4              ---------------------------E----L--Q-SKYSSSF
AtBglu40             ------------------------------------------TS
AtBglu41             ------LI---LLEEATE---------------QQQ-EYKFQEK
AtBglu42             --------------------------------------NKGKKE
AtBglu43             -----------------------------------------DQK
AtBglu44             -----------------------------------------NNK
AtBglu45             --------------------------------------DIVDNY
AtBglu46             ---------------------------------------DQIDK
AtBglu47             ---------------------------------QKE-DTNFFLI
```

```
AtBglu5          ---------------------------E----L--Q-SNFSSSS
AtBglu7          ---------------------------Q----LQRK-CSGSSSL
AtBglu8          ---------------------------Q----LQRN-FSGSSSL
AtBglu9          ---------------------------Q----LWSN-FSVSSSL
BD1G10890        -------------------------------------------V
BD1G10920        -------------------------------------------V
BD1G10930        -------------------------------------------G
BD1G10940        TSSSPWVLLPLLVSLYLV---------------LPSISLFSSWN
BD1G19270        -----------------------------------------KRS
BD1G33040        -----------------------------------------AIM
BD1G42690        -------------------------------------------Q
BD1G70170        ------------------------------------------SS
BD2G09190        ---------------------------------ALE-TPPNTMS
BD2G09200        --------------------------------------VIVNGK
BD2G27770        ----------------------------------SD-ARSHAQQ
BD2G37450        --------------------------------TDKL-LEVLDAR
BD2G57640        --------------------------------------IKPDTN
BD2G59650        ---------------------------LENTGSREK-VLQVNEI
BD2G59660        ---------------------------L------GN-AGPHAEQ
BD3G00650        ------------------------------------------SS
BD3G40000        -------------------------------------------I
BD3G40010        -------------------------------------------V
BD3G45610        ---------------------------Q------ED-LTRNTVS
BD3G45630        ---------------------------H------ED-LTLNTVS
BD3G45640        ---------------------------Q------ED-LTLNTVS
BD3G45650        -----------------------------------T-QEGLMPK
BD4G08040        ------------------------------------------EE
BD4G09920        MEGLQDPLSWFVRCITRPFSHKKKIHYI----EDDT-VSYSISS
BD4G34930        ----------------------------------EM-VREDMHE
BD4G34940        ---------------------------------S---EKPYSEK
BD4G34950        ---------------------------------S---GKGYGVA
BD5G13260        -------------------------------------------K
BD5G13270        -------------------------------------------N
BD5G15530        ----------------------------------S--QPQHYTA
BD5G15540        ----------------------------------QV-QALKADS
GRMZM2G012236    -----------------------------------E-SLEHMVS
GRMZM2G015804    ------------------------------------------SS
GRMZM2G031660    ----------------------------------------LEAQ
GRMZM2G055699    ---------------------------T-----DDA-RSHAAEQ
GRMZM2G069024    ---------------------------L------DD-AGSHVEQ
GRMZM2G108133    -------------------------------------PDGAYSQ
GRMZM2G110948    ------------------------------------------TN
GRMZM2G112704    ------------------------------------------EN
GRMZM2G128197    TSNGPALLVSLLVSLGVL---------------LPS-IFMVSSV
GRMZM2G148176    -----------------------------------------KRS
GRMZM2G163544    -----------------------------------S-GSTVASS
GRMZM2G177661    ------------------------------------------SS
GRMZM2G376416    ---------------------------I----RSEN-QPHVSVL
```

```
GRMZM2G457040    ----------------------------------AA-QARRADS
Os10bglu34       ------------------------------------------SS
Os11bglu36       MEGLQDPLSCFIRCIFAPFSRQKKIHYI----E-DD-VISYSIN
Os12bglu38       ------------------------------------------EV
Os1bglu1         ------------------------------------------KN
Os1bglu2         --------------------------------------VLIAPQ
Os1bglu3         --------------------------------------LQIAQQ
Os1bglu4         --------------------------------------NKADMN
Os1bglu5         ---------------------------M------AA-AESRAQQ
Os3bglu6         -------------------------------------------T
Os3bglu7         -------------------------------------------H
Os3bglu8         ISSSHRLLLPLLVSLHFL---------------FPS-FFMFLSL
Os4bglu10        -------------------------------------------K
Os4bglu11        ---------------------------R----LKRV-AYNAFSN
Os4bglu12        -------------------------------------------K
Os4bglu13        -------------------------------------------K
Os4bglu14        ----------------------------------SQ-LHKFNSY
Os4bglu16        ----------------------------------QV-RSRRADS
Os4bglu18        ----------------------------------------HENQ
Os4bglu27        -------------------------------------------R
Os4bglu28        -------------------------------------------R
Os4bglu9         ---------------------------------PGG-RVCQYRH
Os5bglu19        ----------------------------------VS-TAFHAQL
Os5bglu21        ---------------------------F------VS-AASHAQL
Os5bglu22        ---------------------------P-----DEH-EHEHADQ
Os5bglu23        --------------------------------------IEKMLS
Os5gblu20        ---------------------------F------VS-AASHAQL
Os6bglu24        -------------------------------------------N
Os6bglu25        -----------------------------------------AII
Os7bglu26        -----------------------------------------KRN
Os9bglu29        -----------------------------------------KKY
Os9bglu30        -------------------------------------------H
Os9bglu31        ---------------------------------ALA-GGGAYSQ
Os9bglu32        ---------------------------------R---EDINPET
Os9bglu33        ---------------------------------P---EKSYATL
SB01G010825      TSNGAALLVSLFVSLCVL---------------LPS-VFMVSSV
SB01G010830      -------------------------------------------H
SB01G013360      -----------------R---------------APH-GMLLQTL
SB01G043030      ------------------------------------------SS
SB02G028400      -------------------------------------------R
SB02G029620      ---------------------------------------LLDRA
SB02G041550      -----------------------------------------KRS
SB03G037780      ---------------------------L------DD-AGSHVEQ
SB03G042690      --------------------------------------NKPDTN
SB06G019830      -----------------------------------------VLL
SB06G019840      -------------------------------------------K
SB06G019850      -------------------------------------------K
SB06G019860      -------------------------------------------K
```

```
SB06G019880    -------------------------------------------K
SB06G022410    -----------------------------------E-SPKHMVS
SB06G022420    -----------------------------------E-SPKHMVS
SB06G022450    -----------------------------------G-NSYQYTA
SB06G022460    ---------------------------------HYS-PQKISDM
SB06G022490    ----------------------------------EA-QARRADA
SB06G022500    ----------------------------------------HEAQ
SB06G022510    ----------------------------------------VEAQ
SB08G007586    ---------------------------N----K-IL-TPAGQLN
SB08G007650    ---------------------------N----K-IL-TPASLTN
SB09G018160    ----------------------------------AT-ATSHEQI
SB10G012220    -------------------------------------------E
SB10G022300    ---------------------------L-----GHG-HGSTYSE
SB10G027600    -----------------------------------S-GSTIASS
SB10G028060    ------------------------------------------ED
SbDhr1         ---------------------------N----K-IL-TPAGQLN
SbDhr2         ---------------------------S----EINK-IKVVTPA
Zm-p60.1       --------------------------------------KILTPA
ZmGLU2         --------------------------------------KIITPA
ZmGLU3         --------------------------------------KVLTPA
ZmGLU4         ------------------------------------------DK
ZmGLU5         --------------------------------------KILTPA
ZmGLU6         ----------------------------------------AGSA
ZmGLU7         ---------------------------D----MTGI--IPPSAY
ZmGLU8         ---------------------------D----KTGI-IPPPSAY
```
