# Supplementary material for: Combining Rational and Random Strategies in β-Glucosidase Zm-p60.1 Protein Library Construction
Source: PLoS One. 2014 Sep 26;9(9):e108292. doi: 10.1371/journal.pone.0108292 (PMC4178128; doi:10.1371/journal.pone.0108292)
Supplement: File S3 — Multiple sequence alignment (File S2, where position 1833 corresponds to the position of W373 in Zm-p60.1 or in File S3 position 18 in detailed view). (PDF) [file pone.0108292.s003.pdf]

Segment of an alignment of 167 β-glucosidases by T-coffee (version 9.01).
Position 18 corresponds to the W337 in the Zm-p60.1

```
                                 10        20        30        40
                        ....|....|....|....|....|....|....|....|..
AC155376.2_FGT005       S-GES-IGE--R-AASEW-L-----------VIVPWGLHKLL
AC211140.2_FGT007       N-GKP-IGD--R-ANSIW-L-----------YIVPSGIRKLM
AC217401.3_FGT001       N-GKP-IGP--Q-ANSKW-L-----------YIVPEGMYGCV
AC217687.3_FGT004       N-GKP-IGP--Q-ANSKW-L-----------YIAPTGMYGCV
AtBglu1                 ---------------------------------------GIL
AtBglu10                ------AAN--S--SFLL-W-----------EATPWGLEGIL
AtBglu11                ------VGN--TS-IENE-Y-----------ANTPWSLQQIL
AtBglu12                E-GVP-IGP--K-AASDW-L-----------LIYPKGIRDLL
AtBglu13                E-GVP-IGP--K-AASDW-L-----------LIYPKGIRDLL
AtBglu14                D----------------------------------GGIRDLI
AtBglu15                D-GVP-IGP--K-AASDW-L-----------LIYPKGIRDLV
AtBglu16                N-GVP-IGP--A-AGSDW-L-----------LIYPKGIRDLL
AtBglu17                N-GVP-VGE--P-TSADW-L-----------FICPEGFQDVL
AtBglu18                D-GYK-IGS--K-PFNGK-L-----------DVYSKGLRYLL
AtBglu19                DGSIK-IGS--Q-PNTAK-M-----------AVYAKGLRKLM
AtBglu2                 ------LGN--F--SGFG-Y-----------DVFPWAMESVL
AtBglu20                DKFNA-FAN--K-PDVAK-V-----------EVYAKGLRSLL
AtBglu21                D-HSA-IGS--M-PLTAA-L-----------PVYAKGFRKLL
AtBglu22                D-HSA-IGS--Q-PLTAA-L-----------PVYAKGFRSLL
AtBglu23                Q-NYA-IGS--K-PLTAA-L-----------NVYSRGFRSLL
AtBglu24                N-NIT-IGS--K-PETGP-L-----------PVYSTGFRKVL
AtBglu25                D-GFK-IGS--Q-PATAK-Y-----------PVCADGLRKVL
AtBglu26                D-GKQ-IAK--Q-GGSEW-S-----------FTYPTGLRNIL
AtBglu27                A-GQT-LGV--R-GGSEW-D-----------FLYPQGLRKFL
AtBglu28                S-NHQ-FGP--G-EDRGI-L-----------QSHPEGLRKVL
AtBglu29                S-NHE-TGP--G-DDRGK-I-----------HSHPEGLRRVL
AtBglu3                 ----V-LGN--F--SAFE-Y-----------AVAPWAMESVL
AtBglu30                S-GHI-IGP--G-EERGF-L-----------FSHPEGLRKVL
AtBglu31                T-GDT-ISL--ESDGTKI-L-----------WSYPEGLRKIL
AtBglu32                S-GDH-ISS--ESDGTKI-L-----------WSYPEGLRKLL
AtBglu33                N-NLS-LPD--LQTTSMG-I-----------VIYPAGLKNIL
AtBglu34                N-GVP-IGV--V-APS---F-----------VYYPPGFRQIL
AtBglu35                N-GSP-IGV--V-ASS---F-----------VYYPPGFRQIL
AtBglu36                N-GVS-IGV--K-ASI---N-----------FDV-KDLRHLV
AtBglu37                N-GQP-PGP--P-FSK-G-S-----------YYHPRGMLNVM
AtBglu38                T-GHA-PGP--P-FNA-A-S-----------YYYPKGIYYVM
AtBglu39                K-DGP-IGP--W-FNA-D-S-----------YYHPRGILNVL
AtBglu4                 ----D-FGK--S--LDFQ-Y-----------ANTPWAMEVVL
AtBglu40                G-LST-IGD--R-ASSIW-L-----------YIVPRGMRSLM
AtBglu41                G-GVA-IGE--R-AGSSW-L-----------HIVPWGIRKLA
AtBglu42                N-GDL-IGE--R-AASDW-L-----------YAVPWGIRKTL
AtBglu43                N-GTP-IGP--R-AHSEW-L-----------YNVPWGMYKAL
AtBglu44                L-GKP-IGP--R-AYSSW-L-----------YNVPWGMYKAL
AtBglu45                G-NVT-IGE--L-TDVNW-Q-----------HIDPTGFHKML
AtBglu46                G-NVS-IGE--L-TDVNW-Q-----------HIDPNGFRKML
AtBglu47                D-GLR-LG-------------------------EPVGMEEML
AtBglu5                 --------------MGAY-Y-----------PVAPWTMEAVL
AtBglu7                 ------AGN--A--SLFE-F-----------DAVPWGLEGIL
AtBglu8                 ------TGN--S--SSFV-F-----------DAVPWGLEGVL
AtBglu9                 ------TGN--S--SFLV-W-----------EATPWGLEGIL
BD1G10890               N-GIP-IGP--K-GE-VW--------------------KSSS
BD1G10920               N-GIP-IGP--K-ANSNW-L-----------YIVPTGMYGCV
BD1G10930               N-GKP-IGP--K-ANSDW-L-----------YIVPTGMYGCV
BD1G10940               N-GKP-IGP--L-ANSNW-L-----------YIVPTGMYGCV
BD1G19270               N-GVP-IGA--R-ANSYW-L-----------YIVPWGINKAV
BD1G33040               H-GKR-IGE--T-AASSW-L-----------HIVPWGMFKLM
```

```
BD1G42690       N-GTY-IGP--K-AGSSW-L-----------YIYPKGIEELL
BD1G70170       N-GKA-IGD--R-ANSIW-L-----------YIVPRGMRSLM
BD2G09190       ---------------------------------------STTGEEL
BD2G09200       --------------STTG-F-----------YVPGFGLQEVL
```

```
                          10        20        30        40
                 ....|....|....|....|....|....|....|....|..
BD2G27770        N-DSG-TGQ--F--IPIN-M-----------PDDPQGLQCML
BD2G37450        D-SPS-LSP--S-ASSPPSLGLSPASSNLPHALVPSSRSPLR
BD2G57640        T-GEG-IGE--R-AASEW-L-----------LIVPWGLRKAI
BD2G59650        T-DPP-SGQ--F--NPED-F-----------PNDPDGLQFVL
BD2G59660        T-DPP-VGK--Y--APTA-I-----------PNDPEGLQLMM
BD3G00650        D-GKP-IGD--K-ANSIW-L-----------YIVPGSMRSLM
BD3G40000        N-GVP-IGP--P-AFTPI-F-----------FNYPPGLRELL
BD3G40010        D-GVP-IGP--P-AYTPI-F-----------YNYPPGLRELL
BD3G45610        T-DPP-AGQ--G--VPTN-V-----------PSDPDGLHLVL
BD3G45630        ------------------------------------------
BD3G45640        T-GPP-AGQ--D--ERMS-L-----------F----------
BD3G45650        T-GPP-AGQ--G--APTN-V-----------PSDPKGLQLVL
BD4G08040        N-GVL-IGK--Q-AFSNW-L-----------YVVPWGFYKAV
BD4G09920        --------------------------------VYPDGLFRVL
BD4G34930        K-PFL-FG---L--KSDI-M-----------TSTPWALKKML
BD4G34940        -------D---NI-SKVQ-V-----------ETAPWSLSKLL
BD4G34950        -------K---DI-QEGH-L-----------ESAPWALGKLL
BD5G13260        N-GVA-IGP--Q-AASPW-L-----------YVYPPGFRELL
BD5G13270        N-GVP-IGP--Q-YASPG-L-----------YVYPQGLRDLL
BD5G15530        D-GVL-IGE--P-TGTPY-F-----------YDVPHGMEKVV
BD5G15540        D-GVL-IGK--P-TAFKG-Y-----------YDVPQGMEQAV
GRMZM2G012236    N-GIP-IGK--L-TQDAN-T-----------YVVPSSMEKLV
GRMZM2G015804    N-GKP-IGD--R-ANSIW-L-----------YIVPSGIRKLM
GRMZM2G031660    D-GIP-IGP--P-TAMPT-F-----------YVVPDGIEKMV
GRMZM2G055699    D-DPA-TGQ--F--VPIN-M-----------PSDPQGLQCML
GRMZM2G069024    T-DPP-IGQ--H--APTS-I-----------PADPRGLQLLV
GRMZM2G108133    N-QFP-FG---AL-TSDF-M-----------TSTPWALKKML
GRMZM2G110948    D-GVP-IGP--R-ANSDW-L-----------YIVPWGLYKAV
GRMZM2G112704    D-GVP-IGK--R-AYSDW-L-----------YVVPWGLYKAL
GRMZM2G128197    N-GVQ-IGQ--M-AHSIW-L-----------YIVPSGMYGVV
GRMZM2G148176    N-GVP-IGA--H-ANSYW-L-----------YIVPWGINKAV
GRMZM2G163544    H-GKK-IGE--T-AASGW-L-----------HIVPWGMFKLM
GRMZM2G177661    N-GKA-IGD--R-ANSIW-L-----------YIVPSGMRSLM
GRMZM2G376416    S-TVT-RNQQVPR-LGLR-N-----------HEAPWALSKLL
GRMZM2G457040    D-GVK-IGG--D-TALAG-F-----------FDVPEAIELAI
Os10bglu34       N-GQP-IGD--R-ANSIW-L-----------YIVPRSMRILM
Os11bglu36       --------------------------------VYPDGLFRIL
Os12bglu38       N-GVP-IGK--Q-AYSNW-L-----------YVVPWGIYKAV
Os1bglu1         D-GVP-IGP--R-ANSDW-L-----------YIVPWGLYKAV
Os1bglu2         T-DPP-TEK--Y--EPTE-Y-----------PNDPKGLQLAL
Os1bglu3         Q-GVRFIAQ-TMQ-APTR-S-----------MGDPHGLQLML
Os1bglu4         T-GEK-IGE--R-AASEW-L-----------FIVPWGLRKLL
Os1bglu5         T-DPP-PGK--A--APTS-I-----------GPDPQGLRLMV
Os3bglu6         N-GKP-IGD--R-ANSIW-L-----------YIVPRGMRSLM
Os3bglu7         N-GKP-IGP--Q-ANSNW-L-----------YIVPWGMYGCV
Os3bglu8         N-GVP-IGQ--Q-ANSNW-L-----------YIVPTGMYGAV
Os4bglu10        N-GIP-IGP--Q-AASFW-F-----------YIYPEGLRELL
Os4bglu11        N-GIP-IGP--Q-AASFW-F-----------HIYPEGICEML
Os4bglu12        N-GIP-IGP--Q-AASPW-L-----------YVYPQGFRDLL
Os4bglu13        N-GIP-IGP--Q-AASPW-L-----------YIYPQGFRELV
Os4bglu14        D-GVP-IGK--A-TGAPF-F-----------HDVPRGMEEAV
Os4bglu16        N-GKI-IGK--P-TALHG-Y-----------FVVPEAMEKVV
Os4bglu18        N-GLP-IGT--P-TAMPT-F-----------YVVPDGIEKMV
Os4bglu27        N-GVP-IGP--P-AFTKI-F-----------FTYAPGLRELL
Os4bglu28        N-SIP-IGP--P-AYTPI-F-----------FNYPPGLRELL
Os4bglu9         ------------------------------------------
Os5bglu19        N-STP-TRE---F--LPG-T-----------SLDPRGLELAL
Os5bglu21        ----------------PG-T-----------IVDPRGLEHAL
Os5bglu22        N-DTP-SGQ--F--VPTR-L-----------PRDPKGLQCML
Os5bglu23        N-DTP-SDK--C--LSIY-Q----------------------
```

```
                         10        20        30        40
               ....|....|....|....|....|....|....|....|..
Os5gblu20      N-DTP-TPE---F--LPG-N-----------TVDPQGLENAL
Os6bglu24      N-GTD-IGP--K-AGSSW-L-----------YIYPKGIEELL
Os6bglu25      H-GKK-IGE--T-AASSW-L-----------HIVPWGMFKLM
Os7bglu26      N-GVP-IGA--Q-ANSYW-L-----------YIVPWGINKAV
Os9bglu29      N-GKP-IGP--Q-EFTPI-F-----------FNYPPGLRELL
Os9bglu30      N-GKP-IGP--Q-EFTPI-F-----------FNYPPGLRELL
Os9bglu31      N-EFP-LG---L--TSDF-M-----------TSTPWALKKML
Os9bglu32      -------G---GF-DKEH-Y-----------QLHPWALGKML
Os9bglu33      -----------ED-DTEN-I-----------QCHSWSLGKVL
SB01G010825    N-GVQ-IGQ--L-AHSVW-L-----------YIVPTGMYGVV
SB01G010830    N-GKP-IGP--Q-ANSNW-L-----------YIVPSGMYGCV
SB01G013360    N-GKP-IGD--R-ANSIW-L-----------YIVPGSMRSLM
SB01G043030    N-GKP-IGD--R-ANSIW-L-----------YIVPSGIRKLM
SB02G028400    D-GVP-IGT--P-EFVPI-F-----------FEYPQGLRELL
SB02G029620    F-PIR-LN---SL-TIDF-K-----------TSKPWALKKLL
SB02G041550    N-SIP-IGA--H-ANSYW-L-----------YIVPWGINKAV
SB03G037780    T-DPP-VGQ--H--APTS-I-----------PADPRGLQLMV
SB03G042690    S-GEK-IGE--R-AASEW-L-----------FIVPWGLRKVL
SB06G019830    G-GLP-IGR--Q-AASPS-L-----------YIYPQGFLELL
SB06G019840    N-GVP-IGP--Q-AASSW-L-----------YIYPQGFRDLL
SB06G019850    V-SQL-VLR--L-HRSPW-L-----------FIYPRGFRELL
SB06G019860    N-GVP-IGP--Q-AASSW-L-----------FIYPRGFRELL
SB06G019880    N-GVP-IGP--Q-AASSW-L-----------FMYPQGFRELL
SB06G022410    N-GIP-IGK--P-TPVAN-N-----------YVVPSSMEKLV
SB06G022420    N-GIL-IGK--P-TPVAN-T-----------CVVPSSMEKLV
SB06G022450    D-GIL-IGE--R-TGSPY-L-----------NVVPYGMEKVV
SB06G022460    D-GIL-IGE--R-TGSPY-I-----------NTVPYGIEKVV
SB06G022490    D-GVK-IGR--D-TALSG-F-----------YDVPEAIEPAI
SB06G022500    N-GIP-IGP--P-TAMPL-F-----------FDVPDGIEKMV
SB06G022510    N-GIP-IGP--P-TAMPK-F-----------YFVPDGIEKMV
SB08G007586    D-GNA-IGP--P-TGNAW-I-----------NMYPKGLHDIL
SB08G007650    ----------------AW-I-----------NMYPKGLHDIL
SB09G018160    D-GPA-AIQ---Y--PAG-T-----------MVDPQGLEHVL
SB10G012220    N-GTV-IGP--K-AGSPW-L-----------YIYPKGIEELL
SB10G022300    S-TVR---NQEPQ-LGLR-N-----------KEAPWALNKVL
SB10G027600    H-GKK-IGD--T-AASGW-L-----------HIVPWGMFKLM
SB10G028060    --------------YSDW-L-----------YVVPWGLYKAL
SbDhr1         D-GNA-IGP--P-TGNAW-I-----------NMYPKGLHDIL
SbDhr2         N-GNT-IGP--A-TGNAW-V-----------YMYPKGLKDIL
Zm-p60.1       D-GKP-IGP--P-MGNPW-I-----------YMYPEGLKDLL
ZmGLU2         D-GKP-IGP--P-MGNPW-I-----------YLYPEGLKDIL
ZmGLU3         D-GNS-IGP--P-MGNPW-I-----------YMYPKGLKDLL
ZmGLU4         D-GEP-IGP--P-MG-NW-I-----------YLYPQGLKDLL
ZmGLU5         D-GNP-IGP--W-MGNSW-I-----------YLYPEGLKDLL
ZmGLU6         D-DKP-IGP--W-MGNPW-I-----------YMYPDGLKDLL
ZmGLU7         D-GNT-IGP--S-MGNPW-I-----------YMYPNGLKDIL
ZmGLU8         D-GNT-IGP--S-MGNSW-I-----------YMYPNGLKDIL
```
