# Supplementary material for: Combining Rational and Random Strategies in β-Glucosidase Zm-p60.1 Protein Library Construction
Source: PLoS One. 2014 Sep 26;9(9):e108292. doi: 10.1371/journal.pone.0108292 (PMC4178128; doi:10.1371/journal.pone.0108292)
Supplement: File S4 — The protein variants expression and purification details. (DOC) [file pone.0108292.s004.doc]

Supplemental Results S4_purification

Proteins were purified as previously described [1].

An ÄKTA FPLC system equipped with UV and conductivity detectors, and an autosampling unit (GE Healthcare; Chalfont S Giles, UK), was used for all chromatography steps. Each bacterial ﬁltrate was applied to a 5 mL HisTrap IMAC HP column charged with Ni ^2+^ions (GE Healthcare, Chalfont St Giles, UK) equilibrated with ﬁve column volumes of buffer A1 [0.02 M Tris–HCl (pH 7.9), 1 M NaCl] and buffer B [0.02 M Tris–HCl (pH 7.9), 1 M NaCl, 0.05 M imidazole] mixed to a ﬁnal imidazole concentration of 0.02 M imidazole. The ballast proteins were washed out with one column volume of buffer mixture A1 + B (0.02 M imidazole) and three column volumes of buffer mixture A1 + B with a linear gradient (0.02–0.05 M) of imidazole. The recombinant enzyme was then eluted with buffer A2 [0.02 M Tris–HCl (pH 7.9), 1 M NaCl, 0.1 MEDTA]. Fractions containing recombinant β-D-glucosidase activities were pooled, desalted and concentrated to a ﬁnal volume of 1.5 mL by ultraﬁltration (using Amicon Ultra-15, 30 kDa cut-off membranes; Millipore, Bedford, MA, USA). Crude enzyme solution was applied to a HiLoad 16/60 Superdex 200 prep grade column (GE Healthcare Bioscience; Uppsala, Sweden) equilibrated with 1.5 column volumes of buffer A [0.05 M Tris–HCl (pH 7), 0.5 M NaCl], the recombinant enzyme was then eluted isocratically with buffer A. Enzyme-containing fractions were pooled, desalted and their volumes were reduced to 80–150 μL by ultraﬁltration (using Amicon Ultra-4, 10 kDa cut-off membranes; Millipore, Bedford, MA, USA). Puriﬁed enzymes were electrophoretically separated in a 10% (w/v) SDS–PAGE gel, followed by staining with BioSafe Coomassie (Bio-Rad Laboratories, Hercules, CA, USA). The purity of the enzymes was conﬁrmed using a GS-800 Calibrated Densitometer (Bio-Rad Laboratories, Hercules, CA, USA).

To ensure the purity of proteins in the saturation mutagenesis project we improved the standard procedure by precisely comparing the W373H mutant and WT elution profiles and identifying contaminants by mass spectrometry in both FPLC and SDS-PAGE fractions.

The gel filtration chromatography elution profile (FPLC chromatogram) of wild-type β-glucosidase Zm-p60.1 clearly shows peaks corresponding to dimeric and monomeric forms of the enzyme (Fig. S4-1). Only fractions containing dimer were collected, further reducing possible impurities and enabling work with protein in the biologically active conformation. Four fractions from the center of the peak were pooled, desalted and concentrated. Puriﬁed wild-type β-glucosidase Zm-p60.1 and mutant W373H were electrophoretically separated in a 10% (w/v) SDS–PAGE gel, followed by staining with BioSafe Coomassie (Bio-Rad Laboratories, Hercules, CA, USA) (Fig. 2). The purity of the enzymes was conﬁrmed using a GS-800 Calibrated Densitometer (Bio-Rad Laboratories, Hercules, CA, USA), which indicated that it was approximately 97.6% (Fig. S4-2).

The mass spectrometry analysis identified only one detectable contaminant in the molecular weight range of the Zm-p60.1 dimer: a dimeric form of Glutamine-fructose-6-phosphate aminotransferase (GlmS). This enzyme is a typical trace contaminant of preparations obtained from procedures involving immobilized metal ion affinity chromatography (IMAC) of proteins tagged with a poly-histidine sequence using *E. coli* BL21(DE3) as a production strain [2]. The amount of this contaminant is generally about 2%, depending partly on the GlmS dimer’s stability. Exactly the same protocol was used to express and purify the other mutants, including use of the *E. coli* BL21(DE3) production strain and FPLC fraction selection and processing.


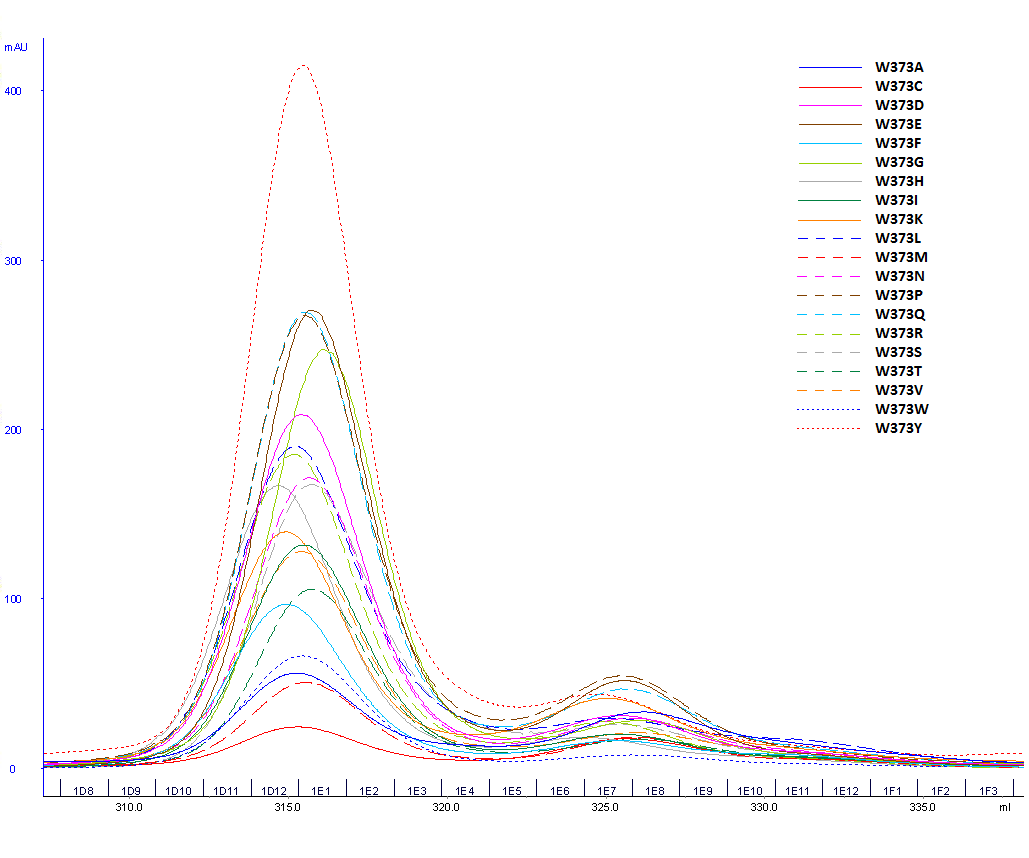


Dimeric form

Monomeric form

Fig. S4-1 - Gel filtration elution profiles from the Äkta HPLC system for all mutants. Only active dimeric fractions were collected (columns 1D11-1E1) and used in subsequent experiments. Differences in heights of the peaks reflect the variability of protein expression in *E. coli* BL21(DE3)pLysS.


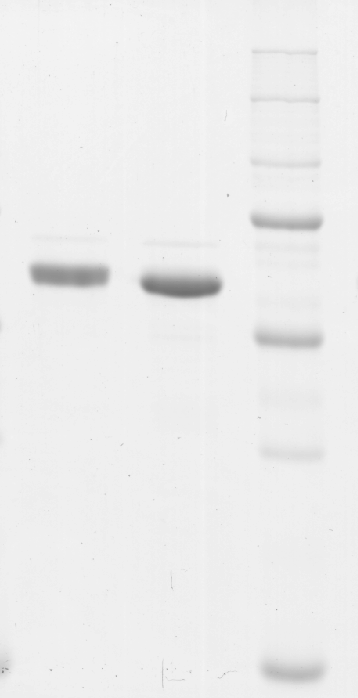


25 kDa

37 kDa

50 kDa

75 kDa

100 kDa

150 kDa

250 kDa

1 2 3

GlmS

Fig. S4-2 - Results of 10% SDS PAGE separation of purified enzymes after gel filtration. Minor bands correspond to the contaminant Glutamine-fructose-6-phosphate aminotransferase (GlmS). Line 1 – Mutant W373H. Line 2 – WT β-glucosidase Zm-p60.1. Line 3 – SDS marker. 1 µg of total protein was loaded into the well.

SUPPLEMENTAL REFERENCES

1. Filipi T, Mazura P, Janda L, Kiran NS, Brzobohatý B (2012) Engineering the cytokinin-glucoside specificity of the maize β-D-glucosidase Zm-p60.1 using site-directed random mutagenesis. Phytochemistry 74: 40–48.

2. Robichon C, Luo J, Causey TB, Benner JS, Samuelson JC (2011) Engineering *Escherichia coli* BL21(DE3) derivative strains to minimize *E. coli* protein contamination after purification by immobilized metal affinity chromatography. Appl Environ Microbiol 77: 4634–4646.
